# Local effects of non-pharmaceutical interventions on mitigation of COVID-19 spread through decreased human mobilities in Japan: a prefecture-level mediation analysis

**DOI:** 10.1038/s41598-024-78583-0

**Published:** 2024-11-06

**Authors:** Shohei Nagata, Yuta Takahashi, Hiroki M. Adachi, Glen D. Johnson, Tomoki Nakaya

**Affiliations:** 1 Co-creation Center for Disaster Resilience, International Research Institute of Disaster Science, Tohoku University, 468-1 Aoba, Aramaki, Aoba-ku, Sendai, 980-0845 Japan; 2 Graduate School of Environmental Studies, Tohoku University, 468-1 Aoba, Aramaki, Aoba- ku, Sendai, 980-0845 Japan; 3 Department of Environmental, Occupational and Geospatial Health Sciences, City University of New York School of Public Health, 55 West 125th Street, New York, NY 10027 USA; 4 Department of Earth Science, Graduate School of Science, Tohoku University, 6-3 Aoba, Aramaki, Aoba-ku, Sendai, 980-8578 Miyagi Japan

**Keywords:** Infectious diseases, Health policy, Public health

## Abstract

To control the COVID-19 epidemic, the Japanese government and the local governments have repeatedly implemented non-pharmaceutical interventions (NPIs) throughout 2020–2022. Using Bayesian state-space mediation models, we examined the effect of repeated NPIs on infection spread mitigation, mediated by human mobility changes in each prefecture during three epidemic phases: from April 1, 2020 to February 28, 2021; from March 1, 2021 to December 16, 2021; and from December 17, 2021 to December 31, 2022. In the first phase, controlling downtown populations at nighttime was effective in mitigating the infection spread in almost all prefectures. In the second and third phases, the effect was not clear, especially in metropolitan prefectures. Controlling visitors from the central prefectures of metropolitan areas was effective in mitigating infection spread in the surrounding prefectures during all phases. These results suggest that the local spread of infection can be mitigated by focusing on nighttime human mobility control in downtown areas before the epidemic spreads widely and transmission routes become more diverse, and that the geospatial spread of infection can be prevented by controlling the flows of people from large cities to other areas.

**Supplementary Information:**

The online version contains supplementary material available at 10.1038/s41598-024-78583-0.

## Introduction

Throughout the pandemic stage of the novel SARS-CoV-2 coronavirus and its human symptoms, collectively referred to as COVID-19 disease, many countries implemented non-pharmaceutical interventions (NPIs) to control human mobility with the intent of reducing human-to-human transmission of this highly infectious airborne respiratory virus. These NPIs included international and internal travel restrictions, school and workplace closures, public event cancellation, and public transport closure^1,2^. As a result of these interventions, human mobility was largely restricted worldwide. Analysis of human mobility data from 52 countries found that, as of March 11, 2020, human mobility had decreased by 63% compared to pre-pandemic levels^3^. Associations between changes in human mobility and infection trends during NPIs have been reported by an abundance of studies from various countries including China^4^, Italy^5^, the USA^6^, and Japan^7,8^. These associations were particularly consistent and robust in the early stages of the pandemic^3^.

**Fig. 1:**
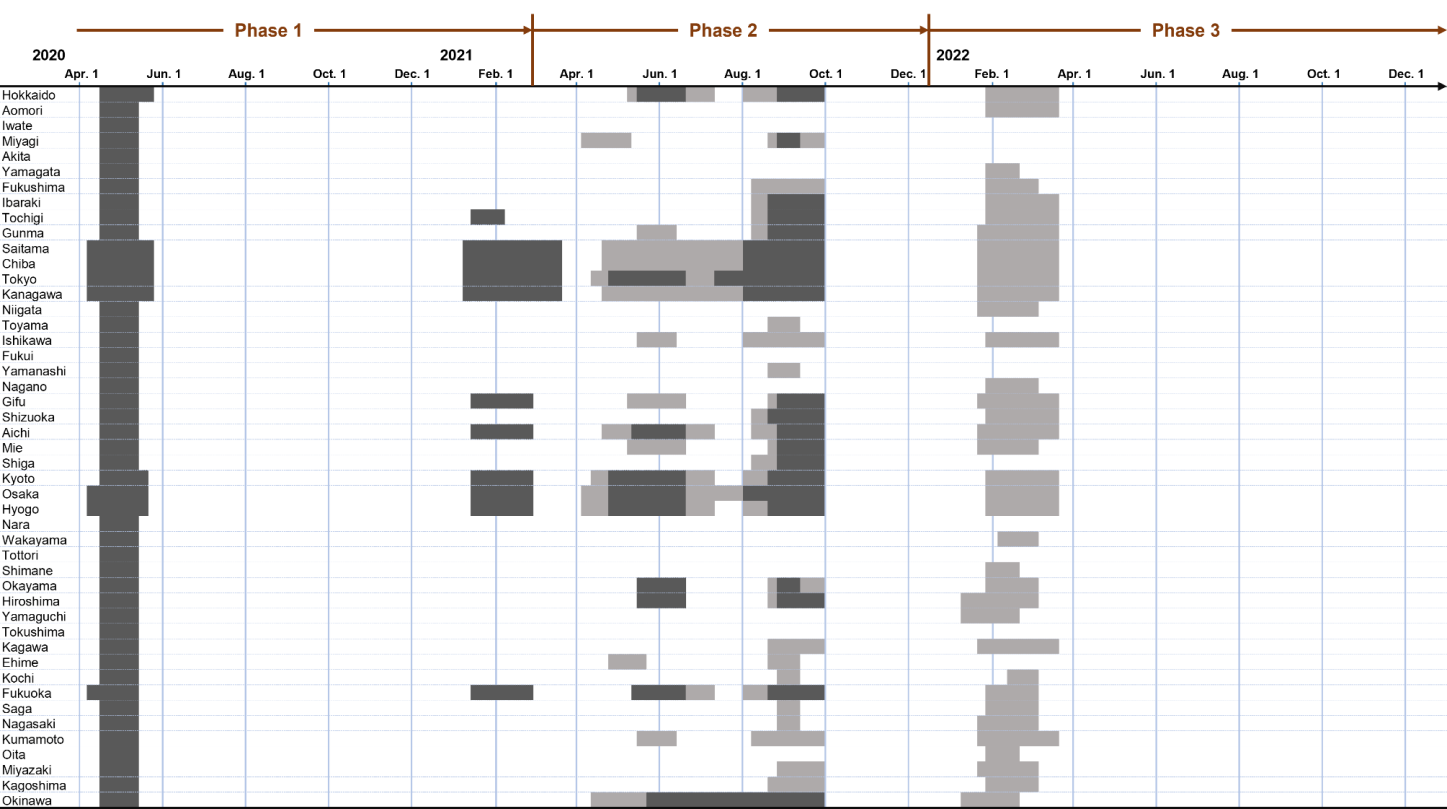
Implementation period of non-pharmaceutical interventions by prefecture. The dates of State of Emergency declarations and Priority Preventative Measures are shown in dark gray and light gray, respectively.

In Japan, NPIs to control human mobility were implemented as State of Emergency declarations (SoEs) and Priority Preventative Measures (PPMs) between 2020 and 2022 at the prefectural level (Fig. 1)^9^. Although the NPIs for implementing the measures varied depending on the timing and scale of infection in each prefecture, the national and local governments generally requested restaurants and bars to operate for shorter hours or to close, while also requesting residents to stay at home or avoid visiting high-risk areas during the periods of the SoEs and the PPMs^10^. For example, Tokyo implemented four SoEs (April 7, 2020 to May 25, 2020, January 8, 2021 to March 21, 2021, April 25, 2021 to June 20, 2021, and July 12, 2021 to September 30, 2021) and three PPMs (April 12, 2021 to April 24, 2021, June 21, 2021 to July 11, 2021, and January 21, 2022 to March 21, 2022). Although there was no legal mandate, residents were requested to refrain from unnecessary outings, especially at night, and unnecessary travel to other prefectures^11,12^.

When the first SoE was implemented in April-May 2020 for all prefectures, human mobility was clearly reduced despite this intervention not being legally binding^7,8,13,14^. Previous studies showed that overall mobility in Tokyo was almost halved during the first SoE^7,13^, with approximately 80% of mobility reduced in densely populated areas and nightlife districts^8,13,14^. The first SoE also significantly reduced inter-prefectural travels, particularly within the Tokyo metropolitan area^13^. Further evidence based on mobility at nightlife districts in the metropolitan areas^8^and travel sizes between prefectures^15^during the first SoE supported that this human mobility reduction was effective for mitigating the spread of the COVID-19 infection in the early stage of the epidemic in Japan, as with the global trend. Another study reported relatively stable relationships between changes in nighttime human mobility in downtown areas and infection spread in the major metropolitan areas of Japan throughout the epidemic period from February 2020 to May 2022^16^.

Although these previous studies have shown positive associations between human mobility and infection growth rates, it is still unclear whether implementing NPIs served as a trigger for reduced human mobility, thereby contributing to infection spread mitigation throughout the multiple waves of the epidemic in Japan. It has been reported that the degree of human mobility reduction during the NPIs became smaller as the NPIs were repeatedly implemented due to self-restraint fatigue^17,18^, indicating a possibility that the NPIs to control human mobility to restrain the infection growth rate became ineffective after the first SoE. Despite such changes in the epidemic situation with the emergence of new strains of SARS-CoV-2 and the introduction of vaccines, few studies have attempted to assess whether the repeated NPIs were effective in reducing spread of infection via reduction of human mobility. In addition, previous studies on the human mobility control in Japan are biased toward metropolitan areas.

In summary, our review of past investigations focused on human mobility control for mitigating the spread of infection in Japan reveals two main research gaps: (1) Existing knowledge is predominantly based on insights gained during the early stages of the pandemic, leaving it unclear whether the effects of human mobility control persisted as the pandemic situation changed; and (2) It remains uncertain whether human mobility control contributed to mitigating the spread of infection across broader areas of Japan, including regions beyond the major metropolitan areas. Our study presented here examined whether the NPIs that were implemented repeatedly between 2020 and 2022 contributed to mitigating the spread of infection through human mobility reduction in each prefecture in Japan by using a mediation analysis framework.

## Methods

We assessed the effect of the NPIs on mitigating the infection growth rate, mediated by the changes in human mobility for each combination of epidemic phase and prefecture. Therefore, data processing and analysis methods described in this section were applied to each of those combinations. We defined the epidemic phases based on reports from the Osaka Institute of Public Health and the National Institute of Infectious Diseases^19,20^; whereby the first epidemic phase was from April 1, 2020 to February 28, 2021, the second phase was from March 1, 2021 to December 16, 2021, corresponding to the Alpha and Delta variants’ waves, and the third phase was from December 17, 2021 to December 31, 2022, corresponding to the Omicron variant’s wave.

### Indicator of the NPIs implementation

We referred to information on the SoEs and PPMs published by the Japanese Cabinet Secretariat^9^, and organized the information into daily binary variables for statistical analysis described below (1 if the day was included in the NPIs period, 0 if the day was NOT included in the NPIs period). The national and local governments implemented either the SoEs or PPMs during the pandemic, depending on the scale of infection within each prefecture. These two measures have never been issued simultaneously within a single prefecture. Although the specific actions for each measure differed by epidemic phase and prefecture, the common goal was to reduce human mobility under the issuance of the SoEs and PPMs^11,12^. Therefore, this study did not distinguish between the SoEs and PPMs, instead regarding them collectively as NPIs for human mobility control.

### Indicator of human mobility change

To observe the changes in human mobility in each prefecture during each epidemic phase, we used “Mobile Spatial Statistics” (DOCOMO InsightMarketing, Inc., Tokyo, Japan), which provided the estimated hourly population in approximately 500 m grid cells by gender, age-group, and prefecture of residence based on cell phone networks of NTT DOCOMO, Inc., the largest cell phone company in Japan^21^. We summed the populations of the 500-meter grid cells according to the conditions described below to determine the population change in each prefecture. The data provided included both resident and visitor populations. The data provider estimated individuals’ prefecture of residence based on their daily location distribution. Since there are no data that show actual population dynamics, it is difficult to assess the accuracy of hourly population estimates. However, according to a technical report from the data provider, when the population is aggregated at the prefectural level, the deviation between the midnight population estimate and the census-based resident population is within 10%^22^.

During the SoE or PPM periods, the governments requested that businesses identified as having a high risk of infection, such as restaurants and bars, close either at night or throughout the day. In addition, they asked residents to refrain from visiting crowded areas and to avoid non-essential travel to other prefectures^10^. To quantitatively evaluate the effectiveness of these requests, we focused on the daily population in downtown areas at daytime and nighttime, along with the number of visitors from other prefectures. In addition, we also observed the number of visitors from Tokyo, Aichi, and Osaka Prefectures which are the centers of the three largest metropolitan areas in Japan. The downtown areas in each prefecture were identified based on hourly population by gender and age-group in each grid before the pandemic. The detailed area classification method is provided in the supplement (*Supplement 1*).

Human mobility change by area classifications, such as the downtown population and the number of visitors, was calculated as the ratio of the daily population to the baseline population in each grid from April 2020 to December 2022^8^. To remove day-of-the-week effects, a 7-day moving average was calculated. We then used natural logarithms to stabilize variation. Specifically, the human mobility change indicator $\:M$ of each setting $\:s$ on day $\:t$ can be defined as follows:

$$\:{M}_{s,t}=\text{ln}\left(\frac{{\sum\:}_{d=t-6}^{t}{m}_{s,d}}{7}\right),$$

$$\:{m}_{s,d}=\frac{{P}_{s,d}}{{B}_{s}},$$

where $\:{P}_{s,d}$ is population of setting $\:s$ on day $\:d$ and $\:{B}_{s}$ is the baseline population of setting $\:s$, taken as the median population of each setting from January 3 to February 6, 2020, as with a previous study^8^. The population settings assigned to $\:s$ are downtown population at daytime (8:00 AM-5:59 PM), downtown population at nighttime (8:00 PM-11:59 PM), visitor population from all other prefectures, visitor population from Tokyo, visitor population from Aichi, and visitor population from Osaka. The term$\:\:{m}_{s,d}$ represents the ratio of the daily population $\:{P}_{s,d}$ to the baseline population $\:{B}_{s}$. Note that for the four visitor population settings, we used the overall population of each prefecture, not just the downtown area.

### Indicator of the COVID-19 infection growth

We used the number of daily reported positive cases per prefecture, published as open data by the Japanese Ministry of Health, Labour and Welfare, to calculate the daily effective reproduction number representing the average number of secondary cases per infectious case at day $\:t$. Following previous studies^23,24^, this was measured as

$$\:{\widehat{R}}_{t}=\frac{1+{\sum\:}_{d=t-6}^{t}{I}_{d}}{1+{\sum\:}_{d=t-g-6}^{t-g}{I}_{d}},$$

where $\:{\widehat{R}}_{t}$ estimates the effective reproduction number on day *t*, $\:{I}_{d}$ is the number of reported positive cases on day $\:d$, and $\:g$ is the constant generation time. We set the constant $\:g$to 5, following a report from the National Institute of Infectious Diseases of Japan^24^. We added 1 to the denominator and numerator to avoid zero division for the periods when positive cases are not reported for several consecutive days.

In our preliminary analysis, using $\:{\widehat{R}}_{t}$ per day, we encountered difficulties with converging parameter estimates in the statistical analysis described below due to large fluctuations in values, especially during the early stages of the epidemic. We therefore chose to calculate the final response variable for infection growth, $\:{y}_{t},$ as the logarithm of a 7-day moving average of $\:{\widehat{R}}_{t}\:$to exclude day-of-week bias and stabilize variation, similar to previous studies^8,16^ and how data was presented to the public globally throughout much of the pandemic (for example, https://github.com/nytimes/covid-19-data). More specifically, this is expressed as follows:

$$\:{y}_{t}=\text{ln}\left(\frac{{\sum\:}_{d=t-6}^{t}{\widehat{R}}_{d}}{7}\right)$$

### Statistical analysis

**Fig. 2:**
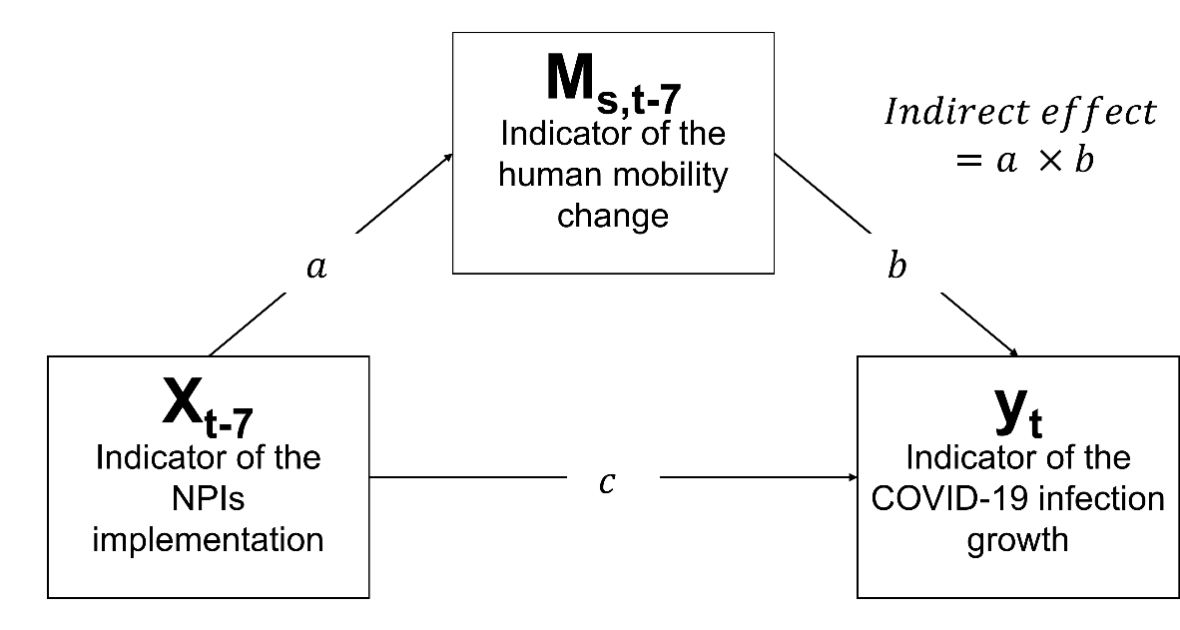
Mediation model for evaluation of the effect of the NPIs implementation on the infection growth mitigation.

In order to test the hypothesis that implementing NPIs reduced infection growth at least partially by reducing human mobility, we specified a mediation pathway that is diagrammed in Fig. 2^25^, where *x* indicates implementation of an NPI, *M* is the human mobility change and *y*is infection growth as described above. A similar framework was used in a study evaluating the effectiveness of an intervention in mitigating COVID-19 infection in Switzerland^26^. Since the indicator of infection growth was defined based on the date of report, not the date of onset, we assumed a seven-day lag in the effect of NPI implementation and the changes in human mobility on infection growth, based on previous studies^16,27,28^. The parameter $\:a$ represents the effect of NPI implementation on human mobility change, while parameter $\:b$ represents the effect of human mobility change on infection growth and the parameter $\:c$ represents the direct effect of NPI implementation on infection growth that is not mediated by the human mobility changes. The effect of mitigating the spread of infection by factors other than human mobility, such as increased mask use and enhanced social distancing practices during the NPIs, is adjusted by parameter *c*. Given this specification, human mobility change is considered to be a mediating variable if the indirect effect, expressed as $\:a\:\times\:\:b$, is statistically significantly negative under the condition that $\:a$ is negative and $\:b$ is positive.

We estimated the parameters as a Bayesian state-space model as follows:

$$\begin{aligned}{y}_{t}=&\:{\mu\:}_{t}+b{\widehat{M}}_{s,t-7}+{cx}_{t-7}+{\zeta\:}_{t} \\ =&\:{\mu\:}_{t}+b\left({\theta\:}_{s,t-7}+{ax}_{t-7}\right)+{cx}_{t-7}+{\zeta\:}_{t}, \\ {M}_{s,t}=&\:{\theta\:}_{s,t}+{ax}_{t}+{\delta\:}_{t}, \\ {\theta\:}_{s,t}=&\:{\theta\:}_{s,t-7}+{\gamma\:}_{t}, \\ {\mu\:}_{t}=&\:{\mu\:}_{t-7}+{\epsilon\:}_{t}, \end{aligned}$$

where $\:{x}_{t}$, $\:{M}_{s,t}$, and $\:{y}_{t}$ correspond to the variables in Fig. 2. The parameters $\:{\theta\:}_{s,t}$ and $\:{\mu\:}_{t}$ represent the state of human mobility and the regional infection growth, respectively, that are not affected by the NPI implementation at *t* and are not directly observed through the data. Note that $\:{\theta\:}_{s,t}$ and $\:{\mu\:}_{t}$ are assumed to be dependent on their seven-day prior states. This expresses the temporal trends of the states over longer periods than the moving average interval of the past six days to compute $\:{M}_{s,t}$ and $\:{y}_{t}$. Additionally, $\:{\gamma\:}_{t}$, $\:{\delta\:}_{t}$, $\:{\epsilon\:}_{t}$, and $\:{\zeta\:}_{t}$ are the independent and identically distributed white noise terms such that $\:{\gamma\:}_{t}\sim{}_{iid}N\left(0,{\sigma\:}_{\gamma\:}^{2}\right)$, $\:{\delta\:}_{t}\sim{}_{iid}N\left(0,{\sigma\:}_{\delta\:}^{2}\right)$, $\:{\epsilon\:}_{t}\sim{}_{iid}N\left(0,{\sigma\:}_{\epsilon}^{2}\right),$ and $\:{\zeta\:}_{t}\sim{}_{iid}N\left(0,{\sigma\:}_{\zeta\:}^{2}\right)$. The mobility estimate, $\:{\widehat{M}}_{s,t-7}$ used to estimate $\:{y}_{t}$ represents the unobserved state of human mobility affected by NPI implementation at $\:t-7$. Note that the population data used in this study was not based on information from a single point in time but was updated daily, therefore incorporating stochastic forces like weather that are captured by the random error terms in the model. Consequently, there was no need to incorporate such factors as separate effects in the model. We computed the posterior distributions of $\:a$, $\:b$, and the indirect effect by full Bayesian estimation using Stan (Stan Development Team, https://mc-stan.org/), and considered the indirect effect significant if the 95% credible interval (CI) of the posterior distribution did not contain zero. To estimate the posterior distribution of the indirect effect and parameters, we employed the Hamiltonian Monte Carlo, a variant of Markov Chain Monte Carlo method, performed by using R 4.0.2 and Rstan 2.21.7. Convergence of the posterior distribution was considered acceptable if the Rhat diagnostic indicator was less than 1.1^29^. If the distributions of either $\:a$, $\:b$, or the indirect effects did not converge at 400,000 iterations, the model was considered un-evaluable. We set the burn-in period to be one-fifth of the total number of iterations and the thinning interval to six.

To evaluate the effect of implementing NPIs by prefecture and epidemic phase, we categorized the results of estimated parameter combinations into the following five types: “A” for the cases of statistically significant negative indirect effect with negative $\:a$ and positive $\:b$ estimates (i.e. successful reduction of epidemic growth via mobility reduction, as designed) ; “B” for the cases of statistically significant positive indirect effect with negative $\:a$ and negative $\:b$ estimates (i.e. mobility decreased but infection growth unsuccessfully increased); “C” for the cases of statistically significant positive indirect effect with positive $\:a$ and positive $\:b$ estimates (i.e. unsuccessfully increased mobility and infection growth); “D” for the cases of statistically significant negative indirect effect with positive $\:a$ and negative $\:b$ estimates (i.e. mobility increased but infection growth decreased) and “E” for the cases of non-significant indirect effect. The classification results were visualized by prefecture and epidemic phase through a series of maps.

## Results

**Fig. 3:**
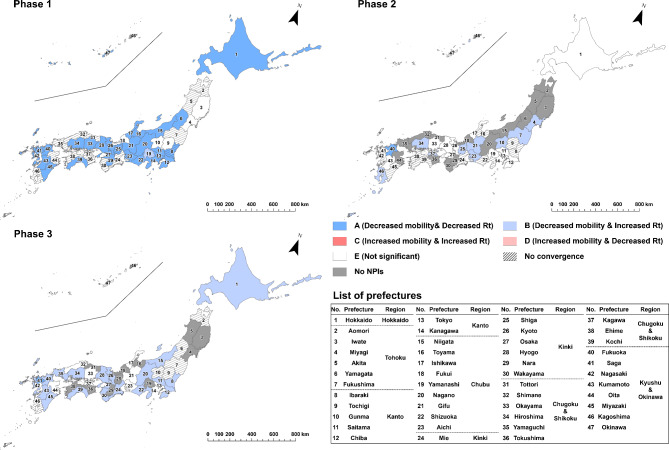
Indirect effects of implementing NPIs on COVID-19 infection growth, mediated by reduction in daytime human mobility in downtown areas. “A,” represented by dark blue, indicates prefectures with a negative indirect effect characterized by negative *a* and positive *b* estimates (i.e., successful reduction of epidemic growth via mobility reduction, as designed); “B,” represented by light blue, indicates prefectures with a positive indirect effect characterized by negative *a* and negative *b* estimates (i.e., mobility decreased, but infection growth unsuccessfully increased); “C,” represented by dark red, indicates prefectures with a positive indirect effect characterized by positive *a* and positive *b* estimates (i.e., both mobility and infection growth unsuccessfully increased); “D,” represented by light red, indicates prefectures with a negative indirect effect characterized by positive *a* and negative *b* estimates (i.e., mobility increased, but infection growth decreased); and “E,” represented by white, indicates prefectures with a non-significant indirect effect. The numbers on the maps correspond to the numbers in the list of prefectures.

**Fig. 4:**
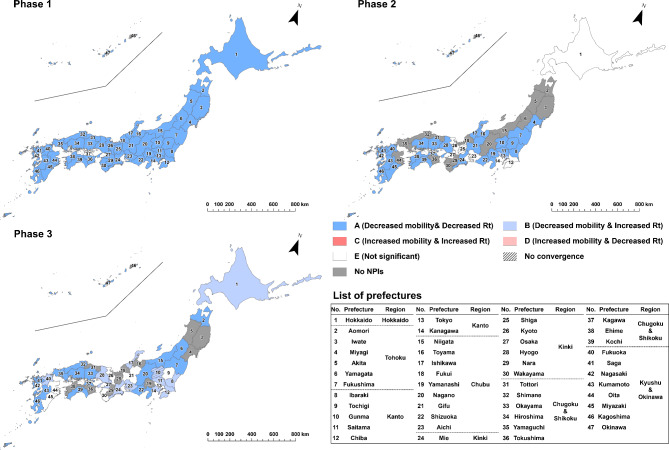
Indirect effects of implementing NPIs on COVID-19 infection growth, mediated by reduction in nighttime human mobility in downtown areas. “A,” represented by dark blue, indicates prefectures with a negative indirect effect characterized by negative *a* and positive *b* estimates (i.e., successful reduction of epidemic growth via mobility reduction, as designed); “B,” represented by light blue, indicates prefectures with a positive indirect effect characterized by negative *a* and negative *b* estimates (i.e., mobility decreased, but infection growth unsuccessfully increased); “C,” represented by dark red, indicates prefectures with a positive indirect effect characterized by positive *a* and positive *b* estimates (i.e., both mobility and infection growth unsuccessfully increased); “D,” represented by light red, indicates prefectures with a negative indirect effect characterized by positive *a* and negative *b* estimates (i.e., mobility increased, but infection growth decreased); and “E,” represented by white, indicates prefectures with a non-significant indirect effect. The numbers on the maps correspond to the numbers in the list of prefectures.

**Fig. 5:**
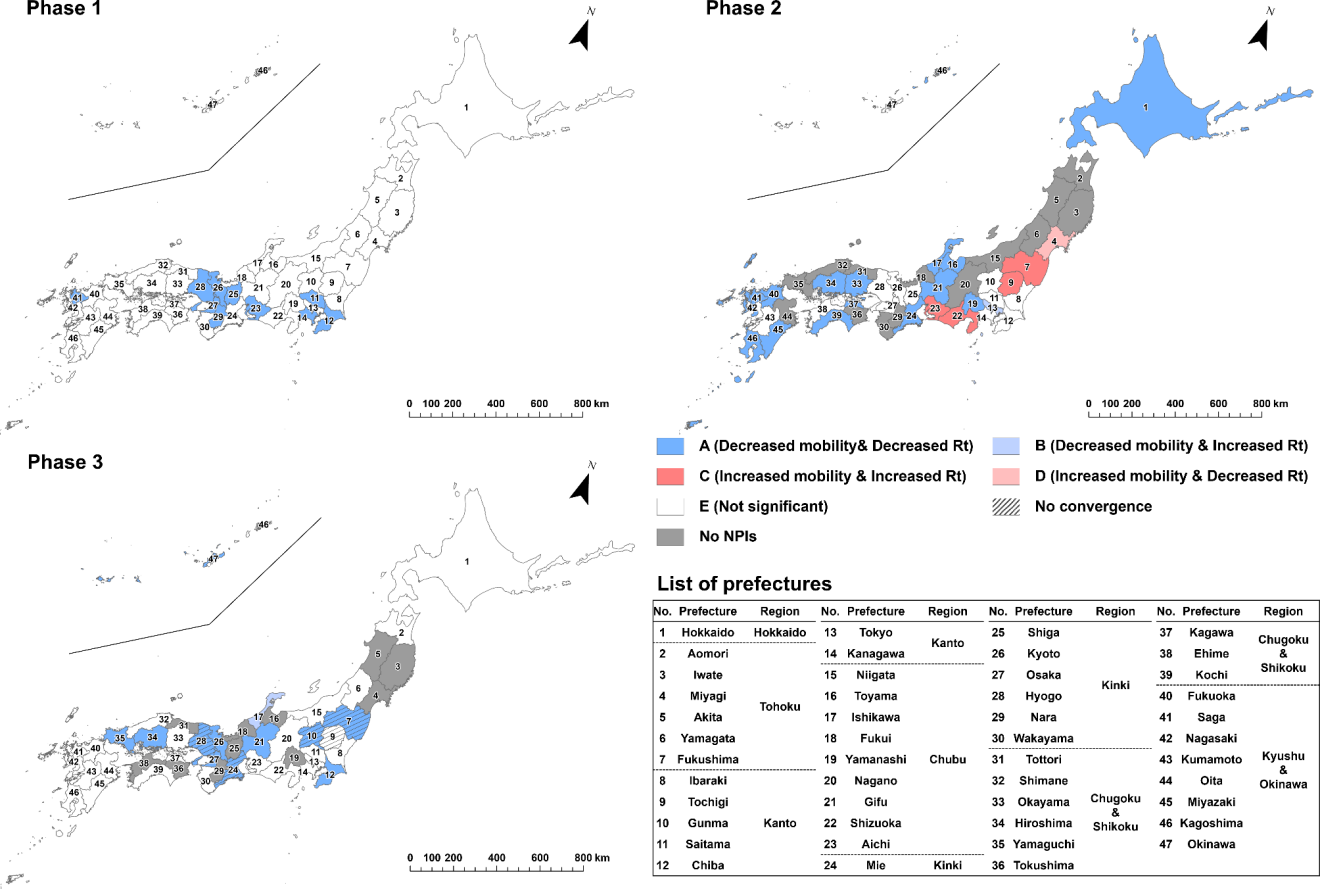
Indirect effects of implementing NPIs on COVID-19 infection growth, mediated by reduction in visitors from other prefectures. “A,” represented by dark blue, indicates prefectures with a negative indirect effect characterized by negative *a* and positive *b* estimates (i.e., successful reduction of epidemic growth via mobility reduction, as designed); “B,” represented by light blue, indicates prefectures with a positive indirect effect characterized by negative *a* and negative *b* estimates (i.e., mobility decreased, but infection growth unsuccessfully increased); “C,” represented by dark red, indicates prefectures with a positive indirect effect characterized by positive *a* and positive *b* estimates (i.e., both mobility and infection growth unsuccessfully increased); “D,” represented by light red, indicates prefectures with a negative indirect effect characterized by positive *a* and negative *b* estimates (i.e., mobility increased, but infection growth decreased); and “E,” represented by white, indicates prefectures with a non-significant indirect effect. The numbers on the maps correspond to the numbers in the list of prefectures.

**Fig. 6:**
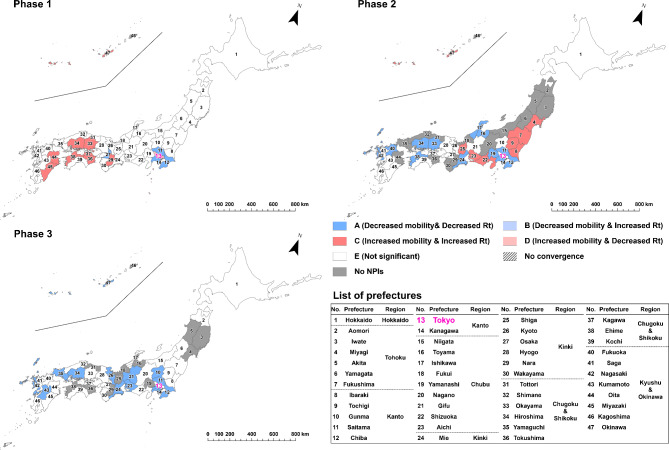
Indirect effects of implementing NPIs on COVID-19 infection growth, mediated by reduction in visitors from Tokyo. “A,” represented by dark blue, indicates prefectures with a negative indirect effect characterized by negative *a* and positive *b* estimates (i.e., successful reduction of epidemic growth via mobility reduction, as designed); “B,” represented by light blue, indicates prefectures with a positive indirect effect characterized by negative *a* and negative *b* estimates (i.e., mobility decreased, but infection growth unsuccessfully increased); “C,” represented by dark red, indicates prefectures with a positive indirect effect characterized by positive *a* and positive *b* estimates (i.e., both mobility and infection growth unsuccessfully increased); “D,” represented by light red, indicates prefectures with a negative indirect effect characterized by positive *a* and negative *b* estimates (i.e., mobility increased, but infection growth decreased); and “E,” represented by white, indicates prefectures with a non-significant indirect effect. The numbers on the maps correspond to the numbers in the list of prefectures. The Tokyo location corresponds to area #13 highlighted in purple.

**Fig. 7:**
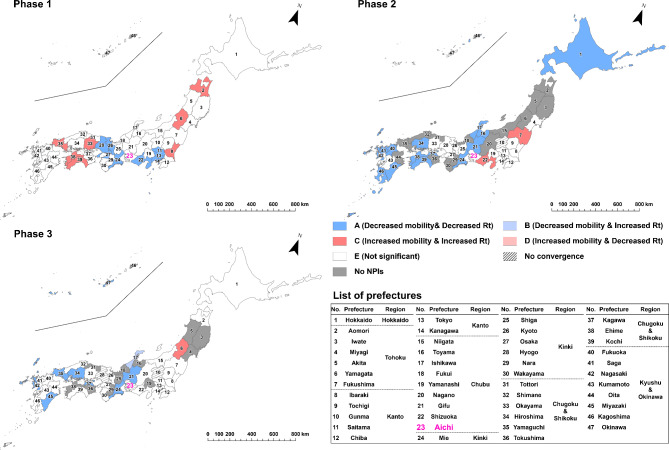
Indirect effects of implementing NPIs on COVID-19 infection growth, mediated by reduction in visitors from Aichi. “A,” represented by dark blue, indicates prefectures with a negative indirect effect characterized by negative *a* and positive *b* estimates (i.e., successful reduction of epidemic growth via mobility reduction, as designed); “B,” represented by light blue, indicates prefectures with a positive indirect effect characterized by negative *a* and negative *b* estimates (i.e., mobility decreased, but infection growth unsuccessfully increased); “C,” represented by dark red, indicates prefectures with a positive indirect effect characterized by positive *a* and positive *b* estimates (i.e., both mobility and infection growth unsuccessfully increased); “D,” represented by light red, indicates prefectures with a negative indirect effect characterized by positive *a* and negative *b* estimates (i.e., mobility increased, but infection growth decreased); and “E,” represented by white, indicates prefectures with a non-significant indirect effect. The numbers on the maps correspond to the numbers in the list of prefectures. The Aichi location corresponds to area #23 highlighted in purple.

**Fig. 8:**
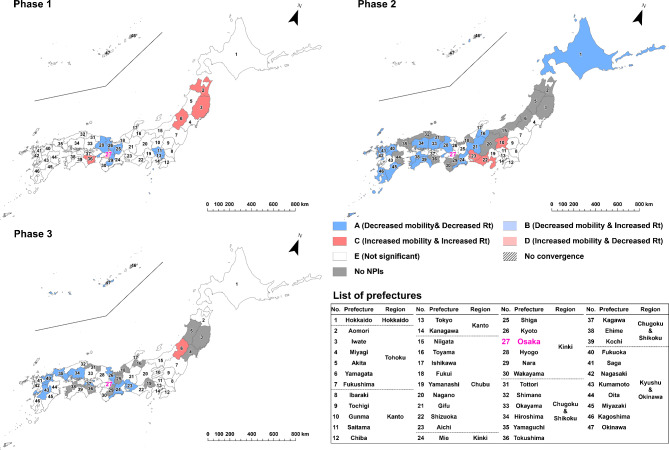
Indirect effects of implementing NPIs on COVID-19 infection growth, mediated by reduction in visitors from Osaka. “A,” represented by dark blue, indicates prefectures with a negative indirect effect characterized by negative *a* and positive *b* estimates (i.e., successful reduction of epidemic growth via mobility reduction, as designed); “B,” represented by light blue, indicates prefectures with a positive indirect effect characterized by negative *a* and negative *b* estimates (i.e., mobility decreased, but infection growth unsuccessfully increased); “C,” represented by dark red, indicates prefectures with a positive indirect effect characterized by positive *a* and positive *b* estimates (i.e., both mobility and infection growth unsuccessfully increased); “D,” represented by light red, indicates prefectures with a negative indirect effect characterized by positive *a* and negative *b* estimates (i.e., mobility increased, but infection growth decreased); and “E,” represented by white, indicates prefectures with a non-significant indirect effect. The numbers on the maps correspond to the numbers in the list of prefectures. The Osaka location corresponds to area #27 highlighted in purple.

Estimated results of the state-space models are mapped in Figs. 3, 4, 5, 6, 7 and 8 by type of human mobility, where each figure shows a map for each of the three epidemic phases. The NPIs were implemented in all 47 prefectures in the first phase, 33 prefectures in the second phase and 36 prefectures in the third phase (Fig. 1). Daily changes in the indicators of human mobility and infection growth, as well as the numerical details of prefectural estimates of $\:a$, $\:b$, $\:c$ (the direct effects), the indirect effects ($\:a\times\:b$), the total effects ($\:a\times\:b+c$), and the proportion of the indirect effects ($\:\text{I}\text{n}\text{d}\text{i}\text{r}\text{e}\text{c}\text{t}\:\text{e}\text{f}\text{f}\text{e}\text{c}\text{t}/\text{T}\text{o}\text{t}\text{a}\text{l}\:\text{e}\text{f}\text{f}\text{e}\text{c}\text{t}\times\:100$)^25^ are provided in the supplement (*Supplement 2*,* Figures S3-S9; Supplement 3*,* Tables S1-S18*). Focusing on Tokyo results in the first phase, implementation of NPIs changed the indicator of COVID-19 infection growth by -0.082 (95% CI = -0.189 to -0.015) via changes in downtown daytime human mobility (Table S1). Since the infection growth indicator was expressed as the logarithm of $\:{\widehat{R}}_{t}$ smoothed by 7-day moving average, the estimate of -0.082 indicated that the smoothed $\:{\widehat{R}}_{t}$ for day $\:t$ decreased by a factor of 0.92 (= exp(-0.082)) due to the daytime human mobility control in downtown when day $\:t-7$ was included in the NPIs period. The percentage of the daytime population control effect on mitigating the infection growth was 18.469% (3.458–43.117%) of the total effect of NPIs implementation. In the case of downtown nighttime human mobility control (Table S4), the mean of the indirect effect was − 0.028 (95% CI = -0.073 to -0.006) and the percentage of the indirect effect was 6.217% (1.130–16.690%). In case of the visitor population control (Table S7), no significant indirect effect was found (mean = -0.031; 95% CI = -0.078 to 0.003).

Figure 3 shows that, in the first phase, implementation of NPIs were estimated to be effective in mitigating growth of infection by reducing daytime human mobility in downtown areas for 26 prefectures, out of 32 prefectures where the posterior distribution converged. In the second and third phases, there were few Type A prefectures, whereas *Rt* actually increased even though implementation of NPIs reduced human mobility (Type B).

For nighttime human mobility in downtown areas (Fig. 4), all prefectures except Kagawa were classified as Type A in the first phase, meaning statistically significant reduction of infection growth from NPI implementation through human mobility reduction. In the second phase, 25 of the 33 prefectures where the NPIs were implemented continued to be classified as Type A, while some prefectures changed to Type B or Type E in central parts of metropolitan areas such as Tokyo, Hokkaido, Aichi, Kyoto, and Osaka. In the third phase, the number of Type A prefectures further decreased; in Hokkaido, Kanto, and Kinki regions, all prefectures were classified as Type B or E. Of the 18 non-Type A prefectures (except for Gunma since the posterior distribution of the indirect effect did not converge), 11 prefectures were Type B, meaning the *Rt* increased although the human mobility decreased during the NPIs. Focusing only on the parameter $\:a$, of the prefectures where the NPIs were implemented, the posterior distributions converged with a significant negative value in all 47 prefectures in the first phase, all 33 prefectures in the second phase, and 34 of the 36 prefectures in the third phase (Table S4 - S6). That is, although implementation of the NPIs generally reduced the nighttime human mobility at downtown areas throughout the first phase to the third phase, its effect of human mobility control on mitigating the infection growth decreased with each phase.

In terms of the total effects of the NPIs via reduction of nighttime human mobility in downtown areas (Table S4 - S6), Tokyo revealed a significant total effect (mean = -0.470; 95% CI = -0.561 to -0.378) with a significant indirect effect (mean = -0.028; 95% CI = -0.073 to -0.006) in the first phase. Although the total effect was not significant in the second phase (mean = 0.002; 95% CI = -0.049 to 0.051), it became significant again in the third phase (mean = -0.307; 95% CI = -0.421 to -0.194). However, no significant indirect effects were observed in Tokyo in either the second (mean = 0.019; 95% CI = 0.002 to 0.038) or third phases (mean = 0.086; 95% CI = -0.002 to 0.170). That is, mitigation of infection growth in Tokyo during the NPIs in the third phase may be from factors other than reducing nighttime human mobility in downtown areas. For Hiroshima, which was classified as Type A in all phases, the total and indirect effects were significant in all phases. The means of the total effects were − 0.652 (95% CI = -0.908 to -0.391) in the first phase, -0.213 (95% CI = -0.341 to -0.082) in the second phase, and − 0.728 (95% CI = -0.904 to -0.551) in the third phase. The means of the indirect effects were − 0.142 (95% CI = -0.217 to -0.084) in the first phase, -0.104 (95% CI = -0.165 to -0.054) in the second phase, and − 1.850 (95% CI = -4.489 to -0.358) in the third phase.

Regarding the effect of NPIs through mobility of visitors from other prefectures (Fig. 5), Type A areas were distributed around metropolitan areas such as Saitama, Chiba, Kanagawa, Aichi, Kyoto, Osaka, and Hyogo in the first phase. In the second phase, most Type A prefectures were distributed in Chubu, Chugoku and Shikoku, and Kyushu and Okinawa regions while Miyagi, Fukushima, Tochigi, Shizuoka and Aichi were classified as Type C or D, indicating increased visitors’ mobility during the NPIs. In the third phase, of the 31 prefectures that could be evaluated, the Type A prefectures were only Chiba, Gifu, Kyoto, Osaka, Hiroshima, Yamaguchi and Okinawa.

In the case of mobility of visitors from Tokyo (Fig. 6) within the first phase, prefectures around Tokyo, such as Saitama, Chiba and Kanagawa were classified as Type A, while some prefectures located in Kinki, Chugoku and Shikoku, and Kyushu Okinawa regions were classified as Type C. In the second and third phases, Type A prefectures continued to be observed adjacent to Tokyo, but also in several regions away from Tokyo. Several prefectures, including Miyagi, Fukushima, Ibaraki, Tochigi, Shizuoka, Aichi and Shiga, were classified as Type C in the second phase, indicating that the increase in human mobility from Tokyo contributed to the increase in *Rt* during the NPIs. Regarding visitors from Aichi or Osaka, infection growth was reduced in their neighboring prefectures and the western prefectures throughout all three NPI phases and in Hokkaido during the second phase (Figs. 7 and 8).

Note that in the mediation analysis, the parameters did not converge for several prefectures in each condition. This can be attributed to excessive changes in the COVID-19 infection growth indicators in prefectures with small infection sizes or to large variations in the human mobility indicators.

## Discussion

In Japan, several studies have shown that human mobility reduction was positively associated with mitigating infection growth in Tokyo and other metropolitan areas during the first SoE^7,8^. However, there is lacking evaluation of whether repeated NPIs during subsequent epidemic phases were effective for controlling infection through expected changes in human mobility. Furthermore, the effects of the NPIs across Japan, including areas outside of the major metropolitan regions, remained unclear. By considering a framework of mediation analysis, this study evaluated the hypothesis that implementing prefecture-level NPIs mitigated infection growth during the epidemic period from 2020 to 2022 through changes in human mobility.

Our mediation model confirmed that, within the first epidemic phase, NPIs mitigated growth of infection in more than half of the prefectures at least in part due to human mobility reduction in downtowns during both daytime and nighttime. In particular, the effect of NPIs on mitigating infection growth through human mobility reduction at nighttime was clearly confirmed in most of the prefectures (46 of 47). A previous study showed that changes in human mobility in nightlife places were associated with the early stage of the COVID-19 epidemic in the major metropolitan areas in Japan^8^. Given that several epidemiological surveys have reported that nightlife settings had a higher transmission risk than other settings such as households and health care facilities^30,31^, focused nighttime human mobility control in downtown areas should be especially critical for reducing the spread of COVID-19 in the early phase. In addition, this study revealed that nighttime mobility reduction in downtown areas from implementing NPIs was effective in almost all prefectures, including non-metropolitan areas where downtowns are generally small.

However, in the second phase, the expected effect of NPIs acting through nighttime human mobility reduction in downtowns was no longer observed in several prefectures located in the major metropolitan areas, such as Chiba, Tokyo, Kanagawa, Aichi, Kyoto and Osaka. Moreover, in the third phase, the effect was not observed in all prefectures located in the Kanto and Kinki regions, which roughly correspond to the Tokyo and Osaka metropolitan areas. Focusing on the second phase, there was no significant indirect effect of human mobility control on mitigating the spread of infection in prefectures including Hokkaido, Chiba, Kanagawa, Aichi, and Kyoto, despite reduced human mobility during the NPIs. It is possible that widespread vaccination during the second phase, due to its infection-preventive effects^32,33^, altered the relationship between human mobility and the spread of infection. Furthermore, despite the significant reduction in human mobility during NPIs in the latter two phases, *Rt*increased in Tokyo in the second phase and several other metropolitan prefectures such as Saitama, Chiba, Kanagawa and Osaka in the third phase. The pattern of transmission in Japan has been reported as initially spreading in nightlife-related settings, followed by widespread infection in household and health care-related settings in later stages of each epidemic wave^31^. Thus, in those prefectures, there is a possibility that the major transmission routes had already shifted from nightlife-related to household and health care-related settings with the expansion of the epidemic before the NPIs were issued. As a result, reducing nighttime human mobility in downtown areas did not clearly contribute to reducing infection spread in later phases. In addition, the speed of infection growth increased during the second and third phases due to emergence of new variants such as Delta and Omicron^34,35^, thus making it more difficult to control transmission through human mobility reduction at specific places considered high-risk, such as nightlife spots, before the infection became widespread.

It is noteworthy, however, that the effect was sustained in areas with relatively small epidemic size. In the Tohoku, Chugoku and Shikoku regions, which are remote from Tokyo and Osaka, all prefectures where NPIs were implemented showed effectiveness in mitigating spread of infection through nighttime human mobility reduction in downtown areas in both the second and third phases. Even during the epidemic phases of the Delta and Omicron variants, the NPIs appear to effectively reduce human mobility at nightlife places as a countermeasure against transmission before the widespread diffusion of the infection in areas where the local epidemic sizes are still small. By controlling human mobility before the infection spread widely, it was possible to shorten the duration of the NPIs in the first phase and in some prefectures outside of the Tokyo and Osaka metropolitan areas, such as Miyagi, Fukushima, Toyama, Ishikawa, Okayama, and Hiroshima, during the second phase (Fig. 1).

Implementing NPIs in the first phase also appeared to mitigate spread of infection through reducing daytime human mobility in downtowns within more than half of the prefectures. However, in the second and third phases, these effects were not observed in most of the prefectures, including those where human mobility was significantly reduced. This observation suggests that the daytime human mobility reduction associated with NPIs was less effective in mitigating the spread of infection than the nighttime human mobility reduction that was particularly emphasized in Japan.

There was a clear decline in inter-regional travel within large metropolitan areas that also had a large scale of infection in the early stages of the epidemic^13^, suggesting that those travel controls reduced opportunities of transmission in urban areas. Focusing on the movement of people from central prefectures of major metropolitan areas, including Tokyo, Aichi and Osaka, the reduction of visitors from each central prefecture during the NPIs was estimated to be effective in mitigating the spread of infection in neighboring prefectures throughout the first to third phases. This suggests that reducing travel to neighboring prefectures for daily errands or work during the NPIs was effective in reducing the spatial spread of COVID-19. However, there were also some cases where increased human mobility from the central prefectures during NPIs is associated with the spread of infection. For example, increased travel from Tokyo contributed to spread of the infection in Nara, Okayama, Hiroshima, Tokushima, Kagawa, Ehime, Oita, Miyazaki and Okinawa during the NPIs in the first phase; and in Miyagi, Fukushima, Ibaraki, Tochigi, Shizuoka, Aichi, Shiga and Okinawa in the second phase. During the NPIs, it is possible that people moved away from major cities with high infection risks to rural areas with a smaller scale of infection. In particular, the period of the NPIs in the second phase included the consecutive vacation season known as Golden Week from late April to early May and the summer vacation season in August. It indicates that increased travel to rural areas including traveling back to rural hometowns triggered or amplified the local spread of infection in rural areas.

In each phase, the effects of NPIs via controlling each human mobility setting tended to be geographically synchronized in some neighboring prefectures, especially in the Kanto or Kinki regions. These regions include major metropolitan areas of Japan, where people’s living and economic activities are spread across multiple prefectures. Therefore, it is suggested that in those prefectures, the trend of infection growth and the effect of human mobility control were also linked.

This study showed that the effect of NPIs on infection growth mitigation through controlling human mobility varied with conditions such as epidemic phase and region. First, controlling nighttime human mobility in downtown areas during the NPIs was clearly effective in mitigating infection growth throughout Japan in the early stages of the epidemic. Second, during subsequent epidemics associated with the Delta and Omicron variants, it became difficult to mitigate infection growth in urban areas where the infection spread widely, even though the nighttime human mobility in downtown areas was reduced by the NPI implementation, indicating a change in major transmission routes from nightlife-related to household and health care-related. However, the interventions were still effective in non-metropolitan prefectures with relatively small epidemic sizes. Third, the governments’ requests that people refrain from inter-prefectural travel during the NPIs was effective in reducing the spatial spread of the infection from urban areas to adjacent areas over the epidemic period from 2020 to 2022.

This study has several limitations. First, we analyzed the effect of NPIs without distinguishing between SoE and PPM. Although they were both intended to control human mobility, their implementation criteria and coverage differed. In addition, each prefecture’s localized interventions to control human mobility were outside the scope of this study. However, note that our results should be insightful for getting an overview of the effects of NPIs targeting human mobility control in Japan. Second, aside from the human mobility control, other factors such as vaccination campaigns, increased awareness of personal infection prevention, widespread mask usage, and enhanced social distancing practices also contributed to mitigating the spread of infection. While the combined effect of these factors during the NPIs periods was adjusted by the direct effect of the mediation analysis, this study did not consider their impact outside the NPIs periods. Third, although the national and prefectural governments implemented policies to promote inter-regional travel during the COVID-19 pandemic, including “Go To Travel” campaign from July to December 2020, when the infection trends were relatively calm, this study did not evaluate the impact of these policies on the spread of infection. It is difficult to organize the implementation history of such policies throughout Japan since various policies were implemented by each municipality to restore the local economy during the pandemic. Further research is needed to examine more detailed pathways of differently implemented policies on epidemic trends at various scales and geographic settings. Fourth, there are several limitations regarding the human mobility data used in this study. The human mobility change indicators are based on the location data of NTT DOCOMO cell phone subscribers, meaning that the mobility of international travelers, who are at a higher risk of spreading infection, was not considered. In addition, in terms of daytime and nighttime human mobility changes, we focused only on mobility in downtown areas, excluding other potentially relevant locations such as shopping streets, workplaces, public transportation, and residential places, which might also have a significant relationship with the spread of infection. However, during most of the study period, the Japanese government implemented entry restrictions, and the NPIs were particularly aimed at controlling human mobility in downtown areas. Therefore, our approach is likely well-suited for evaluating the human mobility control measures implemented by the Japanese and local governments to mitigate the spread of infection.

## Conclusion

This study evaluated the effect of NPIs on mitigating COVID-19 infection spread through controlling human mobility in each prefecture of Japan throughout 2020–2022. Findings of this study provide evidence that reducing nighttime human mobility in downtown areas through implementing NPIs contributed to mitigating the local spread of infection in the early stages of the epidemic, although the effects diminished as the infection spread widely. However, even during outbreaks of new strains, nighttime human mobility control in downtown areas was effective in mitigating transmission in non-metropolitan regions where the infection spread had been limited compared to metropolitan regions. Furthermore, our results suggest that reducing human movement from large cities to adjacent areas during the NPIs was effective in controlling transmission throughout the epidemic period from 2020 to 2022. These findings help to evaluate the effectiveness of NPIs in controlling the spread of COVID-19 and should be considered for future emerging infectious disease control policies.

## Electronic supplementary material

Below is the link to the electronic supplementary material.

Supplementary Material 1

## Data Availability

Mobile Spatial Statistics is a product of DOCOMO InsightMarketing, Inc., Tokyo, Japan and cannot be shared openly. The other data and programs will be made available upon approved request from the corresponding author.

## Abbreviations

COVID-19: Coronavirus disease 2019
NPIs: Non-pharmaceutical interventions
SoEs: State of Emergency declarations
PPMs: Priority Preventative Measures
