# Supplementary material for: Local effects of non-pharmaceutical interventions on mitigation of COVID-19 spread through decreased human mobilities in Japan: a prefecture-level mediation analysis
Source: Sci Rep. 2024 Nov 6;14:26996. doi: 10.1038/s41598-024-78583-0 (PMC11541980; doi:10.1038/s41598-024-78583-0)
Supplement: Supplementary file 1 — Supplementary Material 1 [file 41598_2024_78583_MOESM1_ESM.docx]

**Supplementary Material**

**Local effects of non-pharmaceutical interventions on mitigation of COVID-19 spread through decreased human mobilities in Japan: A prefecture-level mediation analysis**

**Shohei Nagata, Yuta Takahashi, Hiroki M. Adachi, Glen D. Johnson, and Tomoki Nakaya***

***** tomoki.nakaya.c8@tohoku.ac.jp

**Supplement 1: The method for identification of downtown areas**

This section describes how to identify downtown areas based on the characteristics of the daily population.

**Material**

We employed “Mobile Spatial Statistics” (DOCOMO InsightMarketing, Inc., Tokyo, Japan) to classify high-resolution areas throughout Japan based on the population on a daily basis. The data provided the estimated hourly population in approximately 500m x 500m grid cells based on the cell phone networks of NTT DOCOMO.^1^ We used data from 60 randomly chosen weekdays in 2019 for the area classification process as described below. The sum of the 60-day population was calculated for each of the 336 categories based on two gender groups (male, female), seven age groups (15-19, 20-29, 30-39, 40-49, 50-59, 60-69, 70-79), and 24 hourly time groups (i.e. 0:00-0:59 AM, 1:00-1:59 AM, etc.) combinations. Specifically, we excluded grids with the 60-day population total of 0 for categories with more than 25% (i.e., more than 84 categories) to reduce the computational load.

**Classification Method**

First, we compressed the data characterizing hourly population change from 336 variables to eight variables by using Non-negative Matrix Factorization (NMF).^2^ The number of factors to be extracted from NMF was determined exploratively by referring to the cophenetic correlation coefficient. We used Non-Negative Double Singular Value Decomposition to determine the initial value of the NMF. Next, based on the scores of eight factors of NMF, each grid cell was classified into six groups by using Clustering Large Applications (CLARA), an extended method of k-medoid for a large-scale dataset.^3^ In determining the number of groups, we compared clustering results from two to ten groups and adopted the results from six groups due to their high interpretability.

**Classification Result**

Based on characteristics of the hourly population changes on weekdays (Figure S1) and the spatial distribution (Figure S2), we interpreted the six groups as follows: Low-density Residential Districts, Depopulated or Mountainous Districts, No-resident Districts with Daytime Population Growth, High-density Residential Districts, Business and Residential Districts, and Business and Downtown Districts. During the daytime, the population decreases in both Low-density and High-density Residential Districts, while it increases in the other groups, although the scale of population change varies (Figure S1). In addition, the Business and Downtown Districts group is distributed across the most highly urbanized areas, while the Business and Residential Districts group is distributed in the next most urbanized areas (Figure S2). The No-resident Districts with Daytime Population Growth group is primarily distributed in industrial zones.


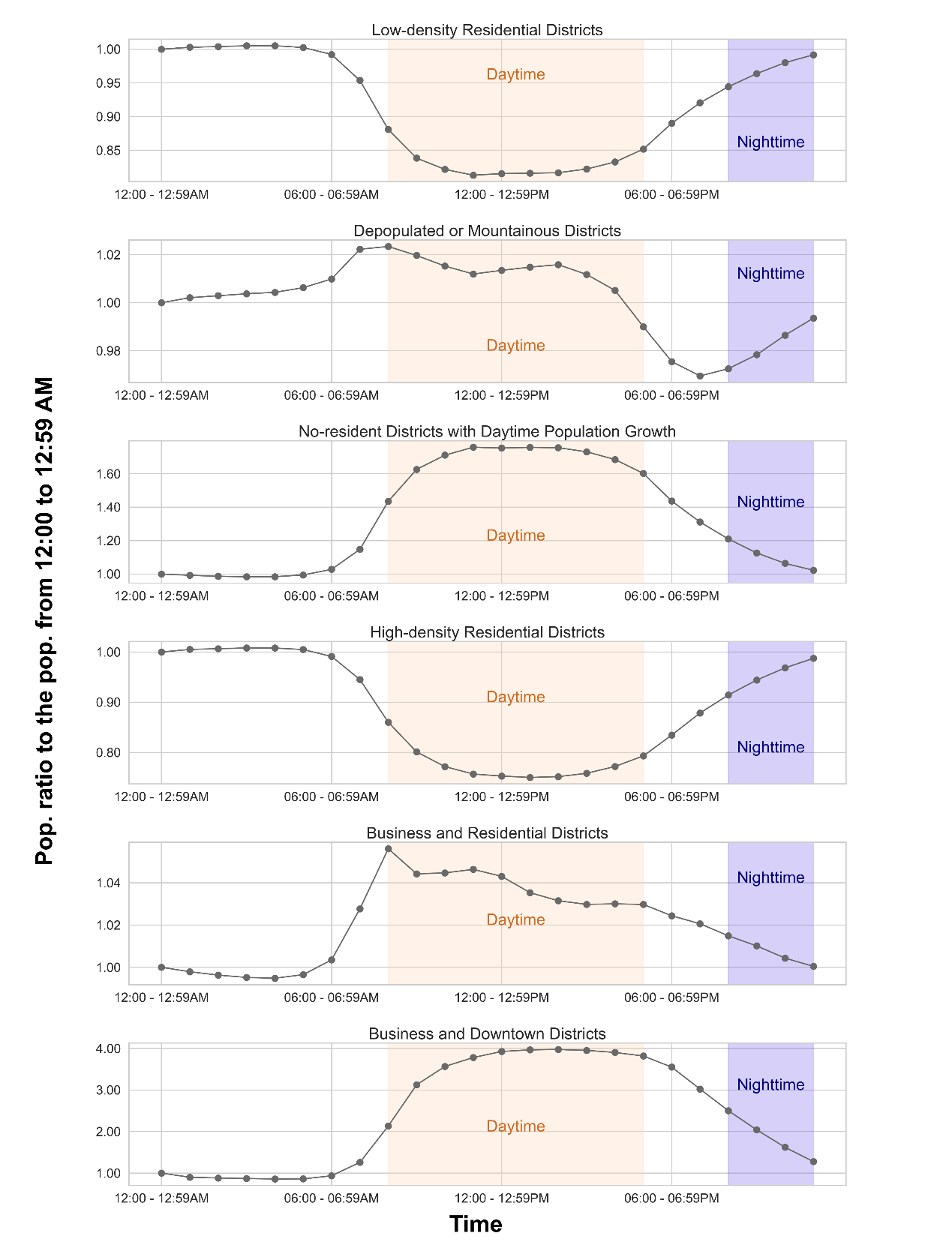


**Figure S1. Population ratio to the population from 12:00 to 12:59 AM on weekdays by cluster groups**

Note: The population ratio was calculated based on the median population at each time period across 60 randomly selected weekdays.


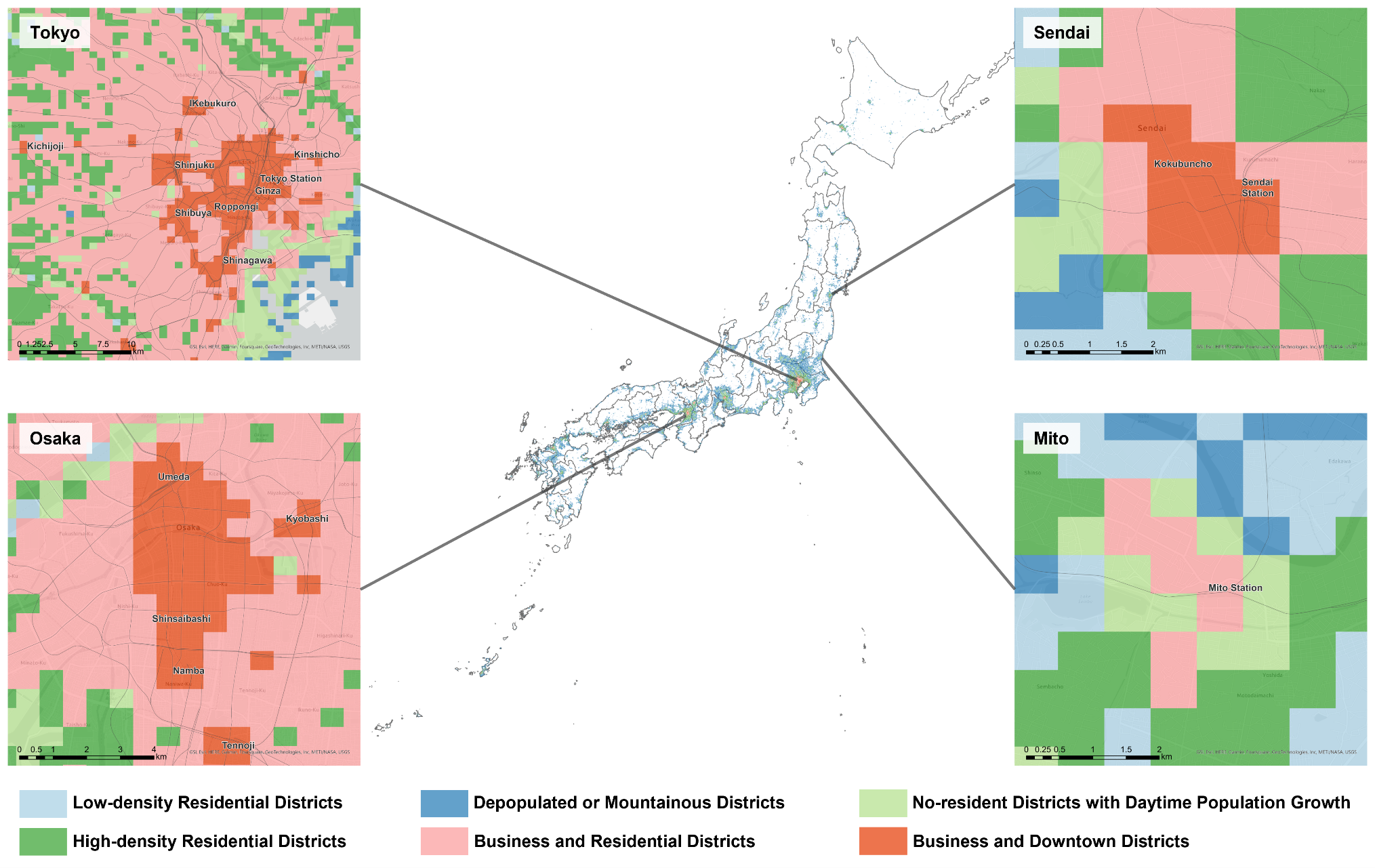


**Figure S2. Spatial distribution of each cluster group**

The Business and Downtown Districts we defined was consistent with the distribution of major downtown areas in megacities such as Tokyo and Osaka. Our defined downtown areas also covered the actual downtown areas in Sendai City, Miyagi Prefecture, the central city of the Tohoku region. However, in the case of central cities in rural prefectures, the Business and Downtown Districts did not exist because the human mobility trends were different from those in the large cities. As a typical example of a central city in a rural prefecture, in the case of Mito City, Ibaraki Prefecture, the grids around Mito Station, located in the center of the city, were classified as Business and Residential Districts. Such cases were frequently observed in other rural prefectures. Therefore, for the daytime and nighttime human mobility analysis in this study, we essentially defined the Business and Downtown Districts as downtown areas, but for prefectures where that group did not exist, we used the Business and Residential Districts as a proxy for downtown areas.

References

1. Terada M, Nagata T, Kobayashi M. Population Estimation Technology for Mobile Spatial Statistics. *NTT DOCOMO Technical Journal*. 2013;24(1). Accessed April 12, 2023. https://www.docomo.ne.jp/english/binary/pdf/corporate/technology/rd/technical_journal/bn/vol14_3/vol14_3_010en.pdf

2. Lee D, Seung HS. Algorithms for Non-negative Matrix Factorization. In: Leen T, Dietterich T, Tresp V, eds. *Advances in Neural Information Processing Systems*. Vol 13. MIT Press; 2000. https://proceedings.neurips.cc/paper_files/paper/2000/file/f9d1152547c0bde01830b7e8bd60024c-Paper.pdf

3. Kaufman L, Rousseeuw PJ. *Finding Groups in Data: An Introduction to Cluster Analysis*. John Wiley & Sons, Inc.; 1990. doi:10.1002/9780470316801

**Supplement 2: Changes in the human mobility and COVID-19 infection growth indicators**

This section shows the daily changes in the indicators used in the mediation analysis.

**
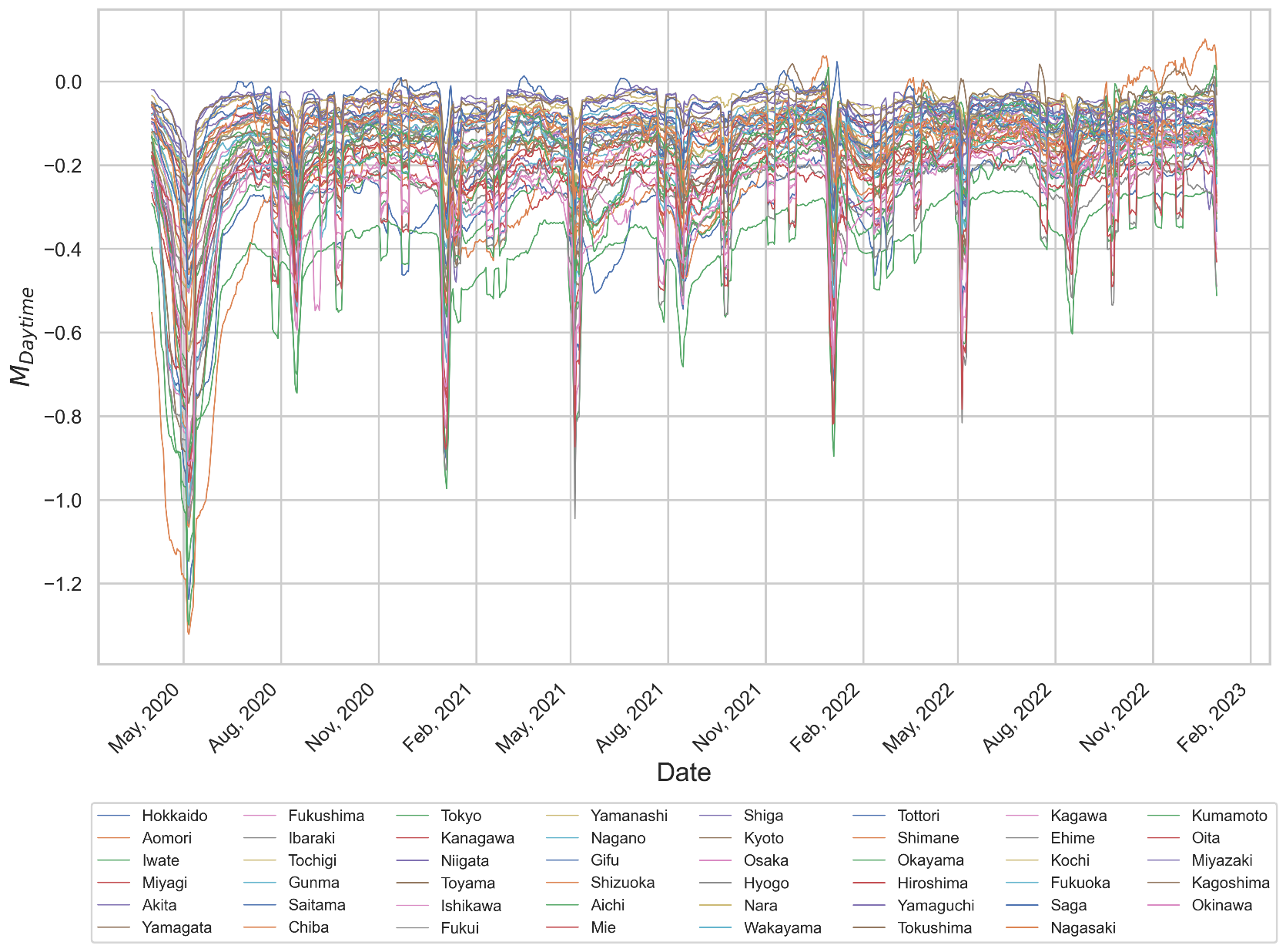
**

**Figure S3. Changes in indicators of daytime population in downtown areas in each prefecture**

**
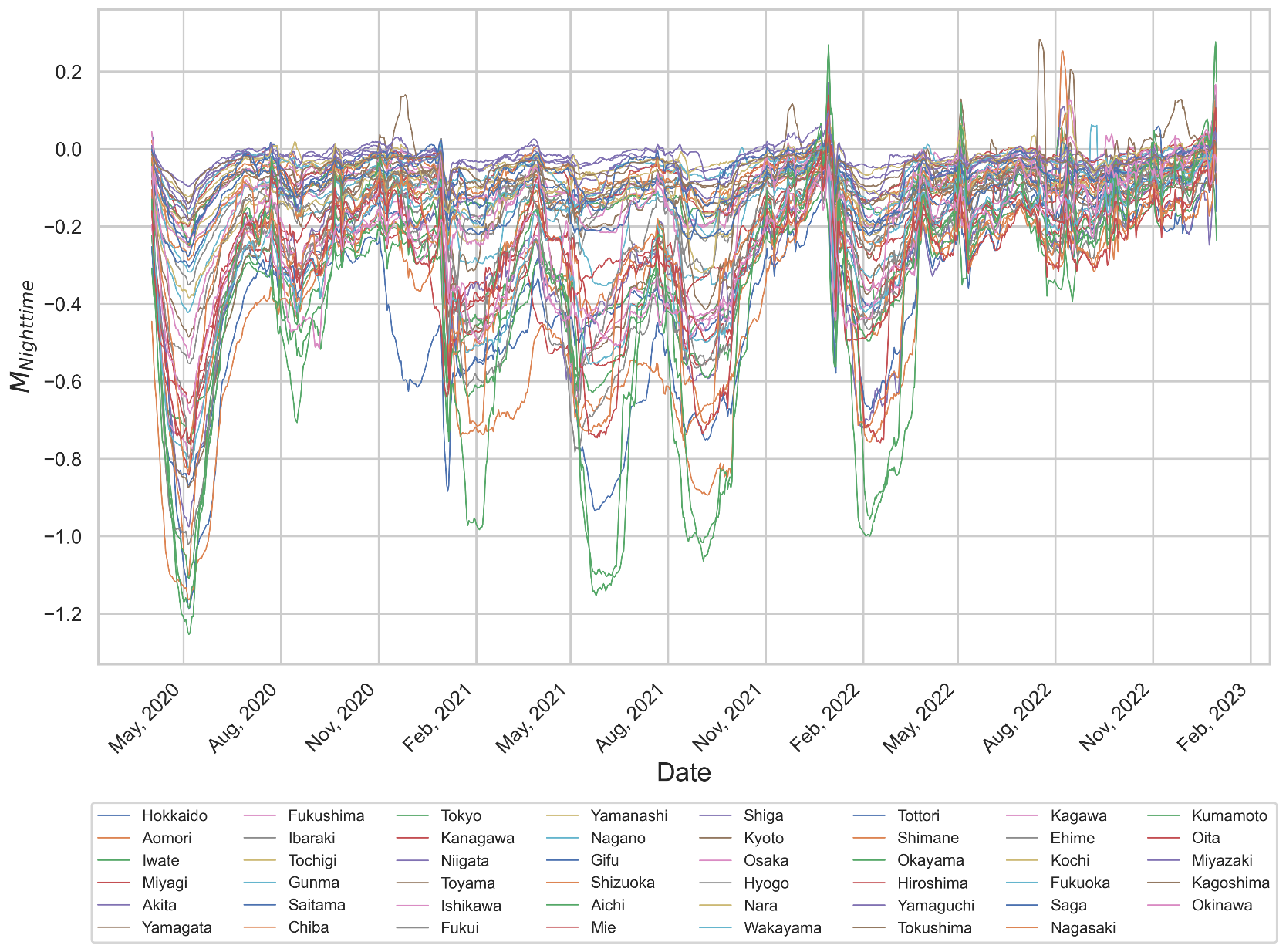
**

**Figure S4. Changes in indicators of nighttime population in downtown areas in each prefecture**

**
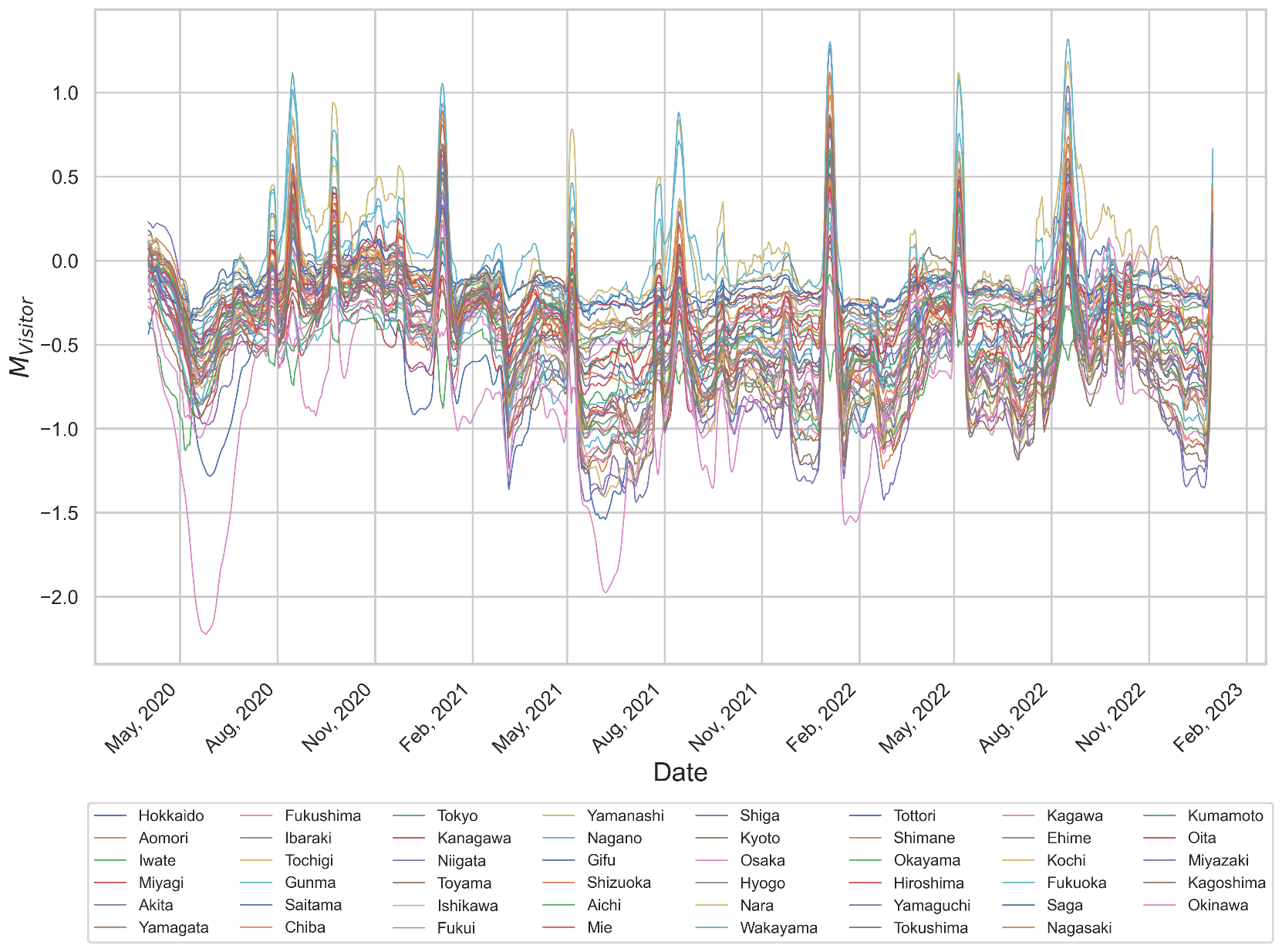
**

**Figure S5. Changes in indicators of visitor population from other prefectures in each prefecture**

**
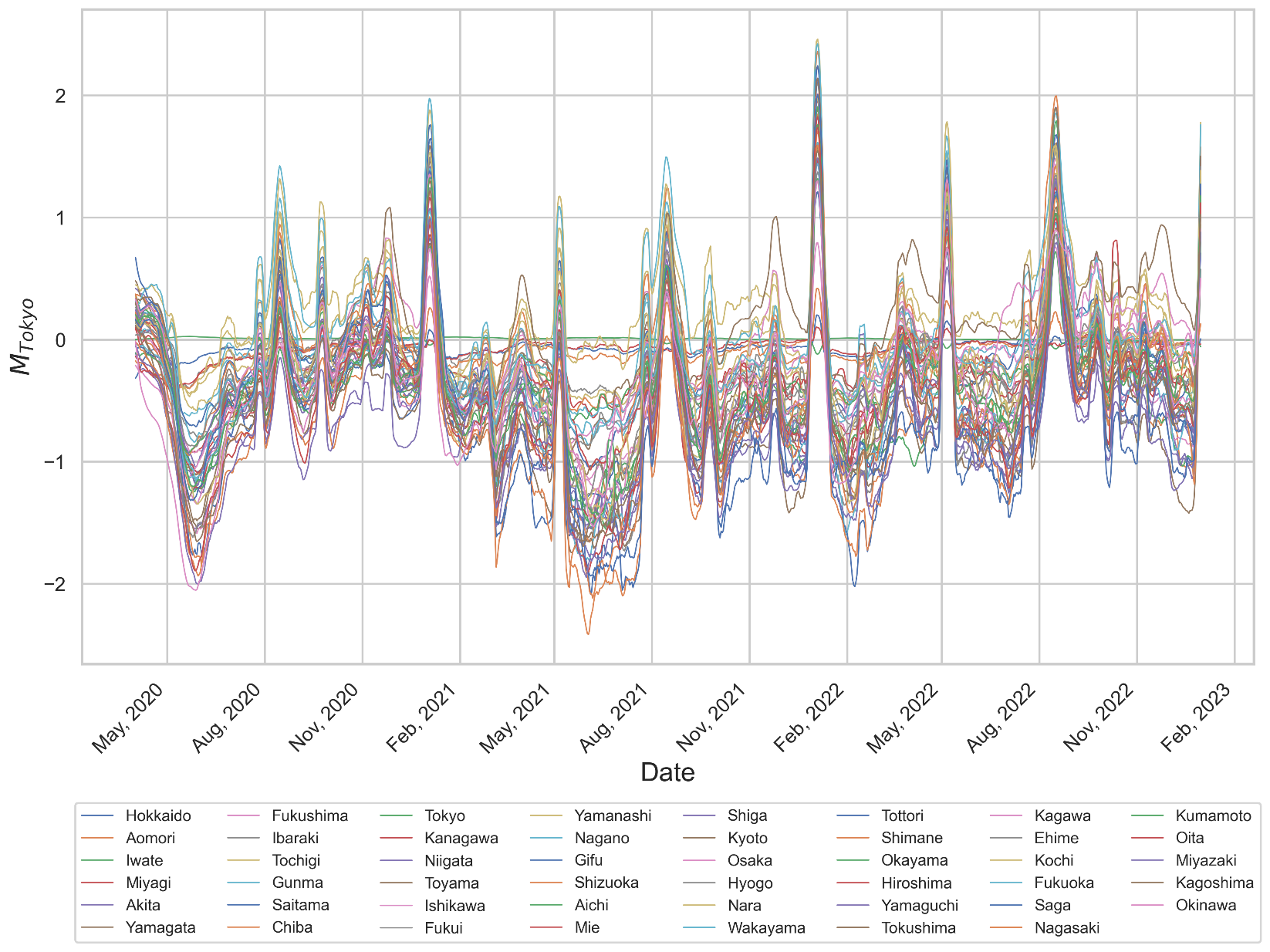
**

**Figure S6. Changes in indicators of visitor population from Tokyo in each prefecture**

**
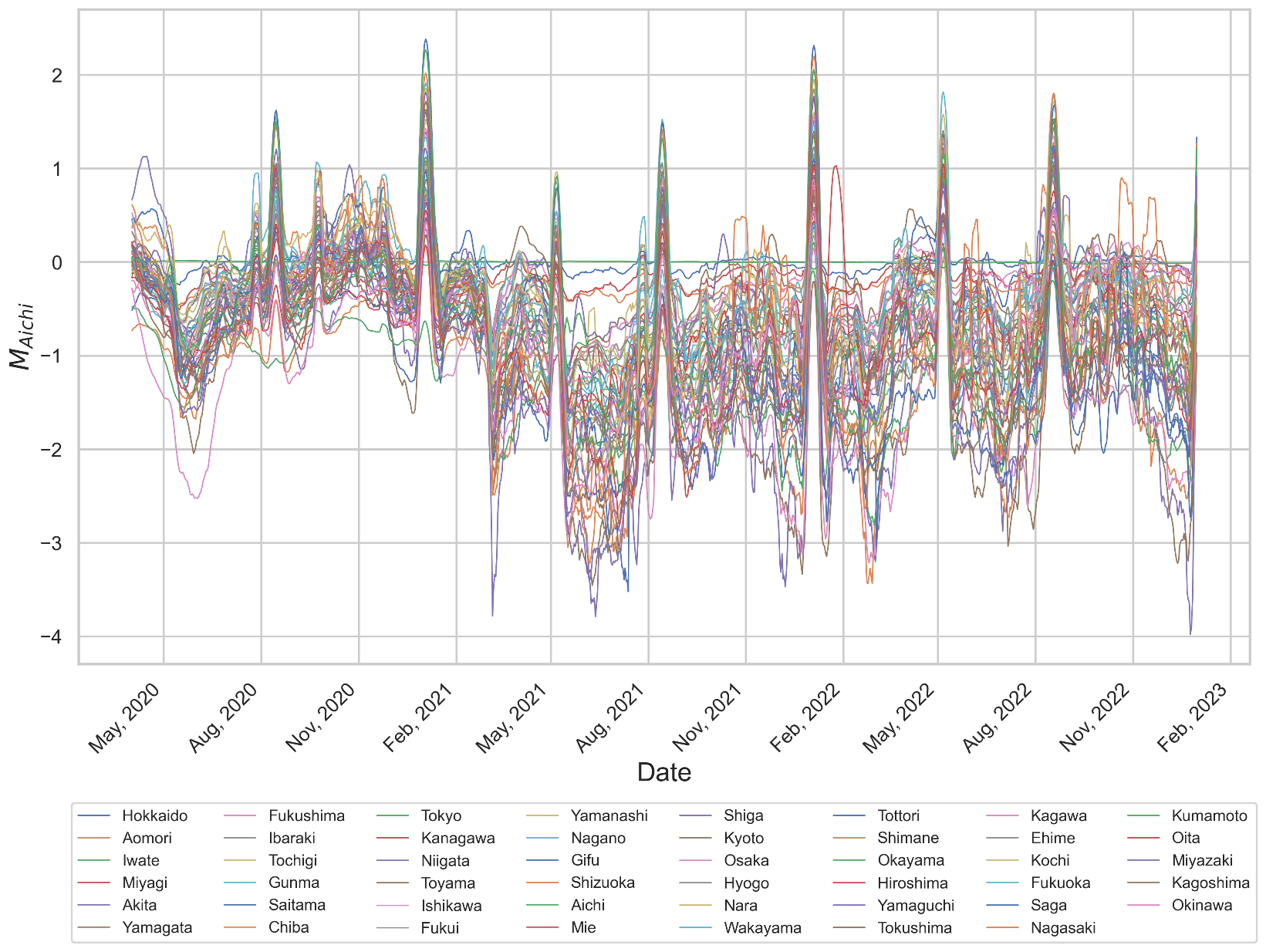
**

**Figure S7. Changes in indicators of visitor population from Aichi in each prefecture**

**
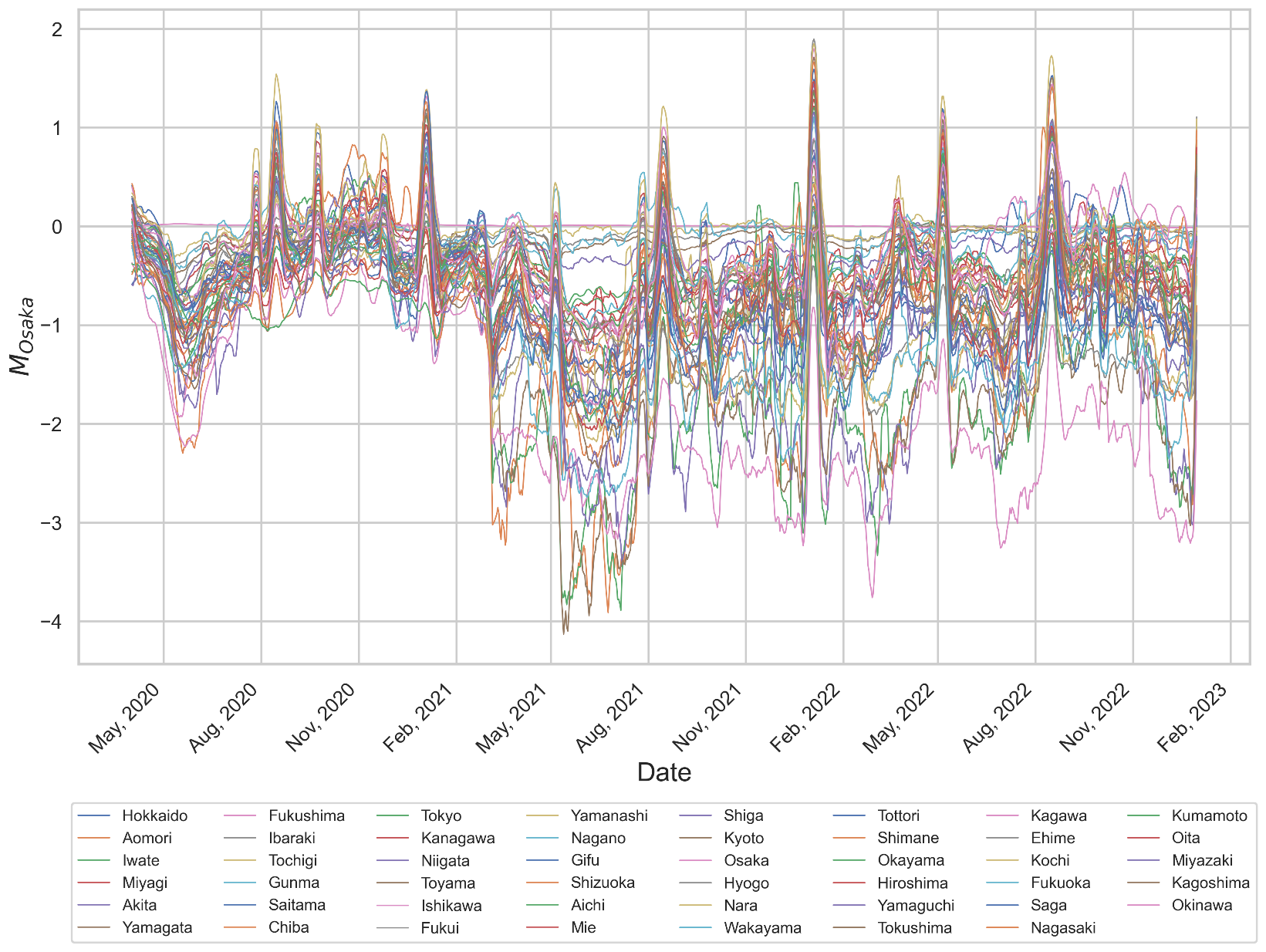
**

**Figure S8. Changes in indicators of visitor population from Osaka in each prefecture**

**
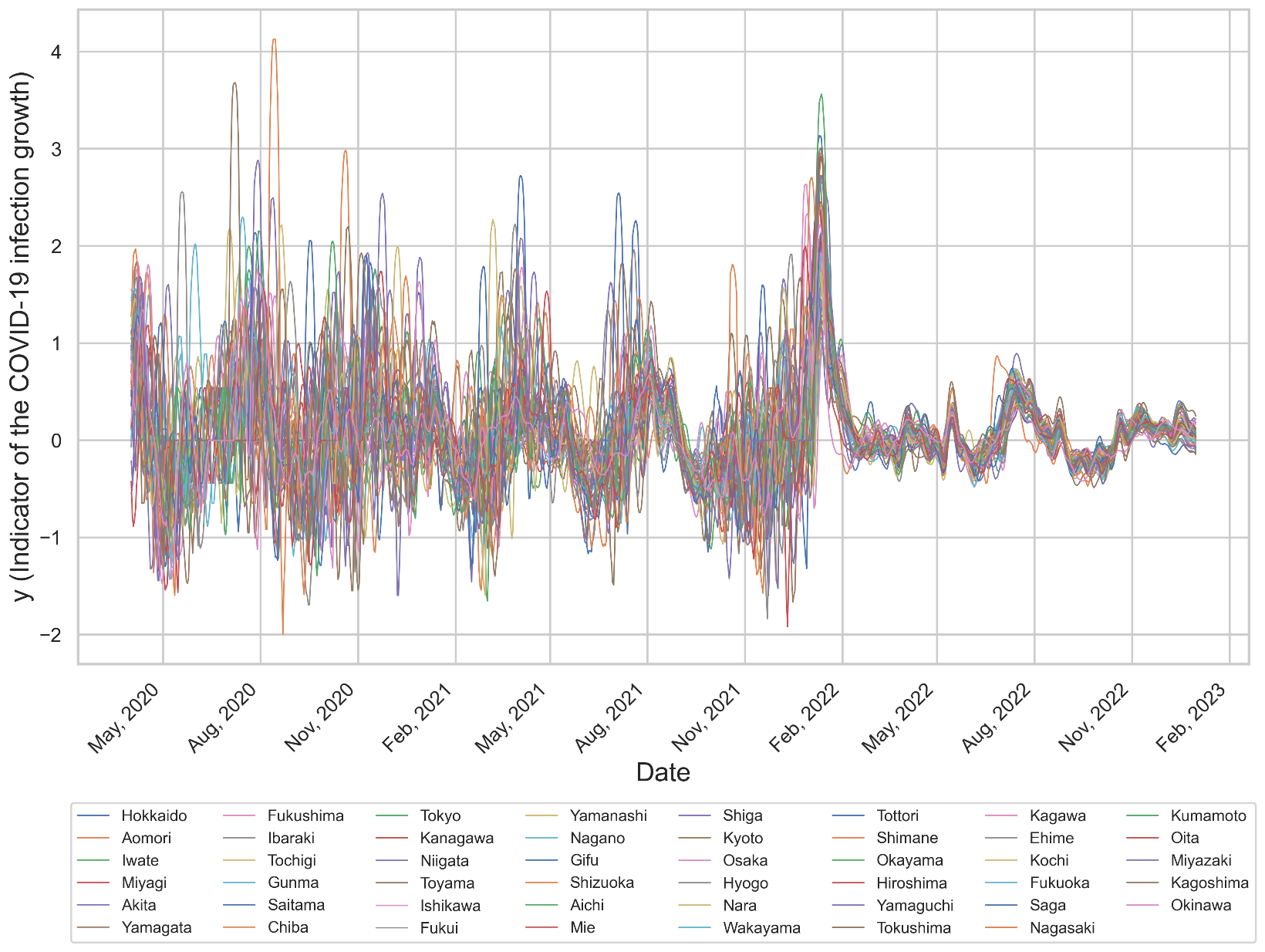
**

**Figure S9. Changes in indicators of the COVID-19 infection growth in each prefecture**

**Supplement 3: Estimation results for** $\boldsymbol{a}$**,** $\boldsymbol{b}$**, c (direct effects), indirect effects, and total effects**

This section shows the details of prefectural estimates of $a$, $b$, $c$ (the direct effects), the indirect effects ($a\times b$), the total effects ($a\times b+c$), and the proportion of the indirect effects (${Indirect effect}/{Total effect\times100}$). Note that the proportion of the indirect effect to the total effect may be greater than 100% and negative in cases of so-called inconsistent mediation, where the signs of the indirect and direct effects are different. For example, in cases where the indirect effect serves to increase the dependent variable, while the direct effect has the opposite effect, the proportion of the indirect effect may exceed 100%. In cases where the indirect effect tends to decrease the dependent variable, while the direct effect has the opposite effect, the proportion of the indirect effect will be negative.

**Table S1. Effects of NPIs on mitigating the COVID-19 infection mediated by daytime human mobility in downtown areas during Phase 1**

| **Prefecture Number** | **Prefecture Name** | **Parameters** | **Mean** | **95% Credible Interval** | | **Rhat** |
| --- | --- | --- | --- | --- | --- | --- |
|  |  |  |  | **Low** | **High** |  |
| 1 | Hokkaido | a | -0.443 | -0.500 | -0.380 | 1.003 |
|  |  | b | 2.998 | 1.996 | 4.615 | 1.031 |
|  |  | c (Direct effect) | 1.122 | 0.633 | 1.857 | 1.028 |
|  |  | Indirect effect | -1.334 | -2.126 | -0.819 | 1.029 |
|  |  | Total effect | -0.212 | -0.360 | -0.062 | 1.004 |
|  |  | Indirect effect (%) | 728.760 | 340.210 | 1783.764 | 1.000 |
| 2 | Aomori | a | -0.123 | -0.141 | -0.104 | 1.002 |
|  |  | b | -10.507 | -185.458 | 70.976 | 1.454 |
|  |  | c (Direct effect) | -1.319 | -22.648 | 8.735 | 1.448 |
|  |  | Indirect effect | 1.294 | -8.755 | 22.634 | 1.448 |
|  |  | Total effect | -0.025 | -0.292 | 0.245 | 1.000 |
|  |  | Indirect effect (%) | -5998.077 | -59212.388 | 57312.650 | 1.000 |
| 3 | Iwate | a | -0.121 | -0.147 | -0.095 | 1.008 |
|  |  | b | 0.200 | -12.967 | 11.572 | 1.076 |
|  |  | c (Direct effect) | -0.087 | -1.730 | 1.292 | 1.074 |
|  |  | Indirect effect | -0.019 | -1.427 | 1.614 | 1.076 |
|  |  | Total effect | -0.106 | -0.287 | 0.075 | 1.000 |
|  |  | Indirect effect (%) | 498.348 | -4906.343 | 5092.987 | 1.000 |
| 4 | Miyagi | a | -0.419 | -0.465 | -0.370 | 1.030 |
|  |  | b | -6.245 | -69.186 | 52.458 | 1.092 |
|  |  | c (Direct effect) | -3.325 | -30.448 | 21.589 | 1.091 |
|  |  | Indirect effect | 2.589 | -22.327 | 29.720 | 1.091 |
|  |  | Total effect | -0.736 | -0.928 | -0.544 | 1.001 |
|  |  | Indirect effect (%) | -351.324 | -4162.058 | 3091.212 | 1.088 |
| 5 | Akita | a | -0.114 | -0.135 | -0.093 | 1.088 |
|  |  | b | -8.995 | -69.242 | 154.669 | 1.364 |
|  |  | c (Direct effect) | -1.347 | -9.000 | 16.991 | 1.363 |
|  |  | Indirect effect | 0.985 | -17.337 | 8.582 | 1.363 |
|  |  | Total effect | -0.362 | -0.633 | -0.098 | 1.001 |
|  |  | Indirect effect (%) | -533.106 | -5494.892 | 5944.871 | 1.000 |
| 6 | Yamagata | a | -0.122 | -0.141 | -0.103 | 1.015 |
|  |  | b | 17.813 | 8.871 | 36.872 | 1.054 |
|  |  | c (Direct effect) | 1.417 | 0.267 | 3.736 | 1.042 |
|  |  | Indirect effect | -2.181 | -4.562 | -1.017 | 1.048 |
|  |  | Total effect | -0.764 | -1.001 | -0.558 | 1.039 |
|  |  | Indirect effect (%) | 286.498 | 133.613 | 584.633 | 1.031 |
| 7 | Fukushima | a | -0.153 | -0.168 | -0.134 | 1.280 |
|  |  | b | 51.764 | -197.781 | 130.019 | 3.079 |
|  |  | c (Direct effect) | 7.407 | -30.825 | 20.918 | 3.103 |
|  |  | Indirect effect | -8.122 | -21.551 | 30.100 | 3.103 |
|  |  | Total effect | -0.715 | -0.922 | -0.511 | 1.034 |
|  |  | Indirect effect (%) | 1166.245 | -4350.175 | 3716.164 | 2.838 |
| 8 | Ibaraki | a | -0.274 | -0.310 | -0.230 | 1.102 |
|  |  | b | 13.373 | 3.641 | 24.135 | 1.797 |
|  |  | c (Direct effect) | 3.068 | 0.218 | 6.549 | 1.793 |
|  |  | Indirect effect | -3.734 | -7.141 | -0.899 | 1.786 |
|  |  | Total effect | -0.666 | -0.842 | -0.492 | 1.066 |
|  |  | Indirect effect (%) | 572.463 | 131.592 | 1316.016 | 1.806 |
| 9 | Tochigi | a | -0.048 | -0.067 | -0.029 | 1.000 |
|  |  | b | 0.592 | -0.738 | 1.935 | 1.009 |
|  |  | c (Direct effect) | -0.317 | -0.525 | -0.107 | 1.003 |
|  |  | Indirect effect | -0.029 | -0.102 | 0.036 | 1.009 |
|  |  | Total effect | -0.346 | -0.515 | -0.174 | 1.001 |
|  |  | Indirect effect (%) | 10.333 | -8.979 | 42.936 | 1.005 |
| 10 | Gunma | a | -0.237 | -0.267 | -0.192 | 1.066 |
|  |  | b | -0.504 | -35.881 | 29.481 | 1.087 |
|  |  | c (Direct effect) | -0.538 | -9.160 | 6.857 | 1.088 |
|  |  | Indirect effect | 0.119 | -7.301 | 8.711 | 1.089 |
|  |  | Total effect | -0.419 | -0.644 | -0.195 | 1.006 |
|  |  | Indirect effect (%) | -5.942 | -2210.429 | 1799.023 | 1.051 |
| 11 | Saitama | a | -0.089 | -0.144 | -0.036 | 1.003 |
|  |  | b | 1.110 | 0.719 | 1.530 | 1.003 |
|  |  | c (Direct effect) | -0.213 | -0.344 | -0.076 | 1.002 |
|  |  | Indirect effect | -0.102 | -0.198 | -0.032 | 1.004 |
|  |  | Total effect | -0.314 | -0.422 | -0.205 | 1.000 |
|  |  | Indirect effect (%) | 33.356 | 9.905 | 68.840 | 1.003 |
| 12 | Chiba | a | -0.137 | -0.180 | -0.096 | 1.001 |
|  |  | b | 0.978 | 0.685 | 1.287 | 1.002 |
|  |  | c (Direct effect) | -0.198 | -0.338 | -0.056 | 1.001 |
|  |  | Indirect effect | -0.134 | -0.204 | -0.080 | 1.002 |
|  |  | Total effect | -0.332 | -0.451 | -0.212 | 1.000 |
|  |  | Indirect effect (%) | 42.170 | 21.141 | 75.406 | 1.001 |
| 13 | Tokyo | a | -0.100 | -0.172 | -0.027 | 1.001 |
|  |  | b | 0.784 | 0.431 | 1.236 | 1.003 |
|  |  | c (Direct effect) | -0.365 | -0.475 | -0.234 | 1.001 |
|  |  | Indirect effect | -0.082 | -0.189 | -0.015 | 1.002 |
|  |  | Total effect | -0.447 | -0.531 | -0.362 | 1.000 |
|  |  | Indirect effect (%) | 18.469 | 3.458 | 43.117 | 1.002 |
| 14 | Kanagawa | a | -0.049 | -0.100 | 0.000 | 1.002 |
|  |  | b | 0.765 | 0.422 | 1.149 | 1.003 |
|  |  | c (Direct effect) | -0.181 | -0.295 | -0.063 | 1.001 |
|  |  | Indirect effect | -0.039 | -0.096 | 0.000 | 1.003 |
|  |  | Total effect | -0.220 | -0.326 | -0.113 | 1.000 |
|  |  | Indirect effect (%) | 19.011 | 0.114 | 54.145 | 1.002 |
| 15 | Niigata | a | -0.207 | -0.268 | -0.143 | 1.000 |
|  |  | b | 1.200 | 0.596 | 1.809 | 1.000 |
|  |  | c (Direct effect) | 0.163 | -0.159 | 0.485 | 1.000 |
|  |  | Indirect effect | -0.250 | -0.425 | -0.109 | 1.000 |
|  |  | Total effect | -0.087 | -0.326 | 0.155 | 1.000 |
|  |  | Indirect effect (%) | 398.442 | -2666.385 | 2766.018 | 1.000 |
| 16 | Toyama | a | -0.265 | -0.320 | -0.206 | 1.002 |
|  |  | b | 5.874 | 2.397 | 12.141 | 1.006 |
|  |  | c (Direct effect) | 1.123 | -0.024 | 3.193 | 1.006 |
|  |  | Indirect effect | -1.594 | -3.675 | -0.540 | 1.006 |
|  |  | Total effect | -0.472 | -0.757 | -0.180 | 1.000 |
|  |  | Indirect effect (%) | 366.502 | 94.965 | 1119.366 | 1.000 |
| 17 | Ishikawa | a | -0.116 | -0.148 | -0.084 | 1.002 |
|  |  | b | 3.215 | 1.905 | 4.614 | 1.005 |
|  |  | c (Direct effect) | 0.011 | -0.334 | 0.365 | 1.002 |
|  |  | Indirect effect | -0.377 | -0.614 | -0.191 | 1.006 |
|  |  | Total effect | -0.366 | -0.619 | -0.107 | 1.000 |
|  |  | Indirect effect (%) | 121.781 | 39.038 | 362.588 | 1.000 |
| 18 | Fukui | a | -0.267 | -0.321 | -0.203 | 1.038 |
|  |  | b | -0.184 | -4.783 | 3.806 | 1.089 |
|  |  | c (Direct effect) | -0.746 | -2.198 | 0.524 | 1.085 |
|  |  | Indirect effect | 0.050 | -1.106 | 1.427 | 1.091 |
|  |  | Total effect | -0.696 | -0.980 | -0.417 | 1.008 |
|  |  | Indirect effect (%) | -1.114 | -198.855 | 197.662 | 1.079 |
| 19 | Yamanashi | a | -0.199 | -0.242 | -0.157 | 1.003 |
|  |  | b | -2.130 | -3.862 | -0.642 | 1.019 |
|  |  | c (Direct effect) | -0.935 | -1.457 | -0.471 | 1.014 |
|  |  | Indirect effect | 0.428 | 0.125 | 0.840 | 1.020 |
|  |  | Total effect | -0.507 | -0.758 | -0.263 | 1.001 |
|  |  | Indirect effect (%) | -87.511 | -186.794 | -28.885 | 1.013 |
| 20 | Nagano | a | -0.184 | -0.207 | -0.160 | 1.002 |
|  |  | b | 11.743 | 6.199 | 22.697 | 1.016 |
|  |  | c (Direct effect) | 1.738 | 0.615 | 3.885 | 1.016 |
|  |  | Indirect effect | -2.179 | -4.360 | -1.079 | 1.016 |
|  |  | Total effect | -0.441 | -0.659 | -0.222 | 1.000 |
|  |  | Indirect effect (%) | 531.246 | 216.954 | 1175.159 | 1.006 |
| 21 | Gifu | a | -0.064 | -0.087 | -0.041 | 1.000 |
|  |  | b | 1.717 | 0.640 | 2.910 | 1.007 |
|  |  | c (Direct effect) | -0.418 | -0.559 | -0.269 | 1.002 |
|  |  | Indirect effect | -0.111 | -0.208 | -0.037 | 1.004 |
|  |  | Total effect | -0.528 | -0.649 | -0.403 | 1.000 |
|  |  | Indirect effect (%) | 21.222 | 6.894 | 40.619 | 1.004 |
| 22 | Shizuoka | a | -0.376 | -0.439 | -0.310 | 1.000 |
|  |  | b | 2.147 | 1.105 | 3.375 | 1.001 |
|  |  | c (Direct effect) | 0.298 | -0.250 | 0.921 | 1.001 |
|  |  | Indirect effect | -0.814 | -1.380 | -0.388 | 1.001 |
|  |  | Total effect | -0.516 | -0.769 | -0.256 | 1.000 |
|  |  | Indirect effect (%) | 173.240 | 63.019 | 381.182 | 1.000 |
| 23 | Aichi | a | -0.181 | -0.244 | -0.118 | 1.000 |
|  |  | b | 0.975 | 0.500 | 1.458 | 1.003 |
|  |  | c (Direct effect) | 0.019 | -0.193 | 0.246 | 1.000 |
|  |  | Indirect effect | -0.177 | -0.296 | -0.081 | 1.002 |
|  |  | Total effect | -0.158 | -0.363 | 0.058 | 1.000 |
|  |  | Indirect effect (%) | 103.134 | -639.949 | 925.746 | 1.000 |
| 24 | Mie | a | -0.126 | -0.144 | -0.110 | 1.032 |
|  |  | b | -23.245 | -207.831 | 130.839 | 1.803 |
|  |  | c (Direct effect) | -3.633 | -26.550 | 16.091 | 1.805 |
|  |  | Indirect effect | 3.037 | -16.716 | 25.904 | 1.801 |
|  |  | Total effect | -0.596 | -0.816 | -0.378 | 1.125 |
|  |  | Indirect effect (%) | -396.663 | -4772.507 | 3305.772 | 1.824 |
| 25 | Shiga | a | -0.072 | -0.083 | -0.063 | 1.089 |
|  |  | b | 64.818 | 17.030 | 154.382 | 2.347 |
|  |  | c (Direct effect) | 4.026 | 0.685 | 10.223 | 2.268 |
|  |  | Indirect effect | -4.587 | -10.744 | -1.258 | 2.262 |
|  |  | Total effect | -0.560 | -0.766 | -0.346 | 1.016 |
|  |  | Indirect effect (%) | 865.471 | 211.926 | 2582.448 | 1.971 |
| 26 | Kyoto | a | -0.132 | -0.167 | -0.095 | 1.009 |
|  |  | b | 0.739 | 0.442 | 1.034 | 1.003 |
|  |  | c (Direct effect) | -0.425 | -0.565 | -0.287 | 1.001 |
|  |  | Indirect effect | -0.098 | -0.150 | -0.053 | 1.001 |
|  |  | Total effect | -0.523 | -0.642 | -0.403 | 1.001 |
|  |  | Indirect effect (%) | 19.068 | 9.173 | 32.017 | 1.001 |
| 27 | Osaka | a | -0.128 | -0.181 | -0.072 | 1.001 |
|  |  | b | 1.238 | 0.527 | 2.072 | 1.003 |
|  |  | c (Direct effect) | -0.111 | -0.277 | 0.078 | 1.002 |
|  |  | Indirect effect | -0.161 | -0.325 | -0.051 | 1.003 |
|  |  | Total effect | -0.273 | -0.396 | -0.149 | 1.000 |
|  |  | Indirect effect (%) | 62.427 | 18.069 | 139.096 | 1.002 |
| 28 | Hyogo | a | -0.126 | -0.173 | -0.081 | 1.001 |
|  |  | b | 0.902 | 0.504 | 1.319 | 1.002 |
|  |  | c (Direct effect) | -0.106 | -0.257 | 0.049 | 1.000 |
|  |  | Indirect effect | -0.115 | -0.198 | -0.054 | 1.002 |
|  |  | Total effect | -0.221 | -0.355 | -0.085 | 1.000 |
|  |  | Indirect effect (%) | 58.823 | 20.473 | 147.280 | 1.000 |
| 29 | Nara | a | -0.080 | -0.096 | -0.065 | 1.001 |
|  |  | b | 11.202 | 6.665 | 16.724 | 1.003 |
|  |  | c (Direct effect) | 0.426 | -0.079 | 1.048 | 1.003 |
|  |  | Indirect effect | -0.911 | -1.509 | -0.473 | 1.003 |
|  |  | Total effect | -0.486 | -0.684 | -0.280 | 1.000 |
|  |  | Indirect effect (%) | 198.307 | 86.545 | 387.939 | 1.002 |
| 30 | Wakayama | a | -0.118 | -0.138 | -0.098 | 1.244 |
|  |  | b | 13.402 | -45.014 | 133.735 | 1.423 |
|  |  | c (Direct effect) | 1.466 | -5.403 | 15.541 | 1.405 |
|  |  | Indirect effect | -1.688 | -15.769 | 5.200 | 1.408 |
|  |  | Total effect | -0.222 | -0.484 | 0.032 | 1.049 |
|  |  | Indirect effect (%) | 3631.431 | -8916.993 | 14849.535 | 1.000 |
| 31 | Tottori | a | -0.099 | -0.118 | -0.080 | 1.021 |
|  |  | b | 3.080 | -59.385 | 73.876 | 1.711 |
|  |  | c (Direct effect) | 0.092 | -6.098 | 7.192 | 1.695 |
|  |  | Indirect effect | -0.305 | -7.397 | 5.889 | 1.699 |
|  |  | Total effect | -0.212 | -0.466 | 0.041 | 1.003 |
|  |  | Indirect effect (%) | -2017.630 | -6136.063 | 7247.337 | 1.000 |
| 32 | Shimane | a | -0.146 | -0.173 | -0.119 | 1.069 |
|  |  | b | -56.230 | -145.869 | 21.972 | 2.878 |
|  |  | c (Direct effect) | -8.766 | -22.629 | 2.757 | 2.706 |
|  |  | Indirect effect | 8.293 | -3.232 | 22.154 | 2.708 |
|  |  | Total effect | -0.473 | -0.793 | -0.153 | 1.017 |
|  |  | Indirect effect (%) | -2018.265 | -7141.063 | 737.564 | 1.000 |
| 33 | Okayama | a | -0.199 | -0.257 | -0.148 | 1.002 |
|  |  | b | 1.699 | 1.079 | 2.373 | 1.002 |
|  |  | c (Direct effect) | 0.623 | 0.279 | 0.981 | 1.001 |
|  |  | Indirect effect | -0.341 | -0.559 | -0.189 | 1.003 |
|  |  | Total effect | 0.283 | 0.038 | 0.534 | 1.000 |
|  |  | Indirect effect (%) | -189.014 | -516.867 | -52.824 | 1.000 |
| 34 | Hiroshima | a | -0.340 | -0.403 | -0.274 | 1.001 |
|  |  | b | 3.372 | 1.730 | 5.578 | 1.001 |
|  |  | c (Direct effect) | 0.366 | -0.359 | 1.305 | 1.001 |
|  |  | Indirect effect | -1.164 | -2.115 | -0.521 | 1.001 |
|  |  | Total effect | -0.797 | -1.054 | -0.539 | 1.000 |
|  |  | Indirect effect (%) | 149.327 | 60.715 | 276.175 | 1.001 |
| 35 | Yamaguchi | a | -0.115 | -0.127 | -0.105 | 1.083 |
|  |  | b | -89.679 | -352.445 | 18.723 | 5.705 |
|  |  | c (Direct effect) | -10.737 | -42.612 | 1.895 | 5.215 |
|  |  | Indirect effect | 10.433 | -2.158 | 42.299 | 5.223 |
|  |  | Total effect | -0.304 | -0.557 | -0.044 | 1.020 |
|  |  | Indirect effect (%) | 152157.929 | -25788.337 | 1107.747 | 1.000 |
| 36 | Tokushima | a | -0.114 | -0.141 | -0.088 | 1.019 |
|  |  | b | -2.153 | -27.626 | 19.838 | 1.222 |
|  |  | c (Direct effect) | -0.332 | -3.063 | 1.995 | 1.212 |
|  |  | Indirect effect | 0.241 | -2.106 | 2.980 | 1.216 |
|  |  | Total effect | -0.091 | -0.358 | 0.173 | 1.002 |
|  |  | Indirect effect (%) | 109.692 | -6764.055 | 6362.263 | 1.000 |
| 37 | Kagawa | a | -0.294 | -0.340 | -0.247 | 1.107 |
|  |  | b | 18.000 | -18.640 | 49.736 | 3.233 |
|  |  | c (Direct effect) | 4.588 | -6.117 | 14.096 | 3.071 |
|  |  | Indirect effect | -5.239 | -14.756 | 5.454 | 3.080 |
|  |  | Total effect | -0.651 | -0.900 | -0.408 | 1.006 |
|  |  | Indirect effect (%) | 833.556 | -891.086 | 2526.678 | 2.358 |
| 38 | Ehime | a | -0.322 | -0.377 | -0.272 | 1.171 |
|  |  | b | -0.463 | -7.986 | 13.309 | 1.339 |
|  |  | c (Direct effect) | 0.107 | -2.417 | 4.551 | 1.314 |
|  |  | Indirect effect | 0.119 | -4.313 | 2.644 | 1.317 |
|  |  | Total effect | 0.226 | -0.042 | 0.488 | 1.001 |
|  |  | Indirect effect (%) | 834.412 | -4907.718 | 4163.930 | 1.000 |
| 39 | Kochi | a | -0.122 | -0.137 | -0.106 | 1.001 |
|  |  | b | 15.437 | 7.415 | 27.688 | 1.020 |
|  |  | c (Direct effect) | 1.353 | 0.311 | 2.930 | 1.019 |
|  |  | Indirect effect | -1.884 | -3.449 | -0.879 | 1.020 |
|  |  | Total effect | -0.531 | -0.801 | -0.254 | 1.000 |
|  |  | Indirect effect (%) | 382.428 | 149.663 | 876.128 | 1.001 |
| 40 | Fukuoka | a | -0.132 | -0.182 | -0.081 | 1.000 |
|  |  | b | 1.036 | 0.529 | 1.564 | 1.000 |
|  |  | c (Direct effect) | -0.665 | -0.886 | -0.451 | 1.000 |
|  |  | Indirect effect | -0.137 | -0.239 | -0.058 | 1.000 |
|  |  | Total effect | -0.802 | -1.002 | -0.606 | 1.000 |
|  |  | Indirect effect (%) | 17.379 | 6.943 | 31.577 | 1.000 |
| 41 | Saga | a | -0.193 | -0.224 | -0.160 | 1.000 |
|  |  | b | 6.273 | 3.469 | 9.615 | 1.002 |
|  |  | c (Direct effect) | 1.450 | 0.723 | 2.290 | 1.002 |
|  |  | Indirect effect | -1.217 | -1.995 | -0.630 | 1.002 |
|  |  | Total effect | 0.232 | -0.062 | 0.539 | 1.000 |
|  |  | Indirect effect (%) | -509.239 | -4396.314 | 2938.649 | 1.000 |
| 42 | Nagasaki | a | -0.203 | -0.243 | -0.160 | 1.001 |
|  |  | b | 2.978 | 1.785 | 4.285 | 1.004 |
|  |  | c (Direct effect) | 0.393 | 0.036 | 0.776 | 1.002 |
|  |  | Indirect effect | -0.606 | -0.936 | -0.334 | 1.004 |
|  |  | Total effect | -0.213 | -0.435 | 0.017 | 1.000 |
|  |  | Indirect effect (%) | 404.618 | -877.689 | 1955.821 | 1.000 |
| 43 | Kumamoto | a | -0.296 | -0.353 | -0.237 | 1.006 |
|  |  | b | 3.333 | 1.490 | 6.096 | 1.018 |
|  |  | c (Direct effect) | 0.821 | 0.089 | 1.863 | 1.018 |
|  |  | Indirect effect | -1.005 | -2.023 | -0.397 | 1.017 |
|  |  | Total effect | -0.184 | -0.457 | 0.092 | 1.003 |
|  |  | Indirect effect (%) | 207.287 | -4383.063 | 5336.056 | 1.000 |
| 44 | Oita | a | -0.226 | -0.273 | -0.164 | 1.127 |
|  |  | b | -62.585 | -176.139 | 109.931 | 1.986 |
|  |  | c (Direct effect) | -13.893 | -41.641 | 25.278 | 1.831 |
|  |  | Indirect effect | 13.503 | -25.672 | 41.312 | 1.831 |
|  |  | Total effect | -0.391 | -0.622 | -0.157 | 1.002 |
|  |  | Indirect effect (%) | -4045.552 | -17422.204 | 7277.064 | 1.026 |
| 45 | Miyazaki | a | -0.106 | -0.124 | -0.088 | 1.003 |
|  |  | b | 15.065 | 8.228 | 25.453 | 1.041 |
|  |  | c (Direct effect) | 1.455 | 0.609 | 2.656 | 1.035 |
|  |  | Indirect effect | -1.605 | -2.793 | -0.830 | 1.039 |
|  |  | Total effect | -0.150 | -0.467 | 0.172 | 1.000 |
|  |  | Indirect effect (%) | 10892.708 | -9402.130 | 10399.216 | 1.000 |
| 46 | Kagoshima | a | -0.090 | -0.101 | -0.078 | 1.043 |
|  |  | b | 5.669 | -172.269 | 139.613 | 1.681 |
|  |  | c (Direct effect) | 0.216 | -15.695 | 12.672 | 1.676 |
|  |  | Indirect effect | -0.489 | -12.928 | 15.413 | 1.678 |
|  |  | Total effect | -0.273 | -0.599 | 0.049 | 1.007 |
|  |  | Indirect effect (%) | 209.607 | -15429.525 | 17474.853 | 1.000 |
| 47 | Okinawa | a | -0.348 | -0.389 | -0.308 | 1.003 |
|  |  | b | 13.717 | 7.523 | 21.049 | 1.044 |
|  |  | c (Direct effect) | 3.965 | 1.784 | 6.521 | 1.041 |
|  |  | Indirect effect | -4.763 | -7.346 | -2.588 | 1.039 |
|  |  | Total effect | -0.798 | -1.028 | -0.573 | 1.018 |
|  |  | Indirect effect (%) | 608.953 | 310.075 | 994.933 | 1.039 |

**Table S2. Effects of NPIs on mitigating the COVID-19 infection mediated by daytime human mobility in downtown areas during Phase 2**

| **Prefecture Number** | **Prefecture Name** | **Parameters** | **Mean** | **95% Credible Interval** | | **Rhat** |
| --- | --- | --- | --- | --- | --- | --- |
|  |  |  |  | **Low** | **High** |  |
| 1 | Hokkaido | a | -0.086 | -0.125 | -0.034 | 1.035 |
|  |  | b | -4.760 | -16.094 | 0.788 | 1.094 |
|  |  | c (Direct effect) | -0.621 | -2.003 | -0.036 | 1.094 |
|  |  | Indirect effect | 0.496 | -0.044 | 1.863 | 1.095 |
|  |  | Total effect | -0.125 | -0.229 | -0.022 | 1.002 |
|  |  | Indirect effect (%) | -478.058 | -2442.790 | 63.410 | 1.003 |
| 2 | Aomori | a | - | - | - | - |
|  |  | b | - | - | - | - |
|  |  | c (Direct effect) | - | - | - | - |
|  |  | Indirect effect | - | - | - | - |
|  |  | Total effect | - | - | - | - |
|  |  | Indirect effect (%) | - | - | - | - |
| 3 | Iwate | a | - | - | - | - |
|  |  | b | - | - | - | - |
|  |  | c (Direct effect) | - | - | - | - |
|  |  | Indirect effect | - | - | - | - |
|  |  | Total effect | - | - | - | - |
|  |  | Indirect effect (%) | - | - | - | - |
| 4 | Miyagi | a | -0.085 | -0.103 | -0.066 | 1.001 |
|  |  | b | -10.752 | -18.874 | -6.315 | 1.057 |
|  |  | c (Direct effect) | -1.147 | -1.820 | -0.762 | 1.043 |
|  |  | Indirect effect | 0.911 | 0.484 | 1.632 | 1.044 |
|  |  | Total effect | -0.235 | -0.362 | -0.099 | 1.008 |
|  |  | Indirect effect (%) | -483.935 | -1278.702 | -151.903 | 1.000 |
| 5 | Akita | a | - | - | - | - |
|  |  | b | - | - | - | - |
|  |  | c (Direct effect) | - | - | - | - |
|  |  | Indirect effect | - | - | - | - |
|  |  | Total effect | - | - | - | - |
|  |  | Indirect effect (%) | - | - | - | - |
| 6 | Yamagata | a | - | - | - | - |
|  |  | b | - | - | - | - |
|  |  | c (Direct effect) | - | - | - | - |
|  |  | Indirect effect | - | - | - | - |
|  |  | Total effect | - | - | - | - |
|  |  | Indirect effect (%) | - | - | - | - |
| 7 | Fukushima | a | -0.049 | -0.063 | -0.035 | 1.000 |
|  |  | b | -12.301 | -25.807 | -0.727 | 1.002 |
|  |  | c (Direct effect) | -0.951 | -1.603 | -0.372 | 1.002 |
|  |  | Indirect effect | 0.598 | 0.036 | 1.249 | 1.002 |
|  |  | Total effect | -0.352 | -0.520 | -0.185 | 1.000 |
|  |  | Indirect effect (%) | -184.633 | -468.026 | -9.803 | 1.001 |
| 8 | Ibaraki | a | -0.107 | -0.122 | -0.092 | 1.038 |
|  |  | b | -38.921 | -97.862 | 98.557 | 1.519 |
|  |  | c (Direct effect) | -4.352 | -11.104 | 10.305 | 1.512 |
|  |  | Indirect effect | 4.154 | -10.498 | 10.909 | 1.511 |
|  |  | Total effect | -0.197 | -0.332 | -0.062 | 1.001 |
|  |  | Indirect effect (%) | -2575.966 | -10104.091 | 5880.824 | 1.002 |
| 9 | Tochigi | a | -0.057 | -0.066 | -0.049 | 1.140 |
|  |  | b | -41.731 | -691.300 | 199.052 | 2.820 |
|  |  | c (Direct effect) | -2.576 | -39.564 | 11.432 | 2.808 |
|  |  | Indirect effect | 2.457 | -11.498 | 39.447 | 2.808 |
|  |  | Total effect | -0.119 | -0.274 | 0.050 | 1.072 |
|  |  | Indirect effect (%) | 42.062 | -76791.982 | 61686.509 | 1.000 |
| 10 | Gunma | a | -0.052 | -0.064 | -0.040 | 1.005 |
|  |  | b | -27.186 | -82.439 | -0.476 | 1.062 |
|  |  | c (Direct effect) | -1.732 | -4.538 | -0.310 | 1.057 |
|  |  | Indirect effect | 1.425 | 0.022 | 4.241 | 1.058 |
|  |  | Total effect | -0.308 | -0.428 | -0.181 | 1.001 |
|  |  | Indirect effect (%) | -494.186 | -1561.769 | -6.948 | 1.044 |
| 11 | Saitama | a | -0.021 | -0.036 | -0.005 | 1.002 |
|  |  | b | -0.037 | -1.045 | 1.093 | 1.005 |
|  |  | c (Direct effect) | -0.022 | -0.101 | 0.058 | 1.000 |
|  |  | Indirect effect | 0.000 | -0.030 | 0.023 | 1.005 |
|  |  | Total effect | -0.022 | -0.095 | 0.050 | 1.000 |
|  |  | Indirect effect (%) | -14.276 | -346.448 | 326.471 | 1.000 |
| 12 | Chiba | a | -0.017 | -0.033 | 0.001 | 1.038 |
|  |  | b | -0.164 | -0.750 | 0.406 | 1.017 |
|  |  | c (Direct effect) | -0.049 | -0.127 | 0.029 | 1.001 |
|  |  | Indirect effect | 0.003 | -0.007 | 0.016 | 1.010 |
|  |  | Total effect | -0.046 | -0.122 | 0.030 | 1.001 |
|  |  | Indirect effect (%) | -2.089 | -84.414 | 72.313 | 1.000 |
| 13 | Tokyo | a | -0.103 | -0.120 | -0.081 | 1.413 |
|  |  | b | 8.514 | -3.232 | 31.151 | 3.763 |
|  |  | c (Direct effect) | 0.903 | -0.367 | 3.215 | 3.450 |
|  |  | Indirect effect | -0.898 | -3.209 | 0.356 | 3.471 |
|  |  | Total effect | 0.005 | -0.042 | 0.055 | 1.116 |
|  |  | Indirect effect (%) | -5662.991 | -84106.331 | 69110.652 | 1.000 |
| 14 | Kanagawa | a | -0.044 | -0.059 | -0.027 | 1.001 |
|  |  | b | -0.443 | -2.384 | 1.452 | 1.039 |
|  |  | c (Direct effect) | -0.128 | -0.241 | -0.013 | 1.018 |
|  |  | Indirect effect | 0.019 | -0.071 | 0.110 | 1.042 |
|  |  | Total effect | -0.109 | -0.184 | -0.035 | 1.001 |
|  |  | Indirect effect (%) | -18.091 | -144.400 | 80.802 | 1.001 |
| 15 | Niigata | a | - | - | - | - |
|  |  | b | - | - | - | - |
|  |  | c (Direct effect) | - | - | - | - |
|  |  | Indirect effect | - | - | - | - |
|  |  | Total effect | - | - | - | - |
|  |  | Indirect effect (%) | - | - | - | - |
| 16 | Toyama | a | -0.085 | -0.112 | -0.055 | 1.003 |
|  |  | b | -12.452 | -105.573 | 54.302 | 1.121 |
|  |  | c (Direct effect) | -1.560 | -9.716 | 4.164 | 1.117 |
|  |  | Indirect effect | 1.067 | -4.641 | 9.224 | 1.118 |
|  |  | Total effect | -0.493 | -0.711 | -0.276 | 1.000 |
|  |  | Indirect effect (%) | -228.071 | -2038.815 | 995.370 | 1.106 |
| 17 | Ishikawa | a | -0.021 | -0.033 | -0.008 | 1.003 |
|  |  | b | 2.078 | -0.873 | 4.949 | 1.003 |
|  |  | c (Direct effect) | -0.213 | -0.404 | -0.021 | 1.001 |
|  |  | Indirect effect | -0.044 | -0.125 | 0.017 | 1.004 |
|  |  | Total effect | -0.257 | -0.411 | -0.099 | 1.000 |
|  |  | Indirect effect (%) | 32.826 | -5.297 | 80.278 | 1.000 |
| 18 | Fukui | a | - | - | - | - |
|  |  | b | - | - | - | - |
|  |  | c (Direct effect) | - | - | - | - |
|  |  | Indirect effect | - | - | - | - |
|  |  | Total effect | - | - | - | - |
|  |  | Indirect effect (%) | - | - | - | - |
| 19 | Yamanashi | a | -0.032 | -0.050 | -0.014 | 1.000 |
|  |  | b | 1.712 | -2.730 | 6.490 | 1.002 |
|  |  | c (Direct effect) | -0.150 | -0.472 | 0.187 | 1.001 |
|  |  | Indirect effect | -0.056 | -0.248 | 0.095 | 1.002 |
|  |  | Total effect | -0.206 | -0.441 | 0.034 | 1.000 |
|  |  | Indirect effect (%) | 26.575 | -214.893 | 381.231 | 1.000 |
| 20 | Nagano | a | - | - | - | - |
|  |  | b | - | - | - | - |
|  |  | c (Direct effect) | - | - | - | - |
|  |  | Indirect effect | - | - | - | - |
|  |  | Total effect | - | - | - | - |
|  |  | Indirect effect (%) | - | - | - | - |
| 21 | Gifu | a | -0.032 | -0.041 | -0.022 | 1.006 |
|  |  | b | -19.772 | -29.227 | -13.545 | 1.027 |
|  |  | c (Direct effect) | -0.943 | -1.308 | -0.695 | 1.023 |
|  |  | Indirect effect | 0.626 | 0.357 | 1.014 | 1.027 |
|  |  | Total effect | -0.317 | -0.425 | -0.205 | 1.004 |
|  |  | Indirect effect (%) | -208.779 | -419.441 | -94.875 | 1.018 |
| 22 | Shizuoka | a | -0.140 | -0.155 | -0.124 | 1.002 |
|  |  | b | 37.036 | -1.623 | 84.753 | 1.145 |
|  |  | c (Direct effect) | 5.181 | -0.251 | 12.060 | 1.141 |
|  |  | Indirect effect | -5.189 | -12.060 | 0.226 | 1.141 |
|  |  | Total effect | -0.007 | -0.171 | 0.160 | 1.001 |
|  |  | Indirect effect (%) | 9510.061 | -102155.103 | 103712.920 | 1.000 |
| 23 | Aichi | a | -0.097 | -0.118 | -0.077 | 1.001 |
|  |  | b | -2.747 | -5.733 | -0.765 | 1.009 |
|  |  | c (Direct effect) | -0.287 | -0.620 | -0.074 | 1.008 |
|  |  | Indirect effect | 0.272 | 0.068 | 0.604 | 1.008 |
|  |  | Total effect | -0.015 | -0.078 | 0.046 | 1.000 |
|  |  | Indirect effect (%) | 12702.604 | -11800.265 | 12721.598 | 1.000 |
| 24 | Mie | a | -0.023 | -0.031 | -0.015 | 1.027 |
|  |  | b | -8.647 | -712.963 | 503.729 | 1.955 |
|  |  | c (Direct effect) | -0.565 | -17.301 | 12.299 | 1.852 |
|  |  | Indirect effect | 0.195 | -12.665 | 16.930 | 1.852 |
|  |  | Total effect | -0.370 | -0.464 | -0.275 | 1.004 |
|  |  | Indirect effect (%) | -49.056 | -4686.175 | 3531.778 | 1.810 |
| 25 | Shiga | a | -0.021 | -0.026 | -0.015 | 1.001 |
|  |  | b | -80.045 | -220.828 | -30.788 | 1.053 |
|  |  | c (Direct effect) | -1.748 | -4.742 | -0.695 | 1.051 |
|  |  | Indirect effect | 1.651 | 0.595 | 4.623 | 1.051 |
|  |  | Total effect | -0.097 | -0.273 | 0.088 | 1.001 |
|  |  | Indirect effect (%) | -2924.581 | -17156.083 | 14303.276 | 1.000 |
| 26 | Kyoto | a | -0.030 | -0.049 | -0.011 | 1.023 |
|  |  | b | 0.035 | -0.530 | 0.620 | 1.001 |
|  |  | c (Direct effect) | -0.289 | -0.385 | -0.187 | 1.004 |
|  |  | Indirect effect | -0.001 | -0.020 | 0.018 | 1.002 |
|  |  | Total effect | -0.290 | -0.381 | -0.193 | 1.003 |
|  |  | Indirect effect (%) | 0.450 | -6.337 | 7.963 | 1.001 |
| 27 | Osaka | a | -0.105 | -0.137 | -0.056 | 1.211 |
|  |  | b | 7.190 | -0.243 | 22.617 | 1.681 |
|  |  | c (Direct effect) | 0.747 | -0.153 | 2.496 | 1.724 |
|  |  | Indirect effect | -0.857 | -2.603 | 0.019 | 1.732 |
|  |  | Total effect | -0.110 | -0.178 | -0.041 | 1.011 |
|  |  | Indirect effect (%) | 834.057 | -19.436 | 3080.627 | 1.008 |
| 28 | Hyogo | a | -0.035 | -0.059 | -0.011 | 1.001 |
|  |  | b | 0.008 | -0.485 | 0.658 | 1.005 |
|  |  | c (Direct effect) | -0.144 | -0.210 | -0.077 | 1.001 |
|  |  | Indirect effect | -0.001 | -0.028 | 0.018 | 1.005 |
|  |  | Total effect | -0.145 | -0.209 | -0.080 | 1.001 |
|  |  | Indirect effect (%) | 0.401 | -14.734 | 20.066 | 1.005 |
| 29 | Nara | a | - | - | - | - |
|  |  | b | - | - | - | - |
|  |  | c (Direct effect) | - | - | - | - |
|  |  | Indirect effect | - | - | - | - |
|  |  | Total effect | - | - | - | - |
|  |  | Indirect effect (%) | - | - | - | - |
| 30 | Wakayama | a | - | - | - | - |
|  |  | b | - | - | - | - |
|  |  | c (Direct effect) | - | - | - | - |
|  |  | Indirect effect | - | - | - | - |
|  |  | Total effect | - | - | - | - |
|  |  | Indirect effect (%) | - | - | - | - |
| 31 | Tottori | a | - | - | - | - |
|  |  | b | - | - | - | - |
|  |  | c (Direct effect) | - | - | - | - |
|  |  | Indirect effect | - | - | - | - |
|  |  | Total effect | - | - | - | - |
|  |  | Indirect effect (%) | - | - | - | - |
| 32 | Shimane | a | - | - | - | - |
|  |  | b | - | - | - | - |
|  |  | c (Direct effect) | - | - | - | - |
|  |  | Indirect effect | - | - | - | - |
|  |  | Total effect | - | - | - | - |
|  |  | Indirect effect (%) | - | - | - | - |
| 33 | Okayama | a | -0.047 | -0.061 | -0.033 | 1.019 |
|  |  | b | 0.088 | -0.916 | 1.115 | 1.007 |
|  |  | c (Direct effect) | -0.335 | -0.496 | -0.187 | 1.005 |
|  |  | Indirect effect | -0.004 | -0.054 | 0.044 | 1.006 |
|  |  | Total effect | -0.339 | -0.496 | -0.198 | 1.008 |
|  |  | Indirect effect (%) | 1.236 | -14.303 | 17.459 | 1.006 |
| 34 | Hiroshima | a | -0.071 | -0.090 | -0.052 | 1.001 |
|  |  | b | -8.354 | -12.095 | -5.921 | 1.026 |
|  |  | c (Direct effect) | -1.029 | -1.366 | -0.783 | 1.015 |
|  |  | Indirect effect | 0.600 | 0.345 | 0.961 | 1.016 |
|  |  | Total effect | -0.430 | -0.544 | -0.315 | 1.001 |
|  |  | Indirect effect (%) | -143.954 | -263.761 | -71.474 | 1.011 |
| 35 | Yamaguchi | a | - | - | - | - |
|  |  | b | - | - | - | - |
|  |  | c (Direct effect) | - | - | - | - |
|  |  | Indirect effect | - | - | - | - |
|  |  | Total effect | - | - | - | - |
|  |  | Indirect effect (%) | - | - | - | - |
| 36 | Tokushima | a | - | - | - | - |
|  |  | b | - | - | - | - |
|  |  | c (Direct effect) | - | - | - | - |
|  |  | Indirect effect | - | - | - | - |
|  |  | Total effect | - | - | - | - |
|  |  | Indirect effect (%) | - | - | - | - |
| 37 | Kagawa | a | -0.066 | -0.097 | -0.034 | 1.001 |
|  |  | b | -9.740 | -17.888 | -4.876 | 1.029 |
|  |  | c (Direct effect) | -1.126 | -1.794 | -0.708 | 1.017 |
|  |  | Indirect effect | 0.643 | 0.234 | 1.327 | 1.017 |
|  |  | Total effect | -0.483 | -0.680 | -0.283 | 1.000 |
|  |  | Indirect effect (%) | -143.634 | -359.433 | -42.546 | 1.011 |
| 38 | Ehime | a | -0.132 | -0.156 | -0.102 | 1.068 |
|  |  | b | -31.302 | -101.740 | 63.186 | 1.111 |
|  |  | c (Direct effect) | -4.564 | -13.978 | 8.018 | 1.113 |
|  |  | Indirect effect | 4.149 | -8.404 | 13.562 | 1.113 |
|  |  | Total effect | -0.415 | -0.612 | -0.224 | 1.019 |
|  |  | Indirect effect (%) | -1067.482 | -3742.998 | 2182.329 | 1.080 |
| 39 | Kochi | a | -0.045 | -0.056 | -0.033 | 1.071 |
|  |  | b | 1.228 | -290.763 | 453.613 | 1.570 |
|  |  | c (Direct effect) | -0.351 | -13.491 | 20.723 | 1.554 |
|  |  | Indirect effect | -0.030 | -21.057 | 13.126 | 1.553 |
|  |  | Total effect | -0.382 | -0.676 | -0.094 | 1.017 |
|  |  | Indirect effect (%) | 311.300 | -4928.433 | 7749.051 | 1.000 |
| 40 | Fukuoka | a | -0.043 | -0.067 | -0.018 | 1.000 |
|  |  | b | 1.433 | 0.110 | 3.021 | 1.002 |
|  |  | c (Direct effect) | -0.208 | -0.321 | -0.077 | 1.001 |
|  |  | Indirect effect | -0.062 | -0.159 | -0.004 | 1.001 |
|  |  | Total effect | -0.270 | -0.357 | -0.183 | 1.000 |
|  |  | Indirect effect (%) | 23.865 | 1.440 | 64.499 | 1.001 |
| 41 | Saga | a | 0.014 | -0.004 | 0.032 | 1.000 |
|  |  | b | -2.825 | -5.760 | 0.048 | 1.000 |
|  |  | c (Direct effect) | -0.435 | -0.684 | -0.181 | 1.000 |
|  |  | Indirect effect | -0.038 | -0.116 | 0.015 | 1.000 |
|  |  | Total effect | -0.473 | -0.720 | -0.222 | 1.000 |
|  |  | Indirect effect (%) | 8.753 | -3.556 | 28.737 | 1.000 |
| 42 | Nagasaki | a | 0.009 | -0.011 | 0.029 | 1.000 |
|  |  | b | 2.698 | 0.818 | 4.678 | 1.001 |
|  |  | c (Direct effect) | -0.182 | -0.436 | 0.075 | 1.000 |
|  |  | Indirect effect | 0.024 | -0.030 | 0.091 | 1.000 |
|  |  | Total effect | -0.158 | -0.417 | 0.106 | 1.000 |
|  |  | Indirect effect (%) | -3.736 | -212.564 | 178.054 | 1.000 |
| 43 | Kumamoto | a | -0.056 | -0.074 | -0.037 | 1.000 |
|  |  | b | -1.315 | -2.652 | 0.036 | 1.002 |
|  |  | c (Direct effect) | -0.434 | -0.615 | -0.257 | 1.000 |
|  |  | Indirect effect | 0.073 | -0.002 | 0.162 | 1.002 |
|  |  | Total effect | -0.361 | -0.517 | -0.202 | 1.000 |
|  |  | Indirect effect (%) | -21.424 | -54.399 | 0.519 | 1.002 |
| 44 | Oita | a | - | - | - | - |
|  |  | b | - | - | - | - |
|  |  | c (Direct effect) | - | - | - | - |
|  |  | Indirect effect | - | - | - | - |
|  |  | Total effect | - | - | - | - |
|  |  | Indirect effect (%) | - | - | - | - |
| 45 | Miyazaki | a | -0.038 | -0.047 | -0.028 | 1.028 |
|  |  | b | -336.586 | -3349.312 | 1197.401 | 3.066 |
|  |  | c (Direct effect) | -13.563 | -132.814 | 50.671 | 2.865 |
|  |  | Indirect effect | 13.078 | -51.143 | 132.330 | 2.865 |
|  |  | Total effect | -0.484 | -0.760 | -0.208 | 1.000 |
|  |  | Indirect effect (%) | -2849.834 | -34841.902 | 15115.186 | 1.017 |
| 46 | Kagoshima | a | -0.034 | -0.043 | -0.025 | 1.001 |
|  |  | b | -36.135 | -70.886 | -17.570 | 1.009 |
|  |  | c (Direct effect) | -1.814 | -3.182 | -1.078 | 1.009 |
|  |  | Indirect effect | 1.256 | 0.526 | 2.653 | 1.009 |
|  |  | Total effect | -0.558 | -0.771 | -0.344 | 1.000 |
|  |  | Indirect effect (%) | -239.705 | -593.172 | -84.595 | 1.006 |
| 47 | Okinawa | a | -0.085 | -0.100 | -0.068 | 1.045 |
|  |  | b | -11.426 | -78.789 | 6.834 | 1.647 |
|  |  | c (Direct effect) | -0.999 | -6.580 | 0.519 | 1.621 |
|  |  | Indirect effect | 0.962 | -0.549 | 6.538 | 1.619 |
|  |  | Total effect | -0.037 | -0.168 | 0.099 | 1.167 |
|  |  | Indirect effect (%) | 854.126 | -22147.251 | 19347.561 | 1.000 |

Prefectures where NPIs were not implemented are represented by "-".

**Table S3. Effects of NPIs on mitigating the COVID-19 infection mediated by daytime human mobility in downtown areas during Phase 3**

| **Prefecture Number** | **Prefecture Name** | **Parameters** | **Mean** | **95% Credible Interval** | | **Rhat** |
| --- | --- | --- | --- | --- | --- | --- |
|  |  |  |  | **Low** | **High** |  |
| 1 | Hokkaido | a | -0.093 | -0.119 | -0.066 | 1.004 |
|  |  | b | -10.006 | -13.231 | -7.762 | 1.026 |
|  |  | c (Direct effect) | -1.124 | -1.519 | -0.822 | 1.015 |
|  |  | Indirect effect | 0.928 | 0.595 | 1.353 | 1.014 |
|  |  | Total effect | -0.196 | -0.321 | -0.073 | 1.002 |
|  |  | Indirect effect (%) | -562.719 | -1505.368 | -210.553 | 1.000 |
| 2 | Aomori | a | -0.013 | -0.027 | 0.000 | 1.024 |
|  |  | b | -10.407 | -58.191 | 37.782 | 3.672 |
|  |  | c (Direct effect) | -0.218 | -1.034 | 0.572 | 1.898 |
|  |  | Indirect effect | 0.148 | -0.627 | 0.981 | 1.992 |
|  |  | Total effect | -0.070 | -0.269 | 0.132 | 1.008 |
|  |  | Indirect effect (%) | -1321.207 | -5115.841 | 5349.628 | 1.000 |
| 3 | Iwate | a | - | - | - | - |
|  |  | b | - | - | - | - |
|  |  | c (Direct effect) | - | - | - | - |
|  |  | Indirect effect | - | - | - | - |
|  |  | Total effect | - | - | - | - |
|  |  | Indirect effect (%) | - | - | - | - |
| 4 | Miyagi | a | - | - | - | - |
|  |  | b | - | - | - | - |
|  |  | c (Direct effect) | - | - | - | - |
|  |  | Indirect effect | - | - | - | - |
|  |  | Total effect | - | - | - | - |
|  |  | Indirect effect (%) | - | - | - | - |
| 5 | Akita | a | - | - | - | - |
|  |  | b | - | - | - | - |
|  |  | c (Direct effect) | - | - | - | - |
|  |  | Indirect effect | - | - | - | - |
|  |  | Total effect | - | - | - | - |
|  |  | Indirect effect (%) | - | - | - | - |
| 6 | Yamagata | a | -0.005 | -0.019 | 0.008 | 1.773 |
|  |  | b | 46.303 | -55.378 | 316.931 | 52.459 |
|  |  | c (Direct effect) | 0.664 | -0.890 | 5.041 | 3.170 |
|  |  | Indirect effect | -0.863 | -5.267 | 0.738 | 3.199 |
|  |  | Total effect | -0.199 | -0.397 | -0.021 | 1.169 |
|  |  | Indirect effect (%) | 1713.962 | -1226.220 | 3789.345 | 1.000 |
| 7 | Fukushima | a | -0.026 | -0.040 | -0.014 | 1.002 |
|  |  | b | 2.869 | -346.501 | 485.572 | 1.413 |
|  |  | c (Direct effect) | -0.209 | -10.077 | 10.974 | 1.370 |
|  |  | Indirect effect | -0.129 | -11.327 | 9.730 | 1.370 |
|  |  | Total effect | -0.338 | -0.505 | -0.171 | 1.001 |
|  |  | Indirect effect (%) | 48.403 | -3230.684 | 3559.072 | 1.188 |
| 8 | Ibaraki | a | -0.025 | -0.046 | -0.003 | 1.024 |
|  |  | b | -15.165 | -19.269 | -12.446 | 1.039 |
|  |  | c (Direct effect) | -0.551 | -0.852 | -0.284 | 1.018 |
|  |  | Indirect effect | 0.374 | 0.049 | 0.720 | 1.031 |
|  |  | Total effect | -0.177 | -0.356 | -0.001 | 1.020 |
|  |  | Indirect effect (%) | -898.337 | -1837.401 | 39.546 | 1.000 |
| 9 | Tochigi | a | -0.028 | -0.038 | -0.019 | 1.003 |
|  |  | b | -115.484 | -284.199 | 79.020 | 1.596 |
|  |  | c (Direct effect) | -3.545 | -9.210 | 1.951 | 1.530 |
|  |  | Indirect effect | 3.296 | -2.191 | 8.964 | 1.530 |
|  |  | Total effect | -0.248 | -0.366 | -0.130 | 1.000 |
|  |  | Indirect effect (%) | -1430.230 | -4479.145 | 913.602 | 1.172 |
| 10 | Gunma | a | -0.033 | -0.044 | -0.022 | 1.021 |
|  |  | b | -24.392 | -79.424 | 4.301 | 2.102 |
|  |  | c (Direct effect) | -0.969 | -2.829 | -0.040 | 1.869 |
|  |  | Indirect effect | 0.799 | -0.133 | 2.660 | 1.867 |
|  |  | Total effect | -0.170 | -0.282 | -0.055 | 1.046 |
|  |  | Indirect effect (%) | -544.945 | -2219.286 | 93.566 | 1.001 |
| 11 | Saitama | a | -0.027 | -0.048 | -0.004 | 1.003 |
|  |  | b | -5.490 | -7.060 | -4.147 | 1.062 |
|  |  | c (Direct effect) | -0.424 | -0.595 | -0.277 | 1.002 |
|  |  | Indirect effect | 0.150 | 0.021 | 0.301 | 1.011 |
|  |  | Total effect | -0.275 | -0.409 | -0.147 | 1.016 |
|  |  | Indirect effect (%) | -61.244 | -157.313 | -6.429 | 1.007 |
| 12 | Chiba | a | -0.026 | -0.052 | -0.001 | 1.006 |
|  |  | b | -1.982 | -2.640 | -1.303 | 1.003 |
|  |  | c (Direct effect) | -0.379 | -0.501 | -0.253 | 1.010 |
|  |  | Indirect effect | 0.052 | 0.001 | 0.113 | 1.005 |
|  |  | Total effect | -0.327 | -0.455 | -0.198 | 1.007 |
|  |  | Indirect effect (%) | -17.280 | -45.813 | -0.357 | 1.002 |
| 13 | Tokyo | a | -0.015 | -0.067 | 0.037 | 1.001 |
|  |  | b | -1.923 | -2.626 | -1.439 | 1.006 |
|  |  | c (Direct effect) | -0.332 | -0.438 | -0.231 | 1.000 |
|  |  | Indirect effect | 0.030 | -0.067 | 0.136 | 1.001 |
|  |  | Total effect | -0.301 | -0.413 | -0.190 | 1.000 |
|  |  | Indirect effect (%) | -12.463 | -60.139 | 18.877 | 1.001 |
| 14 | Kanagawa | a | -0.020 | -0.047 | 0.008 | 1.001 |
|  |  | b | -4.497 | -5.460 | -3.683 | 1.005 |
|  |  | c (Direct effect) | -0.418 | -0.538 | -0.305 | 1.003 |
|  |  | Indirect effect | 0.089 | -0.033 | 0.221 | 1.002 |
|  |  | Total effect | -0.329 | -0.463 | -0.194 | 1.000 |
|  |  | Indirect effect (%) | -31.371 | -98.851 | 8.193 | 1.001 |
| 15 | Niigata | a | -0.059 | -0.089 | -0.029 | 1.000 |
|  |  | b | -1.897 | -3.119 | -0.794 | 1.001 |
|  |  | c (Direct effect) | -0.514 | -0.683 | -0.347 | 1.000 |
|  |  | Indirect effect | 0.114 | 0.034 | 0.229 | 1.001 |
|  |  | Total effect | -0.400 | -0.550 | -0.245 | 1.000 |
|  |  | Indirect effect (%) | -30.176 | -72.175 | -8.064 | 1.001 |
| 16 | Toyama | a | - | - | - | - |
|  |  | b | - | - | - | - |
|  |  | c (Direct effect) | - | - | - | - |
|  |  | Indirect effect | - | - | - | - |
|  |  | Total effect | - | - | - | - |
|  |  | Indirect effect (%) | - | - | - | - |
| 17 | Ishikawa | a | -0.030 | -0.042 | -0.018 | 1.000 |
|  |  | b | -2.600 | -5.769 | 0.301 | 1.003 |
|  |  | c (Direct effect) | -0.598 | -0.806 | -0.392 | 1.001 |
|  |  | Indirect effect | 0.080 | -0.008 | 0.204 | 1.003 |
|  |  | Total effect | -0.518 | -0.674 | -0.360 | 1.000 |
|  |  | Indirect effect (%) | -15.601 | -40.751 | 1.706 | 1.003 |
| 18 | Fukui | a | - | - | - | - |
|  |  | b | - | - | - | - |
|  |  | c (Direct effect) | - | - | - | - |
|  |  | Indirect effect | - | - | - | - |
|  |  | Total effect | - | - | - | - |
|  |  | Indirect effect (%) | - | - | - | - |
| 19 | Yamanashi | a | - | - | - | - |
|  |  | b | - | - | - | - |
|  |  | c (Direct effect) | - | - | - | - |
|  |  | Indirect effect | - | - | - | - |
|  |  | Total effect | - | - | - | - |
|  |  | Indirect effect (%) | - | - | - | - |
| 20 | Nagano | a | -0.051 | -0.066 | -0.037 | 1.020 |
|  |  | b | -11.679 | -20.191 | -7.198 | 1.028 |
|  |  | c (Direct effect) | -0.812 | -1.336 | -0.518 | 1.027 |
|  |  | Indirect effect | 0.601 | 0.312 | 1.118 | 1.028 |
|  |  | Total effect | -0.211 | -0.350 | -0.074 | 1.001 |
|  |  | Indirect effect (%) | -344.060 | -962.695 | -110.145 | 1.000 |
| 21 | Gifu | a | -0.005 | -0.018 | 0.009 | 1.001 |
|  |  | b | -28.999 | -38.869 | -21.564 | 1.008 |
|  |  | c (Direct effect) | -0.545 | -0.874 | -0.248 | 1.001 |
|  |  | Indirect effect | 0.136 | -0.250 | 0.549 | 1.001 |
|  |  | Total effect | -0.409 | -0.661 | -0.145 | 1.000 |
|  |  | Indirect effect (%) | -62.398 | -300.424 | 46.478 | 1.000 |
| 22 | Shizuoka | a | -0.039 | -0.063 | -0.014 | 1.020 |
|  |  | b | -6.925 | -8.587 | -5.506 | 1.007 |
|  |  | c (Direct effect) | -0.546 | -0.761 | -0.346 | 1.015 |
|  |  | Indirect effect | 0.270 | 0.089 | 0.488 | 1.022 |
|  |  | Total effect | -0.275 | -0.453 | -0.091 | 1.006 |
|  |  | Indirect effect (%) | -119.919 | -393.656 | -24.189 | 1.000 |
| 23 | Aichi | a | -0.035 | -0.071 | 0.004 | 1.004 |
|  |  | b | -3.862 | -5.021 | -2.862 | 1.015 |
|  |  | c (Direct effect) | -0.463 | -0.627 | -0.313 | 1.002 |
|  |  | Indirect effect | 0.136 | -0.015 | 0.302 | 1.007 |
|  |  | Total effect | -0.327 | -0.462 | -0.198 | 1.010 |
|  |  | Indirect effect (%) | -46.568 | -123.938 | 3.882 | 1.012 |
| 24 | Mie | a | -0.017 | -0.029 | -0.004 | 1.015 |
|  |  | b | -26.065 | -33.393 | -21.159 | 1.069 |
|  |  | c (Direct effect) | -0.649 | -0.967 | -0.330 | 1.024 |
|  |  | Indirect effect | 0.445 | 0.088 | 0.791 | 1.026 |
|  |  | Total effect | -0.205 | -0.357 | -0.050 | 1.018 |
|  |  | Indirect effect (%) | -319.402 | -1119.451 | -25.672 | 1.000 |
| 25 | Shiga | a | - | - | - | - |
|  |  | b | - | - | - | - |
|  |  | c (Direct effect) | - | - | - | - |
|  |  | Indirect effect | - | - | - | - |
|  |  | Total effect | - | - | - | - |
|  |  | Indirect effect (%) | - | - | - | - |
| 26 | Kyoto | a | -0.039 | -0.059 | -0.018 | 1.003 |
|  |  | b | -0.876 | -1.655 | -0.068 | 1.005 |
|  |  | c (Direct effect) | -0.319 | -0.422 | -0.217 | 1.000 |
|  |  | Indirect effect | 0.035 | 0.002 | 0.083 | 1.003 |
|  |  | Total effect | -0.284 | -0.384 | -0.188 | 1.001 |
|  |  | Indirect effect (%) | -12.710 | -34.199 | -0.775 | 1.004 |
| 27 | Osaka | a | -0.053 | -0.090 | -0.016 | 1.066 |
|  |  | b | -3.601 | -4.977 | -2.754 | 1.071 |
|  |  | c (Direct effect) | -0.443 | -0.583 | -0.313 | 1.005 |
|  |  | Indirect effect | 0.193 | 0.052 | 0.341 | 1.034 |
|  |  | Total effect | -0.250 | -0.354 | -0.140 | 1.038 |
|  |  | Indirect effect (%) | -85.590 | -208.979 | -17.123 | 1.015 |
| 28 | Hyogo | a | -0.049 | -0.069 | -0.028 | 1.004 |
|  |  | b | -6.937 | -8.933 | -5.230 | 1.002 |
|  |  | c (Direct effect) | -0.619 | -0.820 | -0.439 | 1.001 |
|  |  | Indirect effect | 0.339 | 0.171 | 0.544 | 1.002 |
|  |  | Total effect | -0.280 | -0.409 | -0.145 | 1.005 |
|  |  | Indirect effect (%) | -134.617 | -305.348 | -49.742 | 1.003 |
| 29 | Nara | a | - | - | - | - |
|  |  | b | - | - | - | - |
|  |  | c (Direct effect) | - | - | - | - |
|  |  | Indirect effect | - | - | - | - |
|  |  | Total effect | - | - | - | - |
|  |  | Indirect effect (%) | - | - | - | - |
| 30 | Wakayama | a | -0.051 | -0.068 | -0.034 | 1.005 |
|  |  | b | -25.960 | -35.321 | -20.173 | 1.097 |
|  |  | c (Direct effect) | -1.392 | -1.974 | -0.924 | 1.029 |
|  |  | Indirect effect | 1.311 | 0.818 | 1.912 | 1.022 |
|  |  | Total effect | -0.080 | -0.235 | 0.076 | 1.006 |
|  |  | Indirect effect (%) | -4297.968 | -17489.557 | 15036.093 | 1.000 |
| 31 | Tottori | a | - | - | - | - |
|  |  | b | - | - | - | - |
|  |  | c (Direct effect) | - | - | - | - |
|  |  | Indirect effect | - | - | - | - |
|  |  | Total effect | - | - | - | - |
|  |  | Indirect effect (%) | - | - | - | - |
| 32 | Shimane | a | -0.008 | -0.033 | 0.017 | 1.001 |
|  |  | b | -34.972 | -72.701 | -19.356 | 1.041 |
|  |  | c (Direct effect) | -0.699 | -1.807 | 0.170 | 1.005 |
|  |  | Indirect effect | 0.308 | -0.573 | 1.416 | 1.004 |
|  |  | Total effect | -0.391 | -0.618 | -0.161 | 1.001 |
|  |  | Indirect effect (%) | -98.550 | -518.653 | 148.036 | 1.000 |
| 33 | Okayama | a | -0.063 | -0.086 | -0.044 | 1.070 |
|  |  | b | -0.640 | -3.065 | 2.270 | 1.032 |
|  |  | c (Direct effect) | -0.151 | -0.454 | 0.111 | 1.070 |
|  |  | Indirect effect | 0.039 | -0.154 | 0.217 | 1.018 |
|  |  | Total effect | -0.112 | -0.361 | 0.082 | 1.173 |
|  |  | Indirect effect (%) | -36.355 | -846.362 | 635.383 | 1.000 |
| 34 | Hiroshima | a | -0.079 | -0.098 | -0.059 | 1.006 |
|  |  | b | -26.695 | -35.721 | -19.508 | 1.082 |
|  |  | c (Direct effect) | -2.858 | -3.892 | -2.057 | 1.057 |
|  |  | Indirect effect | 2.108 | 1.287 | 3.164 | 1.055 |
|  |  | Total effect | -0.750 | -0.924 | -0.573 | 1.000 |
|  |  | Indirect effect (%) | -287.044 | -469.606 | -156.524 | 1.036 |
| 35 | Yamaguchi | a | -0.018 | -0.032 | -0.004 | 1.002 |
|  |  | b | -11.443 | -18.358 | -7.153 | 1.025 |
|  |  | c (Direct effect) | -0.566 | -0.809 | -0.375 | 1.009 |
|  |  | Indirect effect | 0.213 | 0.042 | 0.450 | 1.012 |
|  |  | Total effect | -0.353 | -0.473 | -0.232 | 1.001 |
|  |  | Indirect effect (%) | -63.497 | -152.240 | -11.050 | 1.010 |
| 36 | Tokushima | a | - | - | - | - |
|  |  | b | - | - | - | - |
|  |  | c (Direct effect) | - | - | - | - |
|  |  | Indirect effect | - | - | - | - |
|  |  | Total effect | - | - | - | - |
|  |  | Indirect effect (%) | - | - | - | - |
| 37 | Kagawa | a | -0.028 | -0.055 | 0.005 | 1.080 |
|  |  | b | -8.574 | -11.112 | -6.932 | 1.083 |
|  |  | c (Direct effect) | -0.440 | -0.678 | -0.182 | 1.137 |
|  |  | Indirect effect | 0.242 | -0.042 | 0.507 | 1.057 |
|  |  | Total effect | -0.199 | -0.319 | -0.059 | 1.055 |
|  |  | Indirect effect (%) | -151.749 | -598.405 | 19.901 | 1.000 |
| 38 | Ehime | a | - | - | - | - |
|  |  | b | - | - | - | - |
|  |  | c (Direct effect) | - | - | - | - |
|  |  | Indirect effect | - | - | - | - |
|  |  | Total effect | - | - | - | - |
|  |  | Indirect effect (%) | - | - | - | - |
| 39 | Kochi | a | -0.031 | -0.043 | -0.018 | 1.001 |
|  |  | b | -19.086 | -29.400 | -12.530 | 1.012 |
|  |  | c (Direct effect) | -0.610 | -1.116 | -0.280 | 1.011 |
|  |  | Indirect effect | 0.591 | 0.270 | 1.096 | 1.010 |
|  |  | Total effect | -0.019 | -0.160 | 0.121 | 1.000 |
|  |  | Indirect effect (%) | 3633.739 | -13313.433 | 12051.168 | 1.000 |
| 40 | Fukuoka | a | -0.076 | -0.105 | -0.047 | 1.000 |
|  |  | b | -6.510 | -8.743 | -4.806 | 1.002 |
|  |  | c (Direct effect) | -0.803 | -1.080 | -0.570 | 1.000 |
|  |  | Indirect effect | 0.501 | 0.260 | 0.804 | 1.001 |
|  |  | Total effect | -0.302 | -0.480 | -0.122 | 1.000 |
|  |  | Indirect effect (%) | -207.198 | -531.582 | -63.967 | 1.000 |
| 41 | Saga | a | -0.048 | -0.061 | -0.034 | 1.005 |
|  |  | b | 26.658 | 16.227 | 36.664 | 1.009 |
|  |  | c (Direct effect) | 1.174 | 0.652 | 1.822 | 1.003 |
|  |  | Indirect effect | -1.267 | -1.906 | -0.731 | 1.003 |
|  |  | Total effect | -0.092 | -0.267 | 0.084 | 1.002 |
|  |  | Indirect effect (%) | 750.812 | -12035.191 | 13818.947 | 1.000 |
| 42 | Nagasaki | a | -0.074 | -0.091 | -0.057 | 1.000 |
|  |  | b | -6.359 | -10.188 | -2.491 | 1.001 |
|  |  | c (Direct effect) | -0.906 | -1.249 | -0.580 | 1.001 |
|  |  | Indirect effect | 0.475 | 0.172 | 0.828 | 1.001 |
|  |  | Total effect | -0.431 | -0.617 | -0.237 | 1.000 |
|  |  | Indirect effect (%) | -120.789 | -276.909 | -35.932 | 1.001 |
| 43 | Kumamoto | a | -0.068 | -0.094 | -0.041 | 1.002 |
|  |  | b | -9.244 | -11.878 | -6.872 | 1.005 |
|  |  | c (Direct effect) | -1.129 | -1.502 | -0.758 | 1.006 |
|  |  | Indirect effect | 0.635 | 0.337 | 1.007 | 1.001 |
|  |  | Total effect | -0.494 | -0.789 | -0.170 | 1.008 |
|  |  | Indirect effect (%) | -154.164 | -464.566 | -50.545 | 1.003 |
| 44 | Oita | a | -0.004 | -0.050 | 0.040 | 1.002 |
|  |  | b | -10.440 | -13.063 | -8.672 | 1.080 |
|  |  | c (Direct effect) | -0.281 | -0.692 | 0.117 | 1.000 |
|  |  | Indirect effect | 0.046 | -0.416 | 0.514 | 1.001 |
|  |  | Total effect | -0.234 | -0.466 | -0.004 | 1.001 |
|  |  | Indirect effect (%) | 8.644 | -739.909 | 251.929 | 1.000 |
| 45 | Miyazaki | a | -0.032 | -0.046 | -0.018 | 1.000 |
|  |  | b | -17.434 | -21.879 | -13.599 | 1.023 |
|  |  | c (Direct effect) | -0.929 | -1.209 | -0.662 | 1.004 |
|  |  | Indirect effect | 0.560 | 0.294 | 0.845 | 1.004 |
|  |  | Total effect | -0.369 | -0.573 | -0.165 | 1.001 |
|  |  | Indirect effect (%) | -176.406 | -424.236 | -61.159 | 1.000 |
| 46 | Kagoshima | a | -0.029 | -0.037 | -0.022 | 1.009 |
|  |  | b | 106.374 | -1275.631 | 845.549 | 3.372 |
|  |  | c (Direct effect) | 2.692 | -37.819 | 25.103 | 3.164 |
|  |  | Indirect effect | -3.194 | -25.606 | 37.306 | 3.164 |
|  |  | Total effect | -0.502 | -0.681 | -0.322 | 1.005 |
|  |  | Indirect effect (%) | 691.559 | -7801.399 | 6184.785 | 2.745 |
| 47 | Okinawa | a | -0.014 | -0.054 | 0.024 | 1.002 |
|  |  | b | -5.537 | -7.196 | -4.236 | 1.002 |
|  |  | c (Direct effect) | -0.937 | -1.138 | -0.759 | 1.001 |
|  |  | Indirect effect | 0.085 | -0.123 | 0.331 | 1.003 |
|  |  | Total effect | -0.853 | -1.006 | -0.689 | 1.002 |
|  |  | Indirect effect (%) | -10.723 | -43.537 | 13.118 | 1.004 |

Prefectures where NPIs were not implemented are represented by "-".

**Table S4. Effects of NPIs on mitigating the COVID-19 infection mediated by nighttime human mobility in downtown areas during Phase 1**

| **Prefecture Number** | **Prefecture Name** | **Parameters** | **Mean** | **95% Credible Interval** | | **Rhat** |
| --- | --- | --- | --- | --- | --- | --- |
|  |  |  |  | **Low** | **High** |  |
| 1 | Hokkaido | a | -0.158 | -0.215 | -0.102 | 1.001 |
|  |  | b | 0.705 | 0.475 | 0.939 | 1.004 |
|  |  | c (Direct effect) | 0.054 | -0.126 | 0.236 | 1.003 |
|  |  | Indirect effect | -0.112 | -0.170 | -0.063 | 1.004 |
|  |  | Total effect | -0.057 | -0.216 | 0.108 | 1.002 |
|  |  | Indirect effect (%) | 81.346 | -1669.332 | 1849.928 | 1.000 |
| 2 | Aomori | a | -0.039 | -0.057 | -0.021 | 1.003 |
|  |  | b | 5.075 | 3.051 | 7.193 | 1.002 |
|  |  | c (Direct effect) | 0.597 | 0.238 | 0.962 | 1.001 |
|  |  | Indirect effect | -0.196 | -0.344 | -0.091 | 1.002 |
|  |  | Total effect | 0.401 | 0.083 | 0.722 | 1.001 |
|  |  | Indirect effect (%) | -59.750 | -183.542 | -18.290 | 1.000 |
| 3 | Iwate | a | -0.039 | -0.057 | -0.021 | 1.001 |
|  |  | b | 1.816 | 0.724 | 2.948 | 1.002 |
|  |  | c (Direct effect) | 0.078 | -0.145 | 0.299 | 1.002 |
|  |  | Indirect effect | -0.070 | -0.134 | -0.023 | 1.002 |
|  |  | Total effect | 0.008 | -0.195 | 0.212 | 1.002 |
|  |  | Indirect effect (%) | -130.979 | -1070.917 | 1201.699 | 1.000 |
| 4 | Miyagi | a | -0.104 | -0.155 | -0.057 | 1.002 |
|  |  | b | 0.989 | 0.531 | 1.480 | 1.003 |
|  |  | c (Direct effect) | -0.381 | -0.630 | -0.128 | 1.001 |
|  |  | Indirect effect | -0.104 | -0.190 | -0.042 | 1.003 |
|  |  | Total effect | -0.484 | -0.707 | -0.260 | 1.001 |
|  |  | Indirect effect (%) | 23.384 | 7.399 | 54.477 | 1.002 |
| 5 | Akita | a | -0.042 | -0.056 | -0.027 | 1.000 |
|  |  | b | 10.870 | 7.522 | 14.299 | 1.001 |
|  |  | c (Direct effect) | 0.595 | 0.203 | 0.994 | 1.001 |
|  |  | Indirect effect | -0.453 | -0.707 | -0.252 | 1.001 |
|  |  | Total effect | 0.142 | -0.180 | 0.475 | 1.000 |
|  |  | Indirect effect (%) | -109.951 | -3172.301 | 2927.048 | 1.000 |
| 6 | Yamagata | a | -0.030 | -0.041 | -0.019 | 1.001 |
|  |  | b | 4.894 | 3.260 | 6.517 | 1.005 |
|  |  | c (Direct effect) | -0.287 | -0.562 | -0.026 | 1.007 |
|  |  | Indirect effect | -0.146 | -0.227 | -0.079 | 1.004 |
|  |  | Total effect | -0.434 | -0.688 | -0.189 | 1.006 |
|  |  | Indirect effect (%) | 38.473 | 14.885 | 86.818 | 1.001 |
| 7 | Fukushima | a | -0.036 | -0.049 | -0.022 | 1.001 |
|  |  | b | 4.195 | 3.022 | 5.430 | 1.003 |
|  |  | c (Direct effect) | -0.171 | -0.406 | 0.052 | 1.008 |
|  |  | Indirect effect | -0.150 | -0.230 | -0.084 | 1.002 |
|  |  | Total effect | -0.322 | -0.547 | -0.104 | 1.010 |
|  |  | Indirect effect (%) | 44.656 | 20.991 | 140.628 | 1.000 |
| 8 | Ibaraki | a | -0.044 | -0.062 | -0.025 | 1.001 |
|  |  | b | 2.989 | 2.251 | 3.757 | 1.001 |
|  |  | c (Direct effect) | -0.361 | -0.530 | -0.190 | 1.001 |
|  |  | Indirect effect | -0.131 | -0.201 | -0.070 | 1.001 |
|  |  | Total effect | -0.491 | -0.652 | -0.327 | 1.000 |
|  |  | Indirect effect (%) | 27.318 | 13.810 | 46.307 | 1.001 |
| 9 | Tochigi | a | -0.041 | -0.052 | -0.031 | 1.009 |
|  |  | b | 2.605 | 1.243 | 3.967 | 1.066 |
|  |  | c (Direct effect) | -0.040 | -0.269 | 0.189 | 1.025 |
|  |  | Indirect effect | -0.107 | -0.178 | -0.049 | 1.051 |
|  |  | Total effect | -0.147 | -0.338 | 0.048 | 1.013 |
|  |  | Indirect effect (%) | 66.765 | -597.174 | 776.022 | 1.000 |
| 10 | Gunma | a | -0.062 | -0.085 | -0.038 | 1.007 |
|  |  | b | 1.417 | 0.707 | 2.167 | 1.007 |
|  |  | c (Direct effect) | -0.110 | -0.376 | 0.155 | 1.004 |
|  |  | Indirect effect | -0.087 | -0.147 | -0.038 | 1.008 |
|  |  | Total effect | -0.197 | -0.442 | 0.046 | 1.003 |
|  |  | Indirect effect (%) | 25.568 | -321.595 | 414.977 | 1.000 |
| 11 | Saitama | a | -0.150 | -0.189 | -0.115 | 1.004 |
|  |  | b | 0.992 | 0.629 | 1.369 | 1.002 |
|  |  | c (Direct effect) | -0.142 | -0.295 | 0.015 | 1.000 |
|  |  | Indirect effect | -0.150 | -0.234 | -0.087 | 1.003 |
|  |  | Total effect | -0.292 | -0.409 | -0.174 | 1.000 |
|  |  | Indirect effect (%) | 54.645 | 24.347 | 107.878 | 1.001 |
| 12 | Chiba | a | -0.212 | -0.253 | -0.173 | 1.001 |
|  |  | b | 1.128 | 0.822 | 1.448 | 1.001 |
|  |  | c (Direct effect) | -0.039 | -0.202 | 0.129 | 1.001 |
|  |  | Indirect effect | -0.240 | -0.333 | -0.163 | 1.001 |
|  |  | Total effect | -0.279 | -0.401 | -0.154 | 1.000 |
|  |  | Indirect effect (%) | 92.585 | 46.698 | 177.340 | 1.000 |
| 13 | Tokyo | a | -0.067 | -0.114 | -0.033 | 1.027 |
|  |  | b | 0.404 | 0.113 | 0.772 | 1.025 |
|  |  | c (Direct effect) | -0.442 | -0.547 | -0.333 | 1.030 |
|  |  | Indirect effect | -0.028 | -0.073 | -0.006 | 1.027 |
|  |  | Total effect | -0.470 | -0.561 | -0.378 | 1.019 |
|  |  | Indirect effect (%) | 6.217 | 1.130 | 16.690 | 1.027 |
| 14 | Kanagawa | a | -0.116 | -0.164 | -0.075 | 1.004 |
|  |  | b | 1.639 | 1.113 | 2.280 | 1.005 |
|  |  | c (Direct effect) | 0.056 | -0.102 | 0.243 | 1.003 |
|  |  | Indirect effect | -0.194 | -0.351 | -0.095 | 1.005 |
|  |  | Total effect | -0.137 | -0.244 | -0.029 | 1.001 |
|  |  | Indirect effect (%) | 70.342 | 49.083 | 586.408 | 1.000 |
| 15 | Niigata | a | -0.154 | -0.207 | -0.100 | 1.002 |
|  |  | b | 0.795 | 0.439 | 1.150 | 1.003 |
|  |  | c (Direct effect) | 0.122 | -0.168 | 0.410 | 1.001 |
|  |  | Indirect effect | -0.122 | -0.199 | -0.059 | 1.003 |
|  |  | Total effect | 0.000 | -0.257 | 0.255 | 1.001 |
|  |  | Indirect effect (%) | 1045.823 | -1538.447 | 1550.739 | 1.000 |
| 16 | Toyama | a | -0.055 | -0.087 | -0.021 | 1.009 |
|  |  | b | 3.286 | 2.658 | 3.937 | 1.004 |
|  |  | c (Direct effect) | 0.051 | -0.214 | 0.319 | 1.001 |
|  |  | Indirect effect | -0.180 | -0.299 | -0.068 | 1.006 |
|  |  | Total effect | -0.129 | -0.400 | 0.150 | 1.000 |
|  |  | Indirect effect (%) | 72.891 | -1016.108 | 1147.248 | 1.000 |
| 17 | Ishikawa | a | -0.027 | -0.045 | -0.009 | 1.001 |
|  |  | b | 4.892 | 3.610 | 6.211 | 1.001 |
|  |  | c (Direct effect) | -0.148 | -0.397 | 0.102 | 1.000 |
|  |  | Indirect effect | -0.133 | -0.238 | -0.042 | 1.001 |
|  |  | Total effect | -0.282 | -0.525 | -0.029 | 1.000 |
|  |  | Indirect effect (%) | 55.278 | 11.080 | 217.838 | 1.000 |
| 18 | Fukui | a | -0.064 | -0.100 | -0.027 | 1.007 |
|  |  | b | 1.164 | 0.449 | 1.878 | 1.013 |
|  |  | c (Direct effect) | -0.282 | -0.636 | 0.074 | 1.009 |
|  |  | Indirect effect | -0.075 | -0.147 | -0.019 | 1.014 |
|  |  | Total effect | -0.357 | -0.680 | -0.026 | 1.007 |
|  |  | Indirect effect (%) | 55.827 | 1.871 | 145.598 | 1.000 |
| 19 | Yamanashi | a | -0.041 | -0.063 | -0.019 | 1.001 |
|  |  | b | 1.954 | 0.901 | 2.991 | 1.002 |
|  |  | c (Direct effect) | -0.055 | -0.334 | 0.226 | 1.001 |
|  |  | Indirect effect | -0.081 | -0.151 | -0.028 | 1.001 |
|  |  | Total effect | -0.135 | -0.396 | 0.131 | 1.001 |
|  |  | Indirect effect (%) | -391.935 | -533.530 | 638.432 | 1.000 |
| 20 | Nagano | a | -0.058 | -0.075 | -0.041 | 1.004 |
|  |  | b | 3.888 | 2.348 | 5.014 | 1.078 |
|  |  | c (Direct effect) | 0.086 | -0.300 | 0.340 | 1.044 |
|  |  | Indirect effect | -0.227 | -0.331 | -0.134 | 1.033 |
|  |  | Total effect | -0.140 | -0.443 | 0.085 | 1.052 |
|  |  | Indirect effect (%) | 192.762 | -1301.018 | 1658.627 | 1.000 |
| 21 | Gifu | a | -0.034 | -0.052 | -0.019 | 1.014 |
|  |  | b | 2.936 | 2.002 | 3.876 | 1.005 |
|  |  | c (Direct effect) | -0.138 | -0.311 | 0.031 | 1.002 |
|  |  | Indirect effect | -0.099 | -0.174 | -0.050 | 1.010 |
|  |  | Total effect | -0.238 | -0.391 | -0.082 | 1.002 |
|  |  | Indirect effect (%) | 50.610 | 16.457 | 131.035 | 1.000 |
| 22 | Shizuoka | a | -0.174 | -0.227 | -0.120 | 1.000 |
|  |  | b | 1.877 | 1.495 | 2.277 | 1.002 |
|  |  | c (Direct effect) | 0.207 | -0.065 | 0.482 | 1.000 |
|  |  | Indirect effect | -0.326 | -0.456 | -0.212 | 1.001 |
|  |  | Total effect | -0.119 | -0.372 | 0.141 | 1.000 |
|  |  | Indirect effect (%) | 114.324 | -2499.074 | 2899.386 | 1.000 |
| 23 | Aichi | a | -0.131 | -0.176 | -0.085 | 1.001 |
|  |  | b | 1.330 | 1.026 | 1.630 | 1.001 |
|  |  | c (Direct effect) | 0.091 | -0.078 | 0.264 | 1.001 |
|  |  | Indirect effect | -0.174 | -0.251 | -0.106 | 1.002 |
|  |  | Total effect | -0.083 | -0.245 | 0.084 | 1.000 |
|  |  | Indirect effect (%) | -240.928 | -1808.187 | 1981.999 | 1.000 |
| 24 | Mie | a | -0.048 | -0.066 | -0.030 | 1.001 |
|  |  | b | 5.086 | 3.410 | 6.817 | 1.001 |
|  |  | c (Direct effect) | -0.023 | -0.300 | 0.255 | 1.002 |
|  |  | Indirect effect | -0.244 | -0.394 | -0.124 | 1.001 |
|  |  | Total effect | -0.266 | -0.500 | -0.028 | 1.002 |
|  |  | Indirect effect (%) | 116.533 | 28.372 | 473.131 | 1.000 |
| 25 | Shiga | a | -0.034 | -0.045 | -0.022 | 1.001 |
|  |  | b | 15.157 | 8.828 | 23.222 | 1.002 |
|  |  | c (Direct effect) | 0.258 | -0.146 | 0.750 | 1.002 |
|  |  | Indirect effect | -0.520 | -0.966 | -0.227 | 1.002 |
|  |  | Total effect | -0.262 | -0.495 | -0.020 | 1.001 |
|  |  | Indirect effect (%) | 147.088 | 48.160 | 1056.621 | 1.000 |
| 26 | Kyoto | a | -0.100 | -0.138 | -0.062 | 1.000 |
|  |  | b | 0.690 | 0.397 | 0.979 | 1.001 |
|  |  | c (Direct effect) | -0.439 | -0.576 | -0.300 | 1.000 |
|  |  | Indirect effect | -0.069 | -0.113 | -0.033 | 1.000 |
|  |  | Total effect | -0.508 | -0.631 | -0.383 | 1.000 |
|  |  | Indirect effect (%) | 13.931 | 5.905 | 24.749 | 1.000 |
| 27 | Osaka | a | -0.144 | -0.183 | -0.106 | 1.002 |
|  |  | b | 1.668 | 1.077 | 2.245 | 1.006 |
|  |  | c (Direct effect) | 0.008 | -0.158 | 0.181 | 1.003 |
|  |  | Indirect effect | -0.243 | -0.375 | -0.131 | 1.005 |
|  |  | Total effect | -0.234 | -0.354 | -0.113 | 1.000 |
|  |  | Indirect effect (%) | 113.495 | 48.957 | 235.081 | 1.001 |
| 28 | Hyogo | a | -0.258 | -0.316 | -0.205 | 1.002 |
|  |  | b | 1.595 | 1.167 | 2.044 | 1.002 |
|  |  | c (Direct effect) | 0.307 | 0.086 | 0.542 | 1.001 |
|  |  | Indirect effect | -0.414 | -0.608 | -0.261 | 1.002 |
|  |  | Total effect | -0.107 | -0.243 | 0.029 | 1.000 |
|  |  | Indirect effect (%) | 600.329 | -2300.266 | 3345.118 | 1.000 |
| 29 | Nara | a | -0.021 | -0.030 | -0.012 | 1.006 |
|  |  | b | 10.630 | 7.898 | 13.564 | 1.005 |
|  |  | c (Direct effect) | -0.111 | -0.341 | 0.124 | 1.001 |
|  |  | Indirect effect | -0.224 | -0.369 | -0.117 | 1.008 |
|  |  | Total effect | -0.335 | -0.536 | -0.130 | 1.000 |
|  |  | Indirect effect (%) | 75.979 | 30.019 | 181.040 | 1.000 |
| 30 | Wakayama | a | -0.038 | -0.052 | -0.025 | 1.003 |
|  |  | b | 8.745 | 6.394 | 11.181 | 1.006 |
|  |  | c (Direct effect) | 0.701 | 0.373 | 1.025 | 1.003 |
|  |  | Indirect effect | -0.333 | -0.506 | -0.196 | 1.005 |
|  |  | Total effect | 0.368 | 0.081 | 0.649 | 1.002 |
|  |  | Indirect effect (%) | -113.503 | -355.239 | -38.631 | 1.000 |
| 31 | Tottori | a | -0.055 | -0.074 | -0.037 | 1.001 |
|  |  | b | 3.567 | 2.175 | 4.963 | 1.004 |
|  |  | c (Direct effect) | 0.307 | -0.006 | 0.616 | 1.001 |
|  |  | Indirect effect | -0.198 | -0.312 | -0.105 | 1.002 |
|  |  | Total effect | 0.109 | -0.166 | 0.389 | 1.000 |
|  |  | Indirect effect (%) | 518.767 | -1669.353 | 1591.552 | 1.000 |
| 32 | Shimane | a | -0.069 | -0.090 | -0.049 | 1.001 |
|  |  | b | 2.312 | 0.830 | 3.778 | 1.002 |
|  |  | c (Direct effect) | -0.086 | -0.492 | 0.321 | 1.000 |
|  |  | Indirect effect | -0.160 | -0.281 | -0.055 | 1.003 |
|  |  | Total effect | -0.246 | -0.602 | 0.111 | 1.000 |
|  |  | Indirect effect (%) | 93.306 | -513.386 | 702.661 | 1.000 |
| 33 | Okayama | a | -0.191 | -0.257 | -0.125 | 1.001 |
|  |  | b | 1.193 | 0.950 | 1.435 | 1.001 |
|  |  | c (Direct effect) | 0.665 | 0.430 | 0.900 | 1.002 |
|  |  | Indirect effect | -0.227 | -0.325 | -0.141 | 1.000 |
|  |  | Total effect | 0.437 | 0.217 | 0.659 | 1.002 |
|  |  | Indirect effect (%) | -56.902 | -112.066 | -27.061 | 1.000 |
| 34 | Hiroshima | a | -0.111 | -0.147 | -0.074 | 1.012 |
|  |  | b | 1.274 | 0.905 | 1.718 | 1.067 |
|  |  | c (Direct effect) | -0.511 | -0.784 | -0.231 | 1.058 |
|  |  | Indirect effect | -0.142 | -0.217 | -0.084 | 1.023 |
|  |  | Total effect | -0.652 | -0.908 | -0.391 | 1.048 |
|  |  | Indirect effect (%) | 22.953 | 11.230 | 43.342 | 1.048 |
| 35 | Yamaguchi | a | -0.035 | -0.047 | -0.023 | 1.007 |
|  |  | b | 5.657 | 2.912 | 8.511 | 1.006 |
|  |  | c (Direct effect) | 0.287 | -0.087 | 0.673 | 1.004 |
|  |  | Indirect effect | -0.196 | -0.333 | -0.092 | 1.009 |
|  |  | Total effect | 0.091 | -0.222 | 0.413 | 1.002 |
|  |  | Indirect effect (%) | -67.253 | -1725.710 | 1688.604 | 1.000 |
| 36 | Tokushima | a | -0.028 | -0.041 | -0.016 | 1.001 |
|  |  | b | 4.732 | 1.229 | 8.344 | 1.003 |
|  |  | c (Direct effect) | 0.202 | -0.134 | 0.545 | 1.000 |
|  |  | Indirect effect | -0.135 | -0.275 | -0.030 | 1.003 |
|  |  | Total effect | 0.067 | -0.221 | 0.357 | 1.000 |
|  |  | Indirect effect (%) | -122.421 | -1251.741 | 1264.462 | 1.000 |
| 37 | Kagawa | a | -0.123 | -0.159 | -0.088 | 1.000 |
|  |  | b | 0.578 | -0.280 | 1.434 | 1.001 |
|  |  | c (Direct effect) | -0.476 | -0.822 | -0.134 | 1.002 |
|  |  | Indirect effect | -0.072 | -0.189 | 0.034 | 1.001 |
|  |  | Total effect | -0.548 | -0.830 | -0.270 | 1.001 |
|  |  | Indirect effect (%) | 15.984 | -5.057 | 54.003 | 1.000 |
| 38 | Ehime | a | -0.115 | -0.153 | -0.077 | 1.013 |
|  |  | b | 1.519 | 0.982 | 2.092 | 1.023 |
|  |  | c (Direct effect) | 0.788 | 0.457 | 1.118 | 1.008 |
|  |  | Indirect effect | -0.174 | -0.277 | -0.096 | 1.022 |
|  |  | Total effect | 0.614 | 0.316 | 0.914 | 1.004 |
|  |  | Indirect effect (%) | -30.067 | -55.822 | -14.755 | 1.005 |
| 39 | Kochi | a | -0.030 | -0.043 | -0.016 | 1.001 |
|  |  | b | 7.879 | 5.866 | 10.224 | 1.017 |
|  |  | c (Direct effect) | 0.216 | -0.073 | 0.504 | 1.000 |
|  |  | Indirect effect | -0.234 | -0.371 | -0.116 | 1.006 |
|  |  | Total effect | -0.018 | -0.307 | 0.270 | 1.001 |
|  |  | Indirect effect (%) | 156.927 | -2357.001 | 2543.141 | 1.000 |
| 40 | Fukuoka | a | -0.135 | -0.173 | -0.098 | 1.001 |
|  |  | b | 0.989 | 0.457 | 1.516 | 1.001 |
|  |  | c (Direct effect) | -0.607 | -0.853 | -0.362 | 1.000 |
|  |  | Indirect effect | -0.134 | -0.222 | -0.059 | 1.001 |
|  |  | Total effect | -0.741 | -0.954 | -0.530 | 1.001 |
|  |  | Indirect effect (%) | 18.716 | 7.045 | 34.846 | 1.000 |
| 41 | Saga | a | -0.046 | -0.067 | -0.025 | 1.001 |
|  |  | b | 5.417 | 4.277 | 6.591 | 1.001 |
|  |  | c (Direct effect) | 0.796 | 0.513 | 1.080 | 1.000 |
|  |  | Indirect effect | -0.250 | -0.383 | -0.132 | 1.001 |
|  |  | Total effect | 0.545 | 0.272 | 0.829 | 1.000 |
|  |  | Indirect effect (%) | -50.724 | -108.600 | -19.863 | 1.000 |
| 42 | Nagasaki | a | -0.146 | -0.204 | -0.093 | 1.008 |
|  |  | b | 1.322 | 0.976 | 1.650 | 1.005 |
|  |  | c (Direct effect) | 0.221 | -0.022 | 0.466 | 1.001 |
|  |  | Indirect effect | -0.193 | -0.290 | -0.111 | 1.012 |
|  |  | Total effect | 0.028 | -0.200 | 0.260 | 1.000 |
|  |  | Indirect effect (%) | 10434.854 | -2426.973 | 2475.425 | 1.000 |
| 43 | Kumamoto | a | -0.205 | -0.284 | -0.138 | 1.047 |
|  |  | b | 1.102 | 0.868 | 1.322 | 1.011 |
|  |  | c (Direct effect) | 0.381 | 0.120 | 0.641 | 1.012 |
|  |  | Indirect effect | -0.226 | -0.331 | -0.143 | 1.021 |
|  |  | Total effect | 0.155 | -0.097 | 0.401 | 1.027 |
|  |  | Indirect effect (%) | -62.073 | -1480.797 | 1210.634 | 1.000 |
| 44 | Oita | a | -0.172 | -0.224 | -0.119 | 1.005 |
|  |  | b | 1.361 | 0.864 | 1.892 | 1.022 |
|  |  | c (Direct effect) | 0.128 | -0.155 | 0.417 | 1.008 |
|  |  | Indirect effect | -0.234 | -0.367 | -0.127 | 1.019 |
|  |  | Total effect | -0.107 | -0.336 | 0.132 | 1.002 |
|  |  | Indirect effect (%) | 741.209 | -2175.947 | 2224.422 | 1.000 |
| 45 | Miyazaki | a | -0.044 | -0.060 | -0.029 | 1.001 |
|  |  | b | 7.801 | 6.204 | 9.361 | 1.001 |
|  |  | c (Direct effect) | 0.587 | 0.274 | 0.893 | 1.003 |
|  |  | Indirect effect | -0.347 | -0.496 | -0.212 | 1.002 |
|  |  | Total effect | 0.240 | -0.066 | 0.547 | 1.002 |
|  |  | Indirect effect (%) | -106.261 | -1310.407 | 1014.814 | 1.000 |
| 46 | Kagoshima | a | -0.041 | -0.053 | -0.028 | 1.000 |
|  |  | b | 9.779 | 4.775 | 14.923 | 1.004 |
|  |  | c (Direct effect) | 0.295 | -0.127 | 0.720 | 1.002 |
|  |  | Indirect effect | -0.399 | -0.670 | -0.179 | 1.003 |
|  |  | Total effect | -0.104 | -0.447 | 0.238 | 1.000 |
|  |  | Indirect effect (%) | -292.675 | -2971.541 | 3096.209 | 1.000 |
| 47 | Okinawa | a | -0.105 | -0.146 | -0.065 | 1.002 |
|  |  | b | 1.965 | 1.552 | 2.385 | 1.001 |
|  |  | c (Direct effect) | -0.298 | -0.529 | -0.065 | 1.000 |
|  |  | Indirect effect | -0.207 | -0.305 | -0.122 | 1.002 |
|  |  | Total effect | -0.505 | -0.725 | -0.278 | 1.000 |
|  |  | Indirect effect (%) | 43.267 | 22.281 | 78.724 | 1.001 |

**Table S5. Effects of NPIs on mitigating the COVID-19 infection mediated by nighttime human mobility in downtown areas during Phase 2**

| **Prefecture Number** | **Prefecture Name** | **Parameters** | **Mean** | **95% Credible Interval** | | **Rhat** |
| --- | --- | --- | --- | --- | --- | --- |
|  |  |  |  | **Low** | **High** |  |
| 1 | Hokkaido | a | -0.096 | -0.120 | -0.072 | 1.003 |
|  |  | b | 0.368 | -0.065 | 0.804 | 1.002 |
|  |  | c (Direct effect) | -0.076 | -0.182 | 0.029 | 1.000 |
|  |  | Indirect effect | -0.035 | -0.079 | 0.006 | 1.002 |
|  |  | Total effect | -0.112 | -0.206 | -0.015 | 1.001 |
|  |  | Indirect effect (%) | 31.245 | -10.855 | 177.964 | 1.000 |
| 2 | Aomori | a | - | - | - | - |
|  |  | b | - | - | - | - |
|  |  | c (Direct effect) | - | - | - | - |
|  |  | Indirect effect | - | - | - | - |
|  |  | Total effect | - | - | - | - |
|  |  | Indirect effect (%) | - | - | - | - |
| 3 | Iwate | a | - | - | - | - |
|  |  | b | - | - | - | - |
|  |  | c (Direct effect) | - | - | - | - |
|  |  | Indirect effect | - | - | - | - |
|  |  | Total effect | - | - | - | - |
|  |  | Indirect effect (%) | - | - | - | - |
| 4 | Miyagi | a | -0.075 | -0.093 | -0.058 | 1.003 |
|  |  | b | 1.315 | 0.602 | 2.001 | 1.001 |
|  |  | c (Direct effect) | -0.021 | -0.168 | 0.124 | 1.001 |
|  |  | Indirect effect | -0.099 | -0.159 | -0.043 | 1.000 |
|  |  | Total effect | -0.120 | -0.241 | 0.001 | 1.002 |
|  |  | Indirect effect (%) | 127.997 | -134.446 | 585.694 | 1.000 |
| 5 | Akita | a | - | - | - | - |
|  |  | b | - | - | - | - |
|  |  | c (Direct effect) | - | - | - | - |
|  |  | Indirect effect | - | - | - | - |
|  |  | Total effect | - | - | - | - |
|  |  | Indirect effect (%) | - | - | - | - |
| 6 | Yamagata | a | - | - | - | - |
|  |  | b | - | - | - | - |
|  |  | c (Direct effect) | - | - | - | - |
|  |  | Indirect effect | - | - | - | - |
|  |  | Total effect | - | - | - | - |
|  |  | Indirect effect (%) | - | - | - | - |
| 7 | Fukushima | a | -0.034 | -0.045 | -0.024 | 1.001 |
|  |  | b | 3.920 | 1.775 | 6.134 | 1.002 |
|  |  | c (Direct effect) | -0.075 | -0.295 | 0.148 | 1.001 |
|  |  | Indirect effect | -0.134 | -0.231 | -0.055 | 1.002 |
|  |  | Total effect | -0.209 | -0.390 | -0.029 | 1.001 |
|  |  | Indirect effect (%) | 71.545 | 14.996 | 364.773 | 1.000 |
| 8 | Ibaraki | a | -0.087 | -0.101 | -0.072 | 1.001 |
|  |  | b | 1.738 | 0.269 | 3.275 | 1.001 |
|  |  | c (Direct effect) | 0.022 | -0.211 | 0.264 | 1.001 |
|  |  | Indirect effect | -0.151 | -0.293 | -0.023 | 1.001 |
|  |  | Total effect | -0.129 | -0.278 | 0.023 | 1.000 |
|  |  | Indirect effect (%) | 160.035 | -746.394 | 1096.053 | 1.000 |
| 9 | Tochigi | a | -0.074 | -0.088 | -0.061 | 1.003 |
|  |  | b | 6.116 | 3.464 | 8.988 | 1.006 |
|  |  | c (Direct effect) | 0.573 | 0.225 | 0.943 | 1.006 |
|  |  | Indirect effect | -0.457 | -0.737 | -0.235 | 1.006 |
|  |  | Total effect | 0.116 | -0.058 | 0.296 | 1.003 |
|  |  | Indirect effect (%) | -266.805 | -3313.329 | 2422.849 | 1.000 |
| 10 | Gunma | a | -0.068 | -0.088 | -0.051 | 1.005 |
|  |  | b | 2.697 | 1.724 | 3.771 | 1.007 |
|  |  | c (Direct effect) | 0.013 | -0.153 | 0.184 | 1.014 |
|  |  | Indirect effect | -0.186 | -0.303 | -0.103 | 1.005 |
|  |  | Total effect | -0.173 | -0.291 | -0.064 | 1.022 |
|  |  | Indirect effect (%) | 158.890 | 42.610 | 340.491 | 1.000 |
| 11 | Saitama | a | -0.069 | -0.089 | -0.050 | 1.006 |
|  |  | b | 0.609 | 0.006 | 1.293 | 1.006 |
|  |  | c (Direct effect) | 0.034 | -0.059 | 0.131 | 1.002 |
|  |  | Indirect effect | -0.043 | -0.100 | 0.000 | 1.006 |
|  |  | Total effect | -0.009 | -0.081 | 0.065 | 1.000 |
|  |  | Indirect effect (%) | -5.004 | -1836.261 | 1826.087 | 1.000 |
| 12 | Chiba | a | -0.057 | -0.075 | -0.039 | 1.015 |
|  |  | b | 0.441 | -0.066 | 0.907 | 1.022 |
|  |  | c (Direct effect) | -0.004 | -0.091 | 0.082 | 1.002 |
|  |  | Indirect effect | -0.025 | -0.056 | 0.004 | 1.023 |
|  |  | Total effect | -0.029 | -0.107 | 0.048 | 1.000 |
|  |  | Indirect effect (%) | 129.541 | -868.426 | 880.784 | 1.000 |
| 13 | Tokyo | a | -0.052 | -0.072 | -0.031 | 1.006 |
|  |  | b | -0.364 | -0.671 | -0.031 | 1.008 |
|  |  | c (Direct effect) | -0.016 | -0.069 | 0.035 | 1.001 |
|  |  | Indirect effect | 0.019 | 0.002 | 0.038 | 1.005 |
|  |  | Total effect | 0.002 | -0.049 | 0.051 | 1.002 |
|  |  | Indirect effect (%) | 87.756 | -1120.477 | 1096.157 | 1.000 |
| 14 | Kanagawa | a | -0.042 | -0.057 | -0.030 | 1.006 |
|  |  | b | 0.315 | -0.340 | 1.025 | 1.005 |
|  |  | c (Direct effect) | -0.093 | -0.172 | -0.013 | 1.002 |
|  |  | Indirect effect | -0.013 | -0.046 | 0.014 | 1.004 |
|  |  | Total effect | -0.106 | -0.179 | -0.034 | 1.004 |
|  |  | Indirect effect (%) | 27.553 | -15.198 | 68.424 | 1.000 |
| 15 | Niigata | a | - | - | - | - |
|  |  | b | - | - | - | - |
|  |  | c (Direct effect) | - | - | - | - |
|  |  | Indirect effect | - | - | - | - |
|  |  | Total effect | - | - | - | - |
|  |  | Indirect effect (%) | - | - | - | - |
| 16 | Toyama | a | -0.105 | -0.126 | -0.084 | 1.001 |
|  |  | b | 2.295 | 1.447 | 3.174 | 1.002 |
|  |  | c (Direct effect) | 0.039 | -0.244 | 0.324 | 1.001 |
|  |  | Indirect effect | -0.241 | -0.352 | -0.145 | 1.002 |
|  |  | Total effect | -0.203 | -0.442 | 0.035 | 1.001 |
|  |  | Indirect effect (%) | 35.119 | -660.833 | 1014.365 | 1.000 |
| 17 | Ishikawa | a | -0.043 | -0.055 | -0.032 | 1.001 |
|  |  | b | 5.315 | 3.188 | 7.544 | 1.004 |
|  |  | c (Direct effect) | 0.121 | -0.094 | 0.343 | 1.002 |
|  |  | Indirect effect | -0.231 | -0.366 | -0.127 | 1.004 |
|  |  | Total effect | -0.110 | -0.272 | 0.054 | 1.000 |
|  |  | Indirect effect (%) | 74.415 | -1659.169 | 2173.067 | 1.000 |
| 18 | Fukui | a | - | - | - | - |
|  |  | b | - | - | - | - |
|  |  | c (Direct effect) | - | - | - | - |
|  |  | Indirect effect | - | - | - | - |
|  |  | Total effect | - | - | - | - |
|  |  | Indirect effect (%) | - | - | - | - |
| 19 | Yamanashi | a | -0.165 | -0.182 | -0.147 | 1.001 |
|  |  | b | 6.558 | 3.339 | 9.975 | 1.001 |
|  |  | c (Direct effect) | 1.041 | 0.385 | 1.733 | 1.001 |
|  |  | Indirect effect | -1.082 | -1.697 | -0.537 | 1.001 |
|  |  | Total effect | -0.041 | -0.284 | 0.204 | 1.000 |
|  |  | Indirect effect (%) | -243.871 | -12938.773 | 13414.548 | 1.000 |
| 20 | Nagano | a | - | - | - | - |
|  |  | b | - | - | - | - |
|  |  | c (Direct effect) | - | - | - | - |
|  |  | Indirect effect | - | - | - | - |
|  |  | Total effect | - | - | - | - |
|  |  | Indirect effect (%) | - | - | - | - |
| 21 | Gifu | a | -0.036 | -0.046 | -0.027 | 1.001 |
|  |  | b | 3.810 | 2.632 | 5.008 | 1.000 |
|  |  | c (Direct effect) | -0.004 | -0.138 | 0.130 | 1.000 |
|  |  | Indirect effect | -0.137 | -0.199 | -0.085 | 1.001 |
|  |  | Total effect | -0.141 | -0.254 | -0.027 | 1.000 |
|  |  | Indirect effect (%) | 141.191 | 39.465 | 423.340 | 1.000 |
| 22 | Shizuoka | a | -0.330 | -0.399 | -0.258 | 1.001 |
|  |  | b | 2.854 | 1.695 | 4.036 | 1.002 |
|  |  | c (Direct effect) | 1.168 | 0.589 | 1.774 | 1.002 |
|  |  | Indirect effect | -0.958 | -1.559 | -0.463 | 1.002 |
|  |  | Total effect | 0.209 | 0.040 | 0.381 | 1.000 |
|  |  | Indirect effect (%) | -563.020 | -1944.325 | -182.280 | 1.000 |
| 23 | Aichi | a | -0.074 | -0.092 | -0.057 | 1.003 |
|  |  | b | 0.049 | -0.406 | 0.556 | 1.005 |
|  |  | c (Direct effect) | -0.012 | -0.084 | 0.060 | 1.001 |
|  |  | Indirect effect | -0.004 | -0.043 | 0.031 | 1.004 |
|  |  | Total effect | -0.016 | -0.080 | 0.049 | 1.001 |
|  |  | Indirect effect (%) | -5.145 | -645.702 | 689.154 | 1.000 |
| 24 | Mie | a | -0.027 | -0.035 | -0.019 | 1.002 |
|  |  | b | 3.136 | 1.581 | 4.716 | 1.000 |
|  |  | c (Direct effect) | -0.183 | -0.312 | -0.051 | 1.000 |
|  |  | Indirect effect | -0.086 | -0.139 | -0.041 | 1.000 |
|  |  | Total effect | -0.269 | -0.374 | -0.161 | 1.000 |
|  |  | Indirect effect (%) | 34.045 | 12.595 | 69.945 | 1.000 |
| 25 | Shiga | a | -0.053 | -0.056 | -0.050 | 1.014 |
|  |  | b | -307.568 | -2134.394 | 539.046 | 1.428 |
|  |  | c (Direct effect) | -16.469 | -113.752 | 28.471 | 1.427 |
|  |  | Indirect effect | 16.317 | -28.585 | 113.596 | 1.427 |
|  |  | Total effect | -0.152 | -0.330 | 0.025 | 1.024 |
|  |  | Indirect effect (%) | -26309.283 | -145522.063 | 67265.421 | 1.000 |
| 26 | Kyoto | a | -0.066 | -0.086 | -0.045 | 1.001 |
|  |  | b | 0.341 | -0.179 | 0.842 | 1.008 |
|  |  | c (Direct effect) | -0.249 | -0.358 | -0.136 | 1.004 |
|  |  | Indirect effect | -0.022 | -0.059 | 0.011 | 1.007 |
|  |  | Total effect | -0.271 | -0.367 | -0.173 | 1.002 |
|  |  | Indirect effect (%) | 8.995 | -3.888 | 26.552 | 1.006 |
| 27 | Osaka | a | -0.075 | -0.093 | -0.058 | 1.002 |
|  |  | b | 0.354 | -0.124 | 0.799 | 1.083 |
|  |  | c (Direct effect) | -0.087 | -0.154 | -0.012 | 1.015 |
|  |  | Indirect effect | -0.027 | -0.062 | 0.009 | 1.079 |
|  |  | Total effect | -0.113 | -0.173 | -0.044 | 1.055 |
|  |  | Indirect effect (%) | 26.921 | -9.991 | 75.897 | 1.000 |
| 28 | Hyogo | a | -0.092 | -0.112 | -0.070 | 1.012 |
|  |  | b | 0.512 | 0.137 | 0.904 | 1.003 |
|  |  | c (Direct effect) | -0.094 | -0.168 | -0.021 | 1.001 |
|  |  | Indirect effect | -0.047 | -0.085 | -0.012 | 1.004 |
|  |  | Total effect | -0.141 | -0.203 | -0.078 | 1.002 |
|  |  | Indirect effect (%) | 35.931 | 8.198 | 76.094 | 1.000 |
| 29 | Nara | a | - | - | - | - |
|  |  | b | - | - | - | - |
|  |  | c (Direct effect) | - | - | - | - |
|  |  | Indirect effect | - | - | - | - |
|  |  | Total effect | - | - | - | - |
|  |  | Indirect effect (%) | - | - | - | - |
| 30 | Wakayama | a | - | - | - | - |
|  |  | b | - | - | - | - |
|  |  | c (Direct effect) | - | - | - | - |
|  |  | Indirect effect | - | - | - | - |
|  |  | Total effect | - | - | - | - |
|  |  | Indirect effect (%) | - | - | - | - |
| 31 | Tottori | a | - | - | - | - |
|  |  | b | - | - | - | - |
|  |  | c (Direct effect) | - | - | - | - |
|  |  | Indirect effect | - | - | - | - |
|  |  | Total effect | - | - | - | - |
|  |  | Indirect effect (%) | - | - | - | - |
| 32 | Shimane | a | - | - | - | - |
|  |  | b | - | - | - | - |
|  |  | c (Direct effect) | - | - | - | - |
|  |  | Indirect effect | - | - | - | - |
|  |  | Total effect | - | - | - | - |
|  |  | Indirect effect (%) | - | - | - | - |
| 33 | Okayama | a | -0.259 | -0.303 | -0.213 | 1.002 |
|  |  | b | 0.755 | 0.460 | 1.046 | 1.002 |
|  |  | c (Direct effect) | -0.147 | -0.305 | 0.011 | 1.001 |
|  |  | Indirect effect | -0.196 | -0.284 | -0.116 | 1.002 |
|  |  | Total effect | -0.342 | -0.471 | -0.214 | 1.002 |
|  |  | Indirect effect (%) | 59.669 | 30.030 | 104.461 | 1.000 |
| 34 | Hiroshima | a | -0.070 | -0.092 | -0.048 | 1.003 |
|  |  | b | 1.489 | 0.863 | 2.127 | 1.003 |
|  |  | c (Direct effect) | -0.110 | -0.261 | 0.043 | 1.001 |
|  |  | Indirect effect | -0.104 | -0.165 | -0.054 | 1.001 |
|  |  | Total effect | -0.213 | -0.341 | -0.082 | 1.001 |
|  |  | Indirect effect (%) | 60.371 | 19.438 | 147.513 | 1.000 |
| 35 | Yamaguchi | a | - | - | - | - |
|  |  | b | - | - | - | - |
|  |  | c (Direct effect) | - | - | - | - |
|  |  | Indirect effect | - | - | - | - |
|  |  | Total effect | - | - | - | - |
|  |  | Indirect effect (%) | - | - | - | - |
| 36 | Tokushima | a | - | - | - | - |
|  |  | b | - | - | - | - |
|  |  | c (Direct effect) | - | - | - | - |
|  |  | Indirect effect | - | - | - | - |
|  |  | Total effect | - | - | - | - |
|  |  | Indirect effect (%) | - | - | - | - |
| 37 | Kagawa | a | -0.122 | -0.159 | -0.086 | 1.003 |
|  |  | b | 1.657 | 1.066 | 2.265 | 1.002 |
|  |  | c (Direct effect) | -0.037 | -0.273 | 0.198 | 1.001 |
|  |  | Indirect effect | -0.203 | -0.308 | -0.115 | 1.001 |
|  |  | Total effect | -0.239 | -0.446 | -0.033 | 1.000 |
|  |  | Indirect effect (%) | 109.663 | 29.929 | 425.031 | 1.000 |
| 38 | Ehime | a | -0.082 | -0.105 | -0.058 | 1.018 |
|  |  | b | 2.348 | 1.505 | 3.271 | 1.008 |
|  |  | c (Direct effect) | 0.126 | -0.130 | 0.391 | 1.004 |
|  |  | Indirect effect | -0.193 | -0.296 | -0.107 | 1.000 |
|  |  | Total effect | -0.067 | -0.285 | 0.159 | 1.006 |
|  |  | Indirect effect (%) | -1482.473 | -2470.531 | 2404.399 | 1.000 |
| 39 | Kochi | a | -0.036 | -0.050 | -0.023 | 1.000 |
|  |  | b | 3.814 | 2.070 | 5.571 | 1.000 |
|  |  | c (Direct effect) | 0.007 | -0.314 | 0.332 | 1.000 |
|  |  | Indirect effect | -0.138 | -0.231 | -0.065 | 1.000 |
|  |  | Total effect | -0.132 | -0.425 | 0.169 | 1.000 |
|  |  | Indirect effect (%) | -424.051 | -1065.225 | 1083.405 | 1.000 |
| 40 | Fukuoka | a | -0.088 | -0.105 | -0.072 | 1.013 |
|  |  | b | 0.880 | 0.381 | 1.383 | 1.004 |
|  |  | c (Direct effect) | -0.169 | -0.282 | -0.083 | 1.120 |
|  |  | Indirect effect | -0.078 | -0.129 | -0.033 | 1.006 |
|  |  | Total effect | -0.247 | -0.341 | -0.176 | 1.209 |
|  |  | Indirect effect (%) | 32.672 | 11.674 | 57.718 | 1.025 |
| 41 | Saga | a | -0.032 | -0.045 | -0.021 | 1.038 |
|  |  | b | 4.975 | 3.557 | 6.564 | 1.025 |
|  |  | c (Direct effect) | -0.135 | -0.374 | 0.105 | 1.002 |
|  |  | Indirect effect | -0.159 | -0.249 | -0.091 | 1.048 |
|  |  | Total effect | -0.294 | -0.528 | -0.068 | 1.001 |
|  |  | Indirect effect (%) | 68.574 | 23.615 | 213.394 | 1.000 |
| 42 | Nagasaki | a | -0.142 | -0.191 | -0.092 | 1.000 |
|  |  | b | 0.732 | 0.368 | 1.132 | 1.012 |
|  |  | c (Direct effect) | 0.019 | -0.256 | 0.295 | 1.002 |
|  |  | Indirect effect | -0.104 | -0.178 | -0.047 | 1.008 |
|  |  | Total effect | -0.085 | -0.344 | 0.175 | 1.001 |
|  |  | Indirect effect (%) | -25.078 | -1031.385 | 941.664 | 1.000 |
| 43 | Kumamoto | a | -0.192 | -0.238 | -0.147 | 1.001 |
|  |  | b | 0.738 | 0.392 | 1.085 | 1.001 |
|  |  | c (Direct effect) | -0.045 | -0.243 | 0.155 | 1.000 |
|  |  | Indirect effect | -0.142 | -0.223 | -0.072 | 1.001 |
|  |  | Total effect | -0.187 | -0.345 | -0.025 | 1.000 |
|  |  | Indirect effect (%) | 53.369 | 21.557 | 435.476 | 1.000 |
| 44 | Oita | a | - | - | - | - |
|  |  | b | - | - | - | - |
|  |  | c (Direct effect) | - | - | - | - |
|  |  | Indirect effect | - | - | - | - |
|  |  | Total effect | - | - | - | - |
|  |  | Indirect effect (%) | - | - | - | - |
| 45 | Miyazaki | a | -0.035 | -0.048 | -0.021 | 1.001 |
|  |  | b | 6.298 | 4.138 | 8.428 | 1.000 |
|  |  | c (Direct effect) | -0.146 | -0.427 | 0.134 | 1.000 |
|  |  | Indirect effect | -0.218 | -0.345 | -0.115 | 1.001 |
|  |  | Total effect | -0.364 | -0.637 | -0.091 | 1.000 |
|  |  | Indirect effect (%) | 67.245 | 25.821 | 208.936 | 1.000 |
| 46 | Kagoshima | a | -0.018 | -0.025 | -0.012 | 1.002 |
|  |  | b | 12.552 | 7.603 | 17.763 | 1.001 |
|  |  | c (Direct effect) | -0.032 | -0.321 | 0.261 | 1.000 |
|  |  | Indirect effect | -0.230 | -0.387 | -0.116 | 1.002 |
|  |  | Total effect | -0.263 | -0.522 | -0.002 | 1.000 |
|  |  | Indirect effect (%) | 7.940 | 14.115 | 543.656 | 1.000 |
| 47 | Okinawa | a | -0.050 | -0.074 | -0.024 | 1.019 |
|  |  | b | 1.280 | 0.656 | 1.921 | 1.014 |
|  |  | c (Direct effect) | 0.019 | -0.109 | 0.151 | 1.005 |
|  |  | Indirect effect | -0.064 | -0.117 | -0.025 | 1.023 |
|  |  | Total effect | -0.044 | -0.170 | 0.083 | 1.001 |
|  |  | Indirect effect (%) | 139.794 | -1193.382 | 1245.160 | 1.000 |

Prefectures where NPIs were not implemented are represented by "-".

**Table S6. Effects of NPIs on mitigating the COVID-19 infection mediated by nighttime human mobility in downtown areas during Phase 3**

| **Prefecture Number** | **Prefecture Name** | **Parameters** | **Mean** | **95% Credible Interval** | | **Rhat** |
| --- | --- | --- | --- | --- | --- | --- |
|  |  |  |  | **Low** | **High** |  |
| 1 | Hokkaido | a | -0.202 | -0.240 | -0.164 | 1.002 |
|  |  | b | -1.447 | -2.275 | -0.736 | 1.010 |
|  |  | c (Direct effect) | -0.480 | -0.702 | -0.293 | 1.008 |
|  |  | Indirect effect | 0.295 | 0.140 | 0.508 | 1.010 |
|  |  | Total effect | -0.185 | -0.312 | -0.062 | 1.004 |
|  |  | Indirect effect (%) | -192.118 | -558.237 | -58.577 | 1.000 |
| 2 | Aomori | a | -0.040 | -0.065 | -0.015 | 1.017 |
|  |  | b | 5.410 | 1.999 | 10.777 | 1.084 |
|  |  | c (Direct effect) | 0.154 | -0.128 | 0.552 | 1.047 |
|  |  | Indirect effect | -0.225 | -0.586 | -0.044 | 1.063 |
|  |  | Total effect | -0.071 | -0.273 | 0.133 | 1.002 |
|  |  | Indirect effect (%) | 2178.403 | -2530.131 | 2782.718 | 1.000 |
| 3 | Iwate | a | - | - | - | - |
|  |  | b | - | - | - | - |
|  |  | c (Direct effect) | - | - | - | - |
|  |  | Indirect effect | - | - | - | - |
|  |  | Total effect | - | - | - | - |
|  |  | Indirect effect (%) | - | - | - | - |
| 4 | Miyagi | a | - | - | - | - |
|  |  | b | - | - | - | - |
|  |  | c (Direct effect) | - | - | - | - |
|  |  | Indirect effect | - | - | - | - |
|  |  | Total effect | - | - | - | - |
|  |  | Indirect effect (%) | - | - | - | - |
| 5 | Akita | a | - | - | - | - |
|  |  | b | - | - | - | - |
|  |  | c (Direct effect) | - | - | - | - |
|  |  | Indirect effect | - | - | - | - |
|  |  | Total effect | - | - | - | - |
|  |  | Indirect effect (%) | - | - | - | - |
| 6 | Yamagata | a | -0.045 | -0.057 | -0.032 | 1.000 |
|  |  | b | 7.099 | 0.490 | 15.008 | 1.004 |
|  |  | c (Direct effect) | 0.110 | -0.269 | 0.576 | 1.002 |
|  |  | Indirect effect | -0.320 | -0.749 | -0.021 | 1.003 |
|  |  | Total effect | -0.210 | -0.409 | -0.010 | 1.000 |
|  |  | Indirect effect (%) | 175.800 | -35.054 | 935.143 | 1.000 |
| 7 | Fukushima | a | -0.065 | -0.075 | -0.055 | 1.000 |
|  |  | b | 5.965 | 2.540 | 9.442 | 1.003 |
|  |  | c (Direct effect) | 0.190 | -0.156 | 0.546 | 1.002 |
|  |  | Indirect effect | -0.388 | -0.646 | -0.160 | 1.002 |
|  |  | Total effect | -0.198 | -0.382 | -0.012 | 1.001 |
|  |  | Indirect effect (%) | 145.265 | 31.436 | 1272.948 | 1.000 |
| 8 | Ibaraki | a | -0.075 | -0.089 | -0.060 | 1.003 |
|  |  | b | -8.196 | -11.441 | -5.238 | 1.020 |
|  |  | c (Direct effect) | -0.797 | -1.145 | -0.492 | 1.012 |
|  |  | Indirect effect | 0.620 | 0.359 | 0.944 | 1.017 |
|  |  | Total effect | -0.177 | -0.357 | 0.010 | 1.001 |
|  |  | Indirect effect (%) | -416.833 | -2205.109 | 1181.557 | 1.000 |
| 9 | Tochigi | a | -0.072 | -0.085 | -0.059 | 1.000 |
|  |  | b | 1.567 | -0.512 | 3.682 | 1.005 |
|  |  | c (Direct effect) | -0.130 | -0.329 | 0.076 | 1.003 |
|  |  | Indirect effect | -0.113 | -0.275 | 0.037 | 1.005 |
|  |  | Total effect | -0.243 | -0.359 | -0.126 | 1.000 |
|  |  | Indirect effect (%) | 50.765 | -15.398 | 146.267 | 1.001 |
| 10 | Gunma | a | -0.204 | -0.217 | -0.190 | 1.273 |
|  |  | b | -26.989 | -68.630 | -0.012 | 9.918 |
|  |  | c (Direct effect) | -5.698 | -14.730 | -0.191 | 9.650 |
|  |  | Indirect effect | 5.531 | 0.002 | 14.534 | 9.648 |
|  |  | Total effect | -0.167 | -0.304 | -0.043 | 1.384 |
|  |  | Indirect effect (%) | -4253.474 | -15076.713 | 1.457 | 1.245 |
| 11 | Saitama | a | -0.111 | -0.140 | -0.078 | 1.038 |
|  |  | b | -2.817 | -3.632 | -1.939 | 1.072 |
|  |  | c (Direct effect) | -0.589 | -0.778 | -0.413 | 1.017 |
|  |  | Indirect effect | 0.316 | 0.175 | 0.464 | 1.065 |
|  |  | Total effect | -0.274 | -0.413 | -0.147 | 1.037 |
|  |  | Indirect effect (%) | -125.253 | -250.117 | -52.810 | 1.038 |
| 12 | Chiba | a | -0.110 | -0.141 | -0.078 | 1.004 |
|  |  | b | -1.637 | -2.503 | -0.885 | 1.047 |
|  |  | c (Direct effect) | -0.507 | -0.680 | -0.350 | 1.017 |
|  |  | Indirect effect | 0.182 | 0.084 | 0.318 | 1.040 |
|  |  | Total effect | -0.325 | -0.454 | -0.195 | 1.000 |
|  |  | Indirect effect (%) | -58.803 | -118.209 | -23.764 | 1.021 |
| 13 | Tokyo | a | -0.039 | -0.075 | 0.001 | 1.036 |
|  |  | b | -2.225 | -2.550 | -1.964 | 1.048 |
|  |  | c (Direct effect) | -0.393 | -0.482 | -0.304 | 1.037 |
|  |  | Indirect effect | 0.086 | -0.002 | 0.170 | 1.028 |
|  |  | Total effect | -0.307 | -0.421 | -0.194 | 1.006 |
|  |  | Indirect effect (%) | -31.165 | -78.886 | 0.647 | 1.013 |
| 14 | Kanagawa | a | -0.085 | -0.111 | -0.060 | 1.010 |
|  |  | b | -4.837 | -5.715 | -4.045 | 1.039 |
|  |  | c (Direct effect) | -0.744 | -0.912 | -0.595 | 1.024 |
|  |  | Indirect effect | 0.414 | 0.267 | 0.585 | 1.025 |
|  |  | Total effect | -0.330 | -0.473 | -0.193 | 1.000 |
|  |  | Indirect effect (%) | -134.934 | -267.741 | -63.842 | 1.008 |
| 15 | Niigata | a | -0.340 | -0.408 | -0.268 | 1.002 |
|  |  | b | 1.291 | 0.263 | 2.768 | 1.001 |
|  |  | c (Direct effect) | 0.085 | -0.335 | 0.718 | 1.001 |
|  |  | Indirect effect | -0.448 | -1.065 | -0.083 | 1.001 |
|  |  | Total effect | -0.363 | -0.512 | -0.213 | 1.000 |
|  |  | Indirect effect (%) | 131.355 | 20.562 | 346.373 | 1.001 |
| 16 | Toyama | a | - | - | - | - |
|  |  | b | - | - | - | - |
|  |  | c (Direct effect) | - | - | - | - |
|  |  | Indirect effect | - | - | - | - |
|  |  | Total effect | - | - | - | - |
|  |  | Indirect effect (%) | - | - | - | - |
| 17 | Ishikawa | a | -0.101 | -0.112 | -0.090 | 1.001 |
|  |  | b | -16.968 | -27.020 | -6.368 | 1.006 |
|  |  | c (Direct effect) | -2.270 | -3.407 | -1.124 | 1.006 |
|  |  | Indirect effect | 1.730 | 0.614 | 2.864 | 1.006 |
|  |  | Total effect | -0.540 | -0.693 | -0.387 | 1.000 |
|  |  | Indirect effect (%) | -327.196 | -591.771 | -114.371 | 1.005 |
| 18 | Fukui | a | - | - | - | - |
|  |  | b | - | - | - | - |
|  |  | c (Direct effect) | - | - | - | - |
|  |  | Indirect effect | - | - | - | - |
|  |  | Total effect | - | - | - | - |
|  |  | Indirect effect (%) | - | - | - | - |
| 19 | Yamanashi | a | - | - | - | - |
|  |  | b | - | - | - | - |
|  |  | c (Direct effect) | - | - | - | - |
|  |  | Indirect effect | - | - | - | - |
|  |  | Total effect | - | - | - | - |
|  |  | Indirect effect (%) | - | - | - | - |
| 20 | Nagano | a | -0.072 | -0.089 | -0.054 | 1.000 |
|  |  | b | 1.885 | 0.556 | 3.318 | 1.001 |
|  |  | c (Direct effect) | -0.104 | -0.278 | 0.080 | 1.000 |
|  |  | Indirect effect | -0.136 | -0.263 | -0.036 | 1.001 |
|  |  | Total effect | -0.241 | -0.369 | -0.111 | 1.000 |
|  |  | Indirect effect (%) | 62.323 | 13.946 | 158.386 | 1.000 |
| 21 | Gifu | a | -0.098 | -0.112 | -0.083 | 1.000 |
|  |  | b | 1.218 | -3.505 | 6.024 | 1.004 |
|  |  | c (Direct effect) | -0.369 | -0.967 | 0.231 | 1.003 |
|  |  | Indirect effect | -0.117 | -0.596 | 0.354 | 1.004 |
|  |  | Total effect | -0.487 | -0.711 | -0.261 | 1.000 |
|  |  | Indirect effect (%) | 31.617 | -64.036 | 172.600 | 1.002 |
| 22 | Shizuoka | a | -0.246 | -0.296 | -0.199 | 1.015 |
|  |  | b | 1.049 | 0.350 | 1.842 | 1.013 |
|  |  | c (Direct effect) | -0.014 | -0.268 | 0.256 | 1.010 |
|  |  | Indirect effect | -0.260 | -0.476 | -0.081 | 1.019 |
|  |  | Total effect | -0.274 | -0.452 | -0.096 | 1.000 |
|  |  | Indirect effect (%) | 108.219 | 26.239 | 290.435 | 1.000 |
| 23 | Aichi | a | -0.145 | -0.180 | -0.110 | 1.001 |
|  |  | b | -2.808 | -3.845 | -1.921 | 1.003 |
|  |  | c (Direct effect) | -0.743 | -0.987 | -0.538 | 1.002 |
|  |  | Indirect effect | 0.411 | 0.239 | 0.640 | 1.002 |
|  |  | Total effect | -0.332 | -0.464 | -0.200 | 1.001 |
|  |  | Indirect effect (%) | -131.274 | -256.737 | -63.180 | 1.002 |
| 24 | Mie | a | -0.070 | -0.081 | -0.059 | 1.001 |
|  |  | b | -11.309 | -16.447 | -6.942 | 1.004 |
|  |  | c (Direct effect) | -1.003 | -1.469 | -0.624 | 1.003 |
|  |  | Indirect effect | 0.799 | 0.443 | 1.253 | 1.004 |
|  |  | Total effect | -0.204 | -0.367 | -0.040 | 1.001 |
|  |  | Indirect effect (%) | -569.404 | -1801.890 | -155.841 | 1.000 |
| 25 | Shiga | a | - | - | - | - |
|  |  | b | - | - | - | - |
|  |  | c (Direct effect) | - | - | - | - |
|  |  | Indirect effect | - | - | - | - |
|  |  | Total effect | - | - | - | - |
|  |  | Indirect effect (%) | - | - | - | - |
| 26 | Kyoto | a | -0.157 | -0.196 | -0.116 | 1.000 |
|  |  | b | 0.961 | -0.178 | 2.456 | 1.004 |
|  |  | c (Direct effect) | -0.136 | -0.344 | 0.140 | 1.003 |
|  |  | Indirect effect | -0.152 | -0.415 | 0.028 | 1.004 |
|  |  | Total effect | -0.287 | -0.385 | -0.189 | 1.001 |
|  |  | Indirect effect (%) | 54.558 | -9.692 | 156.702 | 1.004 |
| 27 | Osaka | a | -0.100 | -0.126 | -0.074 | 1.002 |
|  |  | b | -3.330 | -4.130 | -2.601 | 1.010 |
|  |  | c (Direct effect) | -0.585 | -0.740 | -0.441 | 1.003 |
|  |  | Indirect effect | 0.335 | 0.216 | 0.477 | 1.006 |
|  |  | Total effect | -0.250 | -0.362 | -0.144 | 1.001 |
|  |  | Indirect effect (%) | -143.539 | -271.695 | -70.224 | 1.002 |
| 28 | Hyogo | a | -0.171 | -0.202 | -0.139 | 1.000 |
|  |  | b | -2.208 | -3.276 | -1.199 | 1.004 |
|  |  | c (Direct effect) | -0.664 | -0.919 | -0.439 | 1.002 |
|  |  | Indirect effect | 0.379 | 0.192 | 0.613 | 1.003 |
|  |  | Total effect | -0.285 | -0.421 | -0.147 | 1.000 |
|  |  | Indirect effect (%) | -145.377 | -310.264 | -59.129 | 1.000 |
| 29 | Nara | a | - | - | - | - |
|  |  | b | - | - | - | - |
|  |  | c (Direct effect) | - | - | - | - |
|  |  | Indirect effect | - | - | - | - |
|  |  | Total effect | - | - | - | - |
|  |  | Indirect effect (%) | - | - | - | - |
| 30 | Wakayama | a | -0.046 | -0.059 | -0.035 | 1.038 |
|  |  | b | 1.022 | -1.079 | 3.097 | 1.017 |
|  |  | c (Direct effect) | -0.041 | -0.209 | 0.152 | 1.017 |
|  |  | Indirect effect | -0.047 | -0.154 | 0.050 | 1.008 |
|  |  | Total effect | -0.088 | -0.226 | 0.074 | 1.042 |
|  |  | Indirect effect (%) | -105.804 | -551.323 | 670.066 | 1.000 |
| 31 | Tottori | a | - | - | - | - |
|  |  | b | - | - | - | - |
|  |  | c (Direct effect) | - | - | - | - |
|  |  | Indirect effect | - | - | - | - |
|  |  | Total effect | - | - | - | - |
|  |  | Indirect effect (%) | - | - | - | - |
| 32 | Shimane | a | -0.097 | -0.121 | -0.073 | 1.000 |
|  |  | b | 4.315 | 1.909 | 7.225 | 1.001 |
|  |  | c (Direct effect) | 0.084 | -0.289 | 0.535 | 1.000 |
|  |  | Indirect effect | -0.424 | -0.798 | -0.169 | 1.001 |
|  |  | Total effect | -0.339 | -0.551 | -0.123 | 1.000 |
|  |  | Indirect effect (%) | 151.871 | 39.470 | 433.300 | 1.000 |
| 33 | Okayama | a | -0.436 | -0.528 | -0.347 | 1.001 |
|  |  | b | 2.117 | 1.126 | 3.477 | 1.003 |
|  |  | c (Direct effect) | 0.843 | 0.279 | 1.671 | 1.002 |
|  |  | Indirect effect | -0.939 | -1.744 | -0.431 | 1.002 |
|  |  | Total effect | -0.096 | -0.313 | 0.122 | 1.000 |
|  |  | Indirect effect (%) | -4027.500 | -8280.966 | 9634.089 | 1.000 |
| 34 | Hiroshima | a | -0.276 | -0.310 | -0.228 | 1.002 |
|  |  | b | 6.531 | 1.461 | 14.872 | 1.010 |
|  |  | c (Direct effect) | 1.122 | -0.389 | 3.757 | 1.010 |
|  |  | Indirect effect | -1.850 | -4.489 | -0.358 | 1.010 |
|  |  | Total effect | -0.728 | -0.904 | -0.551 | 1.000 |
|  |  | Indirect effect (%) | 258.619 | 48.138 | 645.409 | 1.010 |
| 35 | Yamaguchi | a | -0.065 | -0.074 | -0.053 | 1.055 |
|  |  | b | 12.199 | 7.001 | 18.685 | 1.071 |
|  |  | c (Direct effect) | 0.456 | 0.045 | 0.980 | 1.071 |
|  |  | Indirect effect | -0.795 | -1.339 | -0.394 | 1.071 |
|  |  | Total effect | -0.340 | -0.461 | -0.221 | 1.112 |
|  |  | Indirect effect (%) | 242.154 | 111.932 | 421.848 | 1.088 |
| 36 | Tokushima | a | - | - | - | - |
|  |  | b | - | - | - | - |
|  |  | c (Direct effect) | - | - | - | - |
|  |  | Indirect effect | - | - | - | - |
|  |  | Total effect | - | - | - | - |
|  |  | Indirect effect (%) | - | - | - | - |
| 37 | Kagawa | a | -0.250 | -0.286 | -0.213 | 1.016 |
|  |  | b | 3.155 | 0.297 | 8.201 | 1.100 |
|  |  | c (Direct effect) | 0.615 | -0.116 | 2.061 | 1.099 |
|  |  | Indirect effect | -0.803 | -2.250 | -0.073 | 1.099 |
|  |  | Total effect | -0.188 | -0.322 | -0.049 | 1.005 |
|  |  | Indirect effect (%) | 532.813 | 27.262 | 2009.273 | 1.000 |
| 38 | Ehime | a | - | - | - | - |
|  |  | b | - | - | - | - |
|  |  | c (Direct effect) | - | - | - | - |
|  |  | Indirect effect | - | - | - | - |
|  |  | Total effect | - | - | - | - |
|  |  | Indirect effect (%) | - | - | - | - |
| 39 | Kochi | a | -0.034 | -0.050 | -0.018 | 1.000 |
|  |  | b | 4.915 | 3.197 | 6.862 | 1.002 |
|  |  | c (Direct effect) | 0.146 | -0.010 | 0.309 | 1.001 |
|  |  | Indirect effect | -0.168 | -0.291 | -0.073 | 1.001 |
|  |  | Total effect | -0.022 | -0.164 | 0.120 | 1.000 |
|  |  | Indirect effect (%) | 310.443 | -3277.698 | 3527.853 | 1.000 |
| 40 | Fukuoka | a | -0.137 | -0.172 | -0.102 | 1.001 |
|  |  | b | -2.751 | -4.039 | -1.628 | 1.003 |
|  |  | c (Direct effect) | -0.748 | -1.028 | -0.502 | 1.002 |
|  |  | Indirect effect | 0.381 | 0.194 | 0.633 | 1.003 |
|  |  | Total effect | -0.367 | -0.545 | -0.185 | 1.000 |
|  |  | Indirect effect (%) | -123.911 | -255.538 | -45.428 | 1.000 |
| 41 | Saga | a | -0.084 | -0.100 | -0.067 | 1.000 |
|  |  | b | 6.887 | 3.375 | 10.813 | 1.005 |
|  |  | c (Direct effect) | 0.502 | 0.143 | 0.946 | 1.004 |
|  |  | Indirect effect | -0.581 | -0.993 | -0.266 | 1.005 |
|  |  | Total effect | -0.078 | -0.245 | 0.089 | 1.000 |
|  |  | Indirect effect (%) | 1115.758 | -6976.444 | 7313.922 | 1.000 |
| 42 | Nagasaki | a | -0.353 | -0.422 | -0.293 | 1.006 |
|  |  | b | 2.817 | 1.314 | 5.648 | 1.008 |
|  |  | c (Direct effect) | 0.678 | 0.007 | 2.009 | 1.008 |
|  |  | Indirect effect | -1.020 | -2.335 | -0.422 | 1.009 |
|  |  | Total effect | -0.342 | -0.520 | -0.162 | 1.000 |
|  |  | Indirect effect (%) | 332.766 | 101.827 | 902.541 | 1.004 |
| 43 | Kumamoto | a | -0.445 | -0.517 | -0.375 | 1.000 |
|  |  | b | 1.172 | 0.330 | 2.081 | 1.003 |
|  |  | c (Direct effect) | -0.077 | -0.598 | 0.484 | 1.002 |
|  |  | Indirect effect | -0.524 | -0.978 | -0.145 | 1.003 |
|  |  | Total effect | -0.601 | -0.874 | -0.327 | 1.000 |
|  |  | Indirect effect (%) | 94.930 | 21.242 | 221.329 | 1.001 |
| 44 | Oita | a | -0.274 | -0.320 | -0.228 | 1.001 |
|  |  | b | 0.727 | -0.255 | 1.778 | 1.003 |
|  |  | c (Direct effect) | -0.053 | -0.412 | 0.329 | 1.001 |
|  |  | Indirect effect | -0.201 | -0.507 | 0.070 | 1.003 |
|  |  | Total effect | -0.254 | -0.485 | -0.023 | 1.000 |
|  |  | Indirect effect (%) | 119.594 | -55.873 | 483.107 | 1.000 |
| 45 | Miyazaki | a | -0.076 | -0.090 | -0.061 | 1.004 |
|  |  | b | -3.160 | -7.074 | 0.774 | 1.036 |
|  |  | c (Direct effect) | -0.616 | -0.995 | -0.245 | 1.037 |
|  |  | Indirect effect | 0.241 | -0.058 | 0.594 | 1.038 |
|  |  | Total effect | -0.374 | -0.590 | -0.166 | 1.005 |
|  |  | Indirect effect (%) | -71.729 | -208.246 | 16.633 | 1.018 |
| 46 | Kagoshima | a | -0.037 | -0.044 | -0.029 | 1.000 |
|  |  | b | 8.171 | 1.577 | 15.010 | 1.000 |
|  |  | c (Direct effect) | -0.116 | -0.479 | 0.272 | 1.000 |
|  |  | Indirect effect | -0.304 | -0.598 | -0.055 | 1.000 |
|  |  | Total effect | -0.419 | -0.613 | -0.223 | 1.000 |
|  |  | Indirect effect (%) | 80.100 | 11.073 | 201.072 | 1.000 |
| 47 | Okinawa | a | -0.226 | -0.261 | -0.191 | 1.001 |
|  |  | b | 2.658 | 1.089 | 4.922 | 1.004 |
|  |  | c (Direct effect) | -0.196 | -0.616 | 0.437 | 1.003 |
|  |  | Indirect effect | -0.610 | -1.236 | -0.229 | 1.004 |
|  |  | Total effect | -0.806 | -0.964 | -0.649 | 1.001 |
|  |  | Indirect effect (%) | 76.452 | 28.057 | 156.081 | 1.003 |

Prefectures where NPIs were not implemented are represented by "-".

**Table S7. Effects of NPIs on mitigating the COVID-19 infection mediated by visitors’ mobility during Phase 1**

| **Prefecture Number** | **Prefecture Name** | **Parameters** | **Mean** | **95% Credible Interval** | | **Rhat** |
| --- | --- | --- | --- | --- | --- | --- |
|  |  |  |  | **Low** | **High** |  |
| 1 | Hokkaido | a | 0.053 | -0.089 | 0.190 | 1.001 |
|  |  | b | 0.277 | 0.183 | 0.373 | 1.001 |
|  |  | c (Direct effect) | -0.331 | -0.454 | -0.211 | 1.003 |
|  |  | Indirect effect | 0.015 | -0.025 | 0.056 | 1.001 |
|  |  | Total effect | -0.316 | -0.444 | -0.189 | 1.003 |
|  |  | Indirect effect (%) | -5.400 | -22.620 | 7.220 | 1.001 |
| 2 | Aomori | a | 0.102 | -0.037 | 0.245 | 1.002 |
|  |  | b | 0.348 | 0.082 | 0.623 | 1.001 |
|  |  | c (Direct effect) | -0.038 | -0.303 | 0.228 | 1.001 |
|  |  | Indirect effect | 0.036 | -0.012 | 0.107 | 1.002 |
|  |  | Total effect | -0.003 | -0.271 | 0.266 | 1.001 |
|  |  | Indirect effect (%) | 136.699 | -414.329 | 459.231 | 1.000 |
| 3 | Iwate | a | 0.032 | -0.060 | 0.121 | 1.000 |
|  |  | b | 0.191 | -0.189 | 0.581 | 1.002 |
|  |  | c (Direct effect) | -0.102 | -0.285 | 0.084 | 1.000 |
|  |  | Indirect effect | 0.006 | -0.017 | 0.041 | 1.001 |
|  |  | Total effect | -0.096 | -0.280 | 0.090 | 1.000 |
|  |  | Indirect effect (%) | 81.628 | -130.131 | 103.561 | 1.000 |
| 4 | Miyagi | a | -0.016 | -0.099 | 0.068 | 1.000 |
|  |  | b | -0.002 | -0.282 | 0.282 | 1.000 |
|  |  | c (Direct effect) | -0.728 | -0.926 | -0.533 | 1.000 |
|  |  | Indirect effect | 0.000 | -0.014 | 0.014 | 1.000 |
|  |  | Total effect | -0.728 | -0.926 | -0.533 | 1.000 |
|  |  | Indirect effect (%) | -0.007 | -1.983 | 2.002 | 1.000 |
| 5 | Akita | a | 0.116 | -0.036 | 0.268 | 1.000 |
|  |  | b | 0.168 | -0.159 | 0.509 | 1.000 |
|  |  | c (Direct effect) | -0.363 | -0.635 | -0.092 | 1.000 |
|  |  | Indirect effect | 0.019 | -0.022 | 0.085 | 1.000 |
|  |  | Total effect | -0.343 | -0.621 | -0.071 | 1.000 |
|  |  | Indirect effect (%) | -6.061 | -47.538 | 7.975 | 1.000 |
| 6 | Yamagata | a | -0.010 | -0.133 | 0.115 | 1.000 |
|  |  | b | 0.476 | 0.215 | 0.748 | 1.000 |
|  |  | c (Direct effect) | -0.727 | -0.969 | -0.485 | 1.003 |
|  |  | Indirect effect | -0.005 | -0.071 | 0.056 | 1.000 |
|  |  | Total effect | -0.732 | -0.976 | -0.490 | 1.003 |
|  |  | Indirect effect (%) | 0.618 | -8.609 | 9.764 | 1.000 |
| 7 | Fukushima | a | -0.056 | -0.147 | 0.035 | 1.000 |
|  |  | b | 1.025 | 0.680 | 1.389 | 1.001 |
|  |  | c (Direct effect) | -0.575 | -0.777 | -0.371 | 1.000 |
|  |  | Indirect effect | -0.058 | -0.160 | 0.035 | 1.000 |
|  |  | Total effect | -0.633 | -0.841 | -0.425 | 1.000 |
|  |  | Indirect effect (%) | 9.060 | -6.380 | 25.133 | 1.000 |
| 8 | Ibaraki | a | -0.040 | -0.088 | 0.009 | 1.000 |
|  |  | b | 2.632 | 1.772 | 3.627 | 1.001 |
|  |  | c (Direct effect) | -0.503 | -0.688 | -0.306 | 1.000 |
|  |  | Indirect effect | -0.105 | -0.251 | 0.022 | 1.000 |
|  |  | Total effect | -0.608 | -0.789 | -0.421 | 1.000 |
|  |  | Indirect effect (%) | 17.130 | -4.231 | 41.028 | 1.000 |
| 9 | Tochigi | a | -0.121 | -0.165 | -0.078 | 1.008 |
|  |  | b | -0.300 | -0.909 | 0.334 | 1.010 |
|  |  | c (Direct effect) | -0.448 | -0.646 | -0.257 | 1.027 |
|  |  | Indirect effect | 0.037 | -0.038 | 0.119 | 1.010 |
|  |  | Total effect | -0.411 | -0.574 | -0.256 | 1.039 |
|  |  | Indirect effect (%) | -8.839 | -30.479 | 10.780 | 1.014 |
| 10 | Gunma | a | -0.077 | -0.140 | -0.012 | 1.000 |
|  |  | b | 0.285 | -0.304 | 0.895 | 1.002 |
|  |  | c (Direct effect) | -0.377 | -0.615 | -0.136 | 1.001 |
|  |  | Indirect effect | -0.022 | -0.088 | 0.025 | 1.001 |
|  |  | Total effect | -0.399 | -0.626 | -0.170 | 1.002 |
|  |  | Indirect effect (%) | 6.448 | -5.974 | 29.259 | 1.000 |
| 11 | Saitama | a | -0.099 | -0.118 | -0.081 | 1.000 |
|  |  | b | 2.905 | 2.013 | 3.857 | 1.004 |
|  |  | c (Direct effect) | -0.156 | -0.291 | -0.015 | 1.002 |
|  |  | Indirect effect | -0.289 | -0.401 | -0.191 | 1.003 |
|  |  | Total effect | -0.445 | -0.545 | -0.343 | 1.000 |
|  |  | Indirect effect (%) | 65.627 | 41.527 | 95.956 | 1.003 |
| 12 | Chiba | a | -0.160 | -0.188 | -0.131 | 1.000 |
|  |  | b | 1.104 | 0.427 | 1.804 | 1.001 |
|  |  | c (Direct effect) | -0.343 | -0.500 | -0.179 | 1.001 |
|  |  | Indirect effect | -0.176 | -0.294 | -0.068 | 1.001 |
|  |  | Total effect | -0.519 | -0.623 | -0.409 | 1.000 |
|  |  | Indirect effect (%) | 34.423 | 12.647 | 60.434 | 1.001 |
| 13 | Tokyo | a | -0.053 | -0.112 | 0.006 | 1.004 |
|  |  | b | 0.572 | 0.321 | 0.833 | 1.006 |
|  |  | c (Direct effect) | -0.423 | -0.512 | -0.335 | 1.002 |
|  |  | Indirect effect | -0.031 | -0.078 | 0.003 | 1.005 |
|  |  | Total effect | -0.455 | -0.537 | -0.372 | 1.000 |
|  |  | Indirect effect (%) | 6.937 | -0.704 | 17.324 | 1.005 |
| 14 | Kanagawa | a | -0.115 | -0.139 | -0.092 | 1.001 |
|  |  | b | 0.844 | 0.304 | 1.354 | 1.002 |
|  |  | c (Direct effect) | -0.213 | -0.329 | -0.093 | 1.000 |
|  |  | Indirect effect | -0.098 | -0.165 | -0.034 | 1.002 |
|  |  | Total effect | -0.310 | -0.408 | -0.207 | 1.000 |
|  |  | Indirect effect (%) | 32.338 | 10.764 | 60.277 | 1.001 |
| 15 | Niigata | a | 0.112 | -0.039 | 0.264 | 1.000 |
|  |  | b | 0.176 | -0.025 | 0.374 | 1.000 |
|  |  | c (Direct effect) | -0.330 | -0.532 | -0.125 | 1.000 |
|  |  | Indirect effect | 0.020 | -0.009 | 0.068 | 1.000 |
|  |  | Total effect | -0.310 | -0.515 | -0.100 | 1.000 |
|  |  | Indirect effect (%) | -8.697 | -37.595 | 2.923 | 1.000 |
| 16 | Toyama | a | -0.025 | -0.128 | 0.078 | 1.000 |
|  |  | b | 1.522 | 1.113 | 1.973 | 1.000 |
|  |  | c (Direct effect) | -0.491 | -0.742 | -0.236 | 1.000 |
|  |  | Indirect effect | -0.039 | -0.200 | 0.119 | 1.000 |
|  |  | Total effect | -0.530 | -0.804 | -0.253 | 1.000 |
|  |  | Indirect effect (%) | 5.756 | -33.006 | 36.101 | 1.000 |
| 17 | Ishikawa | a | -0.001 | -0.112 | 0.110 | 1.000 |
|  |  | b | 0.897 | 0.566 | 1.249 | 1.001 |
|  |  | c (Direct effect) | -0.495 | -0.722 | -0.266 | 1.000 |
|  |  | Indirect effect | -0.002 | -0.107 | 0.099 | 1.000 |
|  |  | Total effect | -0.497 | -0.733 | -0.259 | 1.000 |
|  |  | Indirect effect (%) | -0.600 | -27.230 | 21.253 | 1.000 |
| 18 | Fukui | a | -0.133 | -0.226 | -0.038 | 1.000 |
|  |  | b | 0.526 | -0.015 | 1.054 | 1.000 |
|  |  | c (Direct effect) | -0.528 | -0.821 | -0.232 | 1.000 |
|  |  | Indirect effect | -0.070 | -0.174 | 0.003 | 1.000 |
|  |  | Total effect | -0.597 | -0.863 | -0.329 | 1.000 |
|  |  | Indirect effect (%) | 12.806 | -0.454 | 36.269 | 1.000 |
| 19 | Yamanashi | a | 0.047 | -0.102 | 0.196 | 1.000 |
|  |  | b | 0.306 | 0.084 | 0.528 | 1.001 |
|  |  | c (Direct effect) | -0.310 | -0.549 | -0.074 | 1.002 |
|  |  | Indirect effect | 0.014 | -0.033 | 0.070 | 1.000 |
|  |  | Total effect | -0.295 | -0.539 | -0.054 | 1.002 |
|  |  | Indirect effect (%) | -11.516 | -48.765 | 14.893 | 1.000 |
| 20 | Nagano | a | 0.080 | -0.097 | 0.257 | 1.000 |
|  |  | b | 0.605 | 0.434 | 0.794 | 1.001 |
|  |  | c (Direct effect) | -0.534 | -0.730 | -0.341 | 1.000 |
|  |  | Indirect effect | 0.048 | -0.058 | 0.162 | 1.000 |
|  |  | Total effect | -0.486 | -0.702 | -0.270 | 1.000 |
|  |  | Indirect effect (%) | -12.026 | -47.650 | 10.229 | 1.000 |
| 21 | Gifu | a | -0.178 | -0.215 | -0.140 | 1.004 |
|  |  | b | -0.242 | -1.030 | 0.504 | 1.015 |
|  |  | c (Direct effect) | -0.585 | -0.760 | -0.418 | 1.002 |
|  |  | Indirect effect | 0.043 | -0.090 | 0.186 | 1.013 |
|  |  | Total effect | -0.542 | -0.659 | -0.426 | 1.022 |
|  |  | Indirect effect (%) | -8.056 | -36.026 | 16.924 | 1.017 |
| 22 | Shizuoka | a | -0.029 | -0.143 | 0.086 | 1.000 |
|  |  | b | 0.541 | 0.250 | 0.848 | 1.000 |
|  |  | c (Direct effect) | -0.662 | -0.889 | -0.431 | 1.000 |
|  |  | Indirect effect | -0.016 | -0.086 | 0.048 | 1.000 |
|  |  | Total effect | -0.678 | -0.911 | -0.440 | 1.000 |
|  |  | Indirect effect (%) | 2.197 | -7.998 | 12.656 | 1.000 |
| 23 | Aichi | a | -0.065 | -0.096 | -0.033 | 1.005 |
|  |  | b | 1.088 | 0.524 | 1.608 | 1.013 |
|  |  | c (Direct effect) | -0.069 | -0.279 | 0.159 | 1.006 |
|  |  | Indirect effect | -0.071 | -0.124 | -0.026 | 1.016 |
|  |  | Total effect | -0.140 | -0.349 | 0.092 | 1.010 |
|  |  | Indirect effect (%) | 323.728 | -383.517 | 497.210 | 1.000 |
| 24 | Mie | a | -0.095 | -0.188 | 0.001 | 1.000 |
|  |  | b | 0.884 | 0.453 | 1.355 | 1.001 |
|  |  | c (Direct effect) | -0.457 | -0.677 | -0.230 | 1.000 |
|  |  | Indirect effect | -0.085 | -0.200 | 0.000 | 1.000 |
|  |  | Total effect | -0.542 | -0.758 | -0.319 | 1.000 |
|  |  | Indirect effect (%) | 15.977 | -0.076 | 39.351 | 1.000 |
| 25 | Shiga | a | -0.101 | -0.167 | -0.034 | 1.000 |
|  |  | b | 0.756 | 0.220 | 1.368 | 1.002 |
|  |  | c (Direct effect) | -0.431 | -0.654 | -0.205 | 1.000 |
|  |  | Indirect effect | -0.077 | -0.175 | -0.014 | 1.001 |
|  |  | Total effect | -0.508 | -0.715 | -0.301 | 1.000 |
|  |  | Indirect effect (%) | 15.979 | 2.541 | 39.835 | 1.001 |
| 26 | Kyoto | a | -0.086 | -0.123 | -0.049 | 1.008 |
|  |  | b | 0.605 | 0.290 | 0.926 | 1.000 |
|  |  | c (Direct effect) | -0.542 | -0.663 | -0.418 | 1.000 |
|  |  | Indirect effect | -0.052 | -0.092 | -0.021 | 1.003 |
|  |  | Total effect | -0.594 | -0.705 | -0.480 | 1.000 |
|  |  | Indirect effect (%) | 8.894 | 3.324 | 16.469 | 1.003 |
| 27 | Osaka | a | -0.081 | -0.112 | -0.050 | 1.002 |
|  |  | b | 1.391 | 1.011 | 1.763 | 1.002 |
|  |  | c (Direct effect) | -0.166 | -0.282 | -0.048 | 1.000 |
|  |  | Indirect effect | -0.113 | -0.171 | -0.063 | 1.003 |
|  |  | Total effect | -0.279 | -0.395 | -0.159 | 1.001 |
|  |  | Indirect effect (%) | 42.090 | 21.753 | 72.650 | 1.001 |
| 28 | Hyogo | a | -0.135 | -0.172 | -0.098 | 1.000 |
|  |  | b | 2.255 | 1.372 | 3.198 | 1.001 |
|  |  | c (Direct effect) | 0.043 | -0.143 | 0.241 | 1.000 |
|  |  | Indirect effect | -0.304 | -0.463 | -0.170 | 1.000 |
|  |  | Total effect | -0.261 | -0.401 | -0.118 | 1.000 |
|  |  | Indirect effect (%) | 128.388 | 58.484 | 267.363 | 1.000 |
| 29 | Nara | a | -0.087 | -0.133 | -0.040 | 1.000 |
|  |  | b | 3.715 | 2.184 | 5.567 | 1.000 |
|  |  | c (Direct effect) | -0.255 | -0.533 | 0.075 | 1.000 |
|  |  | Indirect effect | -0.326 | -0.618 | -0.115 | 1.000 |
|  |  | Total effect | -0.581 | -0.779 | -0.376 | 1.000 |
|  |  | Indirect effect (%) | 57.579 | 20.336 | 115.421 | 1.000 |
| 30 | Wakayama | a | -0.005 | -0.129 | 0.122 | 1.000 |
|  |  | b | 0.447 | 0.052 | 0.843 | 1.002 |
|  |  | c (Direct effect) | -0.099 | -0.370 | 0.170 | 1.000 |
|  |  | Indirect effect | -0.001 | -0.063 | 0.067 | 1.000 |
|  |  | Total effect | -0.100 | -0.375 | 0.174 | 1.000 |
|  |  | Indirect effect (%) | 11.626 | -197.342 | 224.445 | 1.000 |
| 31 | Tottori | a | -0.016 | -0.101 | 0.070 | 1.000 |
|  |  | b | 0.327 | -0.131 | 0.790 | 1.000 |
|  |  | c (Direct effect) | -0.171 | -0.427 | 0.084 | 1.000 |
|  |  | Indirect effect | -0.005 | -0.047 | 0.029 | 1.000 |
|  |  | Total effect | -0.176 | -0.431 | 0.078 | 1.000 |
|  |  | Indirect effect (%) | -1.699 | -61.212 | 75.340 | 1.000 |
| 32 | Shimane | a | -0.039 | -0.118 | 0.043 | 1.000 |
|  |  | b | 0.851 | 0.228 | 1.494 | 1.000 |
|  |  | c (Direct effect) | -0.383 | -0.712 | -0.056 | 1.000 |
|  |  | Indirect effect | -0.034 | -0.124 | 0.035 | 1.000 |
|  |  | Total effect | -0.417 | -0.745 | -0.092 | 1.000 |
|  |  | Indirect effect (%) | 5.095 | -12.826 | 45.898 | 1.000 |
| 33 | Okayama | a | 0.028 | -0.047 | 0.102 | 1.000 |
|  |  | b | 0.645 | 0.150 | 1.175 | 1.001 |
|  |  | c (Direct effect) | -0.094 | -0.311 | 0.131 | 1.001 |
|  |  | Indirect effect | 0.019 | -0.030 | 0.082 | 1.000 |
|  |  | Total effect | -0.076 | -0.296 | 0.155 | 1.001 |
|  |  | Indirect effect (%) | 13.999 | -298.365 | 287.714 | 1.000 |
| 34 | Hiroshima | a | -0.022 | -0.093 | 0.049 | 1.000 |
|  |  | b | 0.817 | 0.245 | 1.409 | 1.001 |
|  |  | c (Direct effect) | -0.978 | -1.226 | -0.733 | 1.000 |
|  |  | Indirect effect | -0.019 | -0.092 | 0.040 | 1.000 |
|  |  | Total effect | -0.997 | -1.246 | -0.751 | 1.000 |
|  |  | Indirect effect (%) | 1.828 | -4.270 | 9.047 | 1.000 |
| 35 | Yamaguchi | a | -0.001 | -0.077 | 0.074 | 1.000 |
|  |  | b | 0.748 | 0.175 | 1.337 | 1.000 |
|  |  | c (Direct effect) | -0.257 | -0.528 | 0.014 | 1.001 |
|  |  | Indirect effect | -0.002 | -0.068 | 0.060 | 1.000 |
|  |  | Total effect | -0.258 | -0.531 | 0.013 | 1.001 |
|  |  | Indirect effect (%) | -2.362 | -58.344 | 52.699 | 1.000 |
| 36 | Tokushima | a | 0.151 | 0.022 | 0.277 | 1.001 |
|  |  | b | 0.322 | -0.008 | 0.644 | 1.002 |
|  |  | c (Direct effect) | -0.106 | -0.366 | 0.151 | 1.000 |
|  |  | Indirect effect | 0.049 | -0.003 | 0.129 | 1.002 |
|  |  | Total effect | -0.057 | -0.321 | 0.207 | 1.000 |
|  |  | Indirect effect (%) | -101.890 | -616.738 | 568.380 | 1.000 |
| 37 | Kagawa | a | 0.002 | -0.100 | 0.105 | 1.001 |
|  |  | b | 0.174 | -0.223 | 0.595 | 1.003 |
|  |  | c (Direct effect) | -0.620 | -0.870 | -0.370 | 1.003 |
|  |  | Indirect effect | 0.000 | -0.033 | 0.029 | 1.000 |
|  |  | Total effect | -0.621 | -0.871 | -0.371 | 1.003 |
|  |  | Indirect effect (%) | 0.022 | -5.562 | 5.653 | 1.000 |
| 38 | Ehime | a | -0.002 | -0.109 | 0.105 | 1.001 |
|  |  | b | 0.534 | 0.098 | 0.987 | 1.001 |
|  |  | c (Direct effect) | 0.285 | 0.016 | 0.551 | 1.000 |
|  |  | Indirect effect | -0.001 | -0.067 | 0.063 | 1.001 |
|  |  | Total effect | 0.284 | 0.010 | 0.553 | 1.000 |
|  |  | Indirect effect (%) | -16.103 | -46.440 | 32.242 | 1.000 |
| 39 | Kochi | a | 0.076 | -0.069 | 0.222 | 1.000 |
|  |  | b | 0.603 | 0.290 | 0.965 | 1.001 |
|  |  | c (Direct effect) | -0.608 | -0.884 | -0.339 | 1.001 |
|  |  | Indirect effect | 0.047 | -0.041 | 0.154 | 1.000 |
|  |  | Total effect | -0.561 | -0.841 | -0.287 | 1.001 |
|  |  | Indirect effect (%) | -9.724 | -37.279 | 6.966 | 1.000 |
| 40 | Fukuoka | a | -0.064 | -0.123 | -0.003 | 1.001 |
|  |  | b | -0.408 | -0.963 | 0.127 | 1.001 |
|  |  | c (Direct effect) | -0.945 | -1.143 | -0.750 | 1.000 |
|  |  | Indirect effect | 0.027 | -0.008 | 0.084 | 1.001 |
|  |  | Total effect | -0.918 | -1.111 | -0.728 | 1.000 |
|  |  | Indirect effect (%) | -2.929 | -9.609 | 0.940 | 1.001 |
| 41 | Saga | a | -0.084 | -0.131 | -0.036 | 1.000 |
|  |  | b | 2.566 | 1.195 | 4.112 | 1.009 |
|  |  | c (Direct effect) | 0.334 | 0.006 | 0.668 | 1.001 |
|  |  | Indirect effect | -0.217 | -0.424 | -0.070 | 1.004 |
|  |  | Total effect | 0.117 | -0.162 | 0.403 | 1.000 |
|  |  | Indirect effect (%) | -119.855 | -1866.295 | 1589.535 | 1.000 |
| 42 | Nagasaki | a | 0.030 | -0.093 | 0.151 | 1.002 |
|  |  | b | 0.251 | -0.063 | 0.581 | 1.005 |
|  |  | c (Direct effect) | -0.316 | -0.536 | -0.099 | 1.001 |
|  |  | Indirect effect | 0.008 | -0.027 | 0.052 | 1.001 |
|  |  | Total effect | -0.308 | -0.531 | -0.089 | 1.001 |
|  |  | Indirect effect (%) | -3.789 | -27.650 | 10.262 | 1.000 |
| 43 | Kumamoto | a | 0.019 | -0.066 | 0.104 | 1.000 |
|  |  | b | 1.206 | 0.396 | 2.106 | 1.002 |
|  |  | c (Direct effect) | -0.427 | -0.686 | -0.172 | 1.001 |
|  |  | Indirect effect | 0.022 | -0.084 | 0.143 | 1.000 |
|  |  | Total effect | -0.405 | -0.662 | -0.144 | 1.001 |
|  |  | Indirect effect (%) | 18.772 | -57.953 | 21.997 | 1.000 |
| 44 | Oita | a | -0.029 | -0.128 | 0.071 | 1.000 |
|  |  | b | 1.186 | 0.579 | 1.984 | 1.007 |
|  |  | c (Direct effect) | -0.330 | -0.557 | -0.096 | 1.000 |
|  |  | Indirect effect | -0.036 | -0.175 | 0.083 | 1.000 |
|  |  | Total effect | -0.366 | -0.589 | -0.142 | 1.000 |
|  |  | Indirect effect (%) | 8.897 | -32.713 | 54.005 | 1.000 |
| 45 | Miyazaki | a | 0.014 | -0.085 | 0.114 | 1.000 |
|  |  | b | 1.083 | 0.456 | 1.807 | 1.008 |
|  |  | c (Direct effect) | -0.204 | -0.511 | 0.106 | 1.001 |
|  |  | Indirect effect | 0.016 | -0.095 | 0.140 | 1.000 |
|  |  | Total effect | -0.188 | -0.508 | 0.135 | 1.000 |
|  |  | Indirect effect (%) | 581.562 | -276.381 | 242.998 | 1.000 |
| 46 | Kagoshima | a | 0.032 | -0.080 | 0.146 | 1.014 |
|  |  | b | 0.314 | -0.143 | 0.706 | 1.040 |
|  |  | c (Direct effect) | -0.268 | -0.594 | 0.057 | 1.001 |
|  |  | Indirect effect | 0.010 | -0.033 | 0.063 | 1.006 |
|  |  | Total effect | -0.258 | -0.585 | 0.068 | 1.001 |
|  |  | Indirect effect (%) | 4.430 | -66.704 | 47.675 | 1.000 |
| 47 | Okinawa | a | 0.106 | -0.039 | 0.250 | 1.001 |
|  |  | b | 0.383 | 0.258 | 0.508 | 1.001 |
|  |  | c (Direct effect) | -0.865 | -1.072 | -0.661 | 1.000 |
|  |  | Indirect effect | 0.041 | -0.015 | 0.102 | 1.001 |
|  |  | Total effect | -0.825 | -1.038 | -0.613 | 1.000 |
|  |  | Indirect effect (%) | -5.164 | -13.972 | 1.727 | 1.001 |

**Table S8. Effects of NPIs on mitigating the COVID-19 infection mediated by visitors’ mobility during Phase 2**

| **Prefecture Number** | **Prefecture Name** | **Parameters** | **Mean** | **95% Credible Interval** | | **Rhat** |
| --- | --- | --- | --- | --- | --- | --- |
|  |  |  |  | **Low** | **High** |  |
| 1 | Hokkaido | a | -0.118 | -0.210 | -0.025 | 1.000 |
|  |  | b | 0.517 | 0.315 | 0.720 | 1.003 |
|  |  | c (Direct effect) | -0.112 | -0.206 | -0.014 | 1.000 |
|  |  | Indirect effect | -0.061 | -0.117 | -0.012 | 1.001 |
|  |  | Total effect | -0.173 | -0.270 | -0.073 | 1.000 |
|  |  | Indirect effect (%) | 37.951 | 8.424 | 84.166 | 1.000 |
| 2 | Aomori | a | - | - | - | - |
|  |  | b | - | - | - | - |
|  |  | c (Direct effect) | - | - | - | - |
|  |  | Indirect effect | - | - | - | - |
|  |  | Total effect | - | - | - | - |
|  |  | Indirect effect (%) | - | - | - | - |
| 3 | Iwate | a | - | - | - | - |
|  |  | b | - | - | - | - |
|  |  | c (Direct effect) | - | - | - | - |
|  |  | Indirect effect | - | - | - | - |
|  |  | Total effect | - | - | - | - |
|  |  | Indirect effect (%) | - | - | - | - |
| 4 | Miyagi | a | 0.111 | 0.050 | 0.172 | 1.001 |
|  |  | b | -0.521 | -0.942 | -0.164 | 1.004 |
|  |  | c (Direct effect) | -0.083 | -0.222 | 0.064 | 1.001 |
|  |  | Indirect effect | -0.059 | -0.137 | -0.013 | 1.003 |
|  |  | Total effect | -0.143 | -0.265 | -0.013 | 1.000 |
|  |  | Indirect effect (%) | 57.157 | 4.274 | 237.739 | 1.000 |
| 5 | Akita | a | - | - | - | - |
|  |  | b | - | - | - | - |
|  |  | c (Direct effect) | - | - | - | - |
|  |  | Indirect effect | - | - | - | - |
|  |  | Total effect | - | - | - | - |
|  |  | Indirect effect (%) | - | - | - | - |
| 6 | Yamagata | a | - | - | - | - |
|  |  | b | - | - | - | - |
|  |  | c (Direct effect) | - | - | - | - |
|  |  | Indirect effect | - | - | - | - |
|  |  | Total effect | - | - | - | - |
|  |  | Indirect effect (%) | - | - | - | - |
| 7 | Fukushima | a | 0.265 | 0.160 | 0.370 | 1.000 |
|  |  | b | 0.833 | 0.548 | 1.171 | 1.006 |
|  |  | c (Direct effect) | -0.497 | -0.649 | -0.350 | 1.001 |
|  |  | Indirect effect | 0.219 | 0.119 | 0.340 | 1.002 |
|  |  | Total effect | -0.278 | -0.437 | -0.120 | 1.000 |
|  |  | Indirect effect (%) | -91.865 | -236.626 | -32.031 | 1.000 |
| 8 | Ibaraki | a | 0.015 | -0.036 | 0.065 | 1.015 |
|  |  | b | 0.565 | -0.498 | 2.697 | 1.084 |
|  |  | c (Direct effect) | -0.178 | -0.318 | -0.023 | 1.013 |
|  |  | Indirect effect | 0.002 | -0.066 | 0.045 | 1.047 |
|  |  | Total effect | -0.176 | -0.319 | -0.028 | 1.002 |
|  |  | Indirect effect (%) | -3.271 | -57.408 | 58.616 | 1.000 |
| 9 | Tochigi | a | 0.054 | 0.009 | 0.105 | 1.005 |
|  |  | b | 4.012 | 1.564 | 8.550 | 1.036 |
|  |  | c (Direct effect) | -0.277 | -0.508 | -0.100 | 1.009 |
|  |  | Indirect effect | 0.204 | 0.035 | 0.448 | 1.007 |
|  |  | Total effect | -0.073 | -0.223 | 0.089 | 1.001 |
|  |  | Indirect effect (%) | -286.177 | -3024.710 | 2899.856 | 1.000 |
| 10 | Gunma | a | 0.008 | -0.027 | 0.045 | 1.000 |
|  |  | b | 2.886 | 1.547 | 4.797 | 1.004 |
|  |  | c (Direct effect) | -0.301 | -0.413 | -0.176 | 1.000 |
|  |  | Indirect effect | 0.020 | -0.091 | 0.123 | 1.000 |
|  |  | Total effect | -0.281 | -0.396 | -0.160 | 1.000 |
|  |  | Indirect effect (%) | -9.799 | -61.053 | 29.936 | 1.000 |
| 11 | Saitama | a | -0.013 | -0.030 | 0.005 | 1.002 |
|  |  | b | 0.673 | -0.099 | 1.585 | 1.010 |
|  |  | c (Direct effect) | -0.006 | -0.080 | 0.070 | 1.000 |
|  |  | Indirect effect | -0.009 | -0.035 | 0.003 | 1.007 |
|  |  | Total effect | -0.016 | -0.088 | 0.058 | 1.000 |
|  |  | Indirect effect (%) | 1327.691 | -383.651 | 376.899 | 1.000 |
| 12 | Chiba | a | -0.017 | -0.047 | 0.013 | 1.002 |
|  |  | b | 0.898 | 0.334 | 1.596 | 1.005 |
|  |  | c (Direct effect) | -0.019 | -0.097 | 0.062 | 1.001 |
|  |  | Indirect effect | -0.017 | -0.059 | 0.009 | 1.002 |
|  |  | Total effect | -0.036 | -0.112 | 0.040 | 1.000 |
|  |  | Indirect effect (%) | 69.580 | -411.906 | 467.821 | 1.000 |
| 13 | Tokyo | a | -0.039 | -0.078 | -0.003 | 1.038 |
|  |  | b | -0.319 | -0.500 | -0.130 | 1.017 |
|  |  | c (Direct effect) | -0.010 | -0.060 | 0.040 | 1.001 |
|  |  | Indirect effect | 0.012 | 0.001 | 0.029 | 1.027 |
|  |  | Total effect | 0.003 | -0.047 | 0.052 | 1.002 |
|  |  | Indirect effect (%) | 88.496 | -820.335 | 794.632 | 1.000 |
| 14 | Kanagawa | a | -0.013 | -0.031 | 0.005 | 1.016 |
|  |  | b | -0.003 | -0.545 | 0.488 | 1.006 |
|  |  | c (Direct effect) | -0.110 | -0.187 | -0.034 | 1.004 |
|  |  | Indirect effect | 0.000 | -0.009 | 0.009 | 1.003 |
|  |  | Total effect | -0.110 | -0.187 | -0.034 | 1.004 |
|  |  | Indirect effect (%) | -0.060 | -10.089 | 10.640 | 1.000 |
| 15 | Niigata | a | - | - | - | - |
|  |  | b | - | - | - | - |
|  |  | c (Direct effect) | - | - | - | - |
|  |  | Indirect effect | - | - | - | - |
|  |  | Total effect | - | - | - | - |
|  |  | Indirect effect (%) | - | - | - | - |
| 16 | Toyama | a | -0.255 | -0.357 | -0.155 | 1.000 |
|  |  | b | 0.370 | 0.055 | 0.703 | 1.014 |
|  |  | c (Direct effect) | -0.470 | -0.681 | -0.258 | 1.000 |
|  |  | Indirect effect | -0.094 | -0.192 | -0.013 | 1.010 |
|  |  | Total effect | -0.564 | -0.787 | -0.342 | 1.003 |
|  |  | Indirect effect (%) | 16.858 | 2.578 | 34.869 | 1.006 |
| 17 | Ishikawa | a | -0.168 | -0.244 | -0.091 | 1.000 |
|  |  | b | 0.462 | 0.142 | 0.800 | 1.002 |
|  |  | c (Direct effect) | -0.221 | -0.369 | -0.072 | 1.000 |
|  |  | Indirect effect | -0.077 | -0.153 | -0.021 | 1.001 |
|  |  | Total effect | -0.299 | -0.438 | -0.160 | 1.001 |
|  |  | Indirect effect (%) | 27.477 | 6.841 | 61.326 | 1.000 |
| 18 | Fukui | a | - | - | - | - |
|  |  | b | - | - | - | - |
|  |  | c (Direct effect) | - | - | - | - |
|  |  | Indirect effect | - | - | - | - |
|  |  | Total effect | - | - | - | - |
|  |  | Indirect effect (%) | - | - | - | - |
| 19 | Yamanashi | a | -0.285 | -0.437 | -0.133 | 1.000 |
|  |  | b | 0.424 | 0.088 | 0.785 | 1.000 |
|  |  | c (Direct effect) | -0.206 | -0.433 | 0.023 | 1.000 |
|  |  | Indirect effect | -0.119 | -0.247 | -0.022 | 1.000 |
|  |  | Total effect | -0.325 | -0.560 | -0.093 | 1.000 |
|  |  | Indirect effect (%) | 41.660 | 6.941 | 113.405 | 1.000 |
| 20 | Nagano | a | - | - | - | - |
|  |  | b | - | - | - | - |
|  |  | c (Direct effect) | - | - | - | - |
|  |  | Indirect effect | - | - | - | - |
|  |  | Total effect | - | - | - | - |
|  |  | Indirect effect (%) | - | - | - | - |
| 21 | Gifu | a | -0.094 | -0.123 | -0.064 | 1.001 |
|  |  | b | 6.510 | 3.317 | 13.212 | 1.029 |
|  |  | c (Direct effect) | 0.385 | -0.011 | 1.184 | 1.028 |
|  |  | Indirect effect | -0.630 | -1.426 | -0.252 | 1.028 |
|  |  | Total effect | -0.244 | -0.357 | -0.126 | 1.000 |
|  |  | Indirect effect (%) | 273.941 | 96.307 | 719.066 | 1.021 |
| 22 | Shizuoka | a | 0.243 | 0.148 | 0.339 | 1.000 |
|  |  | b | 1.571 | 1.107 | 2.138 | 1.003 |
|  |  | c (Direct effect) | -0.401 | -0.579 | -0.227 | 1.001 |
|  |  | Indirect effect | 0.382 | 0.207 | 0.588 | 1.002 |
|  |  | Total effect | -0.019 | -0.176 | 0.139 | 1.000 |
|  |  | Indirect effect (%) | 64.385 | -8016.436 | 7226.954 | 1.000 |
| 23 | Aichi | a | 0.019 | 0.001 | 0.037 | 1.035 |
|  |  | b | 0.553 | 0.149 | 0.975 | 1.053 |
|  |  | c (Direct effect) | -0.027 | -0.090 | 0.034 | 1.004 |
|  |  | Indirect effect | 0.011 | 0.000 | 0.026 | 1.059 |
|  |  | Total effect | -0.017 | -0.080 | 0.045 | 1.002 |
|  |  | Indirect effect (%) | -3.305 | -470.016 | 459.016 | 1.000 |
| 24 | Mie | a | -0.174 | -0.221 | -0.126 | 1.000 |
|  |  | b | 1.495 | 0.901 | 2.224 | 1.002 |
|  |  | c (Direct effect) | -0.097 | -0.244 | 0.078 | 1.001 |
|  |  | Indirect effect | -0.260 | -0.417 | -0.144 | 1.001 |
|  |  | Total effect | -0.356 | -0.452 | -0.259 | 1.000 |
|  |  | Indirect effect (%) | 74.148 | 39.229 | 125.054 | 1.001 |
| 25 | Shiga | a | -0.007 | -0.063 | 0.055 | 1.002 |
|  |  | b | 2.209 | 1.235 | 3.436 | 1.006 |
|  |  | c (Direct effect) | 0.004 | -0.154 | 0.175 | 1.001 |
|  |  | Indirect effect | -0.021 | -0.171 | 0.103 | 1.002 |
|  |  | Total effect | -0.017 | -0.186 | 0.158 | 1.000 |
|  |  | Indirect effect (%) | 95.576 | -789.313 | 898.308 | 1.000 |
| 26 | Kyoto | a | -0.017 | -0.041 | 0.005 | 1.014 |
|  |  | b | -0.357 | -0.813 | 0.118 | 1.051 |
|  |  | c (Direct effect) | -0.295 | -0.394 | -0.197 | 1.007 |
|  |  | Indirect effect | 0.006 | -0.003 | 0.021 | 1.012 |
|  |  | Total effect | -0.289 | -0.388 | -0.190 | 1.009 |
|  |  | Indirect effect (%) | -2.177 | -8.383 | 1.093 | 1.018 |
| 27 | Osaka | a | -0.023 | -0.048 | 0.008 | 1.013 |
|  |  | b | 0.105 | -0.192 | 0.395 | 1.097 |
|  |  | c (Direct effect) | -0.100 | -0.172 | -0.035 | 1.041 |
|  |  | Indirect effect | -0.002 | -0.012 | 0.005 | 1.056 |
|  |  | Total effect | -0.103 | -0.176 | -0.036 | 1.047 |
|  |  | Indirect effect (%) | 2.463 | -6.407 | 12.013 | 1.001 |
| 28 | Hyogo | a | 0.001 | -0.020 | 0.022 | 1.001 |
|  |  | b | 1.083 | 0.673 | 1.614 | 1.009 |
|  |  | c (Direct effect) | -0.149 | -0.209 | -0.087 | 1.001 |
|  |  | Indirect effect | 0.002 | -0.021 | 0.026 | 1.001 |
|  |  | Total effect | -0.147 | -0.211 | -0.083 | 1.001 |
|  |  | Indirect effect (%) | -1.755 | -22.062 | 13.493 | 1.001 |
| 29 | Nara | a | - | - | - | - |
|  |  | b | - | - | - | - |
|  |  | c (Direct effect) | - | - | - | - |
|  |  | Indirect effect | - | - | - | - |
|  |  | Total effect | - | - | - | - |
|  |  | Indirect effect (%) | - | - | - | - |
| 30 | Wakayama | a | - | - | - | - |
|  |  | b | - | - | - | - |
|  |  | c (Direct effect) | - | - | - | - |
|  |  | Indirect effect | - | - | - | - |
|  |  | Total effect | - | - | - | - |
|  |  | Indirect effect (%) | - | - | - | - |
| 31 | Tottori | a | - | - | - | - |
|  |  | b | - | - | - | - |
|  |  | c (Direct effect) | - | - | - | - |
|  |  | Indirect effect | - | - | - | - |
|  |  | Total effect | - | - | - | - |
|  |  | Indirect effect (%) | - | - | - | - |
| 32 | Shimane | a | - | - | - | - |
|  |  | b | - | - | - | - |
|  |  | c (Direct effect) | - | - | - | - |
|  |  | Indirect effect | - | - | - | - |
|  |  | Total effect | - | - | - | - |
|  |  | Indirect effect (%) | - | - | - | - |
| 33 | Okayama | a | -0.138 | -0.188 | -0.089 | 1.000 |
|  |  | b | 0.366 | 0.005 | 0.750 | 1.003 |
|  |  | c (Direct effect) | -0.307 | -0.470 | -0.150 | 1.002 |
|  |  | Indirect effect | -0.050 | -0.113 | -0.001 | 1.003 |
|  |  | Total effect | -0.357 | -0.518 | -0.209 | 1.002 |
|  |  | Indirect effect (%) | 14.756 | 0.186 | 36.175 | 1.002 |
| 34 | Hiroshima | a | -0.114 | -0.156 | -0.072 | 1.001 |
|  |  | b | 1.132 | 0.789 | 1.507 | 1.007 |
|  |  | c (Direct effect) | -0.179 | -0.297 | -0.056 | 1.001 |
|  |  | Indirect effect | -0.129 | -0.201 | -0.072 | 1.003 |
|  |  | Total effect | -0.308 | -0.421 | -0.193 | 1.001 |
|  |  | Indirect effect (%) | 43.291 | 22.846 | 74.121 | 1.002 |
| 35 | Yamaguchi | a | - | - | - | - |
|  |  | b | - | - | - | - |
|  |  | c (Direct effect) | - | - | - | - |
|  |  | Indirect effect | - | - | - | - |
|  |  | Total effect | - | - | - | - |
|  |  | Indirect effect (%) | - | - | - | - |
| 36 | Tokushima | a | - | - | - | - |
|  |  | b | - | - | - | - |
|  |  | c (Direct effect) | - | - | - | - |
|  |  | Indirect effect | - | - | - | - |
|  |  | Total effect | - | - | - | - |
|  |  | Indirect effect (%) | - | - | - | - |
| 37 | Kagawa | a | -0.110 | -0.209 | -0.012 | 1.000 |
|  |  | b | 0.948 | 0.631 | 1.301 | 1.003 |
|  |  | c (Direct effect) | -0.454 | -0.614 | -0.289 | 1.000 |
|  |  | Indirect effect | -0.103 | -0.208 | -0.012 | 1.000 |
|  |  | Total effect | -0.557 | -0.736 | -0.377 | 1.000 |
|  |  | Indirect effect (%) | 18.381 | 2.446 | 34.950 | 1.000 |
| 38 | Ehime | a | -0.168 | -0.235 | -0.102 | 1.000 |
|  |  | b | -0.032 | -0.414 | 0.332 | 1.000 |
|  |  | c (Direct effect) | -0.404 | -0.592 | -0.219 | 1.000 |
|  |  | Indirect effect | 0.005 | -0.058 | 0.072 | 1.000 |
|  |  | Total effect | -0.399 | -0.590 | -0.211 | 1.000 |
|  |  | Indirect effect (%) | -2.117 | -22.971 | 14.629 | 1.000 |
| 39 | Kochi | a | -0.238 | -0.400 | -0.072 | 1.000 |
|  |  | b | 0.598 | 0.398 | 0.801 | 1.001 |
|  |  | c (Direct effect) | -0.306 | -0.574 | -0.036 | 1.000 |
|  |  | Indirect effect | -0.142 | -0.260 | -0.040 | 1.000 |
|  |  | Total effect | -0.448 | -0.725 | -0.169 | 1.000 |
|  |  | Indirect effect (%) | 33.084 | 9.740 | 80.349 | 1.000 |
| 40 | Fukuoka | a | -0.075 | -0.112 | -0.039 | 1.002 |
|  |  | b | 0.423 | 0.170 | 0.669 | 1.000 |
|  |  | c (Direct effect) | -0.256 | -0.338 | -0.169 | 1.006 |
|  |  | Indirect effect | -0.032 | -0.059 | -0.010 | 1.001 |
|  |  | Total effect | -0.288 | -0.370 | -0.201 | 1.008 |
|  |  | Indirect effect (%) | 11.215 | 3.600 | 21.692 | 1.001 |
| 41 | Saga | a | -0.044 | -0.068 | -0.019 | 1.000 |
|  |  | b | 7.064 | 4.750 | 9.673 | 1.004 |
|  |  | c (Direct effect) | -0.109 | -0.379 | 0.183 | 1.001 |
|  |  | Indirect effect | -0.308 | -0.527 | -0.124 | 1.001 |
|  |  | Total effect | -0.417 | -0.655 | -0.173 | 1.000 |
|  |  | Indirect effect (%) | 81.133 | 30.240 | 182.086 | 1.000 |
| 42 | Nagasaki | a | -0.230 | -0.344 | -0.116 | 1.000 |
|  |  | b | 0.807 | 0.455 | 1.177 | 1.001 |
|  |  | c (Direct effect) | -0.052 | -0.316 | 0.213 | 1.000 |
|  |  | Indirect effect | -0.186 | -0.324 | -0.076 | 1.000 |
|  |  | Total effect | -0.238 | -0.492 | 0.015 | 1.000 |
|  |  | Indirect effect (%) | 64.515 | -193.638 | 501.214 | 1.000 |
| 43 | Kumamoto | a | -0.039 | -0.098 | 0.020 | 1.000 |
|  |  | b | 1.573 | 1.099 | 2.091 | 1.002 |
|  |  | c (Direct effect) | -0.221 | -0.357 | -0.079 | 1.000 |
|  |  | Indirect effect | -0.062 | -0.166 | 0.030 | 1.000 |
|  |  | Total effect | -0.284 | -0.430 | -0.135 | 1.000 |
|  |  | Indirect effect (%) | 21.297 | -14.503 | 58.726 | 1.000 |
| 44 | Oita | a | - | - | - | - |
|  |  | b | - | - | - | - |
|  |  | c (Direct effect) | - | - | - | - |
|  |  | Indirect effect | - | - | - | - |
|  |  | Total effect | - | - | - | - |
|  |  | Indirect effect (%) | - | - | - | - |
| 45 | Miyazaki | a | -0.119 | -0.202 | -0.033 | 1.000 |
|  |  | b | 1.691 | 0.996 | 2.502 | 1.001 |
|  |  | c (Direct effect) | -0.214 | -0.500 | 0.079 | 1.000 |
|  |  | Indirect effect | -0.204 | -0.407 | -0.047 | 1.000 |
|  |  | Total effect | -0.418 | -0.692 | -0.140 | 1.000 |
|  |  | Indirect effect (%) | 55.415 | 12.079 | 136.801 | 1.000 |
| 46 | Kagoshima | a | -0.154 | -0.251 | -0.059 | 1.000 |
|  |  | b | 0.522 | 0.039 | 0.912 | 1.003 |
|  |  | c (Direct effect) | -0.440 | -0.635 | -0.250 | 1.000 |
|  |  | Indirect effect | -0.080 | -0.169 | -0.004 | 1.002 |
|  |  | Total effect | -0.520 | -0.717 | -0.318 | 1.000 |
|  |  | Indirect effect (%) | 15.497 | 0.913 | 32.025 | 1.001 |
| 47 | Okinawa | a | 0.121 | -0.028 | 0.269 | 1.012 |
|  |  | b | 0.238 | 0.126 | 0.367 | 1.012 |
|  |  | c (Direct effect) | -0.066 | -0.191 | 0.056 | 1.003 |
|  |  | Indirect effect | 0.028 | -0.007 | 0.069 | 1.005 |
|  |  | Total effect | -0.038 | -0.164 | 0.089 | 1.002 |
|  |  | Indirect effect (%) | 65.560 | -727.361 | 728.095 | 1.000 |

Prefectures where NPIs were not implemented are represented by "-".

**Table S9. Effects of NPIs on mitigating the COVID-19 infection mediated by visitors’ mobility during Phase 3**

| **Prefecture Number** | **Prefecture Name** | **Parameters** | **Mean** | **95% Credible Interval** | | **Rhat** |
| --- | --- | --- | --- | --- | --- | --- |
|  |  |  |  | **Low** | **High** |  |
| 1 | Hokkaido | a | -0.173 | -0.334 | -0.011 | 1.001 |
|  |  | b | 0.108 | -0.004 | 0.270 | 1.005 |
|  |  | c (Direct effect) | -0.184 | -0.312 | -0.055 | 1.003 |
|  |  | Indirect effect | -0.019 | -0.067 | 0.002 | 1.004 |
|  |  | Total effect | -0.204 | -0.328 | -0.079 | 1.002 |
|  |  | Indirect effect (%) | 7.547 | -1.194 | 41.666 | 1.000 |
| 2 | Aomori | a | -0.004 | -0.233 | 0.216 | 1.006 |
|  |  | b | 0.554 | 0.478 | 0.643 | 1.017 |
|  |  | c (Direct effect) | -0.060 | -0.223 | 0.096 | 1.004 |
|  |  | Indirect effect | -0.003 | -0.130 | 0.121 | 1.007 |
|  |  | Total effect | -0.062 | -0.265 | 0.141 | 1.007 |
|  |  | Indirect effect (%) | -58.880 | -484.723 | 500.255 | 1.000 |
| 3 | Iwate | a | - | - | - | - |
|  |  | b | - | - | - | - |
|  |  | c (Direct effect) | - | - | - | - |
|  |  | Indirect effect | - | - | - | - |
|  |  | Total effect | - | - | - | - |
|  |  | Indirect effect (%) | - | - | - | - |
| 4 | Miyagi | a | - | - | - | - |
|  |  | b | - | - | - | - |
|  |  | c (Direct effect) | - | - | - | - |
|  |  | Indirect effect | - | - | - | - |
|  |  | Total effect | - | - | - | - |
|  |  | Indirect effect (%) | - | - | - | - |
| 5 | Akita | a | - | - | - | - |
| 5 | Akita | b | - | - | - | - |
|  |  | c (Direct effect) | - | - | - | - |
|  |  | Indirect effect | - | - | - | - |
|  |  | Total effect | - | - | - | - |
|  |  | Indirect effect (%) | - | - | - | - |
| 6 | Yamagata | a | 0.166 | -0.060 | 0.388 | 1.003 |
|  |  | b | 0.604 | 0.504 | 0.742 | 1.008 |
|  |  | c (Direct effect) | -0.296 | -0.458 | -0.134 | 1.001 |
|  |  | Indirect effect | 0.101 | -0.036 | 0.244 | 1.004 |
|  |  | Total effect | -0.195 | -0.393 | 0.004 | 1.002 |
|  |  | Indirect effect (%) | -84.282 | -585.024 | 212.674 | 1.000 |
| 7 | Fukushima | a | -0.332 | -0.446 | -0.222 | 1.011 |
|  |  | b | 6.780 | 0.564 | 14.545 | 1.490 |
|  |  | c (Direct effect) | 1.902 | -0.154 | 4.756 | 1.391 |
|  |  | Indirect effect | -2.232 | -5.090 | -0.177 | 1.394 |
|  |  | Total effect | -0.331 | -0.502 | -0.161 | 1.004 |
|  |  | Indirect effect (%) | 745.917 | 52.897 | 1925.428 | 1.001 |
| 8 | Ibaraki | a | -0.048 | -0.126 | 0.031 | 1.000 |
|  |  | b | 2.242 | 1.739 | 2.862 | 1.005 |
|  |  | c (Direct effect) | -0.056 | -0.219 | 0.128 | 1.001 |
|  |  | Indirect effect | -0.111 | -0.307 | 0.065 | 1.000 |
|  |  | Total effect | -0.167 | -0.346 | 0.010 | 1.001 |
|  |  | Indirect effect (%) | 4.006 | -148.190 | 325.376 | 1.000 |
| 9 | Tochigi | a | -0.164 | -0.211 | -0.117 | 1.000 |
|  |  | b | 63.433 | -24.817 | 145.006 | 1.825 |
|  |  | c (Direct effect) | 10.200 | -3.971 | 25.777 | 1.686 |
|  |  | Indirect effect | -10.448 | -26.025 | 3.723 | 1.687 |
|  |  | Total effect | -0.248 | -0.365 | -0.129 | 1.000 |
|  |  | Indirect effect (%) | 4544.383 | -1482.971 | 13047.428 | 1.160 |
| 10 | Gunma | a | -0.148 | -0.193 | -0.109 | 1.070 |
|  |  | b | 11.635 | 4.859 | 19.105 | 1.282 |
|  |  | c (Direct effect) | 1.572 | 0.602 | 2.654 | 1.199 |
|  |  | Indirect effect | -1.706 | -2.826 | -0.771 | 1.181 |
|  |  | Total effect | -0.134 | -0.270 | -0.007 | 1.624 |
|  |  | Indirect effect (%) | 6017.370 | 376.622 | 18497.983 | 1.000 |
| 11 | Saitama | a | -0.027 | -0.060 | 0.006 | 1.004 |
|  |  | b | 3.159 | 2.489 | 4.130 | 1.020 |
|  |  | c (Direct effect) | -0.190 | -0.313 | -0.047 | 1.007 |
|  |  | Indirect effect | -0.088 | -0.218 | 0.017 | 1.005 |
|  |  | Total effect | -0.278 | -0.410 | -0.141 | 1.005 |
|  |  | Indirect effect (%) | 31.112 | -8.301 | 77.041 | 1.006 |
| 12 | Chiba | a | -0.099 | -0.145 | -0.039 | 1.042 |
|  |  | b | 2.955 | 1.821 | 4.305 | 1.082 |
|  |  | c (Direct effect) | -0.017 | -0.254 | 0.269 | 1.069 |
|  |  | Indirect effect | -0.300 | -0.585 | -0.078 | 1.035 |
|  |  | Total effect | -0.316 | -0.456 | -0.180 | 1.083 |
|  |  | Indirect effect (%) | 98.885 | 26.304 | 200.060 | 1.063 |
| 13 | Tokyo | a | 0.013 | -0.028 | 0.053 | 1.001 |
|  |  | b | -1.765 | -2.027 | -1.527 | 1.007 |
|  |  | c (Direct effect) | -0.278 | -0.363 | -0.194 | 1.003 |
|  |  | Indirect effect | -0.023 | -0.094 | 0.051 | 1.001 |
|  |  | Total effect | -0.301 | -0.410 | -0.191 | 1.002 |
|  |  | Indirect effect (%) | 6.292 | -22.605 | 26.897 | 1.001 |
| 14 | Kanagawa | a | -0.018 | -0.053 | 0.015 | 1.005 |
|  |  | b | 1.911 | 1.433 | 2.481 | 1.070 |
|  |  | c (Direct effect) | -0.292 | -0.417 | -0.162 | 1.002 |
|  |  | Indirect effect | -0.036 | -0.110 | 0.027 | 1.010 |
|  |  | Total effect | -0.328 | -0.463 | -0.191 | 1.000 |
|  |  | Indirect effect (%) | 10.606 | -10.636 | 33.256 | 1.008 |
| 15 | Niigata | a | -0.008 | -0.254 | 0.244 | 1.000 |
|  |  | b | 0.289 | 0.224 | 0.366 | 1.004 |
|  |  | c (Direct effect) | -0.343 | -0.477 | -0.208 | 1.000 |
|  |  | Indirect effect | -0.003 | -0.076 | 0.070 | 1.000 |
|  |  | Total effect | -0.346 | -0.496 | -0.195 | 1.000 |
|  |  | Indirect effect (%) | -0.453 | -27.773 | 19.904 | 1.000 |
| 16 | Toyama | a | - | - | - | - |
|  |  | b | - | - | - | - |
|  |  | c (Direct effect) | - | - | - | - |
|  |  | Indirect effect | - | - | - | - |
|  |  | Total effect | - | - | - | - |
|  |  | Indirect effect (%) | - | - | - | - |
| 17 | Ishikawa | a | -0.288 | -0.404 | -0.162 | 1.000 |
|  |  | b | -0.715 | -1.191 | -0.321 | 1.001 |
|  |  | c (Direct effect) | -0.722 | -0.938 | -0.521 | 1.000 |
|  |  | Indirect effect | 0.208 | 0.071 | 0.396 | 1.000 |
|  |  | Total effect | -0.514 | -0.671 | -0.358 | 1.000 |
|  |  | Indirect effect (%) | -41.870 | -89.729 | -13.306 | 1.000 |
| 18 | Fukui | a | - | - | - | - |
|  |  | b | - | - | - | - |
|  |  | c (Direct effect) | - | - | - | - |
|  |  | Indirect effect | - | - | - | - |
|  |  | Total effect | - | - | - | - |
|  |  | Indirect effect (%) | - | - | - | - |
| 19 | Yamanashi | a | - | - | - | - |
|  |  | b | - | - | - | - |
|  |  | c (Direct effect) | - | - | - | - |
|  |  | Indirect effect | - | - | - | - |
|  |  | Total effect | - | - | - | - |
|  |  | Indirect effect (%) | - | - | - | - |
| 20 | Nagano | a | -0.042 | -0.294 | 0.211 | 1.000 |
|  |  | b | 0.243 | 0.193 | 0.297 | 1.002 |
|  |  | c (Direct effect) | -0.207 | -0.326 | -0.086 | 1.000 |
|  |  | Indirect effect | -0.010 | -0.073 | 0.052 | 1.000 |
|  |  | Total effect | -0.217 | -0.350 | -0.084 | 1.000 |
|  |  | Indirect effect (%) | 2.428 | -40.328 | 31.720 | 1.000 |
| 21 | Gifu | a | -0.165 | -0.215 | -0.105 | 1.005 |
|  |  | b | 8.327 | 4.038 | 12.504 | 1.017 |
|  |  | c (Direct effect) | 1.048 | 0.170 | 1.841 | 1.017 |
|  |  | Indirect effect | -1.388 | -2.237 | -0.477 | 1.015 |
|  |  | Total effect | -0.340 | -0.587 | -0.086 | 1.000 |
|  |  | Indirect effect (%) | 488.516 | 141.001 | 1299.271 | 1.000 |
| 22 | Shizuoka | a | -0.098 | -0.255 | 0.060 | 1.005 |
|  |  | b | 0.801 | 0.670 | 0.950 | 1.009 |
|  |  | c (Direct effect) | -0.196 | -0.330 | -0.059 | 1.002 |
|  |  | Indirect effect | -0.078 | -0.204 | 0.048 | 1.005 |
|  |  | Total effect | -0.274 | -0.447 | -0.096 | 1.002 |
|  |  | Indirect effect (%) | 51.274 | -32.519 | 66.305 | 1.000 |
| 23 | Aichi | a | -0.015 | -0.069 | 0.037 | 1.003 |
|  |  | b | 1.511 | 1.120 | 1.963 | 1.018 |
|  |  | c (Direct effect) | -0.305 | -0.437 | -0.172 | 1.003 |
|  |  | Indirect effect | -0.024 | -0.117 | 0.051 | 1.003 |
|  |  | Total effect | -0.329 | -0.460 | -0.199 | 1.001 |
|  |  | Indirect effect (%) | 6.701 | -19.188 | 34.628 | 1.003 |
| 24 | Mie | a | -0.246 | -0.353 | -0.099 | 1.345 |
|  |  | b | 3.872 | 1.279 | 6.444 | 2.187 |
|  |  | c (Direct effect) | 0.832 | -0.053 | 1.781 | 2.040 |
|  |  | Indirect effect | -1.037 | -2.006 | -0.141 | 2.033 |
|  |  | Total effect | -0.205 | -0.348 | -0.048 | 1.019 |
|  |  | Indirect effect (%) | 489.963 | 71.437 | 1774.032 | 1.000 |
| 25 | Shiga | a | - | - | - | - |
|  |  | b | - | - | - | - |
|  |  | c (Direct effect) | - | - | - | - |
|  |  | Indirect effect | - | - | - | - |
|  |  | Total effect | - | - | - | - |
|  |  | Indirect effect (%) | - | - | - | - |
| 26 | Kyoto | a | -0.076 | -0.130 | -0.021 | 1.003 |
|  |  | b | 0.638 | 0.336 | 0.970 | 1.012 |
|  |  | c (Direct effect) | -0.230 | -0.335 | -0.126 | 1.008 |
|  |  | Indirect effect | -0.049 | -0.101 | -0.011 | 1.010 |
|  |  | Total effect | -0.279 | -0.381 | -0.187 | 1.004 |
|  |  | Indirect effect (%) | 18.026 | 3.946 | 38.118 | 1.018 |
| 27 | Osaka | a | -0.034 | -0.060 | -0.007 | 1.002 |
|  |  | b | 0.871 | 0.418 | 1.377 | 1.017 |
|  |  | c (Direct effect) | -0.219 | -0.331 | -0.110 | 1.003 |
|  |  | Indirect effect | -0.030 | -0.064 | -0.005 | 1.010 |
|  |  | Total effect | -0.249 | -0.362 | -0.141 | 1.002 |
|  |  | Indirect effect (%) | 12.572 | 2.071 | 28.768 | 1.007 |
| 28 | Hyogo | a | -0.155 | -0.191 | -0.118 | 1.021 |
|  |  | b | 17.586 | 3.611 | 39.826 | 1.502 |
|  |  | c (Direct effect) | 2.440 | 0.227 | 5.798 | 1.430 |
|  |  | Indirect effect | -2.721 | -6.091 | -0.504 | 1.430 |
|  |  | Total effect | -0.281 | -0.415 | -0.149 | 1.000 |
|  |  | Indirect effect (%) | 1033.859 | 183.624 | 2530.186 | 1.203 |
| 29 | Nara | a | - | - | - | - |
|  |  | b | - | - | - | - |
|  |  | c (Direct effect) | - | - | - | - |
|  |  | Indirect effect | - | - | - | - |
|  |  | Total effect | - | - | - | - |
|  |  | Indirect effect (%) | - | - | - | - |
| 30 | Wakayama | a | -0.026 | -0.154 | 0.106 | 1.000 |
|  |  | b | 1.226 | 0.916 | 1.589 | 1.001 |
|  |  | c (Direct effect) | -0.043 | -0.204 | 0.124 | 1.000 |
|  |  | Indirect effect | -0.033 | -0.201 | 0.128 | 1.000 |
|  |  | Total effect | -0.077 | -0.232 | 0.080 | 1.000 |
|  |  | Indirect effect (%) | 60.800 | -678.415 | 814.288 | 1.000 |
| 31 | Tottori | a | - | - | - | - |
|  |  | b | - | - | - | - |
|  |  | c (Direct effect) | - | - | - | - |
|  |  | Indirect effect | - | - | - | - |
|  |  | Total effect | - | - | - | - |
|  |  | Indirect effect (%) | - | - | - | - |
| 32 | Shimane | a | 0.023 | -0.105 | 0.152 | 1.000 |
|  |  | b | 0.940 | 0.474 | 1.514 | 1.001 |
|  |  | c (Direct effect) | -0.373 | -0.595 | -0.141 | 1.000 |
|  |  | Indirect effect | 0.019 | -0.113 | 0.145 | 1.000 |
|  |  | Total effect | -0.354 | -0.573 | -0.136 | 1.000 |
|  |  | Indirect effect (%) | -6.511 | -65.454 | 34.663 | 1.000 |
| 33 | Okayama | a | 0.001 | -0.112 | 0.120 | 1.009 |
|  |  | b | 1.378 | 1.152 | 1.688 | 1.056 |
|  |  | c (Direct effect) | -0.092 | -0.265 | 0.062 | 1.038 |
|  |  | Indirect effect | 0.003 | -0.154 | 0.170 | 1.007 |
|  |  | Total effect | -0.090 | -0.308 | 0.118 | 1.009 |
|  |  | Indirect effect (%) | 58.373 | -582.444 | 633.992 | 1.000 |
| 34 | Hiroshima | a | -0.442 | -0.558 | -0.331 | 1.014 |
|  |  | b | 1.232 | 0.827 | 1.700 | 1.071 |
|  |  | c (Direct effect) | -0.215 | -0.443 | 0.014 | 1.033 |
|  |  | Indirect effect | -0.540 | -0.745 | -0.363 | 1.036 |
|  |  | Total effect | -0.755 | -0.933 | -0.577 | 1.007 |
|  |  | Indirect effect (%) | 72.213 | 47.401 | 102.176 | 1.038 |
| 35 | Yamaguchi | a | -0.277 | -0.401 | -0.161 | 1.001 |
|  |  | b | 0.956 | 0.634 | 1.343 | 1.024 |
|  |  | c (Direct effect) | -0.093 | -0.232 | 0.055 | 1.006 |
|  |  | Indirect effect | -0.261 | -0.397 | -0.146 | 1.006 |
|  |  | Total effect | -0.354 | -0.475 | -0.234 | 1.000 |
|  |  | Indirect effect (%) | 75.107 | 42.292 | 119.411 | 1.006 |
| 36 | Tokushima | a | - | - | - | - |
|  |  | b | - | - | - | - |
|  |  | c (Direct effect) | - | - | - | - |
|  |  | Indirect effect | - | - | - | - |
|  |  | Total effect | - | - | - | - |
|  |  | Indirect effect (%) | - | - | - | - |
| 37 | Kagawa | a | -0.095 | -0.260 | 0.072 | 1.003 |
|  |  | b | 0.570 | 0.458 | 0.723 | 1.008 |
|  |  | c (Direct effect) | -0.127 | -0.244 | -0.007 | 1.002 |
|  |  | Indirect effect | -0.055 | -0.158 | 0.040 | 1.004 |
|  |  | Total effect | -0.182 | -0.320 | -0.045 | 1.004 |
|  |  | Indirect effect (%) | 21.755 | -42.591 | 92.126 | 1.000 |
| 38 | Ehime | a | - | - | - | - |
|  |  | b | - | - | - | - |
|  |  | c (Direct effect) | - | - | - | - |
|  |  | Indirect effect | - | - | - | - |
|  |  | Total effect | - | - | - | - |
|  |  | Indirect effect (%) | - | - | - | - |
| 39 | Kochi | a | -0.064 | -0.273 | 0.155 | 1.001 |
|  |  | b | 0.550 | 0.436 | 0.664 | 1.006 |
|  |  | c (Direct effect) | 0.015 | -0.105 | 0.135 | 1.000 |
|  |  | Indirect effect | -0.036 | -0.160 | 0.084 | 1.002 |
|  |  | Total effect | -0.021 | -0.161 | 0.124 | 1.001 |
|  |  | Indirect effect (%) | 109.318 | -831.555 | 963.213 | 1.000 |
| 40 | Fukuoka | a | -0.010 | -0.103 | 0.083 | 1.000 |
|  |  | b | 0.613 | 0.407 | 0.854 | 1.002 |
|  |  | c (Direct effect) | -0.305 | -0.465 | -0.145 | 1.001 |
|  |  | Indirect effect | -0.006 | -0.065 | 0.052 | 1.000 |
|  |  | Total effect | -0.311 | -0.477 | -0.144 | 1.001 |
|  |  | Indirect effect (%) | 1.032 | -24.444 | 20.776 | 1.000 |
| 41 | Saga | a | -0.044 | -0.097 | 0.010 | 1.009 |
|  |  | b | 4.284 | 3.253 | 6.013 | 1.068 |
|  |  | c (Direct effect) | 0.154 | -0.058 | 0.463 | 1.047 |
|  |  | Indirect effect | -0.198 | -0.524 | 0.037 | 1.042 |
|  |  | Total effect | -0.044 | -0.211 | 0.124 | 1.000 |
|  |  | Indirect effect (%) | -1061.044 | -2625.784 | 2788.221 | 1.000 |
| 42 | Nagasaki | a | -0.169 | -0.354 | 0.021 | 1.000 |
|  |  | b | 0.730 | 0.548 | 0.957 | 1.001 |
|  |  | c (Direct effect) | -0.192 | -0.362 | -0.011 | 1.000 |
|  |  | Indirect effect | -0.126 | -0.290 | 0.014 | 1.000 |
|  |  | Total effect | -0.318 | -0.499 | -0.136 | 1.000 |
|  |  | Indirect effect (%) | 35.529 | -6.419 | 94.545 | 1.000 |
| 43 | Kumamoto | a | -0.099 | -0.235 | 0.041 | 1.000 |
|  |  | b | 1.667 | 1.357 | 2.000 | 1.003 |
|  |  | c (Direct effect) | -0.295 | -0.514 | -0.070 | 1.001 |
|  |  | Indirect effect | -0.168 | -0.417 | 0.066 | 1.000 |
|  |  | Total effect | -0.462 | -0.739 | -0.188 | 1.000 |
|  |  | Indirect effect (%) | 34.046 | -24.188 | 80.550 | 1.000 |
| 44 | Oita | a | 0.015 | -0.131 | 0.163 | 1.008 |
|  |  | b | 1.425 | 1.171 | 1.707 | 1.001 |
|  |  | c (Direct effect) | -0.252 | -0.428 | -0.066 | 1.002 |
|  |  | Indirect effect | 0.020 | -0.189 | 0.233 | 1.008 |
|  |  | Total effect | -0.232 | -0.465 | -0.009 | 1.003 |
|  |  | Indirect effect (%) | -258.652 | -446.802 | 85.156 | 1.000 |
| 45 | Miyazaki | a | -0.058 | -0.205 | 0.081 | 1.018 |
|  |  | b | 1.061 | 0.921 | 1.236 | 1.019 |
|  |  | c (Direct effect) | -0.299 | -0.454 | -0.149 | 1.002 |
|  |  | Indirect effect | -0.062 | -0.221 | 0.085 | 1.017 |
|  |  | Total effect | -0.361 | -0.570 | -0.161 | 1.012 |
|  |  | Indirect effect (%) | 14.136 | -43.482 | 49.712 | 1.000 |
| 46 | Kagoshima | a | -0.049 | -0.214 | 0.115 | 1.000 |
|  |  | b | 0.827 | 0.689 | 0.966 | 1.000 |
|  |  | c (Direct effect) | -0.243 | -0.415 | -0.066 | 1.000 |
|  |  | Indirect effect | -0.040 | -0.178 | 0.096 | 1.000 |
|  |  | Total effect | -0.283 | -0.502 | -0.059 | 1.000 |
|  |  | Indirect effect (%) | 13.026 | -84.502 | 58.096 | 1.000 |
| 47 | Okinawa | a | -0.632 | -0.793 | -0.468 | 1.002 |
|  |  | b | 0.693 | 0.575 | 0.848 | 1.005 |
|  |  | c (Direct effect) | -0.356 | -0.506 | -0.192 | 1.004 |
|  |  | Indirect effect | -0.439 | -0.617 | -0.298 | 1.005 |
|  |  | Total effect | -0.796 | -0.951 | -0.639 | 1.002 |
|  |  | Indirect effect (%) | 55.275 | 40.024 | 74.426 | 1.005 |

Prefectures where NPIs were not implemented are represented by "-".

**Table S10. Effects of NPIs on mitigating the COVID-19 infection mediated by Tokyo residents’ mobility during Phase 1**

| **Prefecture Number** | **Prefecture Name** | **Parameters** | **Mean** | **95% Credible Interval** | | **Rhat** |
| --- | --- | --- | --- | --- | --- | --- |
|  |  |  |  | **Low** | **High** |  |
| 1 | Hokkaido | a | 0.071 | -0.071 | 0.213 | 1.000 |
|  |  | b | 0.263 | 0.168 | 0.361 | 1.000 |
|  |  | c (Direct effect) | -0.343 | -0.469 | -0.221 | 1.001 |
|  |  | Indirect effect | 0.019 | -0.018 | 0.060 | 1.000 |
|  |  | Total effect | -0.325 | -0.454 | -0.198 | 1.001 |
|  |  | Indirect effect (%) | -6.512 | -23.912 | 5.335 | 1.000 |
| 2 | Aomori | a | 0.194 | -0.005 | 0.399 | 1.002 |
|  |  | b | 0.168 | 0.021 | 0.315 | 1.000 |
|  |  | c (Direct effect) | -0.048 | -0.312 | 0.213 | 1.001 |
|  |  | Indirect effect | 0.033 | -0.003 | 0.088 | 1.002 |
|  |  | Total effect | -0.015 | -0.282 | 0.249 | 1.000 |
|  |  | Indirect effect (%) | 173.233 | -380.377 | 388.570 | 1.000 |
| 3 | Iwate | a | 0.130 | -0.065 | 0.325 | 1.001 |
|  |  | b | 0.088 | -0.020 | 0.196 | 1.000 |
|  |  | c (Direct effect) | -0.117 | -0.299 | 0.066 | 1.001 |
|  |  | Indirect effect | 0.012 | -0.007 | 0.042 | 1.000 |
|  |  | Total effect | -0.106 | -0.286 | 0.078 | 1.001 |
|  |  | Indirect effect (%) | -41.067 | -130.944 | 100.308 | 1.000 |
| 4 | Miyagi | a | 0.114 | -0.022 | 0.248 | 1.000 |
|  |  | b | 0.167 | -0.023 | 0.366 | 1.000 |
|  |  | c (Direct effect) | -0.747 | -0.939 | -0.558 | 1.000 |
|  |  | Indirect effect | 0.019 | -0.006 | 0.062 | 1.000 |
|  |  | Total effect | -0.727 | -0.919 | -0.538 | 1.000 |
|  |  | Indirect effect (%) | -2.708 | -9.126 | 0.824 | 1.000 |
| 5 | Akita | a | 0.186 | -0.015 | 0.387 | 1.001 |
|  |  | b | 0.101 | -0.053 | 0.256 | 1.001 |
|  |  | c (Direct effect) | -0.380 | -0.650 | -0.110 | 1.000 |
|  |  | Indirect effect | 0.019 | -0.011 | 0.066 | 1.001 |
|  |  | Total effect | -0.361 | -0.632 | -0.089 | 1.000 |
|  |  | Indirect effect (%) | -12.912 | -33.175 | 3.557 | 1.000 |
| 6 | Yamagata | a | 0.072 | -0.098 | 0.231 | 1.027 |
|  |  | b | 0.272 | 0.142 | 0.409 | 1.034 |
|  |  | c (Direct effect) | -0.767 | -1.003 | -0.537 | 1.018 |
|  |  | Indirect effect | 0.019 | -0.027 | 0.070 | 1.006 |
|  |  | Total effect | -0.748 | -0.989 | -0.510 | 1.021 |
|  |  | Indirect effect (%) | -2.769 | -10.520 | 3.539 | 1.010 |
| 7 | Fukushima | a | 0.106 | -0.058 | 0.273 | 1.000 |
|  |  | b | 0.466 | 0.318 | 0.624 | 1.001 |
|  |  | c (Direct effect) | -0.723 | -0.916 | -0.531 | 1.000 |
|  |  | Indirect effect | 0.050 | -0.026 | 0.136 | 1.000 |
|  |  | Total effect | -0.673 | -0.877 | -0.473 | 1.000 |
|  |  | Indirect effect (%) | -7.935 | -24.044 | 3.543 | 1.000 |
| 8 | Ibaraki | a | 0.103 | -0.005 | 0.215 | 1.000 |
|  |  | b | 1.182 | 0.788 | 1.663 | 1.000 |
|  |  | c (Direct effect) | -0.771 | -0.943 | -0.598 | 1.000 |
|  |  | Indirect effect | 0.123 | -0.006 | 0.275 | 1.000 |
|  |  | Total effect | -0.648 | -0.830 | -0.462 | 1.000 |
|  |  | Indirect effect (%) | -20.175 | -52.355 | 0.823 | 1.000 |
| 9 | Tochigi | a | -0.170 | -0.282 | -0.054 | 1.000 |
|  |  | b | -0.015 | -0.252 | 0.224 | 1.002 |
|  |  | c (Direct effect) | -0.386 | -0.548 | -0.224 | 1.001 |
|  |  | Indirect effect | 0.003 | -0.041 | 0.049 | 1.001 |
|  |  | Total effect | -0.383 | -0.534 | -0.232 | 1.001 |
|  |  | Indirect effect (%) | -0.628 | -13.139 | 11.943 | 1.001 |
| 10 | Gunma | a | 0.086 | -0.078 | 0.252 | 1.000 |
|  |  | b | 0.102 | -0.113 | 0.327 | 1.001 |
|  |  | c (Direct effect) | -0.427 | -0.648 | -0.209 | 1.001 |
|  |  | Indirect effect | 0.009 | -0.016 | 0.049 | 1.000 |
|  |  | Total effect | -0.418 | -0.638 | -0.197 | 1.001 |
|  |  | Indirect effect (%) | -2.465 | -14.501 | 3.955 | 1.000 |
| 11 | Saitama | a | -0.086 | -0.100 | -0.072 | 1.000 |
|  |  | b | 5.315 | 3.236 | 7.642 | 1.000 |
|  |  | c (Direct effect) | 0.038 | -0.182 | 0.286 | 1.000 |
|  |  | Indirect effect | -0.458 | -0.684 | -0.271 | 1.000 |
|  |  | Total effect | -0.421 | -0.525 | -0.314 | 1.000 |
|  |  | Indirect effect (%) | 110.917 | 61.086 | 178.998 | 1.000 |
| 12 | Chiba | a | -0.161 | -0.191 | -0.130 | 1.000 |
|  |  | b | 0.985 | 0.129 | 1.887 | 1.000 |
|  |  | c (Direct effect) | -0.360 | -0.547 | -0.164 | 1.000 |
|  |  | Indirect effect | -0.158 | -0.304 | -0.021 | 1.000 |
|  |  | Total effect | -0.518 | -0.627 | -0.405 | 1.000 |
|  |  | Indirect effect (%) | 31.014 | 3.941 | 63.671 | 1.000 |
| 13 | Tokyo | a | 0.013 | 0.009 | 0.018 | 1.000 |
|  |  | b | -7.007 | -13.865 | -1.718 | 1.001 |
|  |  | c (Direct effect) | -0.412 | -0.523 | -0.286 | 1.001 |
|  |  | Indirect effect | -0.091 | -0.178 | -0.023 | 1.001 |
|  |  | Total effect | -0.503 | -0.591 | -0.409 | 1.001 |
|  |  | Indirect effect (%) | 18.167 | 4.549 | 36.505 | 1.001 |
| 14 | Kanagawa | a | -0.096 | -0.114 | -0.077 | 1.001 |
|  |  | b | 2.294 | 1.465 | 3.173 | 1.001 |
|  |  | c (Direct effect) | -0.024 | -0.167 | 0.129 | 1.001 |
|  |  | Indirect effect | -0.220 | -0.328 | -0.129 | 1.001 |
|  |  | Total effect | -0.244 | -0.343 | -0.141 | 1.000 |
|  |  | Indirect effect (%) | 96.914 | 46.112 | 180.529 | 1.000 |
| 15 | Niigata | a | 0.142 | -0.058 | 0.324 | 1.025 |
|  |  | b | 0.063 | -0.056 | 0.193 | 1.021 |
|  |  | c (Direct effect) | -0.343 | -0.533 | -0.132 | 1.044 |
|  |  | Indirect effect | 0.009 | -0.010 | 0.039 | 1.014 |
|  |  | Total effect | -0.334 | -0.525 | -0.122 | 1.045 |
|  |  | Indirect effect (%) | -2.963 | -17.511 | 3.176 | 1.000 |
| 16 | Toyama | a | 0.084 | -0.109 | 0.275 | 1.000 |
|  |  | b | 0.550 | 0.415 | 0.686 | 1.001 |
|  |  | c (Direct effect) | -0.674 | -0.926 | -0.430 | 1.000 |
|  |  | Indirect effect | 0.047 | -0.059 | 0.156 | 1.000 |
|  |  | Total effect | -0.628 | -0.898 | -0.363 | 1.000 |
|  |  | Indirect effect (%) | -8.698 | -34.148 | 8.486 | 1.000 |
| 17 | Ishikawa | a | 0.128 | -0.053 | 0.307 | 1.000 |
|  |  | b | 0.368 | 0.231 | 0.510 | 1.001 |
|  |  | c (Direct effect) | -0.619 | -0.840 | -0.400 | 1.000 |
|  |  | Indirect effect | 0.047 | -0.019 | 0.123 | 1.000 |
|  |  | Total effect | -0.571 | -0.801 | -0.343 | 1.000 |
|  |  | Indirect effect (%) | -9.075 | -27.406 | 3.013 | 1.000 |
| 18 | Fukui | a | 0.056 | -0.122 | 0.239 | 1.001 |
|  |  | b | 0.306 | 0.133 | 0.484 | 1.001 |
|  |  | c (Direct effect) | -0.684 | -0.926 | -0.441 | 1.000 |
|  |  | Indirect effect | 0.017 | -0.038 | 0.082 | 1.001 |
|  |  | Total effect | -0.666 | -0.915 | -0.417 | 1.000 |
|  |  | Indirect effect (%) | -2.939 | -14.805 | 5.623 | 1.001 |
| 19 | Yamanashi | a | 0.120 | -0.051 | 0.293 | 1.000 |
|  |  | b | 0.309 | 0.130 | 0.491 | 1.001 |
|  |  | c (Direct effect) | -0.334 | -0.577 | -0.102 | 1.000 |
|  |  | Indirect effect | 0.037 | -0.015 | 0.104 | 1.000 |
|  |  | Total effect | -0.297 | -0.540 | -0.059 | 1.000 |
|  |  | Indirect effect (%) | -16.876 | -83.819 | 6.087 | 1.000 |
| 20 | Nagano | a | 0.205 | -0.009 | 0.415 | 1.000 |
|  |  | b | 0.465 | 0.345 | 0.599 | 1.001 |
|  |  | c (Direct effect) | -0.642 | -0.836 | -0.446 | 1.000 |
|  |  | Indirect effect | 0.096 | -0.004 | 0.207 | 1.001 |
|  |  | Total effect | -0.546 | -0.762 | -0.333 | 1.000 |
|  |  | Indirect effect (%) | -19.314 | -51.494 | 0.591 | 1.000 |
| 21 | Gifu | a | -0.218 | -0.387 | -0.046 | 1.001 |
|  |  | b | -0.114 | -0.248 | 0.010 | 1.001 |
|  |  | c (Direct effect) | -0.575 | -0.693 | -0.455 | 1.001 |
|  |  | Indirect effect | 0.025 | -0.002 | 0.070 | 1.002 |
|  |  | Total effect | -0.550 | -0.667 | -0.431 | 1.001 |
|  |  | Indirect effect (%) | -4.688 | -13.706 | 0.438 | 1.002 |
| 22 | Shizuoka | a | 0.084 | -0.090 | 0.255 | 1.000 |
|  |  | b | 0.307 | 0.147 | 0.471 | 1.000 |
|  |  | c (Direct effect) | -0.725 | -0.952 | -0.494 | 1.000 |
|  |  | Indirect effect | 0.026 | -0.028 | 0.088 | 1.000 |
|  |  | Total effect | -0.699 | -0.932 | -0.463 | 1.000 |
|  |  | Indirect effect (%) | -3.954 | -14.530 | 3.876 | 1.000 |
| 23 | Aichi | a | -0.099 | -0.196 | -0.002 | 1.000 |
|  |  | b | 0.249 | -0.098 | 0.636 | 1.000 |
|  |  | c (Direct effect) | -0.035 | -0.259 | 0.194 | 1.000 |
|  |  | Indirect effect | -0.024 | -0.078 | 0.012 | 1.000 |
|  |  | Total effect | -0.058 | -0.280 | 0.168 | 1.000 |
|  |  | Indirect effect (%) | 10.350 | -307.006 | 309.908 | 1.000 |
| 24 | Mie | a | 0.106 | -0.079 | 0.291 | 1.000 |
|  |  | b | 0.350 | 0.217 | 0.487 | 1.000 |
|  |  | c (Direct effect) | -0.678 | -0.880 | -0.479 | 1.000 |
|  |  | Indirect effect | 0.037 | -0.027 | 0.110 | 1.000 |
|  |  | Total effect | -0.641 | -0.852 | -0.434 | 1.001 |
|  |  | Indirect effect (%) | -6.246 | -20.163 | 3.967 | 1.000 |
| 25 | Shiga | a | 0.184 | -0.014 | 0.381 | 1.000 |
|  |  | b | 0.273 | 0.103 | 0.476 | 1.001 |
|  |  | c (Direct effect) | -0.624 | -0.822 | -0.424 | 1.000 |
|  |  | Indirect effect | 0.051 | -0.003 | 0.134 | 1.000 |
|  |  | Total effect | -0.573 | -0.772 | -0.372 | 1.000 |
|  |  | Indirect effect (%) | -9.482 | -27.219 | 0.543 | 1.000 |
| 26 | Kyoto | a | -0.297 | -0.443 | -0.151 | 1.000 |
|  |  | b | 0.115 | -0.004 | 0.243 | 1.000 |
|  |  | c (Direct effect) | -0.605 | -0.729 | -0.478 | 1.000 |
|  |  | Indirect effect | -0.034 | -0.082 | 0.001 | 1.000 |
|  |  | Total effect | -0.639 | -0.751 | -0.526 | 1.000 |
|  |  | Indirect effect (%) | 5.459 | -0.190 | 13.452 | 1.000 |
| 27 | Osaka | a | -0.189 | -0.295 | -0.083 | 1.000 |
|  |  | b | 0.651 | 0.372 | 0.987 | 1.001 |
|  |  | c (Direct effect) | -0.152 | -0.288 | -0.010 | 1.000 |
|  |  | Indirect effect | -0.122 | -0.216 | -0.049 | 1.000 |
|  |  | Total effect | -0.274 | -0.404 | -0.144 | 1.000 |
|  |  | Indirect effect (%) | 46.816 | 18.238 | 94.073 | 1.000 |
| 28 | Hyogo | a | -0.232 | -0.379 | -0.083 | 1.000 |
|  |  | b | 0.139 | -0.004 | 0.310 | 1.001 |
|  |  | c (Direct effect) | -0.229 | -0.371 | -0.084 | 1.000 |
|  |  | Indirect effect | -0.032 | -0.083 | 0.001 | 1.001 |
|  |  | Total effect | -0.261 | -0.400 | -0.119 | 1.000 |
|  |  | Indirect effect (%) | 13.452 | -0.403 | 38.678 | 1.000 |
| 29 | Nara | a | 0.258 | 0.027 | 0.484 | 1.000 |
|  |  | b | 0.473 | 0.217 | 0.812 | 1.000 |
|  |  | c (Direct effect) | -0.803 | -1.011 | -0.599 | 1.000 |
|  |  | Indirect effect | 0.125 | 0.009 | 0.294 | 1.000 |
|  |  | Total effect | -0.679 | -0.868 | -0.484 | 1.000 |
|  |  | Indirect effect (%) | -19.262 | -50.592 | -1.267 | 1.000 |
| 30 | Wakayama | a | 0.217 | -0.025 | 0.454 | 1.002 |
|  |  | b | 0.238 | 0.115 | 0.361 | 1.001 |
|  |  | c (Direct effect) | -0.265 | -0.521 | -0.005 | 1.001 |
|  |  | Indirect effect | 0.052 | -0.006 | 0.124 | 1.002 |
|  |  | Total effect | -0.213 | -0.474 | 0.048 | 1.001 |
|  |  | Indirect effect (%) | -13.330 | -231.014 | 129.914 | 1.000 |
| 31 | Tottori | a | 0.251 | 0.036 | 0.475 | 1.000 |
|  |  | b | 0.095 | -0.010 | 0.199 | 1.001 |
|  |  | c (Direct effect) | -0.225 | -0.472 | 0.025 | 1.000 |
|  |  | Indirect effect | 0.024 | -0.003 | 0.067 | 1.000 |
|  |  | Total effect | -0.201 | -0.449 | 0.049 | 1.000 |
|  |  | Indirect effect (%) | -22.208 | -117.423 | 74.331 | 1.000 |
| 32 | Shimane | a | 0.127 | -0.068 | 0.324 | 1.022 |
|  |  | b | 0.114 | -0.035 | 0.267 | 1.007 |
|  |  | c (Direct effect) | -0.487 | -0.825 | -0.159 | 1.003 |
|  |  | Indirect effect | 0.015 | -0.010 | 0.058 | 1.018 |
|  |  | Total effect | -0.473 | -0.808 | -0.145 | 1.002 |
|  |  | Indirect effect (%) | -3.652 | -17.801 | 2.428 | 1.000 |
| 33 | Okayama | a | 0.179 | 0.004 | 0.352 | 1.001 |
|  |  | b | 0.203 | 0.072 | 0.338 | 1.001 |
|  |  | c (Direct effect) | -0.171 | -0.384 | 0.050 | 1.001 |
|  |  | Indirect effect | 0.036 | 0.000 | 0.087 | 1.000 |
|  |  | Total effect | -0.134 | -0.349 | 0.089 | 1.001 |
|  |  | Indirect effect (%) | -14.618 | -274.647 | 229.460 | 1.000 |
| 34 | Hiroshima | a | 0.185 | 0.035 | 0.330 | 1.005 |
|  |  | b | 0.249 | 0.063 | 0.451 | 1.009 |
|  |  | c (Direct effect) | -1.077 | -1.321 | -0.831 | 1.000 |
|  |  | Indirect effect | 0.046 | 0.004 | 0.109 | 1.001 |
|  |  | Total effect | -1.031 | -1.274 | -0.785 | 1.001 |
|  |  | Indirect effect (%) | -4.530 | -11.438 | -0.372 | 1.002 |
| 35 | Yamaguchi | a | 0.164 | -0.004 | 0.335 | 1.004 |
|  |  | b | 0.309 | 0.146 | 0.474 | 1.006 |
|  |  | c (Direct effect) | -0.353 | -0.622 | -0.092 | 1.007 |
|  |  | Indirect effect | 0.050 | -0.001 | 0.118 | 1.002 |
|  |  | Total effect | -0.303 | -0.576 | -0.036 | 1.007 |
|  |  | Indirect effect (%) | -28.629 | -112.867 | 2.400 | 1.000 |
| 36 | Tokushima | a | 0.248 | 0.039 | 0.451 | 1.001 |
|  |  | b | 0.255 | 0.127 | 0.384 | 1.003 |
|  |  | c (Direct effect) | -0.161 | -0.418 | 0.093 | 1.002 |
|  |  | Indirect effect | 0.063 | 0.009 | 0.135 | 1.001 |
|  |  | Total effect | -0.098 | -0.356 | 0.161 | 1.001 |
|  |  | Indirect effect (%) | -110.889 | -607.170 | 577.090 | 1.000 |
| 37 | Kagawa | a | 0.240 | 0.056 | 0.425 | 1.001 |
|  |  | b | 0.165 | 0.038 | 0.294 | 1.002 |
|  |  | c (Direct effect) | -0.676 | -0.934 | -0.427 | 1.004 |
|  |  | Indirect effect | 0.040 | 0.004 | 0.093 | 1.000 |
|  |  | Total effect | -0.636 | -0.892 | -0.385 | 1.003 |
|  |  | Indirect effect (%) | -6.646 | -17.434 | -0.613 | 1.000 |
| 38 | Ehime | a | 0.225 | 0.049 | 0.402 | 1.001 |
|  |  | b | 0.259 | 0.124 | 0.396 | 1.001 |
|  |  | c (Direct effect) | 0.218 | -0.045 | 0.480 | 1.000 |
|  |  | Indirect effect | 0.058 | 0.010 | 0.123 | 1.001 |
|  |  | Total effect | 0.276 | 0.008 | 0.541 | 1.000 |
|  |  | Indirect effect (%) | 25.747 | -1.738 | 117.710 | 1.000 |
| 39 | Kochi | a | 0.184 | -0.012 | 0.380 | 1.001 |
|  |  | b | 0.319 | 0.193 | 0.448 | 1.008 |
|  |  | c (Direct effect) | -0.675 | -0.938 | -0.428 | 1.003 |
|  |  | Indirect effect | 0.058 | -0.004 | 0.133 | 1.000 |
|  |  | Total effect | -0.617 | -0.887 | -0.362 | 1.003 |
|  |  | Indirect effect (%) | -10.322 | -27.660 | 0.562 | 1.000 |
| 40 | Fukuoka | a | -0.108 | -0.223 | 0.010 | 1.001 |
|  |  | b | -0.135 | -0.330 | 0.054 | 1.000 |
|  |  | c (Direct effect) | -0.928 | -1.122 | -0.738 | 1.000 |
|  |  | Indirect effect | 0.015 | -0.007 | 0.050 | 1.001 |
|  |  | Total effect | -0.914 | -1.105 | -0.726 | 1.000 |
|  |  | Indirect effect (%) | -1.634 | -5.697 | 0.744 | 1.001 |
| 41 | Saga | a | 0.185 | -0.003 | 0.376 | 1.027 |
|  |  | b | 0.307 | 0.171 | 0.446 | 1.007 |
|  |  | c (Direct effect) | -0.085 | -0.328 | 0.158 | 1.010 |
|  |  | Indirect effect | 0.057 | -0.001 | 0.127 | 1.024 |
|  |  | Total effect | -0.028 | -0.277 | 0.220 | 1.004 |
|  |  | Indirect effect (%) | 7790.793 | -722.391 | 754.441 | 1.000 |
| 42 | Nagasaki | a | 0.163 | -0.004 | 0.333 | 1.002 |
|  |  | b | 0.143 | 0.033 | 0.256 | 1.001 |
|  |  | c (Direct effect) | -0.341 | -0.565 | -0.121 | 1.001 |
|  |  | Indirect effect | 0.023 | -0.001 | 0.060 | 1.001 |
|  |  | Total effect | -0.318 | -0.541 | -0.098 | 1.001 |
|  |  | Indirect effect (%) | -9.109 | -34.061 | 0.500 | 1.000 |
| 43 | Kumamoto | a | 0.173 | -0.001 | 0.348 | 1.001 |
|  |  | b | 0.298 | 0.152 | 0.445 | 1.003 |
|  |  | c (Direct effect) | -0.518 | -0.765 | -0.266 | 1.001 |
|  |  | Indirect effect | 0.051 | 0.000 | 0.117 | 1.000 |
|  |  | Total effect | -0.466 | -0.716 | -0.211 | 1.001 |
|  |  | Indirect effect (%) | -12.725 | -36.768 | 0.044 | 1.000 |
| 44 | Oita | a | 0.195 | 0.006 | 0.389 | 1.001 |
|  |  | b | 0.205 | 0.094 | 0.322 | 1.005 |
|  |  | c (Direct effect) | -0.454 | -0.666 | -0.241 | 1.000 |
|  |  | Indirect effect | 0.040 | 0.001 | 0.093 | 1.001 |
|  |  | Total effect | -0.414 | -0.628 | -0.197 | 1.000 |
|  |  | Indirect effect (%) | -10.587 | -32.556 | -0.178 | 1.000 |
| 45 | Miyazaki | a | 0.207 | 0.029 | 0.384 | 1.001 |
|  |  | b | 0.354 | 0.183 | 0.536 | 1.007 |
|  |  | c (Direct effect) | -0.314 | -0.629 | -0.005 | 1.003 |
|  |  | Indirect effect | 0.073 | 0.009 | 0.156 | 1.003 |
|  |  | Total effect | -0.241 | -0.560 | 0.069 | 1.001 |
|  |  | Indirect effect (%) | -41.070 | -284.729 | 204.155 | 1.000 |
| 46 | Kagoshima | a | 0.077 | -0.099 | 0.250 | 1.000 |
|  |  | b | 0.156 | -0.015 | 0.332 | 1.001 |
|  |  | c (Direct effect) | -0.272 | -0.600 | 0.054 | 1.000 |
|  |  | Indirect effect | 0.012 | -0.016 | 0.055 | 1.001 |
|  |  | Total effect | -0.260 | -0.589 | 0.063 | 1.000 |
|  |  | Indirect effect (%) | -25.531 | -58.218 | 31.522 | 1.000 |
| 47 | Okinawa | a | 0.161 | 0.000 | 0.318 | 1.002 |
|  |  | b | 0.373 | 0.258 | 0.488 | 1.000 |
|  |  | c (Direct effect) | -0.883 | -1.093 | -0.680 | 1.004 |
|  |  | Indirect effect | 0.060 | 0.000 | 0.126 | 1.002 |
|  |  | Total effect | -0.823 | -1.042 | -0.607 | 1.005 |
|  |  | Indirect effect (%) | -7.640 | -17.700 | -0.020 | 1.005 |

**Table S11. Effects of NPIs on mitigating the COVID-19 infection mediated by Tokyo residents’ mobility during Phase 2**

| **Prefecture Number** | **Prefecture Name** | **Parameters** | **Mean** | **95% Credible Interval** | | **Rhat** |
| --- | --- | --- | --- | --- | --- | --- |
|  |  |  |  | **Low** | **High** |  |
| 1 | Hokkaido | a | -0.081 | -0.174 | 0.010 | 1.000 |
|  |  | b | 0.538 | 0.344 | 0.734 | 1.003 |
|  |  | c (Direct effect) | -0.146 | -0.238 | -0.046 | 1.001 |
|  |  | Indirect effect | -0.043 | -0.096 | 0.005 | 1.000 |
|  |  | Total effect | -0.189 | -0.289 | -0.084 | 1.001 |
|  |  | Indirect effect (%) | 23.221 | -3.586 | 56.623 | 1.000 |
| 2 | Aomori | a | - | - | - | - |
|  |  | b | - | - | - | - |
|  |  | c (Direct effect) | - | - | - | - |
|  |  | Indirect effect | - | - | - | - |
|  |  | Total effect | - | - | - | - |
|  |  | Indirect effect (%) | - | - | - | - |
| 3 | Iwate | a | - | - | - | - |
|  |  | b | - | - | - | - |
|  |  | c (Direct effect) | - | - | - | - |
|  |  | Indirect effect | - | - | - | - |
|  |  | Total effect | - | - | - | - |
|  |  | Indirect effect (%) | - | - | - | - |
| 4 | Miyagi | a | 0.083 | 0.008 | 0.158 | 1.000 |
|  |  | b | 0.516 | 0.171 | 0.911 | 1.001 |
|  |  | c (Direct effect) | -0.253 | -0.397 | -0.107 | 1.001 |
|  |  | Indirect effect | 0.044 | 0.002 | 0.115 | 1.000 |
|  |  | Total effect | -0.208 | -0.342 | -0.069 | 1.001 |
|  |  | Indirect effect (%) | -26.702 | -89.267 | -0.752 | 1.000 |
| 5 | Akita | a | - | - | - | - |
|  |  | b | - | - | - | - |
|  |  | c (Direct effect) | - | - | - | - |
|  |  | Indirect effect | - | - | - | - |
|  |  | Total effect | - | - | - | - |
|  |  | Indirect effect (%) | - | - | - | - |
| 6 | Yamagata | a | - | - | - | - |
|  |  | b | - | - | - | - |
|  |  | c (Direct effect) | - | - | - | - |
|  |  | Indirect effect | - | - | - | - |
|  |  | Total effect | - | - | - | - |
|  |  | Indirect effect (%) | - | - | - | - |
| 7 | Fukushima | a | 0.514 | 0.333 | 0.696 | 1.000 |
|  |  | b | 0.486 | 0.324 | 0.672 | 1.005 |
|  |  | c (Direct effect) | -0.525 | -0.656 | -0.395 | 1.002 |
|  |  | Indirect effect | 0.248 | 0.145 | 0.371 | 1.002 |
|  |  | Total effect | -0.277 | -0.416 | -0.138 | 1.000 |
|  |  | Indirect effect (%) | -99.520 | -236.788 | -39.895 | 1.000 |
| 8 | Ibaraki | a | 0.147 | 0.037 | 0.263 | 1.005 |
|  |  | b | 0.698 | 0.090 | 1.943 | 1.004 |
|  |  | c (Direct effect) | -0.234 | -0.378 | -0.100 | 1.002 |
|  |  | Indirect effect | 0.090 | 0.008 | 0.213 | 1.001 |
|  |  | Total effect | -0.143 | -0.288 | 0.002 | 1.002 |
|  |  | Indirect effect (%) | -125.748 | -534.843 | 156.287 | 1.000 |
| 9 | Tochigi | a | 0.351 | 0.188 | 0.521 | 1.000 |
|  |  | b | 0.582 | 0.281 | 1.059 | 1.002 |
|  |  | c (Direct effect) | -0.310 | -0.492 | -0.145 | 1.001 |
|  |  | Indirect effect | 0.201 | 0.081 | 0.380 | 1.001 |
|  |  | Total effect | -0.109 | -0.254 | 0.049 | 1.001 |
|  |  | Indirect effect (%) | -55.161 | -2008.963 | 1485.004 | 1.000 |
| 10 | Gunma | a | 0.104 | -0.015 | 0.223 | 1.000 |
|  |  | b | 0.685 | 0.400 | 1.009 | 1.002 |
|  |  | c (Direct effect) | -0.396 | -0.506 | -0.277 | 1.001 |
|  |  | Indirect effect | 0.072 | -0.010 | 0.171 | 1.000 |
|  |  | Total effect | -0.324 | -0.439 | -0.204 | 1.000 |
|  |  | Indirect effect (%) | -24.321 | -70.020 | 2.518 | 1.000 |
| 11 | Saitama | a | -0.014 | -0.026 | -0.001 | 1.000 |
|  |  | b | 4.189 | 2.043 | 6.888 | 1.002 |
|  |  | c (Direct effect) | 0.049 | -0.040 | 0.155 | 1.000 |
|  |  | Indirect effect | -0.061 | -0.151 | -0.004 | 1.001 |
|  |  | Total effect | -0.012 | -0.085 | 0.061 | 1.000 |
|  |  | Indirect effect (%) | -83.086 | -2282.215 | 2568.412 | 1.000 |
| 12 | Chiba | a | -0.032 | -0.063 | -0.001 | 1.002 |
|  |  | b | 5.101 | 1.992 | 15.038 | 1.004 |
|  |  | c (Direct effect) | 0.161 | -0.047 | 0.808 | 1.003 |
|  |  | Indirect effect | -0.198 | -0.851 | -0.004 | 1.004 |
|  |  | Total effect | -0.037 | -0.114 | 0.039 | 1.000 |
|  |  | Indirect effect (%) | -61.416 | -4052.572 | 5108.681 | 1.000 |
| 13 | Tokyo | a | 0.002 | -0.002 | 0.006 | 1.001 |
|  |  | b | -7.542 | -11.913 | -4.116 | 1.006 |
|  |  | c (Direct effect) | 0.018 | -0.036 | 0.071 | 1.009 |
|  |  | Indirect effect | -0.013 | -0.047 | 0.016 | 1.001 |
|  |  | Total effect | 0.005 | -0.047 | 0.056 | 1.007 |
|  |  | Indirect effect (%) | -20.929 | -888.933 | 985.416 | 1.000 |
| 14 | Kanagawa | a | -0.033 | -0.045 | -0.020 | 1.004 |
|  |  | b | 8.432 | 3.142 | 17.026 | 1.006 |
|  |  | c (Direct effect) | 0.189 | -0.045 | 0.614 | 1.007 |
|  |  | Indirect effect | -0.291 | -0.715 | -0.071 | 1.007 |
|  |  | Total effect | -0.101 | -0.174 | -0.027 | 1.000 |
|  |  | Indirect effect (%) | 374.793 | 59.421 | 1170.458 | 1.000 |
| 15 | Niigata | a | - | - | - | - |
|  |  | b | - | - | - | - |
|  |  | c (Direct effect) | - | - | - | - |
|  |  | Indirect effect | - | - | - | - |
|  |  | Total effect | - | - | - | - |
|  |  | Indirect effect (%) | - | - | - | - |
| 16 | Toyama | a | -0.339 | -0.529 | -0.149 | 1.000 |
|  |  | b | 0.307 | 0.160 | 0.462 | 1.002 |
|  |  | c (Direct effect) | -0.524 | -0.733 | -0.315 | 1.000 |
|  |  | Indirect effect | -0.104 | -0.190 | -0.037 | 1.001 |
|  |  | Total effect | -0.627 | -0.852 | -0.404 | 1.000 |
|  |  | Indirect effect (%) | 16.694 | 6.380 | 30.218 | 1.001 |
| 17 | Ishikawa | a | -0.138 | -0.264 | -0.012 | 1.000 |
|  |  | b | 0.329 | 0.157 | 0.510 | 1.002 |
|  |  | c (Direct effect) | -0.262 | -0.397 | -0.129 | 1.000 |
|  |  | Indirect effect | -0.045 | -0.101 | -0.003 | 1.000 |
|  |  | Total effect | -0.308 | -0.445 | -0.173 | 1.000 |
|  |  | Indirect effect (%) | 15.183 | 1.159 | 35.330 | 1.000 |
| 18 | Fukui | a | - | - | - | - |
|  |  | b | - | - | - | - |
|  |  | c (Direct effect) | - | - | - | - |
|  |  | Indirect effect | - | - | - | - |
|  |  | Total effect | - | - | - | - |
|  |  | Indirect effect (%) | - | - | - | - |
| 19 | Yamanashi | a | -0.294 | -0.458 | -0.132 | 1.000 |
|  |  | b | 0.395 | 0.105 | 0.706 | 1.000 |
|  |  | c (Direct effect) | -0.220 | -0.446 | 0.006 | 1.000 |
|  |  | Indirect effect | -0.115 | -0.233 | -0.025 | 1.000 |
|  |  | Total effect | -0.335 | -0.571 | -0.102 | 1.000 |
|  |  | Indirect effect (%) | 35.494 | 7.940 | 100.658 | 1.000 |
| 20 | Nagano | a | - | - | - | - |
|  |  | b | - | - | - | - |
|  |  | c (Direct effect) | - | - | - | - |
|  |  | Indirect effect | - | - | - | - |
|  |  | Total effect | - | - | - | - |
|  |  | Indirect effect (%) | - | - | - | - |
| 21 | Gifu | a | -0.113 | -0.253 | 0.026 | 1.000 |
|  |  | b | 0.558 | 0.428 | 0.702 | 1.001 |
|  |  | c (Direct effect) | -0.212 | -0.299 | -0.121 | 1.000 |
|  |  | Indirect effect | -0.063 | -0.142 | 0.015 | 1.000 |
|  |  | Total effect | -0.274 | -0.376 | -0.169 | 1.000 |
|  |  | Indirect effect (%) | 21.953 | -6.805 | 47.433 | 1.000 |
| 22 | Shizuoka | a | 0.435 | 0.287 | 0.582 | 1.000 |
|  |  | b | 1.013 | 0.693 | 1.384 | 1.003 |
|  |  | c (Direct effect) | -0.478 | -0.687 | -0.274 | 1.002 |
|  |  | Indirect effect | 0.441 | 0.244 | 0.686 | 1.002 |
|  |  | Total effect | -0.036 | -0.196 | 0.122 | 1.000 |
|  |  | Indirect effect (%) | -569.622 | -8383.756 | 8095.394 | 1.000 |
| 23 | Aichi | a | 0.097 | 0.041 | 0.152 | 1.009 |
|  |  | b | 1.443 | 1.216 | 1.673 | 1.003 |
|  |  | c (Direct effect) | -0.151 | -0.211 | -0.089 | 1.011 |
|  |  | Indirect effect | 0.139 | 0.060 | 0.214 | 1.013 |
|  |  | Total effect | -0.013 | -0.074 | 0.048 | 1.004 |
|  |  | Indirect effect (%) | -49.340 | -7368.615 | 6649.312 | 1.000 |
| 24 | Mie | a | -0.236 | -0.381 | -0.090 | 1.000 |
|  |  | b | 0.346 | 0.228 | 0.479 | 1.000 |
|  |  | c (Direct effect) | -0.279 | -0.370 | -0.184 | 1.000 |
|  |  | Indirect effect | -0.082 | -0.145 | -0.029 | 1.000 |
|  |  | Total effect | -0.360 | -0.453 | -0.267 | 1.000 |
|  |  | Indirect effect (%) | 22.716 | 8.559 | 40.029 | 1.000 |
| 25 | Shiga | a | 0.518 | 0.306 | 0.735 | 1.000 |
|  |  | b | 0.490 | 0.342 | 0.657 | 1.003 |
|  |  | c (Direct effect) | -0.267 | -0.402 | -0.137 | 1.000 |
|  |  | Indirect effect | 0.252 | 0.141 | 0.384 | 1.000 |
|  |  | Total effect | -0.015 | -0.171 | 0.146 | 1.000 |
|  |  | Indirect effect (%) | -159.563 | -5203.762 | 5179.942 | 1.000 |
| 26 | Kyoto | a | -0.088 | -0.186 | 0.011 | 1.000 |
|  |  | b | 0.370 | 0.166 | 0.575 | 1.000 |
|  |  | c (Direct effect) | -0.231 | -0.328 | -0.131 | 1.000 |
|  |  | Indirect effect | -0.034 | -0.086 | 0.003 | 1.000 |
|  |  | Total effect | -0.264 | -0.356 | -0.172 | 1.000 |
|  |  | Indirect effect (%) | 12.989 | -1.307 | 34.691 | 1.000 |
| 27 | Osaka | a | 0.020 | -0.059 | 0.101 | 1.001 |
|  |  | b | 0.753 | 0.561 | 0.956 | 1.003 |
|  |  | c (Direct effect) | -0.123 | -0.188 | -0.058 | 1.000 |
|  |  | Indirect effect | 0.015 | -0.046 | 0.075 | 1.001 |
|  |  | Total effect | -0.108 | -0.176 | -0.041 | 1.001 |
|  |  | Indirect effect (%) | -22.818 | -133.543 | 35.560 | 1.000 |
| 28 | Hyogo | a | 0.077 | -0.025 | 0.179 | 1.000 |
|  |  | b | 0.446 | 0.253 | 0.692 | 1.001 |
|  |  | c (Direct effect) | -0.181 | -0.251 | -0.114 | 1.000 |
|  |  | Indirect effect | 0.035 | -0.010 | 0.094 | 1.000 |
|  |  | Total effect | -0.146 | -0.209 | -0.082 | 1.000 |
|  |  | Indirect effect (%) | -27.324 | -86.287 | 5.939 | 1.000 |
| 29 | Nara | a | - | - | - | - |
|  |  | b | - | - | - | - |
|  |  | c (Direct effect) | - | - | - | - |
|  |  | Indirect effect | - | - | - | - |
|  |  | Total effect | - | - | - | - |
|  |  | Indirect effect (%) | - | - | - | - |
| 30 | Wakayama | a | - | - | - | - |
|  |  | b | - | - | - | - |
|  |  | c (Direct effect) | - | - | - | - |
|  |  | Indirect effect | - | - | - | - |
|  |  | Total effect | - | - | - | - |
|  |  | Indirect effect (%) | - | - | - | - |
| 31 | Tottori | a | - | - | - | - |
|  |  | b | - | - | - | - |
|  |  | c (Direct effect) | - | - | - | - |
|  |  | Indirect effect | - | - | - | - |
|  |  | Total effect | - | - | - | - |
|  |  | Indirect effect (%) | - | - | - | - |
| 32 | Shimane | a | - | - | - | - |
|  |  | b | - | - | - | - |
|  |  | c (Direct effect) | - | - | - | - |
|  |  | Indirect effect | - | - | - | - |
|  |  | Total effect | - | - | - | - |
|  |  | Indirect effect (%) | - | - | - | - |
| 33 | Okayama | a | -0.254 | -0.377 | -0.127 | 1.000 |
|  |  | b | 0.253 | 0.071 | 0.463 | 1.001 |
|  |  | c (Direct effect) | -0.307 | -0.482 | -0.147 | 1.001 |
|  |  | Indirect effect | -0.064 | -0.136 | -0.015 | 1.002 |
|  |  | Total effect | -0.372 | -0.544 | -0.219 | 1.001 |
|  |  | Indirect effect (%) | 18.078 | 4.006 | 41.464 | 1.002 |
| 34 | Hiroshima | a | -0.158 | -0.260 | -0.055 | 1.000 |
|  |  | b | 0.718 | 0.563 | 0.886 | 1.000 |
|  |  | c (Direct effect) | -0.181 | -0.284 | -0.077 | 1.000 |
|  |  | Indirect effect | -0.114 | -0.196 | -0.038 | 1.000 |
|  |  | Total effect | -0.295 | -0.410 | -0.178 | 1.000 |
|  |  | Indirect effect (%) | 38.750 | 15.444 | 65.967 | 1.000 |
| 35 | Yamaguchi | a | - | - | - | - |
|  |  | b | - | - | - | - |
|  |  | c (Direct effect) | - | - | - | - |
|  |  | Indirect effect | - | - | - | - |
|  |  | Total effect | - | - | - | - |
|  |  | Indirect effect (%) | - | - | - | - |
| 36 | Tokushima | a | - | - | - | - |
|  |  | b | - | - | - | - |
|  |  | c (Direct effect) | - | - | - | - |
|  |  | Indirect effect | - | - | - | - |
|  |  | Total effect | - | - | - | - |
|  |  | Indirect effect (%) | - | - | - | - |
| 37 | Kagawa | a | 0.045 | -0.151 | 0.241 | 1.000 |
|  |  | b | 0.523 | 0.381 | 0.671 | 1.001 |
|  |  | c (Direct effect) | -0.541 | -0.699 | -0.379 | 1.001 |
|  |  | Indirect effect | 0.024 | -0.078 | 0.130 | 1.000 |
|  |  | Total effect | -0.518 | -0.702 | -0.331 | 1.000 |
|  |  | Indirect effect (%) | -5.825 | -32.982 | 13.169 | 1.000 |
| 38 | Ehime | a | -0.124 | -0.242 | -0.005 | 1.000 |
|  |  | b | 0.285 | 0.106 | 0.467 | 1.000 |
|  |  | c (Direct effect) | -0.426 | -0.609 | -0.243 | 1.000 |
|  |  | Indirect effect | -0.035 | -0.084 | -0.001 | 1.000 |
|  |  | Total effect | -0.461 | -0.649 | -0.273 | 1.000 |
|  |  | Indirect effect (%) | 7.766 | 0.256 | 18.451 | 1.000 |
| 39 | Kochi | a | -0.132 | -0.347 | 0.081 | 1.000 |
|  |  | b | 0.340 | 0.198 | 0.486 | 1.000 |
|  |  | c (Direct effect) | -0.403 | -0.674 | -0.128 | 1.000 |
|  |  | Indirect effect | -0.045 | -0.128 | 0.027 | 1.000 |
|  |  | Total effect | -0.448 | -0.729 | -0.167 | 1.001 |
|  |  | Indirect effect (%) | 10.710 | -7.877 | 34.211 | 1.000 |
| 40 | Fukuoka | a | -0.106 | -0.183 | -0.028 | 1.001 |
|  |  | b | 0.226 | 0.051 | 0.448 | 1.003 |
|  |  | c (Direct effect) | -0.279 | -0.365 | -0.193 | 1.002 |
|  |  | Indirect effect | -0.023 | -0.053 | -0.003 | 1.001 |
|  |  | Total effect | -0.303 | -0.391 | -0.215 | 1.002 |
|  |  | Indirect effect (%) | 7.731 | 1.005 | 17.539 | 1.001 |
| 41 | Saga | a | -0.072 | -0.285 | 0.143 | 1.001 |
|  |  | b | 0.249 | 0.140 | 0.359 | 1.001 |
|  |  | c (Direct effect) | -0.503 | -0.748 | -0.259 | 1.000 |
|  |  | Indirect effect | -0.018 | -0.076 | 0.037 | 1.001 |
|  |  | Total effect | -0.521 | -0.774 | -0.273 | 1.000 |
|  |  | Indirect effect (%) | 3.326 | -8.580 | 15.268 | 1.001 |
| 42 | Nagasaki | a | -0.359 | -0.584 | -0.133 | 1.001 |
|  |  | b | 0.203 | 0.083 | 0.327 | 1.002 |
|  |  | c (Direct effect) | -0.162 | -0.417 | 0.095 | 1.000 |
|  |  | Indirect effect | -0.073 | -0.151 | -0.019 | 1.002 |
|  |  | Total effect | -0.235 | -0.490 | 0.021 | 1.000 |
|  |  | Indirect effect (%) | 41.686 | -82.526 | 202.913 | 1.000 |
| 43 | Kumamoto | a | 0.100 | -0.028 | 0.233 | 1.002 |
|  |  | b | 0.498 | 0.365 | 0.637 | 1.007 |
|  |  | c (Direct effect) | -0.376 | -0.495 | -0.255 | 1.000 |
|  |  | Indirect effect | 0.050 | -0.014 | 0.120 | 1.001 |
|  |  | Total effect | -0.326 | -0.461 | -0.189 | 1.000 |
|  |  | Indirect effect (%) | -17.342 | -52.180 | 3.785 | 1.000 |
| 44 | Oita | a | - | - | - | - |
|  |  | b | - | - | - | - |
|  |  | c (Direct effect) | - | - | - | - |
|  |  | Indirect effect | - | - | - | - |
|  |  | Total effect | - | - | - | - |
|  |  | Indirect effect (%) | - | - | - | - |
| 45 | Miyazaki | a | 0.063 | -0.128 | 0.247 | 1.065 |
|  |  | b | 0.538 | 0.381 | 0.703 | 1.030 |
|  |  | c (Direct effect) | -0.380 | -0.633 | -0.131 | 1.021 |
|  |  | Indirect effect | 0.034 | -0.071 | 0.134 | 1.073 |
|  |  | Total effect | -0.346 | -0.616 | -0.078 | 1.008 |
|  |  | Indirect effect (%) | -14.616 | -91.835 | 21.638 | 1.000 |
| 46 | Kagoshima | a | -0.015 | -0.191 | 0.163 | 1.000 |
|  |  | b | 0.436 | 0.312 | 0.560 | 1.001 |
|  |  | c (Direct effect) | -0.482 | -0.648 | -0.313 | 1.000 |
|  |  | Indirect effect | -0.006 | -0.085 | 0.072 | 1.000 |
|  |  | Total effect | -0.488 | -0.671 | -0.306 | 1.000 |
|  |  | Indirect effect (%) | 0.638 | -18.674 | 15.783 | 1.000 |
| 47 | Okinawa | a | 0.180 | 0.010 | 0.352 | 1.003 |
|  |  | b | 0.222 | 0.126 | 0.326 | 1.002 |
|  |  | c (Direct effect) | -0.075 | -0.205 | 0.048 | 1.005 |
|  |  | Indirect effect | 0.040 | 0.002 | 0.087 | 1.004 |
|  |  | Total effect | -0.036 | -0.168 | 0.095 | 1.004 |
|  |  | Indirect effect (%) | -669.857 | -823.371 | 820.346 | 1.000 |

Prefectures where NPIs were not implemented are represented by "-".

**Table S12. Effects of NPIs on mitigating the COVID-19 infection mediated by Tokyo residents’ mobility during Phase 3**

| **Prefecture Number** | **Prefecture Name** | **Parameters** | **Mean** | **95% Credible Interval** | | **Rhat** |
| --- | --- | --- | --- | --- | --- | --- |
|  |  |  |  | **Low** | **High** |  |
| 1 | Hokkaido | a | -0.138 | -0.304 | 0.036 | 1.007 |
|  |  | b | 0.092 | 0.025 | 0.158 | 1.003 |
|  |  | c (Direct effect) | -0.178 | -0.309 | -0.065 | 1.055 |
|  |  | Indirect effect | -0.013 | -0.034 | 0.003 | 1.010 |
|  |  | Total effect | -0.190 | -0.321 | -0.077 | 1.049 |
|  |  | Indirect effect (%) | 7.830 | -1.839 | 23.547 | 1.008 |
| 2 | Aomori | a | -0.134 | -0.483 | 0.228 | 1.006 |
|  |  | b | 0.393 | 0.344 | 0.442 | 1.005 |
|  |  | c (Direct effect) | -0.019 | -0.167 | 0.132 | 1.004 |
|  |  | Indirect effect | -0.053 | -0.191 | 0.091 | 1.006 |
|  |  | Total effect | -0.071 | -0.267 | 0.131 | 1.003 |
|  |  | Indirect effect (%) | 176.875 | -477.045 | 613.795 | 1.000 |
| 3 | Iwate | a | - | - | - | - |
|  |  | b | - | - | - | - |
|  |  | c (Direct effect) | - | - | - | - |
|  |  | Indirect effect | - | - | - | - |
|  |  | Total effect | - | - | - | - |
|  |  | Indirect effect (%) | - | - | - | - |
| 4 | Miyagi | a | - | - | - | - |
|  |  | b | - | - | - | - |
|  |  | c (Direct effect) | - | - | - | - |
|  |  | Indirect effect | - | - | - | - |
|  |  | Total effect | - | - | - | - |
|  |  | Indirect effect (%) | - | - | - | - |
| 5 | Akita | a | - | - | - | - |
|  |  | b | - | - | - | - |
|  |  | c (Direct effect) | - | - | - | - |
|  |  | Indirect effect | - | - | - | - |
|  |  | Total effect | - | - | - | - |
|  |  | Indirect effect (%) | - | - | - | - |
| 6 | Yamagata | a | 0.024 | -0.316 | 0.357 | 1.004 |
|  |  | b | 0.363 | 0.315 | 0.416 | 1.004 |
|  |  | c (Direct effect) | -0.206 | -0.364 | -0.049 | 1.002 |
|  |  | Indirect effect | 0.009 | -0.115 | 0.130 | 1.004 |
|  |  | Total effect | -0.197 | -0.397 | 0.002 | 1.001 |
|  |  | Indirect effect (%) | -14.693 | -248.149 | 89.517 | 1.000 |
| 7 | Fukushima | a | -0.213 | -0.533 | 0.097 | 1.003 |
|  |  | b | 0.154 | 0.047 | 0.301 | 1.003 |
|  |  | c (Direct effect) | -0.248 | -0.428 | -0.063 | 1.000 |
|  |  | Indirect effect | -0.036 | -0.124 | 0.012 | 1.003 |
|  |  | Total effect | -0.284 | -0.456 | -0.111 | 1.001 |
|  |  | Indirect effect (%) | 13.619 | -5.420 | 54.672 | 1.000 |
| 8 | Ibaraki | a | -0.087 | -0.270 | 0.108 | 1.047 |
|  |  | b | 0.789 | 0.606 | 0.971 | 1.082 |
|  |  | c (Direct effect) | -0.082 | -0.228 | 0.072 | 1.082 |
|  |  | Indirect effect | -0.069 | -0.223 | 0.082 | 1.045 |
|  |  | Total effect | -0.151 | -0.331 | 0.024 | 1.042 |
|  |  | Indirect effect (%) | 37.346 | -175.191 | 271.321 | 1.000 |
| 9 | Tochigi | a | -0.364 | -0.491 | -0.237 | 1.005 |
|  |  | b | 10.258 | -8.202 | 29.846 | 1.096 |
|  |  | c (Direct effect) | 3.436 | -3.104 | 10.001 | 1.084 |
|  |  | Indirect effect | -3.684 | -10.254 | 2.843 | 1.084 |
|  |  | Total effect | -0.248 | -0.366 | -0.129 | 1.000 |
|  |  | Indirect effect (%) | 1597.368 | -1127.527 | 4905.681 | 1.065 |
| 10 | Gunma | a | -0.267 | -0.514 | -0.018 | 1.007 |
|  |  | b | 0.208 | 0.128 | 0.346 | 1.007 |
|  |  | c (Direct effect) | -0.106 | -0.223 | 0.015 | 1.005 |
|  |  | Indirect effect | -0.057 | -0.141 | -0.003 | 1.003 |
|  |  | Total effect | -0.163 | -0.278 | -0.048 | 1.004 |
|  |  | Indirect effect (%) | 39.714 | 1.592 | 117.290 | 1.000 |
| 11 | Saitama | a | -0.047 | -0.075 | -0.019 | 1.008 |
|  |  | b | 4.674 | 3.610 | 5.990 | 1.004 |
|  |  | c (Direct effect) | -0.058 | -0.205 | 0.132 | 1.007 |
|  |  | Indirect effect | -0.222 | -0.410 | -0.077 | 1.008 |
|  |  | Total effect | -0.279 | -0.414 | -0.141 | 1.001 |
|  |  | Indirect effect (%) | 81.613 | 33.079 | 162.405 | 1.001 |
| 12 | Chiba | a | -0.151 | -0.175 | -0.127 | 1.024 |
|  |  | b | 19.857 | 12.825 | 33.615 | 1.086 |
|  |  | c (Direct effect) | 2.654 | 1.628 | 4.705 | 1.074 |
|  |  | Indirect effect | -2.981 | -5.015 | -1.956 | 1.072 |
|  |  | Total effect | -0.327 | -0.459 | -0.199 | 1.004 |
|  |  | Indirect effect (%) | 950.472 | 533.995 | 1769.801 | 1.045 |
| 13 | Tokyo | a | 0.007 | -0.007 | 0.018 | 1.075 |
|  |  | b | -6.472 | -7.653 | -5.588 | 1.028 |
|  |  | c (Direct effect) | -0.260 | -0.344 | -0.177 | 1.003 |
|  |  | Indirect effect | -0.043 | -0.123 | 0.042 | 1.081 |
|  |  | Total effect | -0.303 | -0.416 | -0.195 | 1.048 |
|  |  | Indirect effect (%) | 12.547 | -21.164 | 33.725 | 1.077 |
| 14 | Kanagawa | a | -0.078 | -0.093 | -0.064 | 1.235 |
|  |  | b | 17.143 | 8.012 | 33.953 | 1.063 |
|  |  | c (Direct effect) | 1.053 | 0.209 | 2.618 | 1.101 |
|  |  | Indirect effect | -1.358 | -2.950 | -0.543 | 1.101 |
|  |  | Total effect | -0.305 | -0.449 | -0.208 | 1.349 |
|  |  | Indirect effect (%) | 467.672 | 159.909 | 977.781 | 1.142 |
| 15 | Niigata | a | -0.097 | -0.392 | 0.205 | 1.003 |
|  |  | b | 0.230 | 0.181 | 0.284 | 1.006 |
|  |  | c (Direct effect) | -0.329 | -0.461 | -0.194 | 1.003 |
|  |  | Indirect effect | -0.022 | -0.093 | 0.047 | 1.003 |
|  |  | Total effect | -0.351 | -0.505 | -0.202 | 1.003 |
|  |  | Indirect effect (%) | 5.543 | -17.971 | 24.991 | 1.002 |
| 16 | Toyama | a | - | - | - | - |
|  |  | b | - | - | - | - |
|  |  | c (Direct effect) | - | - | - | - |
|  |  | Indirect effect | - | - | - | - |
|  |  | Total effect | - | - | - | - |
|  |  | Indirect effect (%) | - | - | - | - |
| 17 | Ishikawa | a | -0.443 | -0.609 | -0.152 | 1.151 |
|  |  | b | -22.687 | -174.073 | 34.580 | 2.509 |
|  |  | c (Direct effect) | -11.061 | -81.265 | 16.625 | 2.547 |
|  |  | Indirect effect | 10.579 | -17.107 | 80.771 | 2.547 |
|  |  | Total effect | -0.482 | -0.638 | -0.328 | 1.003 |
|  |  | Indirect effect (%) | -2293.197 | -18372.594 | 3706.028 | 2.332 |
| 18 | Fukui | a | - | - | - | - |
|  |  | b | - | - | - | - |
|  |  | c (Direct effect) | - | - | - | - |
|  |  | Indirect effect | - | - | - | - |
|  |  | Total effect | - | - | - | - |
|  |  | Indirect effect (%) | - | - | - | - |
| 19 | Yamanashi | a | - | - | - | - |
|  |  | b | - | - | - | - |
|  |  | c (Direct effect) | - | - | - | - |
|  |  | Indirect effect | - | - | - | - |
|  |  | Total effect | - | - | - | - |
|  |  | Indirect effect (%) | - | - | - | - |
| 20 | Nagano | a | 0.022 | -0.255 | 0.301 | 1.001 |
|  |  | b | 0.220 | 0.177 | 0.266 | 1.001 |
|  |  | c (Direct effect) | -0.232 | -0.346 | -0.114 | 1.000 |
|  |  | Indirect effect | 0.005 | -0.056 | 0.067 | 1.000 |
|  |  | Total effect | -0.227 | -0.357 | -0.095 | 1.000 |
|  |  | Indirect effect (%) | -6.849 | -50.952 | 21.816 | 1.000 |
| 21 | Gifu | a | -0.445 | -0.792 | -0.091 | 1.001 |
|  |  | b | 0.495 | 0.380 | 0.635 | 1.002 |
|  |  | c (Direct effect) | -0.108 | -0.318 | 0.115 | 1.000 |
|  |  | Indirect effect | -0.223 | -0.434 | -0.043 | 1.001 |
|  |  | Total effect | -0.331 | -0.560 | -0.098 | 1.000 |
|  |  | Indirect effect (%) | 38.775 | 17.721 | 166.698 | 1.000 |
| 22 | Shizuoka | a | -0.185 | -0.408 | 0.049 | 1.075 |
|  |  | b | 0.558 | 0.480 | 0.651 | 1.048 |
|  |  | c (Direct effect) | -0.165 | -0.304 | -0.030 | 1.076 |
|  |  | Indirect effect | -0.103 | -0.223 | 0.027 | 1.065 |
|  |  | Total effect | -0.268 | -0.453 | -0.099 | 1.017 |
|  |  | Indirect effect (%) | 36.855 | -18.069 | 81.586 | 1.001 |
| 23 | Aichi | a | -0.214 | -0.378 | -0.047 | 1.001 |
|  |  | b | 0.730 | 0.559 | 0.961 | 1.017 |
|  |  | c (Direct effect) | -0.170 | -0.304 | -0.013 | 1.003 |
|  |  | Indirect effect | -0.159 | -0.319 | -0.031 | 1.004 |
|  |  | Total effect | -0.329 | -0.459 | -0.199 | 1.002 |
|  |  | Indirect effect (%) | 48.207 | 11.654 | 95.627 | 1.003 |
| 24 | Mie | a | -0.431 | -0.728 | -0.131 | 1.004 |
|  |  | b | 0.530 | 0.403 | 0.676 | 1.004 |
|  |  | c (Direct effect) | 0.029 | -0.144 | 0.220 | 1.003 |
|  |  | Indirect effect | -0.230 | -0.421 | -0.064 | 1.003 |
|  |  | Total effect | -0.201 | -0.360 | -0.040 | 1.000 |
|  |  | Indirect effect (%) | 120.033 | 36.224 | 378.244 | 1.000 |
| 25 | Shiga | a | - | - | - | - |
|  |  | b | - | - | - | - |
|  |  | c (Direct effect) | - | - | - | - |
|  |  | Indirect effect | - | - | - | - |
|  |  | Total effect | - | - | - | - |
|  |  | Indirect effect (%) | - | - | - | - |
| 26 | Kyoto | a | -0.257 | -0.444 | -0.060 | 1.000 |
|  |  | b | 0.374 | 0.242 | 0.544 | 1.002 |
|  |  | c (Direct effect) | -0.184 | -0.295 | -0.061 | 1.001 |
|  |  | Indirect effect | -0.098 | -0.203 | -0.019 | 1.001 |
|  |  | Total effect | -0.282 | -0.381 | -0.184 | 1.000 |
|  |  | Indirect effect (%) | 35.159 | 7.409 | 74.411 | 1.001 |
| 27 | Osaka | a | -0.130 | -0.281 | 0.026 | 1.002 |
|  |  | b | 0.525 | 0.402 | 0.671 | 1.019 |
|  |  | c (Direct effect) | -0.186 | -0.278 | -0.089 | 1.009 |
|  |  | Indirect effect | -0.068 | -0.155 | 0.013 | 1.001 |
|  |  | Total effect | -0.254 | -0.362 | -0.141 | 1.008 |
|  |  | Indirect effect (%) | 25.944 | -7.337 | 56.167 | 1.001 |
| 28 | Hyogo | a | -0.235 | -0.480 | 0.012 | 1.000 |
|  |  | b | 0.374 | 0.248 | 0.524 | 1.005 |
|  |  | c (Direct effect) | -0.202 | -0.338 | -0.061 | 1.002 |
|  |  | Indirect effect | -0.089 | -0.203 | 0.004 | 1.001 |
|  |  | Total effect | -0.290 | -0.429 | -0.153 | 1.000 |
|  |  | Indirect effect (%) | 30.706 | -1.789 | 71.464 | 1.001 |
| 29 | Nara | a | - | - | - | - |
|  |  | b | - | - | - | - |
|  |  | c (Direct effect) | - | - | - | - |
|  |  | Indirect effect | - | - | - | - |
|  |  | Total effect | - | - | - | - |
|  |  | Indirect effect (%) | - | - | - | - |
| 30 | Wakayama | a | -0.055 | -0.359 | 0.302 | 1.029 |
|  |  | b | 0.313 | 0.246 | 0.379 | 1.064 |
|  |  | c (Direct effect) | -0.062 | -0.192 | 0.059 | 1.013 |
|  |  | Indirect effect | -0.017 | -0.113 | 0.092 | 1.031 |
|  |  | Total effect | -0.079 | -0.218 | 0.070 | 1.004 |
|  |  | Indirect effect (%) | 3793.565 | -386.144 | 420.949 | 1.000 |
| 31 | Tottori | a | - | - | - | - |
|  |  | b | - | - | - | - |
|  |  | c (Direct effect) | - | - | - | - |
|  |  | Indirect effect | - | - | - | - |
|  |  | Total effect | - | - | - | - |
|  |  | Indirect effect (%) | - | - | - | - |
| 32 | Shimane | a | -0.199 | -0.541 | 0.150 | 1.000 |
|  |  | b | 0.202 | 0.103 | 0.315 | 1.000 |
|  |  | c (Direct effect) | -0.310 | -0.526 | -0.090 | 1.000 |
|  |  | Indirect effect | -0.042 | -0.132 | 0.027 | 1.000 |
|  |  | Total effect | -0.351 | -0.565 | -0.136 | 1.000 |
|  |  | Indirect effect (%) | 12.910 | -9.993 | 47.552 | 1.000 |
| 33 | Okayama | a | -0.227 | -0.493 | 0.042 | 1.000 |
|  |  | b | 0.616 | 0.549 | 0.697 | 1.004 |
|  |  | c (Direct effect) | 0.034 | -0.117 | 0.187 | 1.001 |
|  |  | Indirect effect | -0.140 | -0.305 | 0.026 | 1.001 |
|  |  | Total effect | -0.106 | -0.321 | 0.116 | 1.000 |
|  |  | Indirect effect (%) | -3.907 | -656.425 | 845.677 | 1.000 |
| 34 | Hiroshima | a | -0.827 | -1.045 | -0.606 | 1.001 |
|  |  | b | 0.449 | 0.327 | 0.625 | 1.011 |
|  |  | c (Direct effect) | -0.401 | -0.584 | -0.203 | 1.004 |
|  |  | Indirect effect | -0.370 | -0.535 | -0.243 | 1.005 |
|  |  | Total effect | -0.771 | -0.946 | -0.596 | 1.000 |
|  |  | Indirect effect (%) | 48.325 | 32.070 | 70.254 | 1.006 |
| 35 | Yamaguchi | a | -0.828 | -1.148 | -0.501 | 1.000 |
|  |  | b | 0.176 | 0.123 | 0.263 | 1.014 |
|  |  | c (Direct effect) | -0.210 | -0.329 | -0.085 | 1.002 |
|  |  | Indirect effect | -0.145 | -0.235 | -0.080 | 1.008 |
|  |  | Total effect | -0.355 | -0.477 | -0.234 | 1.001 |
|  |  | Indirect effect (%) | 41.604 | 23.433 | 69.796 | 1.006 |
| 36 | Tokushima | a | - | - | - | - |
|  |  | b | - | - | - | - |
|  |  | c (Direct effect) | - | - | - | - |
|  |  | Indirect effect | - | - | - | - |
|  |  | Total effect | - | - | - | - |
|  |  | Indirect effect (%) | - | - | - | - |
| 37 | Kagawa | a | -0.247 | -0.466 | 0.021 | 1.063 |
|  |  | b | 0.333 | 0.291 | 0.386 | 1.025 |
|  |  | c (Direct effect) | -0.112 | -0.206 | -0.008 | 1.013 |
|  |  | Indirect effect | -0.082 | -0.158 | 0.007 | 1.053 |
|  |  | Total effect | -0.194 | -0.304 | -0.063 | 1.055 |
|  |  | Indirect effect (%) | 42.540 | -5.751 | 92.851 | 1.000 |
| 38 | Ehime | a | - | - | - | - |
|  |  | b | - | - | - | - |
|  |  | c (Direct effect) | - | - | - | - |
|  |  | Indirect effect | - | - | - | - |
|  |  | Total effect | - | - | - | - |
|  |  | Indirect effect (%) | - | - | - | - |
| 39 | Kochi | a | 0.034 | -0.229 | 0.295 | 1.000 |
|  |  | b | 0.357 | 0.293 | 0.436 | 1.009 |
|  |  | c (Direct effect) | -0.042 | -0.157 | 0.073 | 1.000 |
|  |  | Indirect effect | 0.013 | -0.082 | 0.108 | 1.000 |
|  |  | Total effect | -0.030 | -0.171 | 0.113 | 1.000 |
|  |  | Indirect effect (%) | 132.032 | -701.819 | 807.007 | 1.000 |
| 40 | Fukuoka | a | -0.121 | -0.288 | 0.042 | 1.002 |
|  |  | b | 0.455 | 0.365 | 0.556 | 1.009 |
|  |  | c (Direct effect) | -0.243 | -0.390 | -0.102 | 1.003 |
|  |  | Indirect effect | -0.055 | -0.135 | 0.019 | 1.003 |
|  |  | Total effect | -0.299 | -0.461 | -0.144 | 1.005 |
|  |  | Indirect effect (%) | 18.343 | -8.619 | 44.807 | 1.000 |
| 41 | Saga | a | -0.023 | -0.352 | 0.306 | 1.000 |
|  |  | b | 0.334 | 0.254 | 0.427 | 1.003 |
|  |  | c (Direct effect) | -0.080 | -0.230 | 0.068 | 1.000 |
|  |  | Indirect effect | -0.008 | -0.120 | 0.103 | 1.000 |
|  |  | Total effect | -0.088 | -0.257 | 0.080 | 1.000 |
|  |  | Indirect effect (%) | -7.068 | -440.310 | 438.256 | 1.000 |
| 42 | Nagasaki | a | -0.482 | -0.724 | -0.240 | 1.001 |
|  |  | b | 0.389 | 0.317 | 0.468 | 1.002 |
|  |  | c (Direct effect) | -0.135 | -0.297 | 0.026 | 1.001 |
|  |  | Indirect effect | -0.188 | -0.297 | -0.090 | 1.002 |
|  |  | Total effect | -0.323 | -0.496 | -0.149 | 1.000 |
|  |  | Indirect effect (%) | 62.150 | 30.396 | 114.394 | 1.000 |
| 43 | Kumamoto | a | -0.339 | -0.576 | -0.109 | 1.001 |
|  |  | b | 0.801 | 0.711 | 0.900 | 1.001 |
|  |  | c (Direct effect) | -0.233 | -0.427 | -0.036 | 1.000 |
|  |  | Indirect effect | -0.272 | -0.468 | -0.086 | 1.001 |
|  |  | Total effect | -0.504 | -0.767 | -0.242 | 1.000 |
|  |  | Indirect effect (%) | 54.361 | 23.505 | 89.593 | 1.001 |
| 44 | Oita | a | -0.048 | -0.311 | 0.213 | 1.000 |
|  |  | b | 0.599 | 0.524 | 0.689 | 1.002 |
|  |  | c (Direct effect) | -0.216 | -0.387 | -0.045 | 1.000 |
|  |  | Indirect effect | -0.029 | -0.187 | 0.127 | 1.000 |
|  |  | Total effect | -0.245 | -0.473 | -0.018 | 1.000 |
|  |  | Indirect effect (%) | -46.203 | -171.505 | 76.884 | 1.000 |
| 45 | Miyazaki | a | -0.224 | -0.452 | 0.004 | 1.001 |
|  |  | b | 0.610 | 0.536 | 0.691 | 1.003 |
|  |  | c (Direct effect) | -0.237 | -0.394 | -0.073 | 1.001 |
|  |  | Indirect effect | -0.137 | -0.281 | 0.003 | 1.001 |
|  |  | Total effect | -0.374 | -0.582 | -0.160 | 1.001 |
|  |  | Indirect effect (%) | 35.134 | -1.026 | 69.318 | 1.000 |
| 46 | Kagoshima | a | -0.215 | -0.435 | 0.007 | 1.001 |
|  |  | b | 0.474 | 0.380 | 0.569 | 1.000 |
|  |  | c (Direct effect) | -0.165 | -0.349 | 0.028 | 1.002 |
|  |  | Indirect effect | -0.102 | -0.212 | 0.004 | 1.001 |
|  |  | Total effect | -0.267 | -0.473 | -0.047 | 1.002 |
|  |  | Indirect effect (%) | 47.629 | -2.559 | 125.124 | 1.001 |
| 47 | Okinawa | a | -0.589 | -0.755 | -0.423 | 1.002 |
|  |  | b | 0.611 | 0.531 | 0.706 | 1.008 |
|  |  | c (Direct effect) | -0.412 | -0.541 | -0.279 | 1.008 |
|  |  | Indirect effect | -0.361 | -0.491 | -0.248 | 1.004 |
|  |  | Total effect | -0.772 | -0.922 | -0.619 | 1.005 |
|  |  | Indirect effect (%) | 46.722 | 34.559 | 60.717 | 1.006 |

Prefectures where NPIs were not implemented are represented by "-".

**Table S13. Effects of NPIs on mitigating the COVID-19 infection mediated by Aichi residents’ mobility during Phase 1**

| **Prefecture Number** | **Prefecture Name** | **Parameters** | **Mean** | **95% Credible Interval** | | **Rhat** |
| --- | --- | --- | --- | --- | --- | --- |
|  |  |  |  | **Low** | **High** |  |
| 1 | Hokkaido | a | 0.023 | -0.153 | 0.198 | 1.000 |
|  |  | b | 0.233 | 0.148 | 0.325 | 1.001 |
|  |  | c (Direct effect) | -0.319 | -0.444 | -0.195 | 1.000 |
|  |  | Indirect effect | 0.006 | -0.036 | 0.049 | 1.000 |
|  |  | Total effect | -0.314 | -0.444 | -0.185 | 1.000 |
|  |  | Indirect effect (%) | -2.382 | -19.846 | 10.926 | 1.000 |
| 2 | Aomori | a | 0.302 | 0.068 | 0.537 | 1.000 |
|  |  | b | 0.202 | 0.060 | 0.343 | 1.000 |
|  |  | c (Direct effect) | -0.046 | -0.306 | 0.214 | 1.000 |
|  |  | Indirect effect | 0.061 | 0.007 | 0.138 | 1.000 |
|  |  | Total effect | 0.015 | -0.253 | 0.281 | 1.000 |
|  |  | Indirect effect (%) | 153.611 | -682.801 | 715.162 | 1.000 |
| 3 | Iwate | a | 0.091 | -0.063 | 0.243 | 1.000 |
|  |  | b | 0.275 | 0.103 | 0.456 | 1.003 |
|  |  | c (Direct effect) | -0.087 | -0.264 | 0.091 | 1.001 |
|  |  | Indirect effect | 0.025 | -0.017 | 0.079 | 1.001 |
|  |  | Total effect | -0.062 | -0.244 | 0.121 | 1.000 |
|  |  | Indirect effect (%) | 26.153 | -427.155 | 392.262 | 1.000 |
| 4 | Miyagi | a | 0.190 | 0.064 | 0.317 | 1.000 |
|  |  | b | 0.195 | -0.050 | 0.452 | 1.000 |
|  |  | c (Direct effect) | -0.756 | -0.952 | -0.559 | 1.000 |
|  |  | Indirect effect | 0.037 | -0.009 | 0.102 | 1.000 |
|  |  | Total effect | -0.719 | -0.911 | -0.523 | 1.000 |
|  |  | Indirect effect (%) | -5.365 | -15.528 | 1.192 | 1.000 |
| 5 | Akita | a | 0.405 | 0.117 | 0.684 | 1.000 |
|  |  | b | 0.053 | -0.122 | 0.233 | 1.000 |
|  |  | c (Direct effect) | -0.390 | -0.675 | -0.108 | 1.000 |
|  |  | Indirect effect | 0.023 | -0.048 | 0.116 | 1.000 |
|  |  | Total effect | -0.367 | -0.637 | -0.097 | 1.000 |
|  |  | Indirect effect (%) | -7.804 | -47.352 | 18.148 | 1.000 |
| 6 | Yamagata | a | 0.284 | 0.084 | 0.485 | 1.001 |
|  |  | b | 0.128 | 0.022 | 0.235 | 1.000 |
|  |  | c (Direct effect) | -0.863 | -1.103 | -0.622 | 1.001 |
|  |  | Indirect effect | 0.036 | 0.003 | 0.085 | 1.001 |
|  |  | Total effect | -0.826 | -1.064 | -0.587 | 1.002 |
|  |  | Indirect effect (%) | -4.520 | -11.133 | -0.423 | 1.001 |
| 7 | Fukushima | a | 0.052 | -0.090 | 0.196 | 1.000 |
|  |  | b | 0.407 | 0.097 | 0.739 | 1.000 |
|  |  | c (Direct effect) | -0.709 | -0.920 | -0.495 | 1.000 |
|  |  | Indirect effect | 0.021 | -0.039 | 0.094 | 1.000 |
|  |  | Total effect | -0.688 | -0.895 | -0.477 | 1.000 |
|  |  | Indirect effect (%) | -3.205 | -15.077 | 5.769 | 1.000 |
| 8 | Ibaraki | a | 0.170 | 0.060 | 0.279 | 1.000 |
|  |  | b | 0.516 | 0.048 | 0.980 | 1.001 |
|  |  | c (Direct effect) | -0.779 | -0.962 | -0.589 | 1.000 |
|  |  | Indirect effect | 0.090 | 0.004 | 0.211 | 1.001 |
|  |  | Total effect | -0.689 | -0.876 | -0.496 | 1.000 |
|  |  | Indirect effect (%) | -13.651 | -35.372 | -0.566 | 1.001 |
| 9 | Tochigi | a | -0.032 | -0.107 | 0.042 | 1.001 |
|  |  | b | -0.348 | -0.761 | 0.071 | 1.009 |
|  |  | c (Direct effect) | -0.409 | -0.561 | -0.256 | 1.000 |
|  |  | Indirect effect | 0.012 | -0.015 | 0.054 | 1.000 |
|  |  | Total effect | -0.397 | -0.547 | -0.246 | 1.000 |
|  |  | Indirect effect (%) | -3.140 | -14.562 | 3.935 | 1.000 |
| 10 | Gunma | a | 0.118 | -0.009 | 0.247 | 1.000 |
|  |  | b | 0.115 | -0.267 | 0.498 | 1.001 |
|  |  | c (Direct effect) | -0.432 | -0.656 | -0.212 | 1.001 |
|  |  | Indirect effect | 0.014 | -0.034 | 0.076 | 1.000 |
|  |  | Total effect | -0.418 | -0.642 | -0.194 | 1.000 |
|  |  | Indirect effect (%) | -4.131 | -23.820 | 8.259 | 1.000 |
| 11 | Saitama | a | -0.112 | -0.220 | -0.011 | 1.001 |
|  |  | b | 0.732 | 0.327 | 1.204 | 1.002 |
|  |  | c (Direct effect) | -0.412 | -0.523 | -0.295 | 1.000 |
|  |  | Indirect effect | -0.076 | -0.151 | -0.009 | 1.000 |
|  |  | Total effect | -0.489 | -0.601 | -0.372 | 1.000 |
|  |  | Indirect effect (%) | 15.562 | 2.011 | 30.130 | 1.000 |
| 12 | Chiba | a | -0.294 | -0.390 | -0.200 | 1.000 |
|  |  | b | -0.023 | -0.273 | 0.241 | 1.001 |
|  |  | c (Direct effect) | -0.538 | -0.658 | -0.415 | 1.000 |
|  |  | Indirect effect | 0.008 | -0.068 | 0.085 | 1.001 |
|  |  | Total effect | -0.530 | -0.635 | -0.420 | 1.000 |
|  |  | Indirect effect (%) | -1.597 | -17.225 | 12.576 | 1.001 |
| 13 | Tokyo | a | -0.234 | -0.299 | -0.169 | 1.003 |
|  |  | b | 0.187 | 0.057 | 0.307 | 1.003 |
|  |  | c (Direct effect) | -0.478 | -0.561 | -0.391 | 1.000 |
|  |  | Indirect effect | -0.044 | -0.077 | -0.013 | 1.002 |
|  |  | Total effect | -0.522 | -0.604 | -0.434 | 1.000 |
|  |  | Indirect effect (%) | 8.428 | 2.530 | 14.737 | 1.002 |
| 14 | Kanagawa | a | -0.251 | -0.341 | -0.163 | 1.000 |
|  |  | b | 0.424 | 0.126 | 0.714 | 1.002 |
|  |  | c (Direct effect) | -0.267 | -0.370 | -0.153 | 1.000 |
|  |  | Indirect effect | -0.104 | -0.177 | -0.034 | 1.002 |
|  |  | Total effect | -0.371 | -0.476 | -0.252 | 1.000 |
|  |  | Indirect effect (%) | 28.191 | 10.070 | 48.480 | 1.001 |
| 15 | Niigata | a | 0.117 | -0.065 | 0.298 | 1.001 |
|  |  | b | 0.323 | 0.129 | 0.526 | 1.009 |
|  |  | c (Direct effect) | -0.291 | -0.495 | -0.082 | 1.001 |
|  |  | Indirect effect | 0.038 | -0.021 | 0.113 | 1.000 |
|  |  | Total effect | -0.253 | -0.463 | -0.037 | 1.001 |
|  |  | Indirect effect (%) | -26.437 | -116.857 | 11.574 | 1.000 |
| 16 | Toyama | a | 0.152 | -0.001 | 0.307 | 1.000 |
|  |  | b | 1.066 | 0.719 | 1.461 | 1.000 |
|  |  | c (Direct effect) | -0.719 | -0.976 | -0.461 | 1.000 |
|  |  | Indirect effect | 0.162 | -0.001 | 0.347 | 1.000 |
|  |  | Total effect | -0.557 | -0.833 | -0.282 | 1.000 |
|  |  | Indirect effect (%) | -33.718 | -98.637 | 0.084 | 1.000 |
| 17 | Ishikawa | a | 0.043 | -0.100 | 0.188 | 1.000 |
|  |  | b | 1.003 | 0.705 | 1.330 | 1.000 |
|  |  | c (Direct effect) | -0.525 | -0.755 | -0.296 | 1.000 |
|  |  | Indirect effect | 0.042 | -0.102 | 0.189 | 1.000 |
|  |  | Total effect | -0.483 | -0.720 | -0.246 | 1.000 |
|  |  | Indirect effect (%) | -11.263 | -56.993 | 19.585 | 1.000 |
| 18 | Fukui | a | -0.075 | -0.217 | 0.070 | 1.000 |
|  |  | b | 0.533 | 0.174 | 0.888 | 1.000 |
|  |  | c (Direct effect) | -0.541 | -0.803 | -0.272 | 1.000 |
|  |  | Indirect effect | -0.039 | -0.134 | 0.039 | 1.000 |
|  |  | Total effect | -0.580 | -0.836 | -0.316 | 1.000 |
|  |  | Indirect effect (%) | 6.933 | -8.250 | 25.492 | 1.000 |
| 19 | Yamanashi | a | -0.029 | -0.186 | 0.134 | 1.000 |
|  |  | b | 0.412 | 0.162 | 0.673 | 1.003 |
|  |  | c (Direct effect) | -0.252 | -0.500 | -0.007 | 1.005 |
|  |  | Indirect effect | -0.012 | -0.087 | 0.059 | 1.000 |
|  |  | Total effect | -0.264 | -0.514 | -0.020 | 1.005 |
|  |  | Indirect effect (%) | 4.058 | -49.243 | 57.566 | 1.000 |
| 20 | Nagano | a | -0.029 | -0.210 | 0.159 | 1.000 |
|  |  | b | 0.739 | 0.522 | 0.998 | 1.000 |
|  |  | c (Direct effect) | -0.407 | -0.612 | -0.203 | 1.000 |
|  |  | Indirect effect | -0.022 | -0.165 | 0.114 | 1.000 |
|  |  | Total effect | -0.429 | -0.649 | -0.205 | 1.000 |
|  |  | Indirect effect (%) | 3.068 | -40.773 | 35.593 | 1.000 |
| 21 | Gifu | a | -0.168 | -0.201 | -0.136 | 1.000 |
|  |  | b | -0.229 | -1.129 | 0.614 | 1.002 |
|  |  | c (Direct effect) | -0.592 | -0.788 | -0.405 | 1.002 |
|  |  | Indirect effect | 0.038 | -0.104 | 0.193 | 1.002 |
|  |  | Total effect | -0.553 | -0.672 | -0.437 | 1.001 |
|  |  | Indirect effect (%) | -6.970 | -35.639 | 19.234 | 1.002 |
| 22 | Shizuoka | a | -0.083 | -0.147 | -0.018 | 1.000 |
|  |  | b | 1.795 | 1.155 | 2.507 | 1.000 |
|  |  | c (Direct effect) | -0.457 | -0.699 | -0.214 | 1.000 |
|  |  | Indirect effect | -0.150 | -0.296 | -0.030 | 1.000 |
|  |  | Total effect | -0.607 | -0.845 | -0.365 | 1.000 |
|  |  | Indirect effect (%) | 25.176 | 5.588 | 51.464 | 1.000 |
| 23 | Aichi | a | 0.007 | 0.005 | 0.009 | 1.000 |
|  |  | b | 0.132 | -164.666 | 187.510 | 1.004 |
|  |  | c (Direct effect) | -0.090 | -1.482 | 1.058 | 1.004 |
|  |  | Indirect effect | 0.030 | -1.081 | 1.421 | 1.004 |
|  |  | Total effect | -0.060 | -0.279 | 0.166 | 1.000 |
|  |  | Indirect effect (%) | 71.170 | -6025.068 | 5705.076 | 1.000 |
| 24 | Mie | a | -0.102 | -0.192 | -0.012 | 1.000 |
|  |  | b | 1.019 | 0.576 | 1.515 | 1.000 |
|  |  | c (Direct effect) | -0.397 | -0.624 | -0.169 | 1.000 |
|  |  | Indirect effect | -0.106 | -0.236 | -0.010 | 1.000 |
|  |  | Total effect | -0.503 | -0.722 | -0.283 | 1.000 |
|  |  | Indirect effect (%) | 21.731 | 2.211 | 51.470 | 1.000 |
| 25 | Shiga | a | -0.120 | -0.240 | 0.003 | 1.000 |
|  |  | b | 0.844 | 0.406 | 1.335 | 1.000 |
|  |  | c (Direct effect) | -0.390 | -0.615 | -0.159 | 1.000 |
|  |  | Indirect effect | -0.102 | -0.245 | 0.002 | 1.000 |
|  |  | Total effect | -0.492 | -0.699 | -0.284 | 1.000 |
|  |  | Indirect effect (%) | 21.468 | -0.517 | 54.525 | 1.000 |
| 26 | Kyoto | a | -0.273 | -0.388 | -0.156 | 1.000 |
|  |  | b | 0.286 | 0.122 | 0.461 | 1.000 |
|  |  | c (Direct effect) | -0.565 | -0.685 | -0.443 | 1.000 |
|  |  | Indirect effect | -0.078 | -0.143 | -0.029 | 1.000 |
|  |  | Total effect | -0.643 | -0.752 | -0.535 | 1.000 |
|  |  | Indirect effect (%) | 12.237 | 4.376 | 22.752 | 1.000 |
| 27 | Osaka | a | -0.139 | -0.222 | -0.055 | 1.000 |
|  |  | b | 1.661 | 1.263 | 2.102 | 1.000 |
|  |  | c (Direct effect) | -0.060 | -0.205 | 0.100 | 1.000 |
|  |  | Indirect effect | -0.230 | -0.384 | -0.089 | 1.000 |
|  |  | Total effect | -0.290 | -0.417 | -0.162 | 1.000 |
|  |  | Indirect effect (%) | 81.196 | 35.055 | 145.352 | 1.000 |
| 28 | Hyogo | a | -0.176 | -0.264 | -0.086 | 1.000 |
|  |  | b | 2.085 | 1.237 | 3.181 | 1.000 |
|  |  | c (Direct effect) | 0.094 | -0.137 | 0.387 | 1.000 |
|  |  | Indirect effect | -0.364 | -0.647 | -0.157 | 1.000 |
|  |  | Total effect | -0.270 | -0.412 | -0.126 | 1.000 |
|  |  | Indirect effect (%) | 146.188 | 58.356 | 311.930 | 1.000 |
| 29 | Nara | a | -0.055 | -0.228 | 0.120 | 1.000 |
|  |  | b | 1.308 | 0.871 | 1.884 | 1.000 |
|  |  | c (Direct effect) | -0.505 | -0.750 | -0.226 | 1.000 |
|  |  | Indirect effect | -0.077 | -0.340 | 0.147 | 1.000 |
|  |  | Total effect | -0.583 | -0.777 | -0.382 | 1.000 |
|  |  | Indirect effect (%) | 12.475 | -30.260 | 57.539 | 1.000 |
| 30 | Wakayama | a | 0.134 | -0.152 | 0.398 | 1.017 |
|  |  | b | 0.392 | 0.260 | 0.532 | 1.044 |
|  |  | c (Direct effect) | -0.089 | -0.348 | 0.170 | 1.011 |
|  |  | Indirect effect | 0.053 | -0.060 | 0.167 | 1.025 |
|  |  | Total effect | -0.036 | -0.318 | 0.247 | 1.005 |
|  |  | Indirect effect (%) | -75.084 | -676.977 | 706.280 | 1.000 |
| 31 | Tottori | a | 0.419 | 0.182 | 0.658 | 1.040 |
|  |  | b | 0.052 | -0.089 | 0.187 | 1.020 |
|  |  | c (Direct effect) | -0.239 | -0.474 | 0.028 | 1.041 |
|  |  | Indirect effect | 0.022 | -0.040 | 0.089 | 1.015 |
|  |  | Total effect | -0.217 | -0.458 | 0.044 | 1.054 |
|  |  | Indirect effect (%) | 6.188 | -116.987 | 71.016 | 1.000 |
| 32 | Shimane | a | 0.163 | -0.055 | 0.380 | 1.000 |
|  |  | b | 0.176 | 0.007 | 0.354 | 1.001 |
|  |  | c (Direct effect) | -0.464 | -0.797 | -0.140 | 1.000 |
|  |  | Indirect effect | 0.029 | -0.010 | 0.090 | 1.000 |
|  |  | Total effect | -0.435 | -0.770 | -0.108 | 1.000 |
|  |  | Indirect effect (%) | -5.819 | -39.301 | 2.896 | 1.000 |
| 33 | Okayama | a | 0.195 | 0.032 | 0.363 | 1.000 |
|  |  | b | 0.913 | 0.503 | 1.444 | 1.001 |
|  |  | c (Direct effect) | -0.180 | -0.405 | 0.042 | 1.000 |
|  |  | Indirect effect | 0.177 | 0.027 | 0.375 | 1.000 |
|  |  | Total effect | -0.002 | -0.227 | 0.227 | 1.000 |
|  |  | Indirect effect (%) | 3763.846 | -2486.156 | 2626.450 | 1.000 |
| 34 | Hiroshima | a | 0.065 | -0.088 | 0.217 | 1.000 |
|  |  | b | 0.108 | -0.164 | 0.388 | 1.000 |
|  |  | c (Direct effect) | -1.020 | -1.267 | -0.775 | 1.000 |
|  |  | Indirect effect | 0.007 | -0.021 | 0.047 | 1.000 |
|  |  | Total effect | -1.013 | -1.261 | -0.769 | 1.000 |
|  |  | Indirect effect (%) | -0.694 | -4.795 | 2.042 | 1.000 |
| 35 | Yamaguchi | a | 0.205 | 0.024 | 0.384 | 1.000 |
|  |  | b | 0.518 | 0.234 | 0.837 | 1.004 |
|  |  | c (Direct effect) | -0.374 | -0.668 | -0.090 | 1.003 |
|  |  | Indirect effect | 0.105 | 0.010 | 0.234 | 1.001 |
|  |  | Total effect | -0.268 | -0.556 | 0.012 | 1.002 |
|  |  | Indirect effect (%) | 184.684 | -340.045 | 115.560 | 1.000 |
| 36 | Tokushima | a | 0.124 | -0.123 | 0.369 | 1.000 |
|  |  | b | 0.350 | 0.188 | 0.516 | 1.000 |
|  |  | c (Direct effect) | -0.012 | -0.271 | 0.246 | 1.000 |
|  |  | Indirect effect | 0.044 | -0.043 | 0.141 | 1.000 |
|  |  | Total effect | 0.032 | -0.240 | 0.307 | 1.000 |
|  |  | Indirect effect (%) | -110.045 | -498.815 | 559.232 | 1.000 |
| 37 | Kagawa | a | 0.017 | -0.185 | 0.225 | 1.000 |
|  |  | b | 0.225 | 0.025 | 0.434 | 1.001 |
|  |  | c (Direct effect) | -0.609 | -0.868 | -0.358 | 1.001 |
|  |  | Indirect effect | 0.003 | -0.051 | 0.056 | 1.000 |
|  |  | Total effect | -0.606 | -0.867 | -0.354 | 1.001 |
|  |  | Indirect effect (%) | -0.666 | -10.992 | 8.760 | 1.000 |
| 38 | Ehime | a | 0.308 | 0.050 | 0.570 | 1.000 |
|  |  | b | 0.427 | 0.256 | 0.611 | 1.005 |
|  |  | c (Direct effect) | 0.243 | -0.016 | 0.502 | 1.000 |
|  |  | Indirect effect | 0.131 | 0.020 | 0.269 | 1.000 |
|  |  | Total effect | 0.375 | 0.096 | 0.660 | 1.000 |
|  |  | Indirect effect (%) | 38.385 | 5.832 | 104.799 | 1.000 |
| 39 | Kochi | a | 0.419 | 0.178 | 0.667 | 1.000 |
|  |  | b | 0.632 | 0.386 | 0.917 | 1.002 |
|  |  | c (Direct effect) | -0.795 | -1.100 | -0.503 | 1.003 |
|  |  | Indirect effect | 0.265 | 0.097 | 0.485 | 1.001 |
|  |  | Total effect | -0.529 | -0.812 | -0.248 | 1.001 |
|  |  | Indirect effect (%) | -57.217 | -147.593 | -15.530 | 1.000 |
| 40 | Fukuoka | a | -0.223 | -0.389 | -0.052 | 1.001 |
|  |  | b | -0.184 | -0.423 | 0.046 | 1.000 |
|  |  | c (Direct effect) | -0.962 | -1.165 | -0.762 | 1.000 |
|  |  | Indirect effect | 0.042 | -0.010 | 0.121 | 1.000 |
|  |  | Total effect | -0.921 | -1.116 | -0.729 | 1.000 |
|  |  | Indirect effect (%) | -4.592 | -13.766 | 1.116 | 1.000 |
| 41 | Saga | a | 0.208 | -0.087 | 0.508 | 1.000 |
|  |  | b | 0.218 | 0.108 | 0.332 | 1.000 |
|  |  | c (Direct effect) | -0.039 | -0.284 | 0.203 | 1.000 |
|  |  | Indirect effect | 0.045 | -0.019 | 0.125 | 1.001 |
|  |  | Total effect | 0.007 | -0.249 | 0.262 | 1.000 |
|  |  | Indirect effect (%) | 62.167 | -526.761 | 581.708 | 1.000 |
| 42 | Nagasaki | a | 0.389 | 0.083 | 0.678 | 1.024 |
|  |  | b | 0.085 | -0.003 | 0.174 | 1.008 |
|  |  | c (Direct effect) | -0.325 | -0.538 | -0.112 | 1.003 |
|  |  | Indirect effect | 0.033 | -0.002 | 0.086 | 1.003 |
|  |  | Total effect | -0.292 | -0.512 | -0.073 | 1.002 |
|  |  | Indirect effect (%) | -21.593 | -60.862 | 0.999 | 1.000 |
| 43 | Kumamoto | a | 0.214 | -0.062 | 0.487 | 1.000 |
|  |  | b | 0.191 | 0.072 | 0.313 | 1.001 |
|  |  | c (Direct effect) | -0.454 | -0.703 | -0.208 | 1.001 |
|  |  | Indirect effect | 0.041 | -0.011 | 0.111 | 1.000 |
|  |  | Total effect | -0.414 | -0.667 | -0.161 | 1.000 |
|  |  | Indirect effect (%) | -20.066 | -43.495 | 2.523 | 1.000 |
| 44 | Oita | a | 0.090 | -0.156 | 0.335 | 1.000 |
|  |  | b | 0.310 | 0.200 | 0.428 | 1.003 |
|  |  | c (Direct effect) | -0.377 | -0.575 | -0.178 | 1.000 |
|  |  | Indirect effect | 0.028 | -0.049 | 0.109 | 1.000 |
|  |  | Total effect | -0.350 | -0.560 | -0.137 | 1.000 |
|  |  | Indirect effect (%) | -10.862 | -55.087 | 13.175 | 1.000 |
| 45 | Miyazaki | a | 0.211 | -0.055 | 0.464 | 1.004 |
|  |  | b | 0.347 | 0.217 | 0.481 | 1.002 |
|  |  | c (Direct effect) | -0.278 | -0.576 | 0.014 | 1.001 |
|  |  | Indirect effect | 0.073 | -0.018 | 0.175 | 1.003 |
|  |  | Total effect | -0.205 | -0.514 | 0.098 | 1.001 |
|  |  | Indirect effect (%) | -62.759 | -419.927 | 337.752 | 1.000 |
| 46 | Kagoshima | a | 0.018 | -0.216 | 0.251 | 1.001 |
|  |  | b | 0.186 | -0.002 | 0.378 | 1.003 |
|  |  | c (Direct effect) | -0.234 | -0.559 | 0.089 | 1.001 |
|  |  | Indirect effect | 0.003 | -0.047 | 0.058 | 1.001 |
|  |  | Total effect | -0.231 | -0.558 | 0.096 | 1.000 |
|  |  | Indirect effect (%) | 10.145 | -77.989 | 71.044 | 1.000 |
| 47 | Okinawa | a | 0.074 | -0.102 | 0.247 | 1.000 |
|  |  | b | 0.391 | 0.283 | 0.502 | 1.000 |
|  |  | c (Direct effect) | -0.867 | -1.069 | -0.665 | 1.000 |
|  |  | Indirect effect | 0.029 | -0.040 | 0.100 | 1.000 |
|  |  | Total effect | -0.838 | -1.053 | -0.624 | 1.000 |
|  |  | Indirect effect (%) | -3.720 | -13.649 | 4.443 | 1.000 |

**Table S14. Effects of NPIs on mitigating the COVID-19 infection mediated by Aichi residents’ mobility during Phase 2**

| **Prefecture Number** | **Prefecture Name** | **Parameters** | **Mean** | **95% Credible Interval** | | **Rhat** |
| --- | --- | --- | --- | --- | --- | --- |
|  |  |  |  | **Low** | **High** |  |
| 1 | Hokkaido | a | -0.276 | -0.413 | -0.141 | 1.000 |
|  |  | b | 0.399 | 0.262 | 0.543 | 1.002 |
|  |  | c (Direct effect) | -0.065 | -0.163 | 0.038 | 1.001 |
|  |  | Indirect effect | -0.110 | -0.181 | -0.050 | 1.001 |
|  |  | Total effect | -0.175 | -0.267 | -0.079 | 1.000 |
|  |  | Indirect effect (%) | 67.485 | 29.155 | 137.774 | 1.000 |
| 2 | Aomori | a | - | - | - | - |
|  |  | b | - | - | - | - |
|  |  | c (Direct effect) | - | - | - | - |
|  |  | Indirect effect | - | - | - | - |
|  |  | Total effect | - | - | - | - |
|  |  | Indirect effect (%) | - | - | - | - |
| 3 | Iwate | a | - | - | - | - |
|  |  | b | - | - | - | - |
|  |  | c (Direct effect) | - | - | - | - |
|  |  | Indirect effect | - | - | - | - |
|  |  | Total effect | - | - | - | - |
|  |  | Indirect effect (%) | - | - | - | - |
| 4 | Miyagi | a | 0.144 | 0.034 | 0.252 | 1.000 |
|  |  | b | 0.617 | -0.117 | 1.651 | 1.003 |
|  |  | c (Direct effect) | -0.288 | -0.516 | -0.093 | 1.002 |
|  |  | Indirect effect | 0.097 | -0.011 | 0.316 | 1.002 |
|  |  | Total effect | -0.191 | -0.319 | -0.057 | 1.000 |
|  |  | Indirect effect (%) | -53.901 | -263.816 | 8.278 | 1.000 |
| 5 | Akita | a | - | - | - | - |
|  |  | b | - | - | - | - |
|  |  | c (Direct effect) | - | - | - | - |
|  |  | Indirect effect | - | - | - | - |
|  |  | Total effect | - | - | - | - |
|  |  | Indirect effect (%) | - | - | - | - |
| 6 | Yamagata | a | - | - | - | - |
|  |  | b | - | - | - | - |
|  |  | c (Direct effect) | - | - | - | - |
|  |  | Indirect effect | - | - | - | - |
|  |  | Total effect | - | - | - | - |
|  |  | Indirect effect (%) | - | - | - | - |
| 7 | Fukushima | a | 0.685 | 0.382 | 0.995 | 1.000 |
|  |  | b | 0.175 | 0.020 | 0.336 | 1.002 |
|  |  | c (Direct effect) | -0.434 | -0.616 | -0.259 | 1.000 |
|  |  | Indirect effect | 0.119 | 0.013 | 0.242 | 1.002 |
|  |  | Total effect | -0.315 | -0.485 | -0.147 | 1.000 |
|  |  | Indirect effect (%) | -44.391 | -123.138 | -3.384 | 1.000 |
| 8 | Ibaraki | a | 0.485 | 0.304 | 0.664 | 1.001 |
|  |  | b | -0.028 | -0.146 | 0.089 | 1.003 |
|  |  | c (Direct effect) | -0.193 | -0.327 | -0.058 | 1.001 |
|  |  | Indirect effect | -0.014 | -0.074 | 0.044 | 1.002 |
|  |  | Total effect | -0.207 | -0.339 | -0.074 | 1.000 |
|  |  | Indirect effect (%) | 6.092 | -30.345 | 42.132 | 1.000 |
| 9 | Tochigi | a | 0.366 | 0.224 | 0.517 | 1.000 |
|  |  | b | -0.424 | -1.594 | 0.387 | 1.006 |
|  |  | c (Direct effect) | 0.023 | -0.292 | 0.441 | 1.004 |
|  |  | Indirect effect | -0.148 | -0.536 | 0.146 | 1.005 |
|  |  | Total effect | -0.125 | -0.280 | 0.043 | 1.004 |
|  |  | Indirect effect (%) | 258.627 | -821.199 | 1198.558 | 1.000 |
| 10 | Gunma | a | 0.085 | -0.026 | 0.199 | 1.019 |
|  |  | b | -5.898 | -107.013 | 9.017 | 1.354 |
|  |  | c (Direct effect) | -0.043 | -1.226 | 5.309 | 1.130 |
|  |  | Indirect effect | -0.251 | -5.605 | 0.934 | 1.130 |
|  |  | Total effect | -0.294 | -0.414 | -0.171 | 1.000 |
|  |  | Indirect effect (%) | 86.385 | -359.307 | 1929.798 | 1.114 |
| 11 | Saitama | a | -0.029 | -0.155 | 0.096 | 1.001 |
|  |  | b | 0.094 | -0.014 | 0.211 | 1.008 |
|  |  | c (Direct effect) | -0.017 | -0.089 | 0.056 | 1.001 |
|  |  | Indirect effect | -0.003 | -0.020 | 0.011 | 1.002 |
|  |  | Total effect | -0.019 | -0.092 | 0.053 | 1.001 |
|  |  | Indirect effect (%) | -0.652 | -187.142 | 199.407 | 1.000 |
| 12 | Chiba | a | -0.040 | -0.159 | 0.083 | 1.002 |
|  |  | b | 0.099 | -0.022 | 0.235 | 1.007 |
|  |  | c (Direct effect) | -0.035 | -0.112 | 0.042 | 1.001 |
|  |  | Indirect effect | -0.004 | -0.025 | 0.009 | 1.001 |
|  |  | Total effect | -0.040 | -0.116 | 0.038 | 1.001 |
|  |  | Indirect effect (%) | -23.345 | -127.718 | 161.948 | 1.000 |
| 13 | Tokyo | a | -0.012 | -0.075 | 0.051 | 1.006 |
|  |  | b | 0.073 | -0.019 | 0.165 | 1.004 |
|  |  | c (Direct effect) | 0.004 | -0.046 | 0.053 | 1.005 |
|  |  | Indirect effect | -0.001 | -0.007 | 0.005 | 1.008 |
|  |  | Total effect | 0.003 | -0.048 | 0.053 | 1.004 |
|  |  | Indirect effect (%) | 3.439 | -135.563 | 128.725 | 1.000 |
| 14 | Kanagawa | a | -0.123 | -0.232 | -0.015 | 1.020 |
|  |  | b | -0.077 | -0.251 | 0.112 | 1.058 |
|  |  | c (Direct effect) | -0.113 | -0.190 | -0.036 | 1.003 |
|  |  | Indirect effect | 0.010 | -0.014 | 0.055 | 1.072 |
|  |  | Total effect | -0.103 | -0.179 | -0.029 | 1.021 |
|  |  | Indirect effect (%) | -10.582 | -151.759 | 17.171 | 1.000 |
| 15 | Niigata | a | - | - | - | - |
|  |  | b | - | - | - | - |
|  |  | c (Direct effect) | - | - | - | - |
|  |  | Indirect effect | - | - | - | - |
|  |  | Total effect | - | - | - | - |
|  |  | Indirect effect (%) | - | - | - | - |
| 16 | Toyama | a | -0.404 | -0.596 | -0.210 | 1.000 |
|  |  | b | 0.184 | 0.044 | 0.327 | 1.002 |
|  |  | c (Direct effect) | -0.503 | -0.714 | -0.293 | 1.001 |
|  |  | Indirect effect | -0.074 | -0.150 | -0.015 | 1.001 |
|  |  | Total effect | -0.577 | -0.803 | -0.354 | 1.001 |
|  |  | Indirect effect (%) | 12.977 | 2.946 | 25.938 | 1.000 |
| 17 | Ishikawa | a | -0.267 | -0.387 | -0.146 | 1.000 |
|  |  | b | 0.326 | 0.150 | 0.510 | 1.002 |
|  |  | c (Direct effect) | -0.222 | -0.359 | -0.083 | 1.000 |
|  |  | Indirect effect | -0.087 | -0.157 | -0.033 | 1.001 |
|  |  | Total effect | -0.308 | -0.443 | -0.174 | 1.000 |
|  |  | Indirect effect (%) | 29.371 | 10.496 | 58.120 | 1.000 |
| 18 | Fukui | a | - | - | - | - |
|  |  | b | - | - | - | - |
|  |  | c (Direct effect) | - | - | - | - |
|  |  | Indirect effect | - | - | - | - |
|  |  | Total effect | - | - | - | - |
|  |  | Indirect effect (%) | - | - | - | - |
| 19 | Yamanashi | a | -0.252 | -0.512 | 0.005 | 1.000 |
|  |  | b | 0.257 | 0.029 | 0.524 | 1.000 |
|  |  | c (Direct effect) | -0.230 | -0.455 | -0.004 | 1.000 |
|  |  | Indirect effect | -0.063 | -0.166 | 0.006 | 1.000 |
|  |  | Total effect | -0.293 | -0.522 | -0.067 | 1.000 |
|  |  | Indirect effect (%) | 25.481 | -3.638 | 85.155 | 1.000 |
| 20 | Nagano | a | - | - | - | - |
|  |  | b | - | - | - | - |
|  |  | c (Direct effect) | - | - | - | - |
|  |  | Indirect effect | - | - | - | - |
|  |  | Total effect | - | - | - | - |
|  |  | Indirect effect (%) | - | - | - | - |
| 21 | Gifu | a | -0.092 | -0.114 | -0.069 | 1.000 |
|  |  | b | 9.614 | 5.289 | 18.716 | 1.004 |
|  |  | c (Direct effect) | 0.655 | 0.171 | 1.623 | 1.004 |
|  |  | Indirect effect | -0.900 | -1.882 | -0.426 | 1.004 |
|  |  | Total effect | -0.245 | -0.360 | -0.126 | 1.000 |
|  |  | Indirect effect (%) | 392.751 | 160.057 | 925.245 | 1.001 |
| 22 | Shizuoka | a | 0.099 | 0.050 | 0.149 | 1.000 |
|  |  | b | 2.824 | 1.914 | 3.874 | 1.001 |
|  |  | c (Direct effect) | -0.279 | -0.449 | -0.112 | 1.001 |
|  |  | Indirect effect | 0.280 | 0.128 | 0.463 | 1.001 |
|  |  | Total effect | 0.001 | -0.158 | 0.159 | 1.000 |
|  |  | Indirect effect (%) | 2686.480 | -5586.218 | 5379.571 | 1.000 |
| 23 | Aichi | a | -0.002 | -0.004 | 0.000 | 1.000 |
|  |  | b | -40.948 | -50.807 | -31.937 | 1.000 |
|  |  | c (Direct effect) | -0.099 | -0.175 | -0.020 | 1.000 |
|  |  | Indirect effect | 0.084 | -0.005 | 0.172 | 1.000 |
|  |  | Total effect | -0.015 | -0.077 | 0.047 | 1.000 |
|  |  | Indirect effect (%) | -391.588 | -4290.523 | 4263.943 | 1.000 |
| 24 | Mie | a | -0.181 | -0.219 | -0.143 | 1.000 |
|  |  | b | 2.331 | 1.446 | 3.671 | 1.001 |
|  |  | c (Direct effect) | 0.067 | -0.132 | 0.355 | 1.001 |
|  |  | Indirect effect | -0.422 | -0.693 | -0.246 | 1.000 |
|  |  | Total effect | -0.355 | -0.452 | -0.257 | 1.000 |
|  |  | Indirect effect (%) | 121.246 | 66.997 | 213.227 | 1.001 |
| 25 | Shiga | a | -0.031 | -0.180 | 0.126 | 1.000 |
|  |  | b | 0.598 | 0.284 | 0.953 | 1.001 |
|  |  | c (Direct effect) | -0.077 | -0.240 | 0.093 | 1.000 |
|  |  | Indirect effect | -0.019 | -0.120 | 0.074 | 1.000 |
|  |  | Total effect | -0.096 | -0.269 | 0.082 | 1.000 |
|  |  | Indirect effect (%) | 41.878 | -318.682 | 387.374 | 1.000 |
| 26 | Kyoto | a | -0.043 | -0.143 | 0.055 | 1.001 |
|  |  | b | -0.006 | -0.152 | 0.144 | 1.001 |
|  |  | c (Direct effect) | -0.289 | -0.383 | -0.194 | 1.000 |
|  |  | Indirect effect | 0.000 | -0.011 | 0.011 | 1.000 |
|  |  | Total effect | -0.289 | -0.382 | -0.194 | 1.000 |
|  |  | Indirect effect (%) | -0.038 | -4.106 | 3.970 | 1.000 |
| 27 | Osaka | a | 0.027 | -0.062 | 0.116 | 1.001 |
|  |  | b | 0.544 | 0.384 | 0.711 | 1.004 |
|  |  | c (Direct effect) | -0.125 | -0.188 | -0.060 | 1.000 |
|  |  | Indirect effect | 0.014 | -0.035 | 0.063 | 1.001 |
|  |  | Total effect | -0.110 | -0.178 | -0.042 | 1.001 |
|  |  | Indirect effect (%) | -31.060 | -106.467 | 27.483 | 1.000 |
| 28 | Hyogo | a | 0.060 | -0.036 | 0.155 | 1.000 |
|  |  | b | 0.598 | 0.372 | 0.847 | 1.000 |
|  |  | c (Direct effect) | -0.180 | -0.251 | -0.109 | 1.000 |
|  |  | Indirect effect | 0.035 | -0.021 | 0.098 | 1.000 |
|  |  | Total effect | -0.145 | -0.208 | -0.081 | 1.000 |
|  |  | Indirect effect (%) | -27.852 | -92.038 | 13.146 | 1.000 |
| 29 | Nara | a | - | - | - | - |
|  |  | b | - | - | - | - |
|  |  | c (Direct effect) | - | - | - | - |
|  |  | Indirect effect | - | - | - | - |
|  |  | Total effect | - | - | - | - |
|  |  | Indirect effect (%) | - | - | - | - |
| 30 | Wakayama | a | - | - | - | - |
|  |  | b | - | - | - | - |
|  |  | c (Direct effect) | - | - | - | - |
|  |  | Indirect effect | - | - | - | - |
|  |  | Total effect | - | - | - | - |
|  |  | Indirect effect (%) | - | - | - | - |
| 31 | Tottori | a | - | - | - | - |
|  |  | b | - | - | - | - |
|  |  | c (Direct effect) | - | - | - | - |
|  |  | Indirect effect | - | - | - | - |
|  |  | Total effect | - | - | - | - |
|  |  | Indirect effect (%) | - | - | - | - |
| 32 | Shimane | a | - | - | - | - |
|  |  | b | - | - | - | - |
|  |  | c (Direct effect) | - | - | - | - |
|  |  | Indirect effect | - | - | - | - |
|  |  | Total effect | - | - | - | - |
|  |  | Indirect effect (%) | - | - | - | - |
| 33 | Okayama | a | -0.585 | -0.733 | -0.440 | 1.000 |
|  |  | b | 0.254 | -0.101 | 0.701 | 1.007 |
|  |  | c (Direct effect) | -0.193 | -0.453 | 0.092 | 1.005 |
|  |  | Indirect effect | -0.144 | -0.384 | 0.062 | 1.006 |
|  |  | Total effect | -0.336 | -0.493 | -0.197 | 1.002 |
|  |  | Indirect effect (%) | 45.317 | -19.083 | 136.107 | 1.005 |
| 34 | Hiroshima | a | -0.457 | -0.573 | -0.342 | 1.000 |
|  |  | b | 0.675 | 0.443 | 0.931 | 1.000 |
|  |  | c (Direct effect) | -0.027 | -0.188 | 0.148 | 1.000 |
|  |  | Indirect effect | -0.308 | -0.452 | -0.189 | 1.000 |
|  |  | Total effect | -0.335 | -0.452 | -0.214 | 1.000 |
|  |  | Indirect effect (%) | 94.993 | 53.049 | 159.353 | 1.000 |
| 35 | Yamaguchi | a | - | - | - | - |
|  |  | b | - | - | - | - |
|  |  | c (Direct effect) | - | - | - | - |
|  |  | Indirect effect | - | - | - | - |
|  |  | Total effect | - | - | - | - |
|  |  | Indirect effect (%) | - | - | - | - |
| 36 | Tokushima | a | - | - | - | - |
|  |  | b | - | - | - | - |
|  |  | c (Direct effect) | - | - | - | - |
|  |  | Indirect effect | - | - | - | - |
|  |  | Total effect | - | - | - | - |
|  |  | Indirect effect (%) | - | - | - | - |
| 37 | Kagawa | a | -0.456 | -0.728 | -0.184 | 1.001 |
|  |  | b | 0.402 | 0.282 | 0.533 | 1.010 |
|  |  | c (Direct effect) | -0.396 | -0.557 | -0.230 | 1.001 |
|  |  | Indirect effect | -0.182 | -0.310 | -0.070 | 1.003 |
|  |  | Total effect | -0.578 | -0.763 | -0.396 | 1.002 |
|  |  | Indirect effect (%) | 31.540 | 13.923 | 51.020 | 1.001 |
| 38 | Ehime | a | -0.248 | -0.483 | -0.016 | 1.000 |
|  |  | b | 0.193 | 0.075 | 0.319 | 1.001 |
|  |  | c (Direct effect) | -0.392 | -0.574 | -0.210 | 1.000 |
|  |  | Indirect effect | -0.047 | -0.106 | -0.002 | 1.000 |
|  |  | Total effect | -0.439 | -0.629 | -0.251 | 1.000 |
|  |  | Indirect effect (%) | 10.927 | 0.614 | 25.562 | 1.000 |
| 39 | Kochi | a | -0.712 | -1.077 | -0.345 | 1.000 |
|  |  | b | 0.244 | 0.119 | 0.375 | 1.000 |
|  |  | c (Direct effect) | -0.249 | -0.534 | 0.038 | 1.000 |
|  |  | Indirect effect | -0.173 | -0.316 | -0.064 | 1.000 |
|  |  | Total effect | -0.422 | -0.704 | -0.138 | 1.000 |
|  |  | Indirect effect (%) | 42.475 | 14.337 | 120.425 | 1.000 |
| 40 | Fukuoka | a | -0.240 | -0.362 | -0.117 | 1.000 |
|  |  | b | 0.505 | 0.249 | 0.802 | 1.001 |
|  |  | c (Direct effect) | -0.176 | -0.291 | -0.040 | 1.001 |
|  |  | Indirect effect | -0.122 | -0.241 | -0.043 | 1.001 |
|  |  | Total effect | -0.298 | -0.383 | -0.214 | 1.000 |
|  |  | Indirect effect (%) | 41.596 | 14.375 | 85.229 | 1.001 |
| 41 | Saga | a | -0.669 | -1.097 | -0.237 | 1.000 |
|  |  | b | 0.246 | 0.152 | 0.356 | 1.002 |
|  |  | c (Direct effect) | -0.301 | -0.537 | -0.057 | 1.000 |
|  |  | Indirect effect | -0.165 | -0.311 | -0.050 | 1.001 |
|  |  | Total effect | -0.466 | -0.704 | -0.219 | 1.000 |
|  |  | Indirect effect (%) | 37.312 | 11.617 | 78.436 | 1.000 |
| 42 | Nagasaki | a | -0.805 | -1.269 | -0.334 | 1.000 |
|  |  | b | 0.113 | 0.030 | 0.199 | 1.000 |
|  |  | c (Direct effect) | -0.103 | -0.371 | 0.168 | 1.000 |
|  |  | Indirect effect | -0.091 | -0.194 | -0.018 | 1.000 |
|  |  | Total effect | -0.194 | -0.446 | 0.066 | 1.000 |
|  |  | Indirect effect (%) | 24.816 | -291.798 | 395.001 | 1.000 |
| 43 | Kumamoto | a | -0.004 | -0.246 | 0.248 | 1.001 |
|  |  | b | 0.438 | 0.200 | 0.721 | 1.014 |
|  |  | c (Direct effect) | -0.329 | -0.458 | -0.195 | 1.001 |
|  |  | Indirect effect | -0.006 | -0.126 | 0.097 | 1.001 |
|  |  | Total effect | -0.335 | -0.475 | -0.193 | 1.000 |
|  |  | Indirect effect (%) | -0.237 | -41.327 | 33.322 | 1.001 |
| 44 | Oita | a | - | - | - | - |
|  |  | b | - | - | - | - |
|  |  | c (Direct effect) | - | - | - | - |
|  |  | Indirect effect | - | - | - | - |
|  |  | Total effect | - | - | - | - |
|  |  | Indirect effect (%) | - | - | - | - |
| 45 | Miyazaki | a | -0.600 | -1.015 | -0.173 | 1.001 |
|  |  | b | 0.186 | 0.061 | 0.329 | 1.001 |
|  |  | c (Direct effect) | -0.319 | -0.616 | -0.020 | 1.000 |
|  |  | Indirect effect | -0.114 | -0.264 | -0.018 | 1.001 |
|  |  | Total effect | -0.433 | -0.709 | -0.153 | 1.000 |
|  |  | Indirect effect (%) | 30.556 | 3.765 | 87.190 | 1.000 |
| 46 | Kagoshima | a | -0.821 | -1.132 | -0.506 | 1.000 |
|  |  | b | 0.225 | 0.095 | 0.353 | 1.007 |
|  |  | c (Direct effect) | -0.377 | -0.565 | -0.188 | 1.000 |
|  |  | Indirect effect | -0.184 | -0.316 | -0.071 | 1.005 |
|  |  | Total effect | -0.561 | -0.759 | -0.357 | 1.002 |
|  |  | Indirect effect (%) | 33.104 | 13.727 | 55.955 | 1.003 |
| 47 | Okinawa | a | -0.025 | -0.245 | 0.188 | 1.002 |
|  |  | b | 0.153 | 0.064 | 0.265 | 1.002 |
|  |  | c (Direct effect) | -0.038 | -0.162 | 0.089 | 1.004 |
|  |  | Indirect effect | -0.004 | -0.043 | 0.029 | 1.002 |
|  |  | Total effect | -0.042 | -0.169 | 0.085 | 1.004 |
|  |  | Indirect effect (%) | 44.229 | -245.801 | 259.219 | 1.000 |

Prefectures where NPIs were not implemented are represented by "-".

**Table S15. Effects of NPIs on mitigating the COVID-19 infection mediated by Aichi residents’ mobility during Phase 3**

| **Prefecture Number** | **Prefecture Name** | **Parameters** | **Mean** | **95% Credible Interval** | | **Rhat** |
| --- | --- | --- | --- | --- | --- | --- |
|  |  |  |  | **Low** | **High** |  |
| 1 | Hokkaido | a | -0.128 | -0.320 | 0.075 | 1.000 |
|  |  | b | 0.002 | -0.199 | 0.209 | 1.001 |
|  |  | c (Direct effect) | -0.209 | -0.337 | -0.080 | 1.000 |
|  |  | Indirect effect | 0.001 | -0.039 | 0.045 | 1.001 |
|  |  | Total effect | -0.208 | -0.332 | -0.084 | 1.000 |
|  |  | Indirect effect (%) | -0.700 | -26.570 | 21.992 | 1.001 |
| 2 | Aomori | a | -0.161 | -0.556 | 0.234 | 1.000 |
|  |  | b | 0.333 | 0.256 | 0.418 | 1.004 |
|  |  | c (Direct effect) | -0.018 | -0.190 | 0.157 | 1.000 |
|  |  | Indirect effect | -0.054 | -0.190 | 0.079 | 1.000 |
|  |  | Total effect | -0.071 | -0.274 | 0.132 | 1.000 |
|  |  | Indirect effect (%) | -3.230 | -540.983 | 624.441 | 1.000 |
| 3 | Iwate | a | - | - | - | - |
|  |  | b | - | - | - | - |
|  |  | c (Direct effect) | - | - | - | - |
|  |  | Indirect effect | - | - | - | - |
|  |  | Total effect | - | - | - | - |
|  |  | Indirect effect (%) | - | - | - | - |
| 4 | Miyagi | a | - | - | - | - |
|  |  | b | - | - | - | - |
|  |  | c (Direct effect) | - | - | - | - |
|  |  | Indirect effect | - | - | - | - |
|  |  | Total effect | - | - | - | - |
|  |  | Indirect effect (%) | - | - | - | - |
| 5 | Akita | a | - | - | - | - |
|  |  | b | - | - | - | - |
|  |  | c (Direct effect) | - | - | - | - |
|  |  | Indirect effect | - | - | - | - |
|  |  | Total effect | - | - | - | - |
|  |  | Indirect effect (%) | - | - | - | - |
| 6 | Yamagata | a | 0.668 | 0.279 | 1.063 | 1.003 |
|  |  | b | 0.293 | 0.235 | 0.367 | 1.006 |
|  |  | c (Direct effect) | -0.397 | -0.592 | -0.210 | 1.003 |
|  |  | Indirect effect | 0.197 | 0.078 | 0.348 | 1.005 |
|  |  | Total effect | -0.200 | -0.406 | 0.003 | 1.001 |
|  |  | Indirect effect (%) | -142.315 | -794.668 | 250.648 | 1.000 |
| 7 | Fukushima | a | -0.228 | -0.549 | 0.097 | 1.000 |
|  |  | b | 0.001 | -0.148 | 0.126 | 1.001 |
|  |  | c (Direct effect) | -0.342 | -0.523 | -0.162 | 1.000 |
|  |  | Indirect effect | 0.002 | -0.037 | 0.052 | 1.001 |
|  |  | Total effect | -0.340 | -0.510 | -0.170 | 1.000 |
|  |  | Indirect effect (%) | -0.302 | -15.747 | 12.687 | 1.001 |
| 8 | Ibaraki | a | -0.082 | -0.304 | 0.151 | 1.009 |
|  |  | b | 0.472 | 0.402 | 0.539 | 1.024 |
|  |  | c (Direct effect) | -0.118 | -0.264 | 0.023 | 1.003 |
|  |  | Indirect effect | -0.038 | -0.144 | 0.071 | 1.008 |
|  |  | Total effect | -0.157 | -0.334 | 0.017 | 1.003 |
|  |  | Indirect effect (%) | 84.970 | -142.923 | 164.815 | 1.000 |
| 9 | Tochigi | a | -0.163 | -0.354 | 0.037 | 1.000 |
|  |  | b | 0.090 | -0.188 | 0.299 | 1.002 |
|  |  | c (Direct effect) | -0.233 | -0.362 | -0.103 | 1.001 |
|  |  | Indirect effect | -0.012 | -0.066 | 0.042 | 1.003 |
|  |  | Total effect | -0.245 | -0.362 | -0.127 | 1.000 |
|  |  | Indirect effect (%) | 5.531 | -17.656 | 31.192 | 1.002 |
| 10 | Gunma | a | -0.385 | -0.503 | -0.268 | 1.170 |
|  |  | b | 0.228 | -5.713 | 5.460 | 3.046 |
|  |  | c (Direct effect) | -0.168 | -2.864 | 2.149 | 3.022 |
|  |  | Indirect effect | 0.002 | -2.318 | 2.726 | 3.029 |
|  |  | Total effect | -0.167 | -0.282 | -0.048 | 1.014 |
|  |  | Indirect effect (%) | 12796.926 | -3234.990 | 2043.387 | 1.000 |
| 11 | Saitama | a | 0.074 | -0.138 | 0.291 | 1.002 |
|  |  | b | 0.391 | 0.335 | 0.455 | 1.018 |
|  |  | c (Direct effect) | -0.307 | -0.418 | -0.197 | 1.001 |
|  |  | Indirect effect | 0.029 | -0.054 | 0.114 | 1.002 |
|  |  | Total effect | -0.278 | -0.417 | -0.141 | 1.001 |
|  |  | Indirect effect (%) | -14.371 | -66.775 | 15.610 | 1.000 |
| 12 | Chiba | a | -0.078 | -0.208 | 0.048 | 1.000 |
|  |  | b | 0.526 | 0.408 | 0.679 | 1.002 |
|  |  | c (Direct effect) | -0.283 | -0.401 | -0.165 | 1.003 |
|  |  | Indirect effect | -0.041 | -0.116 | 0.025 | 1.000 |
|  |  | Total effect | -0.325 | -0.455 | -0.195 | 1.002 |
|  |  | Indirect effect (%) | 12.291 | -9.537 | 33.736 | 1.001 |
| 13 | Tokyo | a | 0.064 | -0.038 | 0.161 | 1.008 |
|  |  | b | 0.302 | 0.192 | 0.415 | 1.029 |
|  |  | c (Direct effect) | -0.321 | -0.428 | -0.214 | 1.009 |
|  |  | Indirect effect | 0.019 | -0.011 | 0.052 | 1.009 |
|  |  | Total effect | -0.302 | -0.412 | -0.190 | 1.011 |
|  |  | Indirect effect (%) | -6.938 | -22.137 | 3.480 | 1.010 |
| 14 | Kanagawa | a | 0.005 | -0.164 | 0.173 | 1.001 |
|  |  | b | 0.401 | 0.312 | 0.499 | 1.005 |
|  |  | c (Direct effect) | -0.330 | -0.449 | -0.206 | 1.002 |
|  |  | Indirect effect | 0.002 | -0.068 | 0.069 | 1.000 |
|  |  | Total effect | -0.328 | -0.464 | -0.191 | 1.002 |
|  |  | Indirect effect (%) | -1.663 | -27.599 | 18.501 | 1.000 |
| 15 | Niigata | a | -0.221 | -0.524 | 0.084 | 1.000 |
|  |  | b | 0.266 | 0.179 | 0.367 | 1.003 |
|  |  | c (Direct effect) | -0.275 | -0.427 | -0.124 | 1.001 |
|  |  | Indirect effect | -0.060 | -0.154 | 0.021 | 1.000 |
|  |  | Total effect | -0.335 | -0.488 | -0.182 | 1.001 |
|  |  | Indirect effect (%) | 17.921 | -7.579 | 47.684 | 1.001 |
| 16 | Toyama | a | - | - | - | - |
|  |  | b | - | - | - | - |
|  |  | c (Direct effect) | - | - | - | - |
|  |  | Indirect effect | - | - | - | - |
|  |  | Total effect | - | - | - | - |
|  |  | Indirect effect (%) | - | - | - | - |
| 17 | Ishikawa | a | -0.366 | -0.515 | -0.206 | 1.000 |
|  |  | b | -0.577 | -1.062 | -0.229 | 1.002 |
|  |  | c (Direct effect) | -0.726 | -0.971 | -0.514 | 1.001 |
|  |  | Indirect effect | 0.214 | 0.065 | 0.443 | 1.002 |
|  |  | Total effect | -0.512 | -0.666 | -0.358 | 1.000 |
|  |  | Indirect effect (%) | -43.335 | -99.754 | -12.024 | 1.001 |
| 18 | Fukui | a | - | - | - | - |
|  |  | b | - | - | - | - |
|  |  | c (Direct effect) | - | - | - | - |
|  |  | Indirect effect | - | - | - | - |
|  |  | Total effect | - | - | - | - |
|  |  | Indirect effect (%) | - | - | - | - |
| 19 | Yamanashi | a | - | - | - | - |
|  |  | b | - | - | - | - |
|  |  | c (Direct effect) | - | - | - | - |
|  |  | Indirect effect | - | - | - | - |
|  |  | Total effect | - | - | - | - |
|  |  | Indirect effect (%) | - | - | - | - |
| 20 | Nagano | a | -0.048 | -0.315 | 0.219 | 1.001 |
|  |  | b | 0.244 | 0.183 | 0.330 | 1.007 |
|  |  | c (Direct effect) | -0.200 | -0.322 | -0.076 | 1.001 |
|  |  | Indirect effect | -0.011 | -0.078 | 0.055 | 1.001 |
|  |  | Total effect | -0.212 | -0.346 | -0.078 | 1.000 |
|  |  | Indirect effect (%) | 2.996 | -44.361 | 36.009 | 1.000 |
| 21 | Gifu | a | -0.145 | -0.181 | -0.106 | 1.001 |
|  |  | b | 14.635 | 7.015 | 27.587 | 1.003 |
|  |  | c (Direct effect) | 1.757 | 0.525 | 3.588 | 1.003 |
|  |  | Indirect effect | -2.125 | -4.026 | -0.842 | 1.003 |
|  |  | Total effect | -0.367 | -0.620 | -0.109 | 1.000 |
|  |  | Indirect effect (%) | 826.219 | 236.021 | 1666.063 | 1.000 |
| 22 | Shizuoka | a | -0.040 | -0.129 | 0.049 | 1.000 |
|  |  | b | 1.573 | 1.277 | 1.910 | 1.003 |
|  |  | c (Direct effect) | -0.212 | -0.349 | -0.072 | 1.000 |
|  |  | Indirect effect | -0.063 | -0.206 | 0.075 | 1.000 |
|  |  | Total effect | -0.274 | -0.451 | -0.097 | 1.000 |
|  |  | Indirect effect (%) | 14.609 | -54.598 | 64.119 | 1.000 |
| 23 | Aichi | a | 0.008 | -0.001 | 0.016 | 1.506 |
|  |  | b | 39.588 | -27.032 | 248.686 | 13.978 |
|  |  | c (Direct effect) | -0.996 | -4.003 | -0.037 | 8.065 |
|  |  | Indirect effect | 0.668 | -0.292 | 3.649 | 8.028 |
|  |  | Total effect | -0.328 | -0.458 | -0.171 | 1.014 |
|  |  | Indirect effect (%) | -208.389 | -1263.172 | 87.045 | 3.894 |
| 24 | Mie | a | -0.238 | -0.298 | -0.174 | 1.084 |
|  |  | b | 5.829 | 4.541 | 7.357 | 1.032 |
|  |  | c (Direct effect) | 1.202 | 0.792 | 1.683 | 1.058 |
|  |  | Indirect effect | -1.391 | -1.896 | -0.945 | 1.064 |
|  |  | Total effect | -0.189 | -0.344 | -0.044 | 1.010 |
|  |  | Indirect effect (%) | 809.065 | 368.853 | 2399.832 | 1.000 |
| 25 | Shiga | a | - | - | - | - |
|  |  | b | - | - | - | - |
|  |  | c (Direct effect) | - | - | - | - |
|  |  | Indirect effect | - | - | - | - |
|  |  | Total effect | - | - | - | - |
|  |  | Indirect effect (%) | - | - | - | - |
| 26 | Kyoto | a | -0.115 | -0.289 | 0.062 | 1.003 |
|  |  | b | 0.254 | 0.148 | 0.388 | 1.003 |
|  |  | c (Direct effect) | -0.253 | -0.352 | -0.151 | 1.002 |
|  |  | Indirect effect | -0.030 | -0.086 | 0.015 | 1.001 |
|  |  | Total effect | -0.283 | -0.380 | -0.185 | 1.002 |
|  |  | Indirect effect (%) | 10.502 | -5.738 | 31.144 | 1.001 |
| 27 | Osaka | a | -0.068 | -0.217 | 0.077 | 1.004 |
|  |  | b | 0.478 | 0.355 | 0.667 | 1.008 |
|  |  | c (Direct effect) | -0.214 | -0.312 | -0.111 | 1.005 |
|  |  | Indirect effect | -0.033 | -0.114 | 0.036 | 1.004 |
|  |  | Total effect | -0.247 | -0.356 | -0.137 | 1.008 |
|  |  | Indirect effect (%) | 12.475 | -19.808 | 42.957 | 1.001 |
| 28 | Hyogo | a | -0.314 | -0.489 | -0.073 | 1.160 |
|  |  | b | 0.194 | -7.822 | 10.200 | 2.664 |
|  |  | c (Direct effect) | -0.259 | -3.575 | 4.356 | 2.432 |
|  |  | Indirect effect | -0.035 | -4.647 | 3.289 | 2.433 |
|  |  | Total effect | -0.294 | -0.429 | -0.147 | 1.035 |
|  |  | Indirect effect (%) | 9.541 | -1544.296 | 1780.404 | 1.747 |
| 29 | Nara | a | - | - | - | - |
|  |  | b | - | - | - | - |
|  |  | c (Direct effect) | - | - | - | - |
|  |  | Indirect effect | - | - | - | - |
|  |  | Total effect | - | - | - | - |
|  |  | Indirect effect (%) | - | - | - | - |
| 30 | Wakayama | a | -0.107 | -0.462 | 0.255 | 1.002 |
|  |  | b | 0.282 | 0.208 | 0.364 | 1.003 |
|  |  | c (Direct effect) | -0.051 | -0.207 | 0.094 | 1.003 |
|  |  | Indirect effect | -0.030 | -0.130 | 0.073 | 1.002 |
|  |  | Total effect | -0.081 | -0.229 | 0.076 | 1.003 |
|  |  | Indirect effect (%) | -414.884 | -406.486 | 462.541 | 1.000 |
| 31 | Tottori | a | - | - | - | - |
|  |  | b | - | - | - | - |
|  |  | c (Direct effect) | - | - | - | - |
|  |  | Indirect effect | - | - | - | - |
|  |  | Total effect | - | - | - | - |
|  |  | Indirect effect (%) | - | - | - | - |
| 32 | Shimane | a | -0.024 | -0.433 | 0.388 | 1.000 |
|  |  | b | 0.238 | 0.099 | 0.397 | 1.002 |
|  |  | c (Direct effect) | -0.347 | -0.571 | -0.117 | 1.000 |
|  |  | Indirect effect | -0.006 | -0.117 | 0.097 | 1.000 |
|  |  | Total effect | -0.354 | -0.578 | -0.125 | 1.000 |
|  |  | Indirect effect (%) | 0.371 | -42.244 | 37.281 | 1.000 |
| 33 | Okayama | a | -0.197 | -0.474 | 0.074 | 1.000 |
|  |  | b | 0.704 | 0.528 | 0.915 | 1.003 |
|  |  | c (Direct effect) | 0.042 | -0.134 | 0.229 | 1.001 |
|  |  | Indirect effect | -0.138 | -0.340 | 0.052 | 1.000 |
|  |  | Total effect | -0.096 | -0.313 | 0.123 | 1.000 |
|  |  | Indirect effect (%) | -237.851 | -850.630 | 1013.746 | 1.000 |
| 34 | Hiroshima | a | -0.823 | -1.030 | -0.623 | 1.000 |
|  |  | b | 0.676 | 0.445 | 0.948 | 1.001 |
|  |  | c (Direct effect) | -0.193 | -0.446 | 0.074 | 1.001 |
|  |  | Indirect effect | -0.553 | -0.797 | -0.348 | 1.001 |
|  |  | Total effect | -0.747 | -0.924 | -0.568 | 1.000 |
|  |  | Indirect effect (%) | 74.903 | 46.330 | 111.374 | 1.002 |
| 35 | Yamaguchi | a | -0.981 | -1.298 | -0.655 | 1.006 |
|  |  | b | 0.195 | 0.126 | 0.280 | 1.035 |
|  |  | c (Direct effect) | -0.163 | -0.280 | -0.040 | 1.007 |
|  |  | Indirect effect | -0.190 | -0.282 | -0.120 | 1.013 |
|  |  | Total effect | -0.353 | -0.476 | -0.232 | 1.001 |
|  |  | Indirect effect (%) | 54.978 | 34.272 | 85.159 | 1.012 |
| 36 | Tokushima | a | - | - | - | - |
|  |  | b | - | - | - | - |
|  |  | c (Direct effect) | - | - | - | - |
|  |  | Indirect effect | - | - | - | - |
|  |  | Total effect | - | - | - | - |
|  |  | Indirect effect (%) | - | - | - | - |
| 37 | Kagawa | a | -0.355 | -0.714 | -0.018 | 1.002 |
|  |  | b | 0.256 | 0.201 | 0.320 | 1.008 |
|  |  | c (Direct effect) | -0.094 | -0.215 | 0.024 | 1.000 |
|  |  | Indirect effect | -0.091 | -0.184 | -0.005 | 1.002 |
|  |  | Total effect | -0.185 | -0.321 | -0.049 | 1.001 |
|  |  | Indirect effect (%) | 52.857 | 2.921 | 127.548 | 1.000 |
| 38 | Ehime | a | - | - | - | - |
|  |  | b | - | - | - | - |
|  |  | c (Direct effect) | - | - | - | - |
|  |  | Indirect effect | - | - | - | - |
|  |  | Total effect | - | - | - | - |
|  |  | Indirect effect (%) | - | - | - | - |
| 39 | Kochi | a | -0.370 | -0.753 | 0.017 | 1.000 |
|  |  | b | 0.288 | 0.224 | 0.360 | 1.004 |
|  |  | c (Direct effect) | 0.089 | -0.033 | 0.213 | 1.000 |
|  |  | Indirect effect | -0.107 | -0.229 | 0.005 | 1.000 |
|  |  | Total effect | -0.018 | -0.161 | 0.126 | 1.000 |
|  |  | Indirect effect (%) | 355.324 | -2181.637 | 2236.510 | 1.000 |
| 40 | Fukuoka | a | -0.158 | -0.392 | 0.076 | 1.002 |
|  |  | b | 0.415 | 0.282 | 0.616 | 1.006 |
|  |  | c (Direct effect) | -0.217 | -0.377 | -0.042 | 1.001 |
|  |  | Indirect effect | -0.068 | -0.193 | 0.029 | 1.003 |
|  |  | Total effect | -0.285 | -0.450 | -0.116 | 1.000 |
|  |  | Indirect effect (%) | 24.080 | -14.708 | 75.487 | 1.001 |
| 41 | Saga | a | -0.240 | -0.695 | 0.221 | 1.001 |
|  |  | b | 0.273 | 0.200 | 0.357 | 1.003 |
|  |  | c (Direct effect) | -0.015 | -0.174 | 0.152 | 1.001 |
|  |  | Indirect effect | -0.066 | -0.203 | 0.058 | 1.001 |
|  |  | Total effect | -0.081 | -0.248 | 0.090 | 1.000 |
|  |  | Indirect effect (%) | -5.717 | -625.701 | 696.042 | 1.000 |
| 42 | Nagasaki | a | -0.911 | -1.311 | -0.365 | 1.006 |
|  |  | b | 0.845 | 0.323 | 1.470 | 1.011 |
|  |  | c (Direct effect) | 0.440 | -0.234 | 1.283 | 1.010 |
|  |  | Indirect effect | -0.812 | -1.676 | -0.132 | 1.010 |
|  |  | Total effect | -0.372 | -0.555 | -0.188 | 1.000 |
|  |  | Indirect effect (%) | 228.674 | 37.728 | 519.109 | 1.007 |
| 43 | Kumamoto | a | -0.374 | -0.785 | 0.041 | 1.001 |
|  |  | b | 0.449 | 0.377 | 0.543 | 1.001 |
|  |  | c (Direct effect) | -0.362 | -0.574 | -0.142 | 1.000 |
|  |  | Indirect effect | -0.169 | -0.369 | 0.018 | 1.001 |
|  |  | Total effect | -0.531 | -0.796 | -0.267 | 1.001 |
|  |  | Indirect effect (%) | 30.878 | -4.962 | 63.707 | 1.000 |
| 44 | Oita | a | -0.160 | -0.527 | 0.222 | 1.004 |
|  |  | b | 0.598 | 0.418 | 0.806 | 1.017 |
|  |  | c (Direct effect) | -0.148 | -0.370 | 0.063 | 1.003 |
|  |  | Indirect effect | -0.092 | -0.311 | 0.138 | 1.002 |
|  |  | Total effect | -0.240 | -0.468 | -0.016 | 1.001 |
|  |  | Indirect effect (%) | 14.803 | -146.403 | 171.388 | 1.000 |
| 45 | Miyazaki | a | -0.429 | -0.830 | -0.042 | 1.003 |
|  |  | b | 0.361 | 0.311 | 0.424 | 1.018 |
|  |  | c (Direct effect) | -0.214 | -0.378 | -0.054 | 1.004 |
|  |  | Indirect effect | -0.156 | -0.310 | -0.015 | 1.005 |
|  |  | Total effect | -0.369 | -0.579 | -0.166 | 1.004 |
|  |  | Indirect effect (%) | 41.923 | 5.758 | 78.334 | 1.003 |
| 46 | Kagoshima | a | -0.342 | -0.668 | 0.025 | 1.032 |
|  |  | b | 0.315 | 0.254 | 0.377 | 1.001 |
|  |  | c (Direct effect) | -0.217 | -0.390 | -0.044 | 1.001 |
|  |  | Indirect effect | -0.108 | -0.215 | 0.008 | 1.029 |
|  |  | Total effect | -0.325 | -0.521 | -0.118 | 1.009 |
|  |  | Indirect effect (%) | 34.039 | -3.206 | 73.734 | 1.001 |
| 47 | Okinawa | a | -0.959 | -1.167 | -0.742 | 1.000 |
|  |  | b | 0.767 | 0.560 | 1.004 | 1.001 |
|  |  | c (Direct effect) | -0.068 | -0.336 | 0.241 | 1.001 |
|  |  | Indirect effect | -0.742 | -1.087 | -0.449 | 1.001 |
|  |  | Total effect | -0.809 | -0.969 | -0.652 | 1.000 |
|  |  | Indirect effect (%) | 91.659 | 58.487 | 130.244 | 1.001 |

Prefectures where NPIs were not implemented are represented by "-".

**Table S16. Effects of NPIs on mitigating the COVID-19 infection mediated by Osaka residents’ mobility during Phase 1**

| **Prefecture Number** | **Prefecture Name** | **Parameters** | **Mean** | **95% Credible Interval** | | **Rhat** |
| --- | --- | --- | --- | --- | --- | --- |
|  |  |  |  | **Low** | **High** |  |
| 1 | Hokkaido | a | 0.037 | -0.127 | 0.199 | 1.000 |
|  |  | b | 0.230 | 0.147 | 0.317 | 1.000 |
|  |  | c (Direct effect) | -0.307 | -0.430 | -0.184 | 1.000 |
|  |  | Indirect effect | 0.009 | -0.030 | 0.048 | 1.000 |
|  |  | Total effect | -0.298 | -0.427 | -0.171 | 1.000 |
|  |  | Indirect effect (%) | -3.511 | -20.744 | 9.545 | 1.000 |
| 2 | Aomori | a | 0.309 | 0.095 | 0.525 | 1.008 |
|  |  | b | 0.102 | 0.001 | 0.201 | 1.004 |
|  |  | c (Direct effect) | -0.001 | -0.265 | 0.266 | 1.003 |
|  |  | Indirect effect | 0.031 | 0.000 | 0.077 | 1.009 |
|  |  | Total effect | 0.031 | -0.240 | 0.306 | 1.003 |
|  |  | Indirect effect (%) | 13.850 | -316.428 | 353.301 | 1.000 |
| 3 | Iwate | a | 0.216 | 0.068 | 0.359 | 1.000 |
|  |  | b | 0.189 | 0.067 | 0.310 | 1.000 |
|  |  | c (Direct effect) | -0.085 | -0.264 | 0.093 | 1.000 |
|  |  | Indirect effect | 0.041 | 0.008 | 0.087 | 1.000 |
|  |  | Total effect | -0.044 | -0.229 | 0.141 | 1.000 |
|  |  | Indirect effect (%) | -10.359 | -694.666 | 643.902 | 1.000 |
| 4 | Miyagi | a | 0.054 | -0.069 | 0.175 | 1.000 |
|  |  | b | 0.078 | -0.084 | 0.242 | 1.000 |
|  |  | c (Direct effect) | -0.727 | -0.920 | -0.534 | 1.000 |
|  |  | Indirect effect | 0.004 | -0.009 | 0.025 | 1.000 |
|  |  | Total effect | -0.723 | -0.916 | -0.530 | 1.000 |
|  |  | Indirect effect (%) | -0.605 | -3.692 | 1.285 | 1.000 |
| 5 | Akita | a | 0.349 | 0.147 | 0.549 | 1.000 |
|  |  | b | 0.033 | -0.116 | 0.184 | 1.000 |
|  |  | c (Direct effect) | -0.359 | -0.628 | -0.085 | 1.000 |
|  |  | Indirect effect | 0.012 | -0.041 | 0.070 | 1.000 |
|  |  | Total effect | -0.347 | -0.622 | -0.067 | 1.000 |
|  |  | Indirect effect (%) | 13.935 | -40.923 | 14.415 | 1.000 |
| 6 | Yamagata | a | 0.187 | 0.051 | 0.321 | 1.000 |
|  |  | b | 0.535 | 0.334 | 0.738 | 1.001 |
|  |  | c (Direct effect) | -0.938 | -1.171 | -0.706 | 1.001 |
|  |  | Indirect effect | 0.101 | 0.025 | 0.194 | 1.000 |
|  |  | Total effect | -0.838 | -1.069 | -0.605 | 1.000 |
|  |  | Indirect effect (%) | -12.387 | -26.289 | -2.756 | 1.000 |
| 7 | Fukushima | a | -0.024 | -0.160 | 0.111 | 1.001 |
|  |  | b | 0.302 | 0.161 | 0.444 | 1.001 |
|  |  | c (Direct effect) | -0.636 | -0.837 | -0.433 | 1.000 |
|  |  | Indirect effect | -0.007 | -0.052 | 0.035 | 1.001 |
|  |  | Total effect | -0.643 | -0.847 | -0.437 | 1.000 |
|  |  | Indirect effect (%) | 1.090 | -5.935 | 8.129 | 1.001 |
| 8 | Ibaraki | a | 0.043 | -0.044 | 0.128 | 1.000 |
|  |  | b | 0.472 | 0.125 | 0.853 | 1.001 |
|  |  | c (Direct effect) | -0.728 | -0.905 | -0.541 | 1.001 |
|  |  | Indirect effect | 0.021 | -0.020 | 0.076 | 1.000 |
|  |  | Total effect | -0.707 | -0.888 | -0.515 | 1.001 |
|  |  | Indirect effect (%) | -3.139 | -12.244 | 2.706 | 1.000 |
| 9 | Tochigi | a | -0.095 | -0.177 | -0.015 | 1.000 |
|  |  | b | -0.263 | -0.595 | 0.062 | 1.001 |
|  |  | c (Direct effect) | -0.407 | -0.562 | -0.254 | 1.000 |
|  |  | Indirect effect | 0.025 | -0.006 | 0.075 | 1.001 |
|  |  | Total effect | -0.382 | -0.535 | -0.231 | 1.000 |
|  |  | Indirect effect (%) | -7.002 | -22.553 | 1.502 | 1.000 |
| 10 | Gunma | a | -0.017 | -0.142 | 0.108 | 1.000 |
|  |  | b | -0.153 | -0.430 | 0.134 | 1.002 |
|  |  | c (Direct effect) | -0.432 | -0.648 | -0.223 | 1.001 |
|  |  | Indirect effect | 0.003 | -0.024 | 0.034 | 1.000 |
|  |  | Total effect | -0.429 | -0.644 | -0.220 | 1.001 |
|  |  | Indirect effect (%) | -0.760 | -9.268 | 5.670 | 1.000 |
| 11 | Saitama | a | -0.175 | -0.285 | -0.068 | 1.001 |
|  |  | b | 0.254 | 0.005 | 0.545 | 1.002 |
|  |  | c (Direct effect) | -0.424 | -0.535 | -0.309 | 1.000 |
|  |  | Indirect effect | -0.041 | -0.089 | -0.001 | 1.001 |
|  |  | Total effect | -0.465 | -0.583 | -0.345 | 1.001 |
|  |  | Indirect effect (%) | 8.870 | 0.179 | 18.720 | 1.001 |
| 12 | Chiba | a | -0.263 | -0.348 | -0.180 | 1.001 |
|  |  | b | -0.194 | -0.426 | 0.035 | 1.004 |
|  |  | c (Direct effect) | -0.566 | -0.678 | -0.451 | 1.000 |
|  |  | Indirect effect | 0.051 | -0.009 | 0.120 | 1.004 |
|  |  | Total effect | -0.515 | -0.624 | -0.400 | 1.001 |
|  |  | Indirect effect (%) | -10.278 | -25.823 | 1.688 | 1.004 |
| 13 | Tokyo | a | -0.153 | -0.208 | -0.102 | 1.027 |
|  |  | b | 0.226 | 0.117 | 0.347 | 1.098 |
|  |  | c (Direct effect) | -0.467 | -0.557 | -0.394 | 1.329 |
|  |  | Indirect effect | -0.035 | -0.062 | -0.016 | 1.078 |
|  |  | Total effect | -0.501 | -0.594 | -0.420 | 1.388 |
|  |  | Indirect effect (%) | 6.885 | 3.152 | 11.682 | 1.018 |
| 14 | Kanagawa | a | -0.235 | -0.318 | -0.151 | 1.000 |
|  |  | b | -0.015 | -0.253 | 0.244 | 1.001 |
|  |  | c (Direct effect) | -0.301 | -0.409 | -0.190 | 1.000 |
|  |  | Indirect effect | 0.004 | -0.054 | 0.063 | 1.001 |
|  |  | Total effect | -0.297 | -0.414 | -0.180 | 1.000 |
|  |  | Indirect effect (%) | -2.325 | -26.520 | 16.496 | 1.001 |
| 15 | Niigata | a | 0.150 | -0.006 | 0.306 | 1.001 |
|  |  | b | 0.290 | 0.125 | 0.454 | 1.001 |
|  |  | c (Direct effect) | -0.327 | -0.530 | -0.126 | 1.000 |
|  |  | Indirect effect | 0.044 | -0.002 | 0.105 | 1.002 |
|  |  | Total effect | -0.284 | -0.492 | -0.073 | 1.000 |
|  |  | Indirect effect (%) | 4.746 | -82.536 | 1.051 | 1.000 |
| 16 | Toyama | a | -0.029 | -0.172 | 0.113 | 1.000 |
|  |  | b | 1.121 | 0.861 | 1.394 | 1.000 |
|  |  | c (Direct effect) | -0.494 | -0.735 | -0.250 | 1.000 |
|  |  | Indirect effect | -0.033 | -0.195 | 0.129 | 1.000 |
|  |  | Total effect | -0.526 | -0.795 | -0.257 | 1.000 |
|  |  | Indirect effect (%) | 4.094 | -36.790 | 33.912 | 1.000 |
| 17 | Ishikawa | a | -0.030 | -0.185 | 0.124 | 1.000 |
|  |  | b | 0.834 | 0.558 | 1.131 | 1.000 |
|  |  | c (Direct effect) | -0.472 | -0.706 | -0.241 | 1.000 |
|  |  | Indirect effect | -0.026 | -0.165 | 0.103 | 1.000 |
|  |  | Total effect | -0.498 | -0.735 | -0.261 | 1.000 |
|  |  | Indirect effect (%) | 4.318 | -28.050 | 32.891 | 1.000 |
| 18 | Fukui | a | -0.117 | -0.249 | 0.017 | 1.000 |
|  |  | b | 0.404 | 0.001 | 0.807 | 1.000 |
|  |  | c (Direct effect) | -0.551 | -0.833 | -0.269 | 1.000 |
|  |  | Indirect effect | -0.047 | -0.138 | 0.012 | 1.000 |
|  |  | Total effect | -0.598 | -0.863 | -0.332 | 1.000 |
|  |  | Indirect effect (%) | 8.391 | -1.990 | 27.939 | 1.000 |
| 19 | Yamanashi | a | -0.023 | -0.216 | 0.174 | 1.000 |
|  |  | b | 0.280 | 0.125 | 0.443 | 1.001 |
|  |  | c (Direct effect) | -0.260 | -0.504 | -0.019 | 1.003 |
|  |  | Indirect effect | -0.007 | -0.065 | 0.051 | 1.000 |
|  |  | Total effect | -0.267 | -0.512 | -0.025 | 1.003 |
|  |  | Indirect effect (%) | 8.388 | -41.588 | 41.378 | 1.000 |
| 20 | Nagano | a | -0.032 | -0.262 | 0.197 | 1.000 |
|  |  | b | 0.436 | 0.303 | 0.582 | 1.000 |
|  |  | c (Direct effect) | -0.454 | -0.657 | -0.252 | 1.000 |
|  |  | Indirect effect | -0.014 | -0.118 | 0.087 | 1.000 |
|  |  | Total effect | -0.469 | -0.687 | -0.249 | 1.000 |
|  |  | Indirect effect (%) | 2.547 | -26.314 | 23.839 | 1.000 |
| 21 | Gifu | a | -0.209 | -0.319 | -0.098 | 1.000 |
|  |  | b | -0.110 | -0.360 | 0.127 | 1.012 |
|  |  | c (Direct effect) | -0.562 | -0.682 | -0.442 | 1.000 |
|  |  | Indirect effect | 0.023 | -0.026 | 0.083 | 1.011 |
|  |  | Total effect | -0.538 | -0.659 | -0.418 | 1.002 |
|  |  | Indirect effect (%) | -4.489 | -16.916 | 4.717 | 1.011 |
| 22 | Shizuoka | a | -0.067 | -0.173 | 0.040 | 1.000 |
|  |  | b | 0.926 | 0.606 | 1.270 | 1.001 |
|  |  | c (Direct effect) | -0.592 | -0.815 | -0.369 | 1.000 |
|  |  | Indirect effect | -0.062 | -0.170 | 0.037 | 1.000 |
|  |  | Total effect | -0.654 | -0.887 | -0.420 | 1.000 |
|  |  | Indirect effect (%) | 9.288 | -6.579 | 25.223 | 1.000 |
| 23 | Aichi | a | -0.051 | -0.109 | 0.007 | 1.000 |
|  |  | b | 1.037 | 0.255 | 1.932 | 1.002 |
|  |  | c (Direct effect) | -0.027 | -0.249 | 0.202 | 1.000 |
|  |  | Indirect effect | -0.051 | -0.135 | 0.008 | 1.001 |
|  |  | Total effect | -0.078 | -0.300 | 0.147 | 1.000 |
|  |  | Indirect effect (%) | 187.147 | -491.724 | 588.184 | 1.000 |
| 24 | Mie | a | -0.053 | -0.209 | 0.106 | 1.000 |
|  |  | b | 0.414 | 0.157 | 0.688 | 1.002 |
|  |  | c (Direct effect) | -0.538 | -0.752 | -0.320 | 1.000 |
|  |  | Indirect effect | -0.022 | -0.101 | 0.045 | 1.000 |
|  |  | Total effect | -0.560 | -0.776 | -0.340 | 1.000 |
|  |  | Indirect effect (%) | 3.858 | -9.481 | 18.618 | 1.000 |
| 25 | Shiga | a | -0.118 | -0.201 | -0.034 | 1.000 |
|  |  | b | 0.490 | 0.022 | 1.016 | 1.001 |
|  |  | c (Direct effect) | -0.466 | -0.694 | -0.239 | 1.000 |
|  |  | Indirect effect | -0.058 | -0.151 | 0.000 | 1.001 |
|  |  | Total effect | -0.524 | -0.733 | -0.318 | 1.000 |
|  |  | Indirect effect (%) | 11.782 | 0.067 | 33.341 | 1.001 |
| 26 | Kyoto | a | -0.068 | -0.093 | -0.044 | 1.002 |
|  |  | b | 1.227 | 0.702 | 1.776 | 1.001 |
|  |  | c (Direct effect) | -0.458 | -0.598 | -0.319 | 1.000 |
|  |  | Indirect effect | -0.083 | -0.139 | -0.041 | 1.001 |
|  |  | Total effect | -0.541 | -0.664 | -0.420 | 1.000 |
|  |  | Indirect effect (%) | 15.668 | 6.934 | 27.970 | 1.001 |
| 27 | Osaka | a | 0.007 | 0.005 | 0.010 | 1.000 |
|  |  | b | -36.176 | -56.369 | -17.959 | 1.002 |
|  |  | c (Direct effect) | -0.001 | -0.188 | 0.205 | 1.000 |
|  |  | Indirect effect | -0.260 | -0.434 | -0.121 | 1.001 |
|  |  | Total effect | -0.261 | -0.390 | -0.131 | 1.000 |
|  |  | Indirect effect (%) | 107.039 | 43.211 | 222.542 | 1.000 |
| 28 | Hyogo | a | -0.112 | -0.138 | -0.084 | 1.001 |
|  |  | b | 1.986 | 0.824 | 3.218 | 1.003 |
|  |  | c (Direct effect) | -0.009 | -0.217 | 0.207 | 1.001 |
|  |  | Indirect effect | -0.224 | -0.394 | -0.083 | 1.003 |
|  |  | Total effect | -0.233 | -0.371 | -0.093 | 1.000 |
|  |  | Indirect effect (%) | 109.296 | 31.267 | 277.939 | 1.000 |
| 29 | Nara | a | -0.091 | -0.135 | -0.044 | 1.000 |
|  |  | b | 2.931 | 1.354 | 4.766 | 1.000 |
|  |  | c (Direct effect) | -0.319 | -0.597 | 0.000 | 1.000 |
|  |  | Indirect effect | -0.268 | -0.522 | -0.089 | 1.000 |
|  |  | Total effect | -0.587 | -0.786 | -0.381 | 1.000 |
|  |  | Indirect effect (%) | 47.285 | 14.671 | 99.989 | 1.000 |
| 30 | Wakayama | a | -0.025 | -0.144 | 0.096 | 1.008 |
|  |  | b | 0.421 | 0.024 | 0.820 | 1.036 |
|  |  | c (Direct effect) | -0.082 | -0.347 | 0.181 | 1.008 |
|  |  | Indirect effect | -0.010 | -0.074 | 0.049 | 1.006 |
|  |  | Total effect | -0.092 | -0.358 | 0.172 | 1.006 |
|  |  | Indirect effect (%) | 7.146 | -208.953 | 219.365 | 1.000 |
| 31 | Tottori | a | 0.208 | 0.011 | 0.410 | 1.001 |
|  |  | b | 0.106 | -0.084 | 0.297 | 1.000 |
|  |  | c (Direct effect) | -0.202 | -0.452 | 0.046 | 1.000 |
|  |  | Indirect effect | 0.022 | -0.018 | 0.080 | 1.000 |
|  |  | Total effect | -0.180 | -0.437 | 0.076 | 1.000 |
|  |  | Indirect effect (%) | -4.884 | -165.464 | 112.914 | 1.000 |
| 32 | Shimane | a | 0.084 | -0.098 | 0.265 | 1.000 |
|  |  | b | 0.423 | 0.135 | 0.720 | 1.000 |
|  |  | c (Direct effect) | -0.434 | -0.764 | -0.119 | 1.001 |
|  |  | Indirect effect | 0.034 | -0.044 | 0.125 | 1.000 |
|  |  | Total effect | -0.399 | -0.734 | -0.075 | 1.001 |
|  |  | Indirect effect (%) | -14.411 | -70.516 | 14.010 | 1.000 |
| 33 | Okayama | a | 0.057 | -0.076 | 0.192 | 1.000 |
|  |  | b | 0.381 | -0.013 | 0.819 | 1.002 |
|  |  | c (Direct effect) | -0.104 | -0.316 | 0.118 | 1.002 |
|  |  | Indirect effect | 0.023 | -0.028 | 0.100 | 1.000 |
|  |  | Total effect | -0.081 | -0.298 | 0.150 | 1.003 |
|  |  | Indirect effect (%) | -63.440 | -336.191 | 331.408 | 1.000 |
| 34 | Hiroshima | a | 0.024 | -0.100 | 0.148 | 1.000 |
|  |  | b | 0.400 | -0.114 | 0.948 | 1.001 |
|  |  | c (Direct effect) | -1.018 | -1.269 | -0.770 | 1.000 |
|  |  | Indirect effect | 0.008 | -0.053 | 0.076 | 1.000 |
|  |  | Total effect | -1.010 | -1.260 | -0.764 | 1.000 |
|  |  | Indirect effect (%) | -0.879 | -8.045 | 5.279 | 1.000 |
| 35 | Yamaguchi | a | 0.034 | -0.104 | 0.172 | 1.000 |
|  |  | b | 0.580 | 0.282 | 0.890 | 1.001 |
|  |  | c (Direct effect) | -0.229 | -0.496 | 0.033 | 1.001 |
|  |  | Indirect effect | 0.019 | -0.064 | 0.105 | 1.000 |
|  |  | Total effect | -0.210 | -0.483 | 0.062 | 1.001 |
|  |  | Indirect effect (%) | -15.706 | -183.832 | 133.707 | 1.000 |
| 36 | Tokushima | a | 0.204 | 0.011 | 0.400 | 1.000 |
|  |  | b | 0.314 | 0.108 | 0.520 | 1.000 |
|  |  | c (Direct effect) | -0.062 | -0.318 | 0.198 | 1.000 |
|  |  | Indirect effect | 0.064 | 0.002 | 0.155 | 1.000 |
|  |  | Total effect | 0.002 | -0.264 | 0.274 | 1.000 |
|  |  | Indirect effect (%) | 9.729 | -737.198 | 703.227 | 1.000 |
| 37 | Kagawa | a | 0.064 | -0.126 | 0.257 | 1.001 |
|  |  | b | 0.164 | -0.071 | 0.426 | 1.001 |
|  |  | c (Direct effect) | -0.612 | -0.864 | -0.363 | 1.002 |
|  |  | Indirect effect | 0.009 | -0.030 | 0.057 | 1.000 |
|  |  | Total effect | -0.603 | -0.856 | -0.351 | 1.002 |
|  |  | Indirect effect (%) | -1.706 | -11.653 | 5.156 | 1.000 |
| 38 | Ehime | a | 0.097 | -0.090 | 0.284 | 1.001 |
|  |  | b | 0.320 | 0.117 | 0.531 | 1.003 |
|  |  | c (Direct effect) | 0.295 | 0.029 | 0.563 | 1.000 |
|  |  | Indirect effect | 0.031 | -0.029 | 0.105 | 1.001 |
|  |  | Total effect | 0.326 | 0.052 | 0.604 | 1.000 |
|  |  | Indirect effect (%) | 10.126 | -15.502 | 45.130 | 1.000 |
| 39 | Kochi | a | 0.119 | -0.102 | 0.345 | 1.000 |
|  |  | b | 0.489 | 0.294 | 0.718 | 1.001 |
|  |  | c (Direct effect) | -0.581 | -0.857 | -0.314 | 1.003 |
|  |  | Indirect effect | 0.059 | -0.049 | 0.184 | 1.001 |
|  |  | Total effect | -0.523 | -0.811 | -0.238 | 1.002 |
|  |  | Indirect effect (%) | -14.297 | -55.077 | 8.526 | 1.000 |
| 40 | Fukuoka | a | -0.182 | -0.286 | -0.079 | 1.000 |
|  |  | b | -0.088 | -0.547 | 0.364 | 1.000 |
|  |  | c (Direct effect) | -0.924 | -1.140 | -0.714 | 1.000 |
|  |  | Indirect effect | 0.016 | -0.070 | 0.109 | 1.000 |
|  |  | Total effect | -0.908 | -1.102 | -0.716 | 1.000 |
|  |  | Indirect effect (%) | -1.794 | -12.330 | 7.824 | 1.000 |
| 41 | Saga | a | -0.021 | -0.195 | 0.162 | 1.001 |
|  |  | b | 0.428 | 0.202 | 0.666 | 1.005 |
|  |  | c (Direct effect) | 0.052 | -0.205 | 0.304 | 1.001 |
|  |  | Indirect effect | -0.009 | -0.093 | 0.073 | 1.000 |
|  |  | Total effect | 0.044 | -0.218 | 0.307 | 1.001 |
|  |  | Indirect effect (%) | 0.812 | -354.563 | 349.610 | 1.000 |
| 42 | Nagasaki | a | 0.175 | -0.049 | 0.400 | 1.000 |
|  |  | b | 0.176 | 0.053 | 0.313 | 1.002 |
|  |  | c (Direct effect) | -0.290 | -0.521 | -0.065 | 1.001 |
|  |  | Indirect effect | 0.031 | -0.008 | 0.088 | 1.000 |
|  |  | Total effect | -0.259 | -0.494 | -0.026 | 1.001 |
|  |  | Indirect effect (%) | -9.722 | -87.833 | 6.061 | 1.000 |
| 43 | Kumamoto | a | 0.123 | -0.050 | 0.297 | 1.000 |
|  |  | b | 0.320 | 0.094 | 0.565 | 1.001 |
|  |  | c (Direct effect) | -0.449 | -0.699 | -0.200 | 1.001 |
|  |  | Indirect effect | 0.040 | -0.015 | 0.119 | 1.001 |
|  |  | Total effect | -0.409 | -0.665 | -0.157 | 1.001 |
|  |  | Indirect effect (%) | -13.173 | -46.314 | 3.620 | 1.000 |
| 44 | Oita | a | 0.017 | -0.170 | 0.204 | 1.000 |
|  |  | b | 0.382 | 0.204 | 0.592 | 1.001 |
|  |  | c (Direct effect) | -0.331 | -0.545 | -0.117 | 1.001 |
|  |  | Indirect effect | 0.005 | -0.072 | 0.079 | 1.000 |
|  |  | Total effect | -0.326 | -0.543 | -0.111 | 1.001 |
|  |  | Indirect effect (%) | -4.440 | -41.548 | 24.382 | 1.000 |
| 45 | Miyazaki | a | 0.151 | -0.054 | 0.354 | 1.000 |
|  |  | b | 0.364 | 0.175 | 0.572 | 1.001 |
|  |  | c (Direct effect) | -0.199 | -0.515 | 0.110 | 1.000 |
|  |  | Indirect effect | 0.055 | -0.018 | 0.148 | 1.000 |
|  |  | Total effect | -0.144 | -0.468 | 0.176 | 1.000 |
|  |  | Indirect effect (%) | 84.599 | -448.143 | 435.106 | 1.000 |
| 46 | Kagoshima | a | 0.068 | -0.139 | 0.265 | 1.007 |
|  |  | b | 0.229 | 0.055 | 0.414 | 1.017 |
|  |  | c (Direct effect) | -0.223 | -0.575 | 0.121 | 1.075 |
|  |  | Indirect effect | 0.015 | -0.034 | 0.075 | 1.003 |
|  |  | Total effect | -0.208 | -0.559 | 0.142 | 1.071 |
|  |  | Indirect effect (%) | 298.107 | -125.344 | 122.980 | 1.000 |
| 47 | Okinawa | a | -0.036 | -0.211 | 0.142 | 1.000 |
|  |  | b | 0.327 | 0.218 | 0.437 | 1.001 |
|  |  | c (Direct effect) | -0.777 | -0.990 | -0.566 | 1.000 |
|  |  | Indirect effect | -0.012 | -0.071 | 0.048 | 1.000 |
|  |  | Total effect | -0.789 | -1.006 | -0.571 | 1.000 |
|  |  | Indirect effect (%) | 1.369 | -6.731 | 8.903 | 1.000 |

**Table S17. Effects of NPIs on mitigating the COVID-19 infection mediated by Osaka residents’ mobility during Phase 2**

| **Prefecture Number** | **Prefecture Name** | **Parameters** | **Mean** | **95% Credible Interval** | | **Rhat** |
| --- | --- | --- | --- | --- | --- | --- |
|  |  |  |  | **Low** | **High** |  |
| 1 | Hokkaido | a | -0.223 | -0.329 | -0.116 | 1.001 |
|  |  | b | 0.512 | 0.372 | 0.660 | 1.001 |
|  |  | c (Direct effect) | -0.037 | -0.132 | 0.060 | 1.000 |
|  |  | Indirect effect | -0.114 | -0.183 | -0.055 | 1.000 |
|  |  | Total effect | -0.152 | -0.242 | -0.059 | 1.000 |
|  |  | Indirect effect (%) | -129.432 | 36.478 | 181.478 | 1.000 |
| 2 | Aomori | a | - | - | - | - |
|  |  | b | - | - | - | - |
|  |  | c (Direct effect) | - | - | - | - |
|  |  | Indirect effect | - | - | - | - |
|  |  | Total effect | - | - | - | - |
|  |  | Indirect effect (%) | - | - | - | - |
| 3 | Iwate | a | - | - | - | - |
|  |  | b | - | - | - | - |
|  |  | c (Direct effect) | - | - | - | - |
|  |  | Indirect effect | - | - | - | - |
|  |  | Total effect | - | - | - | - |
|  |  | Indirect effect (%) | - | - | - | - |
| 4 | Miyagi | a | 0.161 | 0.071 | 0.247 | 1.000 |
|  |  | b | 0.167 | -0.366 | 0.770 | 1.001 |
|  |  | c (Direct effect) | -0.209 | -0.382 | -0.040 | 1.000 |
|  |  | Indirect effect | 0.030 | -0.056 | 0.154 | 1.001 |
|  |  | Total effect | -0.179 | -0.309 | -0.042 | 1.000 |
|  |  | Indirect effect (%) | -22.388 | -125.433 | 48.202 | 1.000 |
| 5 | Akita | a | - | - | - | - |
|  |  | b | - | - | - | - |
|  |  | c (Direct effect) | - | - | - | - |
|  |  | Indirect effect | - | - | - | - |
|  |  | Total effect | - | - | - | - |
|  |  | Indirect effect (%) | - | - | - | - |
| 6 | Yamagata | a | - | - | - | - |
|  |  | b | - | - | - | - |
|  |  | c (Direct effect) | - | - | - | - |
|  |  | Indirect effect | - | - | - | - |
|  |  | Total effect | - | - | - | - |
|  |  | Indirect effect (%) | - | - | - | - |
| 7 | Fukushima | a | 0.459 | 0.306 | 0.615 | 1.000 |
|  |  | b | 0.105 | -0.051 | 0.258 | 1.002 |
|  |  | c (Direct effect) | -0.399 | -0.569 | -0.230 | 1.000 |
|  |  | Indirect effect | 0.048 | -0.023 | 0.124 | 1.002 |
|  |  | Total effect | -0.351 | -0.508 | -0.196 | 1.000 |
|  |  | Indirect effect (%) | -14.751 | -43.744 | 6.942 | 1.002 |
| 8 | Ibaraki | a | 0.308 | 0.198 | 0.417 | 1.001 |
|  |  | b | -0.108 | -0.344 | 0.112 | 1.008 |
|  |  | c (Direct effect) | -0.177 | -0.317 | -0.031 | 1.002 |
|  |  | Indirect effect | -0.033 | -0.112 | 0.034 | 1.008 |
|  |  | Total effect | -0.211 | -0.346 | -0.075 | 1.000 |
|  |  | Indirect effect (%) | 14.731 | -21.451 | 68.276 | 1.000 |
| 9 | Tochigi | a | 0.413 | 0.251 | 0.582 | 1.000 |
|  |  | b | 0.003 | -0.240 | 0.257 | 1.000 |
|  |  | c (Direct effect) | -0.119 | -0.292 | 0.069 | 1.001 |
|  |  | Indirect effect | 0.001 | -0.101 | 0.106 | 1.000 |
|  |  | Total effect | -0.119 | -0.270 | 0.043 | 1.001 |
|  |  | Indirect effect (%) | -35.316 | -287.883 | 265.654 | 1.000 |
| 10 | Gunma | a | 0.238 | 0.127 | 0.349 | 1.000 |
|  |  | b | 0.355 | 0.115 | 0.634 | 1.002 |
|  |  | c (Direct effect) | -0.386 | -0.520 | -0.252 | 1.000 |
|  |  | Indirect effect | 0.086 | 0.022 | 0.177 | 1.002 |
|  |  | Total effect | -0.301 | -0.420 | -0.178 | 1.000 |
|  |  | Indirect effect (%) | -30.387 | -74.534 | -6.795 | 1.001 |
| 11 | Saitama | a | -0.050 | -0.150 | 0.051 | 1.001 |
|  |  | b | -0.006 | -0.135 | 0.120 | 1.001 |
|  |  | c (Direct effect) | -0.023 | -0.097 | 0.051 | 1.000 |
|  |  | Indirect effect | 0.000 | -0.009 | 0.012 | 1.000 |
|  |  | Total effect | -0.022 | -0.096 | 0.051 | 1.000 |
|  |  | Indirect effect (%) | 11.688 | -110.426 | 108.771 | 1.000 |
| 12 | Chiba | a | -0.038 | -0.150 | 0.071 | 1.001 |
|  |  | b | 0.079 | -0.057 | 0.240 | 1.006 |
|  |  | c (Direct effect) | -0.036 | -0.115 | 0.044 | 1.000 |
|  |  | Indirect effect | -0.004 | -0.024 | 0.007 | 1.003 |
|  |  | Total effect | -0.040 | -0.118 | 0.040 | 1.001 |
|  |  | Indirect effect (%) | -9.527 | -107.628 | 138.474 | 1.000 |
| 13 | Tokyo | a | -0.019 | -0.069 | 0.029 | 1.029 |
|  |  | b | 0.034 | -0.074 | 0.147 | 1.052 |
|  |  | c (Direct effect) | 0.001 | -0.049 | 0.051 | 1.031 |
|  |  | Indirect effect | -0.001 | -0.005 | 0.003 | 1.034 |
|  |  | Total effect | 0.000 | -0.050 | 0.050 | 1.035 |
|  |  | Indirect effect (%) | -26.621 | -87.696 | 84.866 | 1.000 |
| 14 | Kanagawa | a | -0.082 | -0.176 | 0.009 | 1.002 |
|  |  | b | -0.034 | -0.186 | 0.100 | 1.006 |
|  |  | c (Direct effect) | -0.108 | -0.184 | -0.037 | 1.020 |
|  |  | Indirect effect | 0.003 | -0.010 | 0.021 | 1.006 |
|  |  | Total effect | -0.105 | -0.180 | -0.034 | 1.026 |
|  |  | Indirect effect (%) | -2.583 | -27.848 | 11.382 | 1.000 |
| 15 | Niigata | a | - | - | - | - |
|  |  | b | - | - | - | - |
|  |  | c (Direct effect) | - | - | - | - |
|  |  | Indirect effect | - | - | - | - |
|  |  | Total effect | - | - | - | - |
|  |  | Indirect effect (%) | - | - | - | - |
| 16 | Toyama | a | -0.315 | -0.466 | -0.166 | 1.000 |
|  |  | b | 0.294 | 0.064 | 0.532 | 1.003 |
|  |  | c (Direct effect) | -0.519 | -0.735 | -0.304 | 1.000 |
|  |  | Indirect effect | -0.091 | -0.183 | -0.019 | 1.002 |
|  |  | Total effect | -0.611 | -0.844 | -0.377 | 1.001 |
|  |  | Indirect effect (%) | 15.022 | 3.467 | 29.414 | 1.002 |
| 17 | Ishikawa | a | -0.070 | -0.177 | 0.036 | 1.000 |
|  |  | b | 0.394 | 0.132 | 0.671 | 1.001 |
|  |  | c (Direct effect) | -0.297 | -0.433 | -0.162 | 1.000 |
|  |  | Indirect effect | -0.027 | -0.079 | 0.014 | 1.000 |
|  |  | Total effect | -0.324 | -0.467 | -0.184 | 1.000 |
|  |  | Indirect effect (%) | 8.297 | -5.213 | 24.930 | 1.000 |
| 18 | Fukui | a | - | - | - | - |
|  |  | b | - | - | - | - |
|  |  | c (Direct effect) | - | - | - | - |
|  |  | Indirect effect | - | - | - | - |
|  |  | Total effect | - | - | - | - |
|  |  | Indirect effect (%) | - | - | - | - |
| 19 | Yamanashi | a | 0.001 | -0.255 | 0.252 | 1.000 |
|  |  | b | 0.461 | 0.262 | 0.678 | 1.000 |
|  |  | c (Direct effect) | -0.323 | -0.553 | -0.095 | 1.000 |
|  |  | Indirect effect | 0.002 | -0.117 | 0.127 | 1.000 |
|  |  | Total effect | -0.321 | -0.551 | -0.093 | 1.000 |
|  |  | Indirect effect (%) | -2.285 | -67.275 | 39.721 | 1.000 |
| 20 | Nagano | a | - | - | - | - |
|  |  | b | - | - | - | - |
|  |  | c (Direct effect) | - | - | - | - |
|  |  | Indirect effect | - | - | - | - |
|  |  | Total effect | - | - | - | - |
|  |  | Indirect effect (%) | - | - | - | - |
| 21 | Gifu | a | -0.118 | -0.226 | -0.008 | 1.000 |
|  |  | b | 0.514 | 0.324 | 0.720 | 1.001 |
|  |  | c (Direct effect) | -0.186 | -0.299 | -0.067 | 1.000 |
|  |  | Indirect effect | -0.060 | -0.125 | -0.004 | 1.001 |
|  |  | Total effect | -0.246 | -0.359 | -0.128 | 1.000 |
|  |  | Indirect effect (%) | 25.289 | 1.850 | 57.355 | 1.001 |
| 22 | Shizuoka | a | 0.309 | 0.201 | 0.420 | 1.000 |
|  |  | b | 1.042 | 0.650 | 1.440 | 1.002 |
|  |  | c (Direct effect) | -0.332 | -0.509 | -0.152 | 1.001 |
|  |  | Indirect effect | 0.322 | 0.174 | 0.493 | 1.001 |
|  |  | Total effect | -0.010 | -0.166 | 0.149 | 1.000 |
|  |  | Indirect effect (%) | 438.376 | -6215.903 | 6094.561 | 1.000 |
| 23 | Aichi | a | 0.048 | 0.009 | 0.090 | 1.000 |
|  |  | b | 1.747 | 1.189 | 2.349 | 1.004 |
|  |  | c (Direct effect) | -0.095 | -0.161 | -0.027 | 1.000 |
|  |  | Indirect effect | 0.083 | 0.016 | 0.152 | 1.001 |
|  |  | Total effect | -0.012 | -0.074 | 0.048 | 1.000 |
|  |  | Indirect effect (%) | -165.855 | -3993.139 | 4279.464 | 1.000 |
| 24 | Mie | a | -0.211 | -0.311 | -0.112 | 1.000 |
|  |  | b | 0.609 | 0.384 | 0.863 | 1.000 |
|  |  | c (Direct effect) | -0.230 | -0.334 | -0.119 | 1.000 |
|  |  | Indirect effect | -0.128 | -0.215 | -0.059 | 1.000 |
|  |  | Total effect | -0.358 | -0.452 | -0.263 | 1.000 |
|  |  | Indirect effect (%) | 36.123 | 17.117 | 61.198 | 1.000 |
| 25 | Shiga | a | 0.015 | -0.040 | 0.073 | 1.000 |
|  |  | b | 2.896 | 2.034 | 3.932 | 1.003 |
|  |  | c (Direct effect) | -0.055 | -0.198 | 0.095 | 1.000 |
|  |  | Indirect effect | 0.039 | -0.124 | 0.201 | 1.000 |
|  |  | Total effect | -0.015 | -0.193 | 0.167 | 1.000 |
|  |  | Indirect effect (%) | 236.018 | -1055.074 | 1207.438 | 1.000 |
| 26 | Kyoto | a | -0.020 | -0.036 | -0.005 | 1.007 |
|  |  | b | -0.083 | -0.873 | 0.663 | 1.003 |
|  |  | c (Direct effect) | -0.293 | -0.391 | -0.193 | 1.002 |
|  |  | Indirect effect | 0.002 | -0.014 | 0.020 | 1.006 |
|  |  | Total effect | -0.291 | -0.386 | -0.196 | 1.004 |
|  |  | Indirect effect (%) | -0.540 | -7.090 | 5.457 | 1.005 |
| 27 | Osaka | a | 0.000 | -0.003 | 0.002 | 1.000 |
|  |  | b | -27.630 | -36.565 | -19.417 | 1.001 |
|  |  | c (Direct effect) | -0.111 | -0.181 | -0.038 | 1.000 |
|  |  | Indirect effect | 0.005 | -0.063 | 0.070 | 1.000 |
|  |  | Total effect | -0.106 | -0.174 | -0.038 | 1.000 |
|  |  | Indirect effect (%) | -11.824 | -122.319 | 53.814 | 1.000 |
| 28 | Hyogo | a | -0.018 | -0.030 | -0.006 | 1.033 |
|  |  | b | 1.204 | 0.562 | 1.836 | 1.025 |
|  |  | c (Direct effect) | -0.122 | -0.184 | -0.060 | 1.002 |
|  |  | Indirect effect | -0.021 | -0.043 | -0.006 | 1.010 |
|  |  | Total effect | -0.144 | -0.206 | -0.081 | 1.005 |
|  |  | Indirect effect (%) | 15.447 | 4.191 | 33.891 | 1.005 |
| 29 | Nara | a | - | - | - | - |
|  |  | b | - | - | - | - |
|  |  | c (Direct effect) | - | - | - | - |
|  |  | Indirect effect | - | - | - | - |
|  |  | Total effect | - | - | - | - |
|  |  | Indirect effect (%) | - | - | - | - |
| 30 | Wakayama | a | - | - | - | - |
|  |  | b | - | - | - | - |
|  |  | c (Direct effect) | - | - | - | - |
|  |  | Indirect effect | - | - | - | - |
|  |  | Total effect | - | - | - | - |
|  |  | Indirect effect (%) | - | - | - | - |
| 31 | Tottori | a | - | - | - | - |
|  |  | b | - | - | - | - |
|  |  | c (Direct effect) | - | - | - | - |
|  |  | Indirect effect | - | - | - | - |
|  |  | Total effect | - | - | - | - |
|  |  | Indirect effect (%) | - | - | - | - |
| 32 | Shimane | a | - | - | - | - |
|  |  | b | - | - | - | - |
|  |  | c (Direct effect) | - | - | - | - |
|  |  | Indirect effect | - | - | - | - |
|  |  | Total effect | - | - | - | - |
|  |  | Indirect effect (%) | - | - | - | - |
| 33 | Okayama | a | -0.208 | -0.294 | -0.121 | 1.000 |
|  |  | b | 0.522 | 0.072 | 1.076 | 1.005 |
|  |  | c (Direct effect) | -0.255 | -0.447 | -0.066 | 1.003 |
|  |  | Indirect effect | -0.106 | -0.229 | -0.015 | 1.004 |
|  |  | Total effect | -0.361 | -0.540 | -0.210 | 1.001 |
|  |  | Indirect effect (%) | 30.996 | 4.219 | 73.503 | 1.004 |
| 34 | Hiroshima | a | -0.191 | -0.276 | -0.107 | 1.000 |
|  |  | b | 0.810 | 0.617 | 1.015 | 1.001 |
|  |  | c (Direct effect) | -0.168 | -0.275 | -0.059 | 1.000 |
|  |  | Indirect effect | -0.155 | -0.237 | -0.082 | 1.000 |
|  |  | Total effect | -0.323 | -0.436 | -0.207 | 1.000 |
|  |  | Indirect effect (%) | 48.655 | 27.419 | 76.355 | 1.000 |
| 35 | Yamaguchi | a | - | - | - | - |
|  |  | b | - | - | - | - |
|  |  | c (Direct effect) | - | - | - | - |
|  |  | Indirect effect | - | - | - | - |
|  |  | Total effect | - | - | - | - |
|  |  | Indirect effect (%) | - | - | - | - |
| 36 | Tokushima | a | - | - | - | - |
|  |  | b | - | - | - | - |
|  |  | c (Direct effect) | - | - | - | - |
|  |  | Indirect effect | - | - | - | - |
|  |  | Total effect | - | - | - | - |
|  |  | Indirect effect (%) | - | - | - | - |
| 37 | Kagawa | a | -0.098 | -0.279 | 0.083 | 1.000 |
|  |  | b | 0.567 | 0.428 | 0.713 | 1.001 |
|  |  | c (Direct effect) | -0.556 | -0.707 | -0.405 | 1.001 |
|  |  | Indirect effect | -0.055 | -0.160 | 0.048 | 1.000 |
|  |  | Total effect | -0.611 | -0.791 | -0.435 | 1.001 |
|  |  | Indirect effect (%) | 8.468 | -9.363 | 23.323 | 1.000 |
| 38 | Ehime | a | -0.153 | -0.273 | -0.032 | 1.001 |
|  |  | b | 0.268 | 0.072 | 0.467 | 1.004 |
|  |  | c (Direct effect) | -0.400 | -0.583 | -0.215 | 1.000 |
|  |  | Indirect effect | -0.041 | -0.092 | -0.004 | 1.001 |
|  |  | Total effect | -0.441 | -0.630 | -0.250 | 1.000 |
|  |  | Indirect effect (%) | 9.470 | 1.048 | 21.975 | 1.000 |
| 39 | Kochi | a | -0.371 | -0.581 | -0.157 | 1.000 |
|  |  | b | 0.381 | 0.230 | 0.533 | 1.000 |
|  |  | c (Direct effect) | -0.341 | -0.608 | -0.069 | 1.000 |
|  |  | Indirect effect | -0.141 | -0.249 | -0.054 | 1.000 |
|  |  | Total effect | -0.481 | -0.761 | -0.202 | 1.000 |
|  |  | Indirect effect (%) | 32.085 | 11.457 | 69.431 | 1.000 |
| 40 | Fukuoka | a | -0.118 | -0.190 | -0.045 | 1.001 |
|  |  | b | 0.603 | 0.325 | 0.900 | 1.001 |
|  |  | c (Direct effect) | -0.226 | -0.318 | -0.131 | 1.000 |
|  |  | Indirect effect | -0.071 | -0.133 | -0.023 | 1.000 |
|  |  | Total effect | -0.297 | -0.381 | -0.213 | 1.000 |
|  |  | Indirect effect (%) | 24.151 | 7.981 | 46.377 | 1.000 |
| 41 | Saga | a | -0.239 | -0.458 | -0.018 | 1.000 |
|  |  | b | 0.302 | 0.138 | 0.476 | 1.000 |
|  |  | c (Direct effect) | -0.430 | -0.670 | -0.187 | 1.000 |
|  |  | Indirect effect | -0.072 | -0.159 | -0.005 | 1.000 |
|  |  | Total effect | -0.502 | -0.746 | -0.258 | 1.000 |
|  |  | Indirect effect (%) | 15.051 | 1.011 | 36.058 | 1.000 |
| 42 | Nagasaki | a | -0.419 | -0.704 | -0.134 | 1.000 |
|  |  | b | 0.210 | 0.088 | 0.338 | 1.000 |
|  |  | c (Direct effect) | -0.152 | -0.406 | 0.103 | 1.000 |
|  |  | Indirect effect | -0.088 | -0.179 | -0.021 | 1.000 |
|  |  | Total effect | -0.240 | -0.493 | 0.015 | 1.000 |
|  |  | Indirect effect (%) | 41.715 | -73.317 | 246.146 | 1.000 |
| 43 | Kumamoto | a | 0.112 | -0.031 | 0.262 | 1.000 |
|  |  | b | 0.559 | 0.401 | 0.732 | 1.001 |
|  |  | c (Direct effect) | -0.361 | -0.485 | -0.235 | 1.000 |
|  |  | Indirect effect | 0.062 | -0.018 | 0.147 | 1.000 |
|  |  | Total effect | -0.299 | -0.440 | -0.155 | 1.000 |
|  |  | Indirect effect (%) | -24.491 | -79.715 | 5.124 | 1.000 |
| 44 | Oita | a | - | - | - | - |
|  |  | b | - | - | - | - |
|  |  | c (Direct effect) | - | - | - | - |
|  |  | Indirect effect | - | - | - | - |
|  |  | Total effect | - | - | - | - |
|  |  | Indirect effect (%) | - | - | - | - |
| 45 | Miyazaki | a | -0.347 | -0.611 | -0.079 | 1.000 |
|  |  | b | 0.391 | 0.188 | 0.603 | 1.001 |
|  |  | c (Direct effect) | -0.291 | -0.576 | -0.001 | 1.000 |
|  |  | Indirect effect | -0.137 | -0.293 | -0.024 | 1.001 |
|  |  | Total effect | -0.428 | -0.702 | -0.150 | 1.000 |
|  |  | Indirect effect (%) | 32.937 | 5.690 | 97.680 | 1.000 |
| 46 | Kagoshima | a | -0.246 | -0.460 | -0.031 | 1.000 |
|  |  | b | 0.339 | 0.220 | 0.463 | 1.002 |
|  |  | c (Direct effect) | -0.432 | -0.616 | -0.248 | 1.000 |
|  |  | Indirect effect | -0.083 | -0.170 | -0.010 | 1.001 |
|  |  | Total effect | -0.515 | -0.713 | -0.322 | 1.000 |
|  |  | Indirect effect (%) | 16.291 | 2.140 | 33.044 | 1.001 |
| 47 | Okinawa | a | 0.039 | -0.135 | 0.212 | 1.002 |
|  |  | b | 0.266 | 0.157 | 0.394 | 1.003 |
|  |  | c (Direct effect) | -0.051 | -0.173 | 0.072 | 1.002 |
|  |  | Indirect effect | 0.010 | -0.038 | 0.057 | 1.001 |
|  |  | Total effect | -0.041 | -0.169 | 0.085 | 1.002 |
|  |  | Indirect effect (%) | 172.635 | -434.221 | 434.308 | 1.000 |

Prefectures where NPIs were not implemented are represented by "-".

**Table S18. Effects of NPIs on mitigating the COVID-19 infection mediated by Osaka residents’ mobility during Phase 3**

| **Prefecture Number** | **Prefecture Name** | **Parameters** | **Mean** | **95% Credible Interval** | | **Rhat** |
| --- | --- | --- | --- | --- | --- | --- |
|  |  |  |  | **Low** | **High** |  |
| 1 | Hokkaido | a | -0.128 | -0.249 | -0.005 | 1.000 |
|  |  | b | 0.218 | -0.105 | 0.573 | 1.000 |
|  |  | c (Direct effect) | -0.181 | -0.313 | -0.048 | 1.000 |
|  |  | Indirect effect | -0.027 | -0.091 | 0.016 | 1.000 |
|  |  | Total effect | -0.207 | -0.331 | -0.085 | 1.001 |
|  |  | Indirect effect (%) | 14.530 | -9.350 | 55.880 | 1.000 |
| 2 | Aomori | a | -0.237 | -0.585 | 0.110 | 1.002 |
|  |  | b | 0.256 | 0.179 | 0.342 | 1.017 |
|  |  | c (Direct effect) | -0.009 | -0.195 | 0.176 | 1.001 |
|  |  | Indirect effect | -0.060 | -0.154 | 0.029 | 1.001 |
|  |  | Total effect | -0.069 | -0.270 | 0.133 | 1.001 |
|  |  | Indirect effect (%) | 174.489 | -620.854 | 679.000 | 1.000 |
| 3 | Iwate | a | - | - | - | - |
|  |  | b | - | - | - | - |
|  |  | c (Direct effect) | - | - | - | - |
|  |  | Indirect effect | - | - | - | - |
|  |  | Total effect | - | - | - | - |
|  |  | Indirect effect (%) | - | - | - | - |
| 4 | Miyagi | a | - | - | - | - |
|  |  | b | - | - | - | - |
|  |  | c (Direct effect) | - | - | - | - |
|  |  | Indirect effect | - | - | - | - |
|  |  | Total effect | - | - | - | - |
|  |  | Indirect effect (%) | - | - | - | - |
| 5 | Akita | a | - | - | - | - |
|  |  | b | - | - | - | - |
|  |  | c (Direct effect) | - | - | - | - |
|  |  | Indirect effect | - | - | - | - |
|  |  | Total effect | - | - | - | - |
|  |  | Indirect effect (%) | - | - | - | - |
| 6 | Yamagata | a | 0.543 | 0.302 | 0.836 | 1.062 |
|  |  | b | 0.593 | 0.460 | 0.718 | 1.047 |
|  |  | c (Direct effect) | -0.516 | -0.705 | -0.324 | 1.005 |
|  |  | Indirect effect | 0.323 | 0.163 | 0.521 | 1.028 |
|  |  | Total effect | -0.193 | -0.369 | 0.003 | 1.034 |
|  |  | Indirect effect (%) | -510.898 | -1213.734 | 370.482 | 1.000 |
| 7 | Fukushima | a | -0.309 | -0.557 | -0.061 | 1.000 |
|  |  | b | -0.064 | -0.189 | 0.060 | 1.007 |
|  |  | c (Direct effect) | -0.372 | -0.547 | -0.193 | 1.000 |
|  |  | Indirect effect | 0.020 | -0.018 | 0.075 | 1.005 |
|  |  | Total effect | -0.351 | -0.518 | -0.181 | 1.000 |
|  |  | Indirect effect (%) | -6.112 | -24.384 | 5.954 | 1.004 |
| 8 | Ibaraki | a | -0.102 | -0.257 | 0.041 | 1.021 |
|  |  | b | 0.525 | 0.427 | 0.612 | 1.045 |
|  |  | c (Direct effect) | -0.090 | -0.262 | 0.041 | 1.136 |
|  |  | Indirect effect | -0.054 | -0.135 | 0.022 | 1.022 |
|  |  | Total effect | -0.143 | -0.335 | 0.006 | 1.121 |
|  |  | Indirect effect (%) | 42.313 | -40.473 | 145.228 | 1.001 |
| 9 | Tochigi | a | -0.249 | -0.433 | -0.054 | 1.000 |
|  |  | b | -0.155 | -0.529 | 0.109 | 1.003 |
|  |  | c (Direct effect) | -0.297 | -0.464 | -0.152 | 1.001 |
|  |  | Indirect effect | 0.043 | -0.025 | 0.174 | 1.003 |
|  |  | Total effect | -0.254 | -0.371 | -0.137 | 1.000 |
|  |  | Indirect effect (%) | -17.919 | -76.996 | 10.585 | 1.002 |
| 10 | Gunma | a | -0.339 | -0.477 | -0.187 | 1.017 |
|  |  | b | 0.406 | -0.804 | 1.279 | 1.601 |
|  |  | c (Direct effect) | -0.030 | -0.444 | 0.344 | 1.425 |
|  |  | Indirect effect | -0.136 | -0.498 | 0.273 | 1.492 |
|  |  | Total effect | -0.166 | -0.287 | -0.050 | 1.003 |
|  |  | Indirect effect (%) | 95.462 | -198.601 | 459.129 | 1.002 |
| 11 | Saitama | a | -0.004 | -0.178 | 0.174 | 1.002 |
|  |  | b | 0.470 | 0.402 | 0.551 | 1.016 |
|  |  | c (Direct effect) | -0.278 | -0.391 | -0.164 | 1.003 |
|  |  | Indirect effect | -0.002 | -0.084 | 0.082 | 1.002 |
|  |  | Total effect | -0.280 | -0.419 | -0.142 | 1.002 |
|  |  | Indirect effect (%) | -2.147 | -46.087 | 25.623 | 1.002 |
| 12 | Chiba | a | -0.058 | -0.174 | 0.061 | 1.001 |
|  |  | b | 0.638 | 0.417 | 0.929 | 1.004 |
|  |  | c (Direct effect) | -0.285 | -0.419 | -0.148 | 1.001 |
|  |  | Indirect effect | -0.039 | -0.135 | 0.034 | 1.001 |
|  |  | Total effect | -0.324 | -0.452 | -0.196 | 1.001 |
|  |  | Indirect effect (%) | 11.869 | -12.730 | 40.956 | 1.001 |
| 13 | Tokyo | a | 0.050 | -0.017 | 0.119 | 1.002 |
|  |  | b | 0.173 | -0.006 | 0.351 | 1.009 |
|  |  | c (Direct effect) | -0.313 | -0.430 | -0.200 | 1.002 |
|  |  | Indirect effect | 0.009 | -0.003 | 0.028 | 1.002 |
|  |  | Total effect | -0.304 | -0.421 | -0.190 | 1.002 |
|  |  | Indirect effect (%) | -3.034 | -10.894 | 1.045 | 1.002 |
| 14 | Kanagawa | a | -0.047 | -0.196 | 0.101 | 1.002 |
|  |  | b | 0.494 | 0.346 | 0.691 | 1.005 |
|  |  | c (Direct effect) | -0.305 | -0.438 | -0.167 | 1.001 |
|  |  | Indirect effect | -0.025 | -0.111 | 0.046 | 1.003 |
|  |  | Total effect | -0.330 | -0.463 | -0.196 | 1.000 |
|  |  | Indirect effect (%) | 7.109 | -17.355 | 34.251 | 1.003 |
| 15 | Niigata | a | -0.101 | -0.362 | 0.161 | 1.001 |
|  |  | b | 0.358 | 0.247 | 0.503 | 1.004 |
|  |  | c (Direct effect) | -0.295 | -0.443 | -0.144 | 1.001 |
|  |  | Indirect effect | -0.038 | -0.145 | 0.054 | 1.001 |
|  |  | Total effect | -0.333 | -0.483 | -0.185 | 1.002 |
|  |  | Indirect effect (%) | 10.804 | -20.707 | 42.623 | 1.000 |
| 16 | Toyama | a | - | - | - | - |
|  |  | b | - | - | - | - |
|  |  | c (Direct effect) | - | - | - | - |
|  |  | Indirect effect | - | - | - | - |
|  |  | Total effect | - | - | - | - |
|  |  | Indirect effect (%) | - | - | - | - |
| 17 | Ishikawa | a | -0.287 | -0.394 | -0.180 | 1.015 |
|  |  | b | -11.654 | -51.313 | 31.230 | 1.015 |
|  |  | c (Direct effect) | -3.826 | -15.432 | 8.867 | 1.014 |
|  |  | Indirect effect | 3.350 | -9.340 | 14.953 | 1.014 |
|  |  | Total effect | -0.476 | -0.631 | -0.321 | 1.001 |
|  |  | Indirect effect (%) | -723.866 | -3218.492 | 1962.925 | 1.014 |
| 18 | Fukui | a | - | - | - | - |
|  |  | b | - | - | - | - |
|  |  | c (Direct effect) | - | - | - | - |
|  |  | Indirect effect | - | - | - | - |
|  |  | Total effect | - | - | - | - |
|  |  | Indirect effect (%) | - | - | - | - |
| 19 | Yamanashi | a | - | - | - | - |
|  |  | b | - | - | - | - |
|  |  | c (Direct effect) | - | - | - | - |
|  |  | Indirect effect | - | - | - | - |
|  |  | Total effect | - | - | - | - |
|  |  | Indirect effect (%) | - | - | - | - |
| 20 | Nagano | a | -0.075 | -0.436 | 0.294 | 1.000 |
|  |  | b | 0.130 | 0.094 | 0.170 | 1.002 |
|  |  | c (Direct effect) | -0.237 | -0.361 | -0.112 | 1.000 |
|  |  | Indirect effect | -0.010 | -0.058 | 0.038 | 1.000 |
|  |  | Total effect | -0.246 | -0.378 | -0.114 | 1.000 |
|  |  | Indirect effect (%) | 2.952 | -21.754 | 23.194 | 1.000 |
| 21 | Gifu | a | -0.006 | -0.211 | 0.197 | 1.000 |
|  |  | b | 1.141 | 0.831 | 1.530 | 1.001 |
|  |  | c (Direct effect) | -0.430 | -0.641 | -0.214 | 1.000 |
|  |  | Indirect effect | -0.004 | -0.229 | 0.239 | 1.000 |
|  |  | Total effect | -0.433 | -0.676 | -0.182 | 1.000 |
|  |  | Indirect effect (%) | -6.353 | -101.082 | 43.916 | 1.000 |
| 22 | Shizuoka | a | -0.120 | -0.290 | 0.057 | 1.004 |
|  |  | b | 0.741 | 0.580 | 0.941 | 1.006 |
|  |  | c (Direct effect) | -0.188 | -0.344 | -0.029 | 1.002 |
|  |  | Indirect effect | -0.089 | -0.223 | 0.042 | 1.004 |
|  |  | Total effect | -0.277 | -0.452 | -0.100 | 1.002 |
|  |  | Indirect effect (%) | 31.285 | -23.654 | 83.670 | 1.000 |
| 23 | Aichi | a | -0.151 | -0.228 | -0.063 | 1.002 |
|  |  | b | 1.683 | 1.102 | 2.483 | 1.017 |
|  |  | c (Direct effect) | -0.069 | -0.273 | 0.171 | 1.007 |
|  |  | Indirect effect | -0.259 | -0.484 | -0.084 | 1.008 |
|  |  | Total effect | -0.328 | -0.462 | -0.190 | 1.001 |
|  |  | Indirect effect (%) | 81.688 | 26.467 | 166.525 | 1.007 |
| 24 | Mie | a | -0.288 | -0.478 | -0.083 | 1.003 |
|  |  | b | 0.853 | 0.637 | 1.137 | 1.001 |
|  |  | c (Direct effect) | 0.051 | -0.137 | 0.268 | 1.001 |
|  |  | Indirect effect | -0.250 | -0.466 | -0.062 | 1.003 |
|  |  | Total effect | -0.198 | -0.355 | -0.038 | 1.003 |
|  |  | Indirect effect (%) | 151.864 | 35.540 | 434.000 | 1.000 |
| 25 | Shiga | a | - | - | - | - |
|  |  | b | - | - | - | - |
|  |  | c (Direct effect) | - | - | - | - |
|  |  | Indirect effect | - | - | - | - |
|  |  | Total effect | - | - | - | - |
|  |  | Indirect effect (%) | - | - | - | - |
| 26 | Kyoto | a | -0.054 | -0.077 | -0.031 | 1.003 |
|  |  | b | 1.461 | 0.577 | 2.328 | 1.014 |
|  |  | c (Direct effect) | -0.204 | -0.314 | -0.094 | 1.004 |
|  |  | Indirect effect | -0.080 | -0.148 | -0.027 | 1.014 |
|  |  | Total effect | -0.284 | -0.381 | -0.187 | 1.002 |
|  |  | Indirect effect (%) | 28.831 | 9.212 | 57.098 | 1.009 |
| 27 | Osaka | a | 0.003 | -0.003 | 0.010 | 1.013 |
|  |  | b | -17.090 | -22.750 | -12.527 | 1.015 |
|  |  | c (Direct effect) | -0.196 | -0.306 | -0.071 | 1.013 |
|  |  | Indirect effect | -0.054 | -0.175 | 0.047 | 1.012 |
|  |  | Total effect | -0.250 | -0.358 | -0.138 | 1.001 |
|  |  | Indirect effect (%) | 20.182 | -25.889 | 66.245 | 1.009 |
| 28 | Hyogo | a | -0.095 | -0.114 | -0.070 | 1.015 |
|  |  | b | 17.548 | -4.817 | 54.847 | 1.048 |
|  |  | c (Direct effect) | 1.418 | -0.790 | 5.210 | 1.047 |
|  |  | Indirect effect | -1.703 | -5.494 | 0.476 | 1.048 |
|  |  | Total effect | -0.285 | -0.424 | -0.146 | 1.001 |
|  |  | Indirect effect (%) | 644.904 | -154.875 | 2210.606 | 1.038 |
| 29 | Nara | a | - | - | - | - |
|  |  | b | - | - | - | - |
|  |  | c (Direct effect) | - | - | - | - |
|  |  | Indirect effect | - | - | - | - |
|  |  | Total effect | - | - | - | - |
|  |  | Indirect effect (%) | - | - | - | - |
| 30 | Wakayama | a | -0.039 | -0.159 | 0.086 | 1.002 |
|  |  | b | 1.372 | 1.042 | 1.771 | 1.000 |
|  |  | c (Direct effect) | -0.020 | -0.185 | 0.151 | 1.001 |
|  |  | Indirect effect | -0.055 | -0.232 | 0.112 | 1.002 |
|  |  | Total effect | -0.075 | -0.229 | 0.080 | 1.001 |
|  |  | Indirect effect (%) | 128.460 | -699.387 | 872.989 | 1.000 |
| 31 | Tottori | a | - | - | - | - |
|  |  | b | - | - | - | - |
|  |  | c (Direct effect) | - | - | - | - |
|  |  | Indirect effect | - | - | - | - |
|  |  | Total effect | - | - | - | - |
|  |  | Indirect effect (%) | - | - | - | - |
| 32 | Shimane | a | 0.029 | -0.286 | 0.349 | 1.000 |
|  |  | b | 0.269 | 0.122 | 0.450 | 1.000 |
|  |  | c (Direct effect) | -0.366 | -0.582 | -0.144 | 1.000 |
|  |  | Indirect effect | 0.006 | -0.091 | 0.095 | 1.000 |
|  |  | Total effect | -0.360 | -0.577 | -0.144 | 1.000 |
|  |  | Indirect effect (%) | -2.951 | -39.881 | 28.119 | 1.000 |
| 33 | Okayama | a | -0.032 | -0.238 | 0.182 | 1.000 |
|  |  | b | 0.902 | 0.724 | 1.095 | 1.003 |
|  |  | c (Direct effect) | -0.067 | -0.234 | 0.104 | 1.000 |
|  |  | Indirect effect | -0.029 | -0.219 | 0.165 | 1.000 |
|  |  | Total effect | -0.095 | -0.314 | 0.121 | 1.000 |
|  |  | Indirect effect (%) | -1072.769 | -554.755 | 633.235 | 1.000 |
| 34 | Hiroshima | a | -0.620 | -0.811 | -0.437 | 1.000 |
|  |  | b | 0.886 | 0.596 | 1.237 | 1.001 |
|  |  | c (Direct effect) | -0.213 | -0.441 | 0.042 | 1.001 |
|  |  | Indirect effect | -0.545 | -0.790 | -0.348 | 1.001 |
|  |  | Total effect | -0.758 | -0.936 | -0.581 | 1.000 |
|  |  | Indirect effect (%) | 72.482 | 46.579 | 106.064 | 1.001 |
| 35 | Yamaguchi | a | -0.665 | -0.964 | -0.384 | 1.018 |
|  |  | b | 0.308 | 0.193 | 0.452 | 1.063 |
|  |  | c (Direct effect) | -0.153 | -0.284 | -0.017 | 1.016 |
|  |  | Indirect effect | -0.202 | -0.320 | -0.109 | 1.027 |
|  |  | Total effect | -0.355 | -0.486 | -0.238 | 1.004 |
|  |  | Indirect effect (%) | 57.921 | 31.699 | 94.007 | 1.023 |
| 36 | Tokushima | a | - | - | - | - |
|  |  | b | - | - | - | - |
|  |  | c (Direct effect) | - | - | - | - |
|  |  | Indirect effect | - | - | - | - |
|  |  | Total effect | - | - | - | - |
|  |  | Indirect effect (%) | - | - | - | - |
| 37 | Kagawa | a | -0.263 | -0.516 | -0.015 | 1.008 |
|  |  | b | 0.358 | 0.275 | 0.440 | 1.097 |
|  |  | c (Direct effect) | -0.083 | -0.205 | 0.021 | 1.028 |
|  |  | Indirect effect | -0.094 | -0.189 | -0.005 | 1.004 |
|  |  | Total effect | -0.178 | -0.315 | -0.055 | 1.029 |
|  |  | Indirect effect (%) | 58.672 | 3.509 | 120.632 | 1.000 |
| 38 | Ehime | a | - | - | - | - |
|  |  | b | - | - | - | - |
|  |  | c (Direct effect) | - | - | - | - |
|  |  | Indirect effect | - | - | - | - |
|  |  | Total effect | - | - | - | - |
|  |  | Indirect effect (%) | - | - | - | - |
| 39 | Kochi | a | -0.043 | -0.321 | 0.241 | 1.001 |
|  |  | b | 0.432 | 0.349 | 0.517 | 1.001 |
|  |  | c (Direct effect) | -0.001 | -0.118 | 0.118 | 1.000 |
|  |  | Indirect effect | -0.019 | -0.139 | 0.103 | 1.001 |
|  |  | Total effect | -0.020 | -0.162 | 0.123 | 1.001 |
|  |  | Indirect effect (%) | 121.089 | -770.306 | 929.756 | 1.000 |
| 40 | Fukuoka | a | -0.311 | -0.460 | -0.024 | 1.698 |
|  |  | b | 12.568 | 0.487 | 89.139 | 1.154 |
|  |  | c (Direct effect) | 4.112 | -0.313 | 30.237 | 1.148 |
|  |  | Indirect effect | -4.429 | -30.560 | -0.005 | 1.149 |
|  |  | Total effect | -0.317 | -0.489 | -0.141 | 1.033 |
|  |  | Indirect effect (%) | 1396.577 | 1.882 | 9182.203 | 1.010 |
| 41 | Saga | a | -0.222 | -0.550 | 0.109 | 1.000 |
|  |  | b | 0.358 | 0.272 | 0.461 | 1.002 |
|  |  | c (Direct effect) | 0.004 | -0.143 | 0.155 | 1.000 |
|  |  | Indirect effect | -0.080 | -0.204 | 0.039 | 1.000 |
|  |  | Total effect | -0.075 | -0.245 | 0.094 | 1.000 |
|  |  | Indirect effect (%) | 13.095 | -690.319 | 830.027 | 1.000 |
| 42 | Nagasaki | a | -0.288 | -0.608 | 0.026 | 1.001 |
|  |  | b | 0.349 | 0.292 | 0.417 | 1.006 |
|  |  | c (Direct effect) | -0.206 | -0.357 | -0.050 | 1.001 |
|  |  | Indirect effect | -0.101 | -0.220 | 0.009 | 1.001 |
|  |  | Total effect | -0.307 | -0.490 | -0.123 | 1.000 |
|  |  | Indirect effect (%) | 32.728 | -4.450 | 72.331 | 1.000 |
| 43 | Kumamoto | a | -0.483 | -0.744 | -0.216 | 1.000 |
|  |  | b | 0.941 | 0.726 | 1.168 | 1.005 |
|  |  | c (Direct effect) | -0.015 | -0.293 | 0.280 | 1.002 |
|  |  | Indirect effect | -0.458 | -0.765 | -0.186 | 1.001 |
|  |  | Total effect | -0.473 | -0.746 | -0.198 | 1.001 |
|  |  | Indirect effect (%) | 103.016 | 47.114 | 195.747 | 1.000 |
| 44 | Oita | a | -0.017 | -0.314 | 0.282 | 1.001 |
|  |  | b | 0.614 | 0.484 | 0.747 | 1.010 |
|  |  | c (Direct effect) | -0.226 | -0.413 | -0.038 | 1.000 |
|  |  | Indirect effect | -0.012 | -0.200 | 0.169 | 1.001 |
|  |  | Total effect | -0.238 | -0.466 | -0.008 | 1.000 |
|  |  | Indirect effect (%) | -109.227 | -256.526 | 98.487 | 1.000 |
| 45 | Miyazaki | a | 0.087 | -0.189 | 0.361 | 1.002 |
|  |  | b | 0.520 | 0.422 | 0.654 | 1.019 |
|  |  | c (Direct effect) | -0.407 | -0.581 | -0.233 | 1.007 |
|  |  | Indirect effect | 0.045 | -0.100 | 0.191 | 1.003 |
|  |  | Total effect | -0.362 | -0.571 | -0.150 | 1.007 |
|  |  | Indirect effect (%) | -18.069 | -101.639 | 22.008 | 1.000 |
| 46 | Kagoshima | a | -0.251 | -0.536 | 0.029 | 1.005 |
|  |  | b | 0.471 | 0.391 | 0.550 | 1.002 |
|  |  | c (Direct effect) | -0.136 | -0.311 | 0.037 | 1.001 |
|  |  | Indirect effect | -0.118 | -0.266 | 0.014 | 1.006 |
|  |  | Total effect | -0.254 | -0.464 | -0.039 | 1.001 |
|  |  | Indirect effect (%) | 50.677 | -12.199 | 129.709 | 1.000 |
| 47 | Okinawa | a | -0.654 | -0.858 | -0.453 | 1.002 |
|  |  | b | 0.582 | 0.449 | 0.758 | 1.008 |
|  |  | c (Direct effect) | -0.445 | -0.622 | -0.234 | 1.008 |
|  |  | Indirect effect | -0.384 | -0.597 | -0.227 | 1.006 |
|  |  | Total effect | -0.828 | -0.985 | -0.672 | 1.001 |
|  |  | Indirect effect (%) | 46.361 | 28.923 | 70.426 | 1.008 |

Prefectures where NPIs were not implemented are represented by "-".
